# STREMI: a dual-function upstream ORF-encoded regulator of mitochondrial cristae architecture

**DOI:** 10.1038/s44319-026-00783-8

**Published:** 2026-05-02

**Authors:** Ruiyang Guo, Yabo Guo, Runguo Shu, Jiajia Qian, Jiawei Wang, Ruobing Li, Ti Qin, Ziyi Wang, Hongtao Tian, Mengchen Wu, Long Zhou, Xiaogang Guo, Shan Zhang

**Affiliations:** 1 Department of Biochemistry, Department of Cardiology of The First Affiliated Hospital, Zhejiang University School of Medicine, Hangzhou, China; 2 Department of Cardiology, The First Affiliated Hospital, Zhejiang University School of Medicine, Hangzhou, China; 3 Department of Biophysics and Department of Critical Care Medicine of Sir Run Run Shaw Hospital, Zhejiang University School of Medicine, Hangzhou, China

**Keywords:** Evolution & Ecology, Translation & Protein Quality

## Abstract

Eukaryotic mRNAs typically encode a single functional polypeptide, a principle challenged by the discovery of widespread non-canonical peptide-coding ORFs within 5’UTRs. However, their functional significance at the protein level remains underexplored. Using a four-layered pipeline, we identify 14 human transcripts predominantly transcribed in polycistronic forms, each encoding two conserved proteins. Focusing on the *SLC35A4* transcript, we show that its 5’UTR encodes a mitochondrial inner membrane-localized microprotein that we name STREMI (SLC35A4 stress response regulating MICOS interactor). Sharing topology and motifs with the MICOS core subunit MIC10, STREMI regulates mitochondrial cristae morphogenesis in mice and human cells. Additionally, the STREMI-encoding uORF mediates stress-responsive translation of *SLC35A4*—a Golgi nucleotide sugar transporter—upregulating its translation during the integrated stress response. Evolutionary analyses indicate that these bicistronic transcripts likely arose through transcriptional readthrough following retroposition. We propose a mechanism of “gene symbiosis” that enables functional partitioning and coordinated translation of protein pairs from bicistronic transcripts.

## Synopsis

The *SLC35A4* uORF encodes a microprotein that regulates mitochondrial cristae architecture through interaction with the MICOS complex. The uORF and downstream main ORF of this bicistronic transcript likely evolved independently and were subsequently unified by transcriptional readthrough following retroposition.

- Systematic identification of evolutionarily constrained human transcripts with dual protein-coding capacity.
- The *SLC35A4* uORF encodes the mitochondrial microprotein STREMI while simultaneously acting as a cis-regulator of downstream translation.
- STREMI maintains cristae architecture by associating with the MICOS subunit MIC10 through a conserved sequence motif.
- The “gene symbiosis” model explains the emergence of stable bicistronic transcripts and functional partitioning in vertebrates.

## Abstract (web summary)

The *SLC35A4* uORF encodes a microprotein that regulates mitochondrial cristae architecture through interaction with the MICOS complex. The uORF and downstream main ORF of this bicistronic transcript likely evolved independently and were subsequently unified by transcriptional readthrough following retroposition.

## Graphical abstract

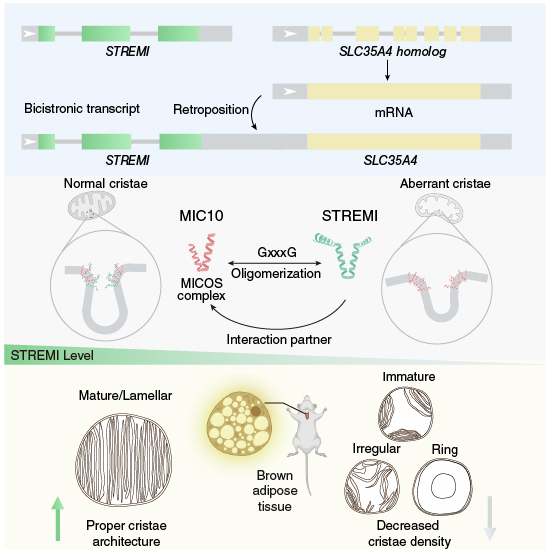

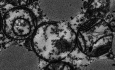

## Introduction

The foundational model of eukaryotic gene expression posits that each mRNA encodes a single polypeptide, a principle governed by the mechanisms of translation initiation and termination(Brito Querido et al, 2024; Schuller and Green, 2018). In this framework, the pre-initiation complex is recruited to the 5’ cap of the mRNA, from where it scans the 5’ untranslated region (5’UTR) to locate the start codon, typically nestled within an optimal Kozak sequence. When translation reaches a stop codon, the elongating ribosome recruits release factors, culminating in peptide release and ribosomal dissociation. This linear, cap-dependent process underpins the view that eukaryotic transcripts are inherently monocistronic—encoding one protein per mRNA—in contrast to the polycistronic architecture often observed in prokaryotes (Kozak, 1999).

However, this long-standing paradigm is increasingly contested by the discovery of non-canonical open reading frames (ORFs) within the 5’UTRs of actively translating mRNAs (Ingolia et al, 2014; Lee et al, 2012). The emergence of ribosome profiling, a technique that quantifies protein synthesis by sequencing ribosome-protected mRNA fragments with single-nucleotide precision, has been instrumental in revealing these upstream ORFs (uORFs) (Ingolia et al, 2019). The ribosome occupancy of many uORFs mirrors that of the downstream canonical ORFs, with 20 ~ 30% of human transcripts harbor translating uORFs, challenging the monocistronic dogma (Chen et al, 2020; Chothani et al, 2022).

While ribosome profiling, corroborated by peptidomics, confirms the translation of uORFs, the functional significance of their encoded peptides remains debated. Unlike canonical proteins, uORF-derived peptides exhibit limited evolutionary conservation or signatures of purifying selection at the protein sequence level (Sandmann et al, 2023; Vakirlis et al, 2022). Moreover, our work and that of others reveal that many of these novel peptides are inherently unstable post-translation, casting doubt on their roles as standalone functional entities (Prensner et al, 2021; Yang et al, 2024; Zhang et al, 2020). To date, only a few human uORFs have been experimentally validated as encoding biologically active polypeptides (Hofman et al, 2024; Huang et al, 2021; Jayaram et al, 2021; Rathore et al, 2018; Rensvold et al, 2022). Among these, the 5’UTR of *SLC35A4* was first reported in 2013 to encode a 103-amino acid (aa) microprotein and was designated PE1 by the Human Proteome Project in 2018(Omenn et al, 2018; Vanderperre et al, 2013). Our group subsequently identified this polypeptide as a mitochondrially localized microprotein, a finding later corroborated by multiple independent studies (Ajala et al, 2025; Liu et al, 2024; Yang et al, 2024; Zhang et al, 2020). More recent work has implicated this microprotein in maintaining mitochondrial architecture, oxidative stress responses, and respiratory function in both cultured cells and mouse models (Ajala et al, 2025; Rocha et al, 2024; Rocha et al, 2025). In mice, its deficiency alters mitochondrial lipid composition under a high-fat diet and impairs fatty acid oxidation (Rocha et al, 2025). Despite this growing interest, the precise molecular function of this uORF-encoded microprotein remains unresolved. Moreover, the functional and regulatory interplay between the upstream and downstream ORFs—and their evolutionary origin—has not been systematically addressed, leaving the broader biological significance of this protein-coding uORF unclear.

To address these unresolved questions, we leveraged long-read RNA sequencing to systematically identify bona fide bicistronic transcripts in humans. Applying rigorous criteria, we identified 14 high-confidence pairs of functional proteins encoded within single mRNAs. Focusing on the *SLC35A4* transcript, we confirmed the endogenous expression of a uORF-encoded peptide and elucidated its role in modulating mitochondrial cristae architecture. Beyond this function in mitochondria, the uORF mediates a stress-responsive activation of its downstream ORF, unveiling a delicate regulatory axis. By reconstructing the evolutionary history of these bicistronic transcripts, we discerned a distinctive “bipartite origin, bicistronic transcription” pattern, shedding light on the genesis of human bicistronic transcripts and their adaptive significance.

## Results

### Identification of human transcripts with dual protein coding regions

**Figure 1:**
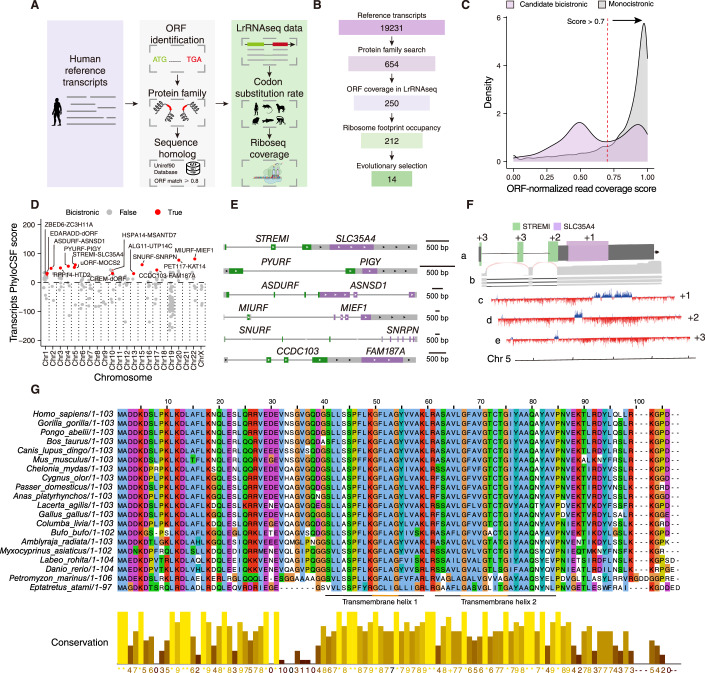
Identification of human transcripts with multiple protein coding regions. (**A**) Schematic workflow for identifying bona fide bicistronic transcripts from the Ensembl human canonical transcript dataset. (**B**) Number of transcripts meeting each sequential selection criterion. (**C**) Distribution of weighted ORF coverage scores derived from long-read RNA sequencing data (Dataset EV1). (**D**) Average PhyloCSF score of protein-coding ORFs in bicistronic transcript candidate, grouped by chromosomal location in the human genome. (**E**) Representative gene structure of high-confidence human bicistronic transcripts. (**F**) Genomic track view of a polycistronic locus in humans, with tracks displayed as follows: a Gene structure: ORFs (*STREMI* in green, *SLC35A4* in purple). b Read coverage from raw long-read RNA sequencing data. c–e PhyloCSF tracks depicting scores for the three forward-strand reading frames ( + 1, +2, +3). (**G**) Protein sequence alignment of STREMI homologs across species. Source data are available online for this figure.

**Figure EV1:**
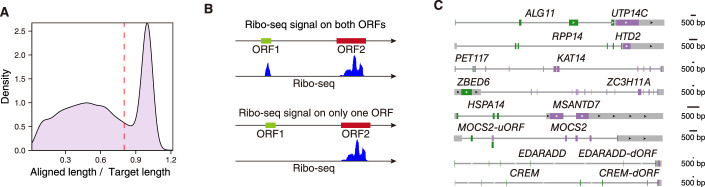
Identification of human transcripts with multiple protein coding regions. (**A**) Distribution of the ratio of aligned length to full length of the closest UniRef90 homolog among proteins with positive InterProScan annotations. (**B**) Schematic illustrating examples of candidate bicistronic transcripts that either pass (top) or fail (bottom) Ribo-seq signal screening. (**C**) Gene structures of remaining identified human bicistronic transcripts, corresponding to Fig. 1E.

To discern human transcripts that encode at least two distinct functional proteins, we developed a proteogenomics pipeline based on evidence of transcription and translation (Fig. 1A). First, we extracted amino acid sequences from all putative protein-coding ORFs (≥ 30 codons) within human reference transcripts. These sequences were searched using InterProScan, a comprehensive protein signature database (Paysan-Lafosse et al, 2023). Next, sequence homology searches against protein databases (UniRef90) were performed to determine the closest homologs in annotated proteome. As many ORFs encode only truncated portions of canonical proteins, we prioritized those exhibiting substantial sequence overlap ( ≥ 80%) with their closest identified homologs (Figs. 1B and EV1A). Given the possibility that single-coding sequence (CDS) isoforms and polycistronic transcripts may be produced simultaneously, we analyzed long-read RNA sequencing data to identify transcripts predominantly expressed in the polycistronic form (Fig. 1C) (Sloan et al, 2016). In total, we identified 250 candidate human polycistronic transcripts, with 87% classified as bicistronic (Dataset EV1).

To further refine our dataset, we implemented two additional criteria to identify functional polycistronic transcripts with active protein translation. First, we filtered ORFs based on ribosome footprint occupancy, a direct measure of translational activity (Fig. EV1B). Additionally, we selected CDSs that are undergoing purifying selection by analyzing aligned genome sequences from 100 vertebrate species (Fig. 1D) (Lin et al, 2011; Pockrandt et al, 2022). Ultimately, we identified 14 human reference transcripts that may encode two functional proteins (Figs. 1E and EV1C; Dataset EV1). Notably, several previously characterized bicistronic transcripts, including *MIURF*-*MIEF1*, *PYURF*-*PIGY* and *ASDURF*-*ASNSD1*, were successfully retrieved (Fig. 1E) (Hofman et al, 2024; Rathore et al, 2018; Rensvold et al, 2022).

Among transcripts that passed our stringent filters, the transcript of *SLC35A4* is predicted to encode a 103-amino acid microprotein in addition to the classical CDS (Fig. 1F), initially identified as a uORF-encoded peptide using mass spectrometry (Vanderperre et al, 2013). This uORF-encoded microprotein (also referred to as SLC35A4-MP or AltSLC35A4 in the literature) is well-conserved across vertebrates and displays strong evolutionary constraint on amino acid changes (evident by positive PhyloCSF scores) in genome alignments of vertebrates (Fig. 1F,G) (Ajala et al, 2025; Rocha et al, 2024; Rocha et al, 2025). Notably, translation of the uORF has been reported to be stress-responsive, making it a promising candidate for studying the potential functional interplay between upstream and downstream ORFs (Andreev et al, 2015). Subsequent studies—including our own—have confirmed mitochondrial localization of the uORF-encoded peptide (Ajala et al, 2025; Rocha et al, 2024; Yang et al, 2024; Zhang et al, 2020). However, its precise submitochondrial position and topology remain unresolved, with evidence supporting both outer- and inner-membrane association (Rocha et al, 2024; Yang et al, 2024). Despite repeated investigation, an integrated mechanistic model for its mitochondrial function is still lacking, and the evolutionary origin of this bicistronic architecture remains unclear. These gaps motivated us to focus our subsequent analyses on the *SLC35A4* transcript.

### The 5’UTR of *SLC35A4* encodes a mitochondrial microprotein required for mitochondrial cristae morphogenesis

**Figure 2:**
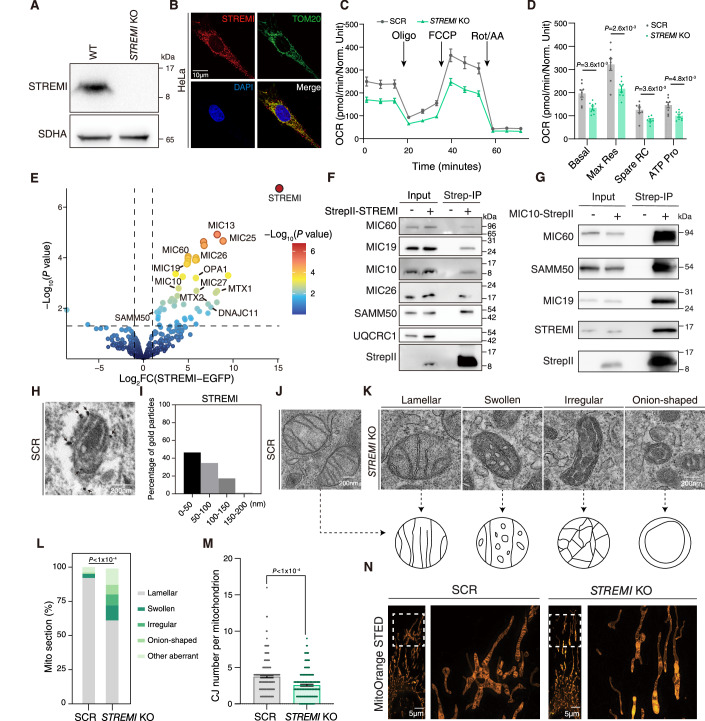
The 5’UTR of *SLC35A4* encodes a mitochondrial microprotein required for mitochondrial cristae morphogenesis. (**A**) Detection of endogenous STREMI in HEK293T lysates using a rabbit polyclonal antibody against STREMI N-terminal residues 2–13. (**B**) Immunostaining of STREMI and the mitochondrial marker TOM20 in transfected HeLa cells. Scale bar, 10 µm. (**C**) Oxygen consumption rate (OCR) measured in scramble (SCR) control and *STREMI* knockout (KO) HEK293T cells during a mitochondrial stress test using a Seahorse XFe96 analyzer. Treatments: oligomycin (Oligo), carbonyl cyanide-4-(trifluoromethoxy)phenylhydrazone (FCCP), rotenone/antimycin A (Rot/AA). Data are presented as mean ± SEM (*n* = 8). (**D**) Basal respiration, maximal respiration (Max Res), spare respiratory capacity (Spare RC), and ATP production (ATP Pro) of SCR control and *STREMI* KO cells measured in the mitochondrial stress test. Data are presented as mean ± SEM (*n* = 8). Basal respiration (*P* = 3.6 × 10^−^^3^), Max Res (*P* = 2.6 ×  10^−^^3^), Spare RC (*P* = 3.6 × 10^−^^3^), and ATP Pro (*P* = 4.8 × 10^−^^3^). *P* values are from two-tailed unpaired *t* tests. (**E**) Volcano plot of proteins enriched in STREMI immunoprecipitation (IP) versus EGFP control, identified by mass spectrometry (MS) (*n* = 3). *P* values are from two-tailed unpaired *t* tests and Benjamini–Hochberg-adjusted. (**F**) Immunoblot analysis of interactions between MICOS subunits and StrepII-STREMI following immunoprecipitation via the StrepII tag. (**G**) StrepII-tagged MIC10 was immunoprecipitated from HEK293T cell mitochondria, followed by immunoblotting of MICOS subunits and STREMI. (**H**) Immunogold detection of endogenous STREMI in HeLa cells. Scale bar, 200 nm. The arrows point to the position of gold particle. (**I**) The histograms show the fraction of gold particles within the indicated distance to the crista junction in nanometers in HeLa cells. (**J**, **K**) Representative transmission electron microscopy (TEM) images of mitochondria from SCR control (**J**) and *STREMI* KO (**K**) HeLa cells. (**L**) Quantitative analysis of mitochondrial cristae morphology in SCR control and *STREMI* KO HeLa cells. Bar chart depicts the percentage of cristae classified as (1) lamellar, (2) swollen, (3) irregular, (4) onion-shaped, or (5) other aberrant shapes, based on TEM (*n* = 237 mitochondria for scramble control; *n* = 265 mitochondria for *STREMI* KO, 5 biological replicates). *P* values were calculated using Fisher’s exact test based on raw counts (*P* < 1 × 10^−^⁴). (**M**) Cristae junction (CJ) abundance in SCR control and *STREMI* KO HeLa cells, quantified by TEM. Data are presented as mean ± SEM (*n* = 228 mitochondria for scramble control; *n* = 213 mitochondria s for *STREMI* KO, 5 biological replicates). *P* values from two-tailed unpaired *t* tests (*P* < 1 × 10^−^⁴). (**N**) Stimulated emission depletion (STED) nanoscopy of live HeLa cells stained with PK Mito-Orange dye. Scale bar, 5 μm. Source data are available online for this figure.

**Figure EV2:**
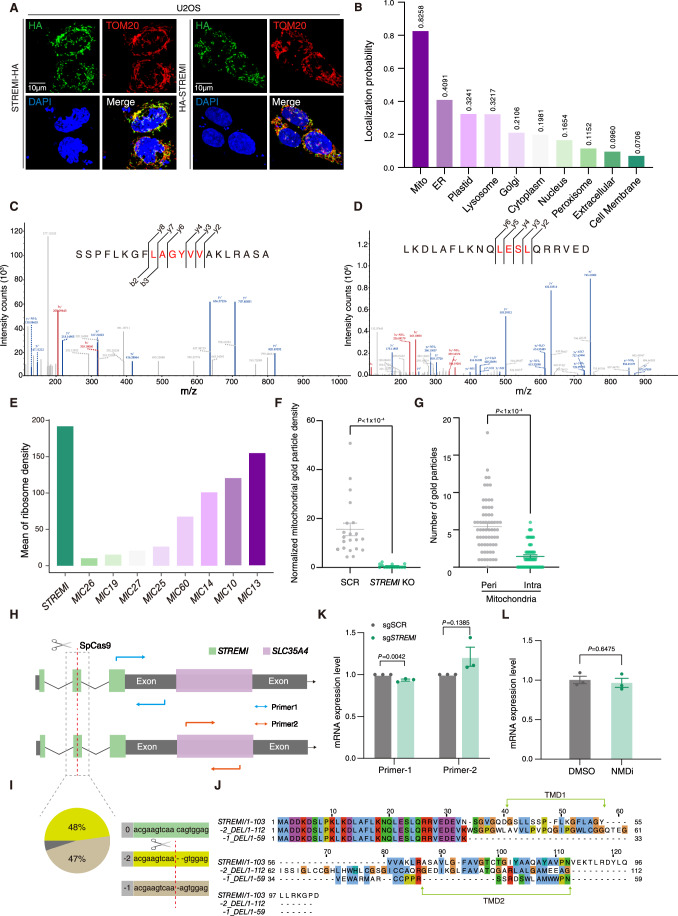
The 5’ UTR of *SLC35A4* encodes a mitochondrial microprotein required for mitochondrial cristae morphogenesis. (**A**) Immunofluorescence analysis of U2OS cells overexpressing N-terminally HA-tagged STREMI (HA-STREMI) or C-terminally HA-tagged STREMI (STREMI-HA), co-stained with mitochondrial marker TOM20. Scale bar, 10 µm. (**B**) Subcellular localization of STREMI predicted by DeepLoc 2.0. (**C**, **D**) MS/MS spectra of STREMI derived peptides identified in immunoprecipitation of Strep-tagged MIC10, a core MICOS subunit, in HEK293T cells via mass spectrometry analysis. (**E**) Ribosome occupancy across coding sequences (CDS) of *STREMI* and MICOS subunits. Mean ribosome density is shown for *STREMI* and subunits of MICOS. Ribosome footprints were extracted from the GWIPS-viz track of UCSC genome browser. (**F**) Mitochondrial gold particle density normalized to non-mitochondrial regions in SCR and *STREMI* KO HeLa cells. Data are presented as mean ± SEM (*n* = 21 mitochondria for SCR; *n* = 15 mitochondria for *STREMI* KO). *P* values are from unpaired two-tailed t tests (*P* < 1 × 10^−^^4^). (**G**) Quantification of gold particles in intra-mitochondrial and peri-mitochondrial regions. The peri-mitochondrial region was defined as the 50-nm zone surrounding the outer mitochondrial membrane. Data are presented as mean ± SEM (*n* = 65 mitochondria). *P* values are from paired two-tailed *t* tests (*P* < 1 × 10^−^^4^). (**H**) Schematic diagram showing the locations of qPCR primer pairs used to assess transcript abundance. (**I**) Distribution of genome-editing outcomes following SpCas9 targeting of the *STREMI* coding sequence. The frequencies of the two predominant frameshift alleles (−2 and −1) are indicated. (**J**) Predicted translation products from WT and the two predominant frameshift alleles in the *STREMI* KO line, shown as a sequence alignment. (**K**) qPCR analysis of transcript levels in SCR and *STREMI* KO cells. Data are presented as mean ± SEM (*n* = 3). *P* values are from two-tailed unpaired *t* tests: Primer 1 (*P* = 0.0042), Primer 2 (*P* = 0.1385). (**L**) qPCR analysis of transcript levels in *STREMI* KO cells following NMD inhibitor (NMDI14) treatment for 12 h. Data are presented as mean ± SEM (*n* = 3). *P* values are from two-tailed unpaired *t* tests (*P* = 0.6475).

To investigate this microprotein further, we generated an antiserum against its N-terminus and detected a ~ 10 kDa band, which was absent following CRISPR-Cas9-mediated deletion of the uORF (Fig. 2A). Consistent with our prior report, the wild-type uORF-encoded peptide co-localized with mitochondrial markers; this localization was further supported using epitope-tagged constructs and agrees with predictions of its subcellular distribution (Figs. 2B and EV2A,B) (Zhang et al, 2020). We next asked whether the uORF-encoded microprotein is required for the overall bioenergetics of mitochondria. In mitochondrial stress test, deficiency of the uORF-encoded microprotein impaired the basal, spare, and maximum respiration rate, as well as the rate of ATP production, in HEK293T cells (Fig. 2C,D).

To investigate the exact molecular function of the microprotein in an unbiased manner, we purified it using an N-terminal Strep-tag II. Notably, all the core subunits of the mitochondrial contact site and cristae organizing system (MICOS) complex (van der Laan et al, 2016), including MIC10, MIC13, MIC19, MIC25-27, and MIC60 were identified as the strongest enriched proteins together with the bait by mass spectrometry (Fig. 2E; Dataset EV2). Because this protein-coding ORF currently lacks an approved human gene name, and in light of its additional role in regulating *SLC35A4* translation (see below), we propose the designation *STREMI* (SLC35A4 stress response regulating MICOS interactor) to reflect its molecular identity.

Beyond the core MICOS subunits, we also observed enrichment of additional components of the mitochondrial intermembrane space bridging (MIB) complex, including MTX1, MTX2, DNAJC11, and SAMM50 (Fig. 2E). Interactions between STREMI and these subunits were validated by western blotting, while no interaction between STREMI and UQCRC1, a highly abundant IMM protein, was detected (Fig. 2F). Moreover, endogenous STREMI was recovered with similar efficiency as multiple MICOS subunits when MIC10, one of the core subunits of MICOS, was purified (Fig. 2G) (Barbot et al, 2015; Bohnert et al, 2015). In this purified sample, two unique peptides contributed by endogenous STREMI were detected with high confidence by mass spectrometry (Fig. EV2C,D). The translation signal of *STREMI* is also comparable to MICOS subunits (Fig. EV2E).

Because MICOS is central to cristae formation and is preferentially enriched at cristae junctions, we next examined the submitochondrial localization of STREMI by immunogold labeling with silver enhancement in HeLa cells. In scrambled controls, gold particles were strongly enriched over mitochondria (Fig. EV2F). In contrast, mitochondrial labeling was rarely observed in *STREMI* KO cells, supporting the specificity of the antibody signal (Fig. EV2F). Gold particles were preferentially distributed along the mitochondrial envelope, with substantially fewer particles detected within the mitochondrial interior (Fig. EV2G), a pattern previously reported for multiple MICOS subunits (Harner et al, 2011). In mitochondria with clearly resolved cristae architecture, 46.5% of gold particles localized within 50 nm of cristae junctions, suggesting that STREMI is preferentially localized to cristae junctions (Fig. 2H,I) (Guarani et al, 2015; Harner et al, 2011).

We next investigated whether STREMI is required for mitochondrial cristae morphogenesis. Under transmission electron microscopy (TEM), *STREMI* knockout (KO) HeLa cells exhibited significantly fewer crista junctions and a higher frequency of mitochondria with disorganized cristae compared to controls (Fig. 2J–M). Labeling of the inner membrane with a STED-compatible dye also revealed that cristae were irregularly distributed along the long axis of mitochondria in *STREMI* KO cells (Fig. 2N) (Liu et al, 2022). This KO line carries only small frameshifting indels in the uORF while preserving the abundance of the parental *SLC35A4* transcript, indicating that the observed defects are most likely attributable to loss of STREMI (Fig. EV2H–L). These observations suggest STREMI is required for the optimal mitochondrial bioenergetics through regulating cristae morphogenesis.

### Stremi deficiency disrupts mitochondrial cristae integrity in mice

**Figure 3:**
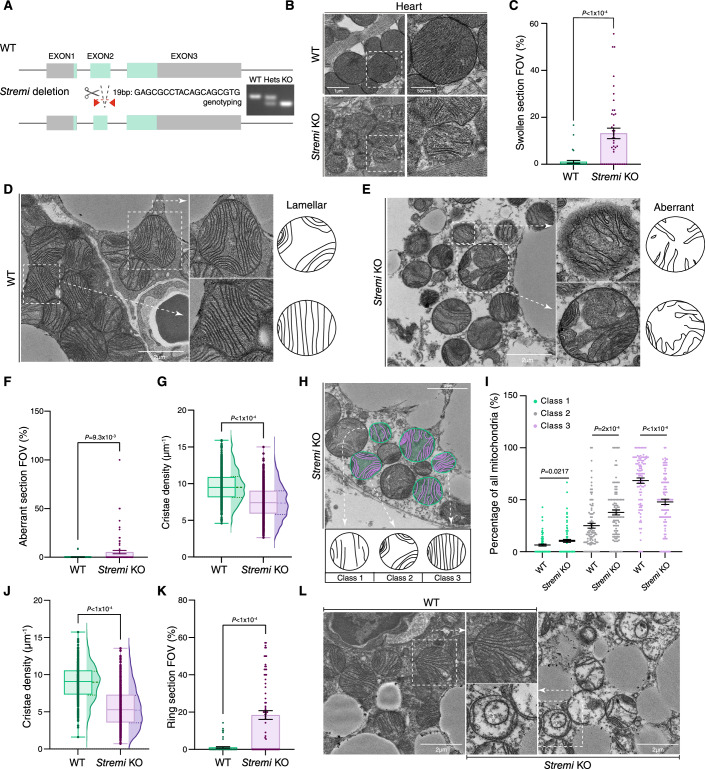
Stremi deficiency disrupts mitochondrial cristae integrity in mice. (**A**) Schematic diagram of the mutation in the *Stremi* knockout mouse line. (**B**) Representative TEM images of heart mitochondria from WT and *Stremi* KO mice. Scale bars: 1 µm (×8500), 500 nm (×22,000). (**C**) Percentage of swollen mitochondria per field of view (FOV) in cardiac tissue TEM images from WT and *Stremi* KO mice. Data are presented as mean ± SEM (*n* = 39 fields for WT; *n* = 44 fields for *Stremi* KO, 2 biological replicates). *P* values are from two-tailed unpaired t tests (*P* < 1 × 10^−^⁴). (**D**, **E**) Representative TEM images of mitochondrial morphology in the brown adipose tissue (BAT) from WT (**D**) and *Stremi* KO mice (**E**). Scale bars, 2 µm. (**F**) Percentage of aberrant mitochondria per FOV in BAT from WT and STREMI KO mice. Data are presented as mean ± SEM (*n* = 65 fields for WT; *n* = 73 fields for *Stremi* KO, 3 biological replicates). *P* values are from two-tailed unpaired *t* tests (*P* = 9.3 × 10^−^^3^). (**G**) Quantification of cristae density in BAT mitochondria from WT and *Stremi* KO mice (*n* = 653 mitochondria for WT; *n* = 671 mitochondria for *Stremi* KO, 4 biological replicates). Box-and-whisker plots show the median (central line), interquartile range (box, 25th to 75th percentile), and minimum to maximum values (whiskers), with all individual data points displayed. *P* values are from two-tailed unpaired t tests (*P* < 1 × 10^−^⁴). (**H**) Representative TEM images showing three mitochondrial subtypes classified by cristae morphology in *Stremi* mice BAT. Scale bar, 2 µm. (**I**) Prevalence of mitochondrial subtypes in BAT from WT and *Stremi* KO mice. Data are presented as mean ± SEM (*n* = 96 fields for WT; *n* = 106 fields for *Stremi* KO, 4 biological replicates). *P* values are from two-tailed unpaired t tests: Class 1 (*P* = 0.0217), Class 2 (*P* = 2 × 10^−^^4^), Class 3 (*P* < 1 × 10^−^^4^). (**J**) Quantification of cristae density in BAT mitochondria from WT and *Stremi* KO mice upon cold exposure (*n* = 311 mitochondria for WT; *n* = 374 mitochondria for *Stremi* KO, 4 biological replicates). Box-and-whisker plots show the median (central line), interquartile range (box, 25th to 75th percentile), and minimum to maximum values (whiskers), with all individual data points displayed. *P* values are from two-tailed unpaired *t* tests (*P* < 1 ×  10^−^^4^). (**K**) Percentage of ring-shaped mitochondria per FOV in BAT from WT and *Stremi* KO mice. Data are presented as mean ± SEM (*n* = 62 fields for WT; *n* = 71 fields for *Stremi* KO, 4 biological replicates). *P* values are from two-tailed unpaired *t* tests (*P* < 1 × 10^−^^4^). (**L**) Representative TEM images of BAT mitochondria from WT and *Stremi* KO mice after cold stress. Scale bars: 2 µm. Source data are available online for this figure.

**Figure EV3:**
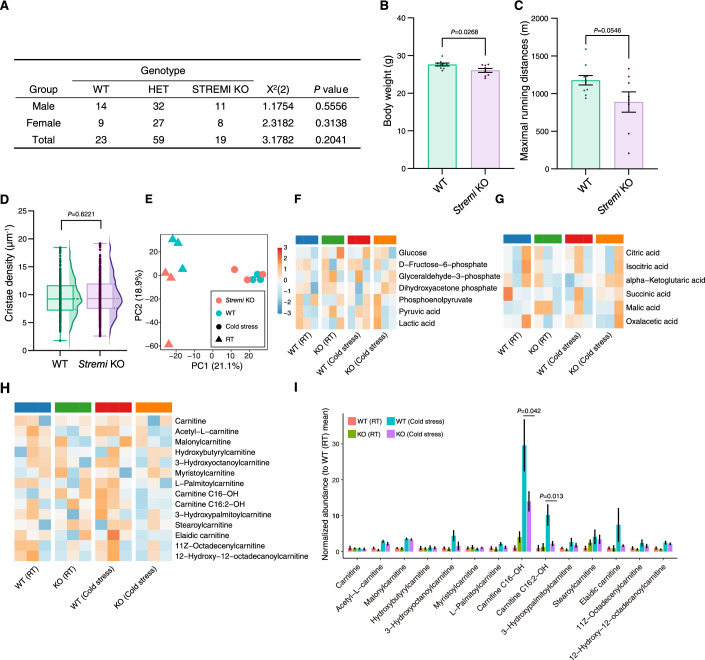
*Stremi* deletion results in mild physiological phenotypes in mice. (**A**) Genotype distribution compared with the expected Mendelian 1:2:1 ratio. Observed and expected numbers are indicated. Statistical significance was assessed by χ² goodness-of-fit test (df = 2). (**B**) Body weight of WT and *Stremi* KO mice. Data are presented as mean ± SEM (*n* = 9 mice for WT; *n* = 8 mice for *Stremi* KO). *P* values are from two-tailed unpaired *t* tests (*P* = 0.0268). (**C**) Maximum running distance of WT and *Stremi* KO mice during treadmill testing. Data are presented as mean ± SEM (*n* = 10 mice for WT; *n* = 8 mice for *Stremi* KO). *P* values are from two-tailed unpaired *t* tests (*P* = 0.0546). (**D**) Quantification of cristae density in heart mitochondria from WT and *Stremi* KO mice (*n* = 321 mitochondria for WT; *n* = 330 mitochondria for *Stremi* KO, 4 biological replicates). Box-and-whisker plots show the median (central line), interquartile range (box, 25th to 75th percentile), and minimum to maximum values (whiskers), with all individual data points displayed. *P* values are from two-tailed unpaired *t* tests (*P* = 0.6221). (**E**) Principal component analysis (PCA) of untargeted metabolomic profiles from brown adipose tissue (BAT) of wild-type (WT) and *Stremi*-knockout (KO) mice housed at room temperature (RT) or subjected to cold stress (4 °C for 5 days). (**F**–**H**) Heatmaps showing the relative abundance of metabolites from glycolysis (**F**), the tricarboxylic acid (TCA) cycle (**G**), and acylcarnitines (**H**). Values were log2-transformed and then z-scored across metabolites within each condition (RT and cold stress processed separately). (**I**) Acylcarnitine abundances in BAT normalized to WT at RT (compound-wise mean). Bars indicate mean normalized abundance and error bars represent SEM. *P* values were calculated using two-way ANOVA followed by Sidak’s multiple-comparisons correction for within-compound comparisons (*n* = 3).

Given the evolutionary conservation of STREMI across mammals, we engineered a mouse line harboring a 19-base-pair deletion in the second exon of *Stremi* using CRISPR-Cas9 (Fig. 3A). This frameshift mutation generates a divergent peptide that deviates from the wild-type (WT) sequence beyond the initial 21 N-terminal residues, ablating essential motifs critical for its subcellular localization and function (detailed below). Intercrossing heterozygous mutants produced offspring at expected Mendelian ratios (Fig. EV3A). Aside from a modest reduction in body weight at 12 weeks, we observed no overt abnormalities in growth or behavior, indicating that STREMI is not essential for normal development under standard laboratory conditions (Fig. EV3B). Consistent with a mild mitochondrial functional impairment, *Stremi* KO mice also exhibited a modest reduction in exhaustion distance during an endurance treadmill challenge (Fig. EV3C).

To probe the role of STREMI in mitochondrial architecture, we used TEM to examine cristae morphology in tissues rich in mitochondria, including cardiac muscle and brown adipose tissue (BAT). In the hearts of *Stremi* KO mice, approximately 8% of mitochondria displayed swollen cristae—an aberration absent in WT controls—though cristae density remained unaffected (Figs. 3B,C and EV3D). In brown adipose tissue, STREMI deficiency manifested as mitochondria with irregularly shaped cristae, deviating from the orderly structures seen in WT counterparts (Fig. 3D–F). Furthermore, the total cristae count was significantly reduced in *Stremi* KO animals (Fig. 3G), paralleled by a notable decline in mitochondria bearing mature cristae (Fig. 3H,I). Given the critical role of brown adipose tissue in thermogenesis, we subjected mice to prolonged cold exposure (4 °C for 5 days) to investigate STREMI’s contribution to cristae integrity under cold stress. In *Stremi* KO brown adipose tissue, cold exposure significantly reduced cristae density (Fig. 3J) and induced a marked increase in ring-shaped cristae across fields of view (Fig. 3K,L). In parallel, untargeted metabolomics of BAT revealed a significant reduction in multiple long-chain hydroxy-acylcarnitines after cold exposure, consistent with decreased fatty-acid oxidation flux (Fig. EV3E–I; Dataset EV3). Notably, impaired fatty-acid oxidation has also been reported upon disruption of proteins required for mitochondrial membrane shaping (Guo et al, 2023; Li et al, 2021; Ngo et al, 2023). These findings indicate STREMI’s critical role in maintaining cristae integrity under cold stress. Collectively, these findings establish STREMI as an important regulator of mitochondrial cristae integrity in both mice and humans.

### STREMI oligomerizes with MIC10 and is required for optimal MICOS assembly

**Figure 4:**
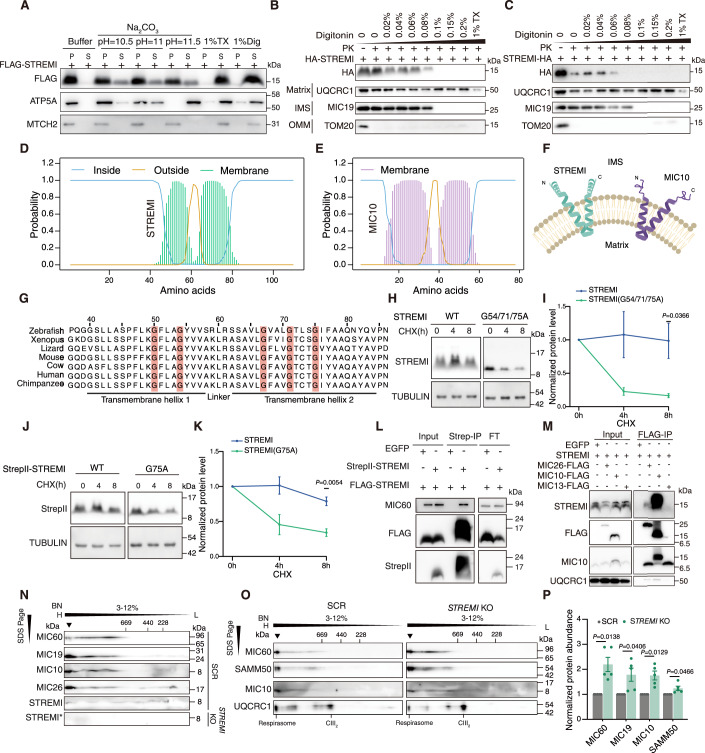
STREMI oligomerizes with MIC10 and is required for optimal MICOS assembly. (**A**) Alkaline extraction of STREMI in HEK293T mitochondrial fractions, with pellet (P) and supernatant (S) fractions analyzed by immunoblotting. (**B**, **C**) Topology of STREMI analyzed by proteinase K digestion with progressive membrane solubilization using digitonin. STREMI carried an N-terminal HA tag (**B**) or a C-terminal HA tag (**C**). TOM20, MIC19, and UQCRC1 were used as markers for the outer membrane (cytosol exposed), intermembrane space (IMS), and matrix, respectively. (**D**, **E**) Transmembrane domains of STREMI (**D**) and MIC10 (**E**) predicted by DeepTMHMM (1.0.42). (**F**) Topology of STREMI (green) and MIC10 (purple) in the mitochondrial inner membrane (IM). (**G**) Multiple sequence alignment of transmembrane segments of STREMI homologs, with evolutionarily conserved GxxxG motifs highlighted in red. (**H**) Cycloheximide-chase analysis of the STREMI triple glycine mutant (G54/71/75 A). (**I**) Quantification of degradation kinetics from (**H**) (*n* = 3 per group). Data are mean ± SEM. *P* values were calculated using an unpaired two-tailed *t* test (*P* = 0.0366). (**J**) Cycloheximide-chase analysis of the STREMI single glycine mutant (G75A). (**K**) Quantification of degradation kinetics from (**J**) (*n* = 3 per group). Data are mean ± SEM. *P* values were calculated using an unpaired two-tailed *t* test (*P* = 0.0054). (**L**) FLAG- and StrepII-STREMI co-expression in HEK293T cells with Strep-Tactin pulldown, showing FLAG-STREMI and endogenous MIC60 in different fractions (FT, flow through). (**M**) STREMI interaction screening with FLAG-MIC26/10/13 via FLAG immunoprecipitation. (**N**) Two-dimensional blue native electrophoresis of mitochondrial lysates from SCR control and *STREMI* KO 293 T cells. Asterisks (*) denote the *STREMI* KO sample. The position of the largest MICOS assembly is marked by a triangle. (**O**) Two-dimensional blue native electrophoresis of mitochondrial lysates from SCR control and *STREMI* KO 293 T cells. The position of the largest MICOS assembly is marked by a triangle. (**P**) Normalized relative abundance of smaller MICOS assemblies compared with the largest MICOS assembly, quantified from SCR control and *STREMI* KO 293T cells. The SCR control ratio was set to 1. Data are presented as mean ± SEM (*n* = 5). *P* values were calculated using two-tailed paired *t* tests: MIC60 (*P* = 0.0138), MIC19 (*P* = 0.0406), MIC10 (*P* = 0.0129), SAMM50 (*P* = 0.0466). Source data are available online for this figure.

**Figure EV4:**
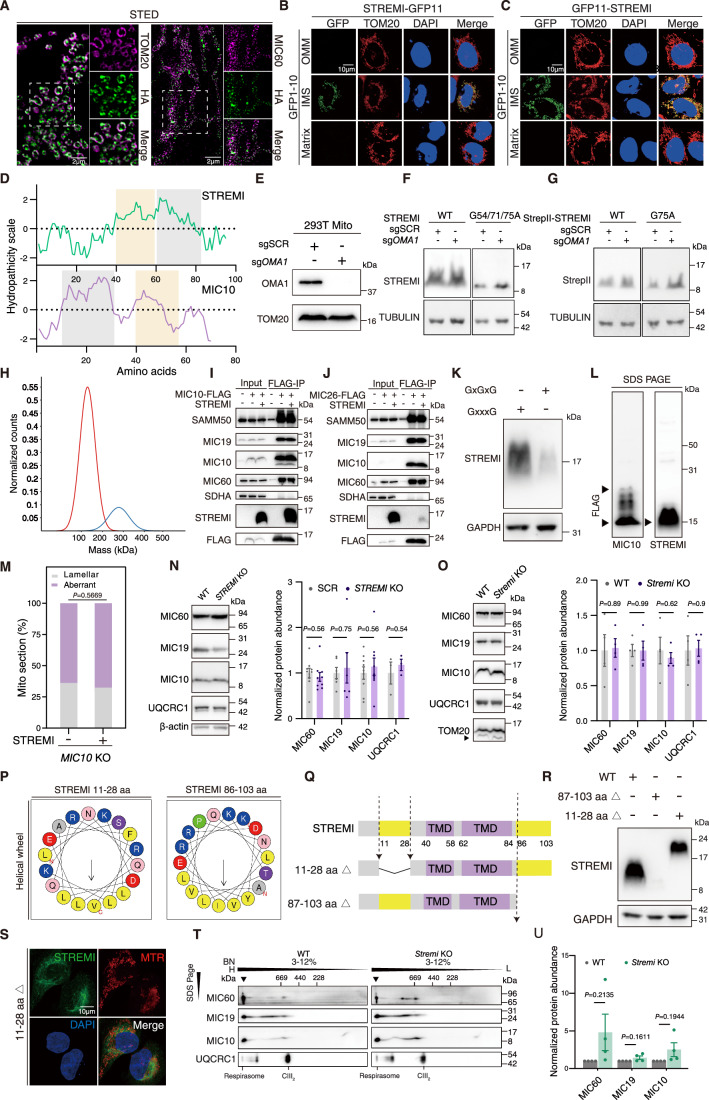
STREMI oligomerizes with MIC10 and is required for optimal MICOS assembly. (**A**) STED imaging of HA-STREMI co-stained with TOM20 (outer mitochondrial membrane marker) and MIC60 (inner mitochondrial membrane marker). Scale bars, 2 µm. (**B**, **C**) Representative confocal images of HeLa cells co-transfected with STREMI-GFP11 (**B**) or GFP11-STREMI (**C**) along with GFP1-10 targeted to the outer mitochondrial membrane (OMM), intermembrane space (IMS), or matrix. GFP fluorescence (green) and TOM20 staining (red) were used to assess the sub-mitochondrial localization of STREMI. Scale bars, 10 μm. (**D**) Hydropathy profiles of STREMI (green) and MIC10 (purple), generated using the Kyte-Doolittle scale, with hydrophobic regions highlighted. (**E**) Immunoblot analysis of OMA1 in mitochondrial lysates from SCR and *OMA1* KO 293T cells. (**F**) Immunoblotting of STREMI(G54/71/75 A) in control and *OMA1* KO HEK 293 T cells. (**G**) Immunoblotting of StrepII-STREMI(G75A) in control and *OMA1* KO HEK 293 T cells. (**H**) Mass photometry analysis of STREMI oligomerization using proteins expressed and purified from *E. coli*. (**I**, **J**) Validation of STREMI-MIC10 (**I**) and STREMI-MIC26 (**J**) associations under identical IP conditions. (**K**) Immunoblotting of STREMI mutant (L52/A69/C73G), in which the GxxxG-motif was replaced by GxGxG. (**L**) Immunoblotting of purified MIC10 and STREMI in SDS–PAGE. MIC10, but not STREMI, forms SDS-resistant oligomers (arrowed). (**M**) Percentage of mitochondria with aberrant morphology in *MIC10* KO with or without STREMI expression in HeLa cells (*n* = 88 mitochondria for MIC10 KO; *n* = 144 mitochondria for MIC10 KO with STREMI expression). *P* values were calculated using Fisher’s exact test based on raw counts (*P* = 0.5669). (**N**) SDS–PAGE of whole cell lysate (WCL) from control and *STREMI* KO HEK293T cells. Protein abundance was normalized to the level of β-actin. Data are presented as mean ± SEM (*n* = 9, except for MIC19 where *n*  =  6 and UQCRC1 where *n*  =  3). *P* values are from two-tailed unpaired *t* tests: MIC60 (*P* = 0.56), MIC19 (*P* = 0.75), MIC10 (*P* = 0.56), UQCRC1 (*P* = 0.54). (**O**) SDS–PAGE of purified WT and *Stremi* KO BAT mitochondria followed by immunoblotting for the core subunits of MICOS. Protein abundance was normalized to the level of TOM20. Data are presented as mean ± SEM (*n* = 4). *P* values are from two-tailed unpaired *t* tests: MIC60 (*P* = 0.89), MIC19 (*P* = 0.99), MIC10 (*P* = 0.62), UQCRC1 (*P* = 0.9). (**P**) Helical wheel projections of STREMI N-terminal (11–28 aa) and C-terminal (87–103 aa) segments. Hydrophobic residues are shown in yellow, negatively charged in red, positively charged in blue, and hydrophilic in purple. (**Q**) STREMI domain architecture: transmembrane domain (TMD) flanked by predicted amphipathic helices (yellow). Truncations ΔN (11–28) and ΔC (87–103) target these helices. (**R**) Immunoblotting of STREMI truncation mutants ΔN (11–28) and ΔC (87–103). (**S**) Confocal images of STREMI truncation mutant ΔN (11–28), co-stained with MitoTracker (red) and DAPI (blue). The STREMI mutant exhibits mislocalization outside mitochondria. Scale bar, 10 μm. (**T**) Two-dimensional blue native electrophoresis of BAT mitochondria from WT and *Stremi* KO mice. The position of the largest MICOS assembly is marked by a triangle. (**U**) Normalized relative abundance of smaller MICOS assemblies compared with the largest MICOS assembly, quantified from BAT mitochondria of WT and *Stremi* KO mice. The WT control ratio was set to 1. Data are presented as mean ± SEM (*n* = 4). *P* values are from two-tailed paired *t* tests: MIC60 (*P* = 0.2135), MIC19 (*P* = 0.1611), MIC10 (*P* = 0.1944).

To elucidate STREMI’s exact role in cristae regulation, we investigated its membrane association and topology. Sodium carbonate extraction revealed that STREMI primarily associates with the membrane fraction, suggesting that it is an integral membrane protein (Fig. 4A). Under STED super-resolution microscopy, STREMI staining was largely enclosed by TOM20, a marker of outer mitochondrial membrane (OMM) (Fig. EV4A). In contrast, STREMI localized in close proximity to MIC60, supporting its residence in the IMM and interaction with the MICOS complex (Fig. EV4A).

Previous studies proposed STREMI as a transmembrane protein (Rocha et al, 2024); however, the precise topology of STREMI remained unclear. To assess the membrane topology of STREMI, we tagged both the N and C termini of STREMI and performed a proteinase K sensitivity assay upon progressive solubilization of the mitochondrial membrane with a gradient of digitonin (Zhang et al, 2020). In this experimental setting, moieties in the intermembrane space (IMS) would be exposed at lower digitonin concentrations due to the higher cholesterol content of the OMM. Interestingly, both the N- and C-terminal tags showed a digestion profile similar to that of MIC19, a protein localized in the IMS (Fig. 4B,C). We recently devised a split-GFP system to report the submitochondrial localization of proteins in vivo (Zhang et al, 2023). When GFP11 tags were fused to both the N and C termini of STREMI, they exclusively complemented IMS-localized GFP1-10 molecules, and not their cytosolic or matrix counterparts, confirming the results of the protease sensitivity assays (Fig. EV4B,C). These data suggest that STREMI is an integral protein with an even number of transmembrane segments. Hydrophobicity analysis and deep-learning predictions unveiled two transmembrane domains of unequal length (Figs. 4D and EV4D), a topology mirrored by MIC10, a hairpin-structured MICOS subunit (Figs. 4E,F and EV4D) (Hallgren et al, 2022). Thus, STREMI emerges as an integral IMM protein with dual-transmembrane segments.

As a core subunit of MICOS, MIC10 oligomerizes via its uneven transmembrane domains at the cristae junction to induce membrane curvature (Barbot et al, 2015; Bohnert et al, 2015). Mechanistically, conserved glycine-rich motifs (GxxxG) within MIC10’s dual-transmembrane domains are essential for its oligomerization (Barbot et al, 2015; Teese and Langosch, 2015). Notably, STREMI also possesses GxxxG glycine motifs in both transmembrane domains, which are highly conserved across evolution (Fig. 4G). Mutating these glycine motifs significantly destabilized STREMI, underscoring their functional importance (Fig. 4H–K). In addition, the degradation of these STREMI mutants partially depends on OMA1, a membrane protease that is required for the quality control of inner membrane proteins (Fig. EV4E–G) (Kaser et al, 2003; Tang et al, 2020). To assess whether the GxxxG motif mediates STREMI oligomerization, we co-expressed STREMI proteins with distinct epitope tags in cells. Purification of Strep-tag II-STREMI efficiently co-purified FLAG-STREMI, indicating oligomer formation in vivo (Fig. 4L). Similarly, recombinant STREMI expressed in *E. coli* formed oligomers ranging from 100 to 300 kDa, as estimated by dynamic light scattering (Fig. EV4H). To probe the interaction pattern between STREMI and MICOS subunits, we co-overexpressed STREMI with several MICOS subunits, including MIC10, MIC19 and MIC26. The strongest interaction was detected between STREMI and MIC10 (Fig. 4M). Overexpression of STREMI did not impair the interaction between MIC10 and other MICOS subunits, suggesting that STREMI does not compete with MIC10 to interact with other MICOS subunits (Fig. EV4I,J). Together, these results indicate that STREMI can oligomerize with MIC10 and shares key topological and motif features with MIC10.

Despite their similarity, STREMI diverges from MIC10 in critical aspects. First, substituting STREMI’s glycine-rich GxxxG motif with MIC10’s GxGxG architecture—by mutating the central residues—led to protein destabilization (Fig. EV4K). Second, unlike MIC10’s robust oligomers, STREMI oligomers were disassociated by ionic detergent treatment (Fig. EV4L). Moreover, STREMI overexpression could not rescue cristae defects in MIC10-deficient cells (Fig. EV4M), nor was STREMI essential for the stability of MIC10 or other MICOS subunits in human HEK293T cells or mouse brown adipose tissue (Fig. EV4N,O). Beyond the hairpin-shaped dual-transmembrane domains, STREMI harbors amphipathic helices at its N- and C-termini, among the structural elements implicated in membrane remodeling (Fig. EV4P) (Hessenberger et al, 2017). Deletion of these motifs led to STREMI mislocalization and destabilization (Fig. EV4Q–S).

To assess how STREMI influences MICOS assembly state, we analyzed STREMI and MICOS complexes by BN-PAGE followed by second-dimension SDS–PAGE. STREMI displayed a broad mobility distribution, similar to MIC26, a component of the MIC10 subcomplex (Fig. 4N). Upon STREMI depletion, the overall mobility pattern of MICOS assemblies was largely preserved; however, we consistently observed an increased relative abundance of smaller MICOS assemblies at the expense of the largest species, which co-migrates with the ~1.7 MDa respirasome (Fig. 4O,P). A similar trend was observed in mouse brown adipose tissue (Fig. EV4T,U). Because prior studies indicate that the highest-molecular-weight MICOS-containing species also includes MIB components, these results suggest that STREMI contributes to maintaining higher-order MICOS/MIB assemblies (Huynen et al, 2016).

### The STREMI-encoding uORF mediates stress-responsive translation of *SLC35A4*

**Figure 5:**
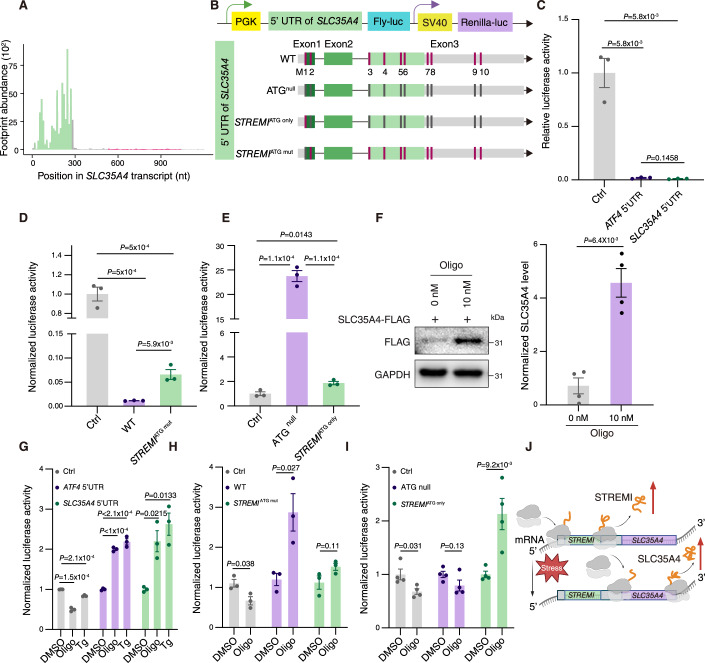
The STREMI-encoding uORF mediates stress-responsive translation of *SLC35A4.* (**A**) Ribosome footprint density across *SLC35A4* transcripts, extracted from the GWIPS-viz track of the UCSC Genome Browser. (**B**) *SLC35A4* 5’UTR variants used in dual-luciferase assays: Ctrl (empty vector), WT 5’UTR, *STREMI*^ATG mut^ (WT with *STREMI*’s start codon mutated), ATG ^null^ (all ATGs mutated to AAA), and *STREMI*^ATG only^ (ATG ^null^ with *STREMI* start codon restored). (**C**) Translation regulation of *SLC35A4* and *ATF4* 5’UTRs assessed by dual-luciferase assays. Data are presented as mean ± SEM (*n* = 3). *P* values are from two-tailed unpaired *t* tests followed by Holm–Bonferroni correction for multiple comparisons: *ATF4* 5’UTR vs. Ctrl (*P* = 5.8 × 10^−^^3^), *SLC35A4* 5’UTR vs. Ctrl (*P* = 5.8 × 10^−^^3^), *SLC35A4* 5’UTR vs. *ATF4* 5’UTR (*P* = 0.1458). (**D**) Dual-luciferase assay of Ctrl, WT and *STREMI*^ATG mut^ variants. Data are presented as mean ± SEM (*n* = 3). *P* values are from two-tailed unpaired t tests followed by Holm–Bonferroni correction for multiple comparisons: WT vs. Ctrl (*P* = 5 × 10^−^^4^), *STREMI*^ATG mut^ vs. Ctrl (*P* = 5 × 10^−^^4^), *STREMI*^ATG mut^ vs. WT (*P* = 5.9 × 10^−^^3^). (**E**) Dual-luciferase assay of Ctrl, ATG ^null^, and *STREMI*^ATG only^ variants. Data are presented as mean ± SEM (*n* = 3). *P* values are from two-tailed unpaired *t* tests followed by Holm–Bonferroni correction for multiple comparisons: ATG^null^ vs. Ctrl (*P* = 1.1 × 10^−^^4^), *STREMI*^ATG only^ vs. Ctrl (*P* = 0.0143), *STREMI*^ATG only^ vs. ATG^null^ (*P* = 1.1 × 10^−^^4^). (**F**) Immunoblotting of SLC35A4 tagged in its native transcript context after 10 nM oligomycin (oligo) treatment for 24 h. Data are presented as mean ± SEM (*n* = 4). *P* values are from two-tailed unpaired *t* tests (*P* = 6.4 × 10^−^^3^). (**G**) Translation regulation of downstream ORFs under integrated stress response (ISR) inducers, oligomycin (Oligo) and thapsigargin (Tg). Data are presented as mean ± SEM (*n* = 3). *P* values are from two-tailed unpaired t tests followed by Holm–Bonferroni correction for multiple comparisons: Ctrl, Oligo vs. DMSO (*P* = 1.5 × 10^−^^4^), Tg vs. DMSO (*P* = 2.1 × 10^−^^4^); *ATF4* 5’UTR, Oligo vs. DMSO (*P* < 1 × 10^−^^4^), Tg vs. DMSO (*P* = 2.1 × 10^−^^4^); *SLC35A4* 5’UTR, Oligo vs. DMSO (*P* = 0.0215), Tg vs. DMSO (*P* = 0.0133). (**H**) Effect of mutating the *STREMI* start codon on ISR-responsive downstream translation. Data are presented as mean ± SEM (*n* = 3). *P* values are from two-tailed unpaired *t* tests: Ctrl, Oligo vs. DMSO (*P* = 0.038); WT, Oligo vs. DMSO (*P* = 0.027); STREMI^ATG mut^, Oligo vs. DMSO (*P* = 0.11). (**I**) Dependence of ISR-responsive translation on the *STREMI* start codon. Data are presented as mean ± SEM (*n* = 4). *P* values are from two-tailed unpaired *t* tests: Ctrl, Oligo vs. DMSO (*P* = 0.031); ATG^null^, Oligo vs. DMSO (*P* = 0.13); *STREMI*^ATG only^, Oligo vs. DMSO (*P* = 9.2 × 10^−^^3^). (**J**) Schematic of ribosome occupancy on *SLC35A4* mRNA during translation. Source data are available online for this figure.

Having elucidated the molecular role of STREMI, we sought to define its interplay with SLC35A4 in translational regulation. Ribosome profiling revealed that the *SLC35A4* ORF is minimally translated under basal conditions, suggesting an inhibitory function exerted by the 5’UTR (Fig. 5A). To probe this further, we inserted the 5’UTR of *SLC35A4* to the upstream of a luciferase coding sequence (Fig. 5B). This construct revealed a pronounced suppression of downstream ORF translation, a behavior reminiscent of the well-characterized, stress-responsive 5’UTR of *ATF4* (Fig. 5C) (Vattem and Wek, 2004). Mutating the *STREMI* start codon partially alleviated this repression, hinting that additional upstream ATGs within the 5’UTR may also play an inhibitory role (Fig. 5D). To dissect the specific role of the start codon in *STREMI*, we engineered two mutants: an ATG-null mutant (lacking all potential start codons) and a mutant retaining only the STREMI-encoding start codon (Fig. 5B). Notably, elimination of all ATGs substantially relieved translational inhibition, whereas reintroduction of the *STREMI* start codon fully reinstated repression (Fig. 5E).

**Figure EV5:**
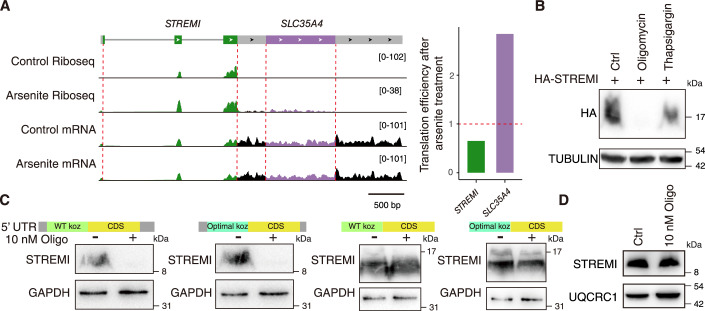
The STREMI-encoding uORF mediates stress-responsive translation of *SLC35A4.* (**A**) Ribosome footprint density on *SLC35A4* transcripts in control and arsenite-treated conditions. Data from Andreev et al (Andreev et al, 2015). (**B**) Immunoblotting of transiently expressed STREMI (within its native 5’UTR) in HEK293T cells treated with oligomycin and thapsigargin. (**C**) Dependence of ISR-responsive translation repression on the Kozak sequence and 5’UTR context. (**D**) Immunoblotting of endogenous STREMI in HEK293T cells after a 24-h treatment with oligomycin.

We next investigated how the *SLC35A4* 5’UTR modulates translation under stress. Prior ribosome profiling data indicated that chemical inducers of the integrated stress response (ISR), such as arsenite, enhance translation of the *SLC35A4* ORF (Fig. EV5A) (Andreev et al, 2015). Consistent with this, oligomycin treatment—a potent ISR trigger—elevated SLC35A4 protein levels (Fig. 5F) (Guo et al, 2020). Moreover, ISR activation by oligomycin or thapsigargin alleviated translational repression mediated by the *SLC35A4* 5’UTR, a response paralleled by the *ATF4* 5’UTR (Fig. 5G). Given that single or multiple uORFs are known to orchestrate stress-responsive translation of downstream canonical ORFs (Vattem and Wek, 2004), we directly assessed the regulatory role of *STREMI*’s uORF. Ablation of *STREMI*’s start codon abolished the stress responsiveness of the 5’UTR (Fig. 5H), whereas its restoration in an ATG-null background fully rescued this property (Fig. 5I). These findings establish that the STREMI-encoding uORF is the principal regulator of stress-induced *SLC35A4* translation (Fig. 5J).

Ribosome profiling revealed that enhanced *SLC35A4* translation under stress coincides with a modest reduction in *STREMI* translation (Fig. EV5A) (Andreev et al, 2015). Accordingly, acute ISR induction suppressed translation of transiently expressed *STREMI* within its native 5’UTR context to varying degrees (Fig. EV5B). Notably, this effect was lost upon removal of the *SLC35A4* 5’UTR sequences flanking the *STREMI* ORF, but not upon mutation of *STREMI* Kozak sequence, underscoring the critical role of the UTR context (Fig. EV5C). Intriguingly, endogenous STREMI protein levels remained stable after 24 h of 10 nM oligomycin treatment, likely reflecting its high stability (Fig. EV5D). Given the transient nature of cellular stress responses, we propose that this attenuation of *STREMI* translation facilitates stress-dependent *SLC35A4* expression without compromising steady-state STREMI abundance.

### SLC35A4 induction drives coordinated upregulation of secretory and cytosolic chaperones

**Figure 6:**
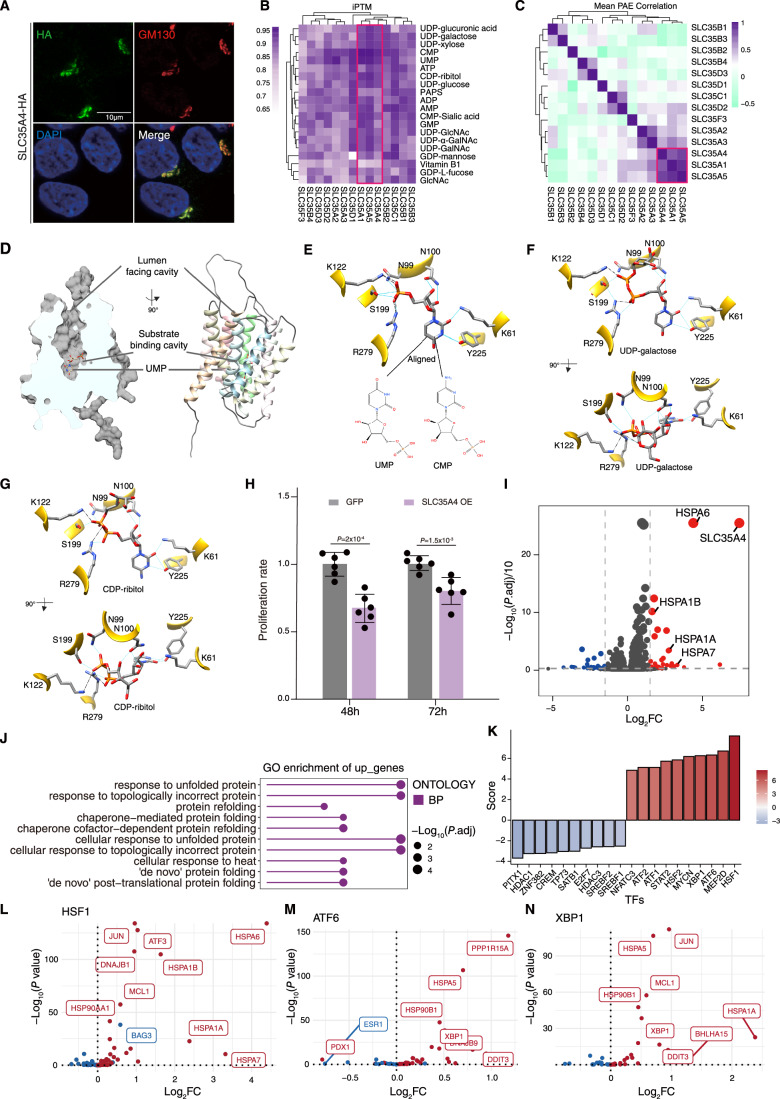
SLC35A4 induction drives coordinated upregulation of secretory and cytosolic chaperones. (**A**) Immunostaining of HA tagged SLC35A4 and the Golgi marker GM130 in HEK293T cells. Scale bar, 10 µm. (**B**) Predicted interaction confidence scores (ipTM) between SLC35A4, other deorphanized SLC35 family transporters, and known substrates, as modeled by AlphaFold3. ipTM >0.8 indicates confident prediction. (**C**) Correlation analysis of AlphaFold3-predicted transporter-substrate binding scores, based on the mean of lowest predicted aligned error (PAE) between chain pairs. (**D**) Surface and ribbon models of AlphaFold3-predicted SLC35A4 structures bound to UMP. The surface view is sliced to expose the luminal substrate-binding cavity. (**E**) Close-up view of the nucleotide-binding site, where Lys61, Asn100, and Tyr225 form hydrogen bonds to stabilize the base. Models of UMP and CMP bound structures were aligned. (**F**, **G**) Electrostatic and hydrogen bond interactions between UDP-galactose (**F**) or CDP-ribitol (**G**) and conserved residues in the binding cavity. Seven coordinating residues (Lys61, Asn99, Asn100, Lys122, Ser199, Tyr225, Arg279) are highlighted. (**H**) Cell proliferation rate of SLC35A4-overexpressing HEK293T cells. Data are presented as mean ± SEM (*n* = 6). *P* values are from two-tailed unpaired *t* tests: 48 h (*P* = 2 × 10^−^^4^), 72 h (*P* = 1.5 × 10^−^^3^). (**I**) Differential gene expression profiling in SLC35A4-overexpressing versus control in HEK293T cells. Volcano plot highlights significantly upregulated (red) and downregulated genes (blue) identified by Benjamini–Hochberg-adjusted Wilcoxon rank-sum test (*n* = 3). (**J**) Gene ontology (GO) enrichment analysis identifies the top 10 significantly upregulated biological processes in cells transfected with SLC35A4. (**K**) Transcriptional regulator activity landscape. Top 10 transcription factors (TFs) with activated (red) and repressed (blue) regulatory potentials are ranked by normalized enrichment scores. (**L**–**N**) Volcano plots of HSF1- (**L**), ATF6- (**M**), and XBP1-regulated (**N**) target genes (*n* = 3). Red and blue dots indicate genes consistent or inconsistent with the predicted regulatory effect. Target genes were defined using the CollecTRI database (Müller-Dott et al, 2023), and differential expression was analyzed with DESeq2 (Wald test). Source data are available online for this figure.

**Figure EV6:**
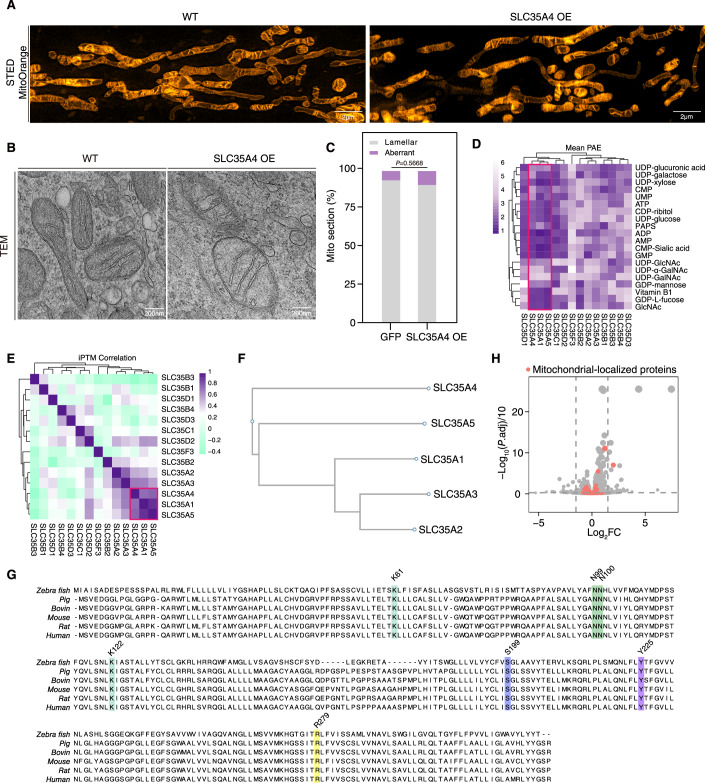
SLC35A4 induction drives coordinated upregulation of secretory and cytosolic chaperones. (**A**) STED nanoscopy analysis of mitochondrial cristae morphology in HeLa cells overexpressing SLC35A4 stained with PK Mito-Orange dye. Scale bar, 2 μm. (**B**) Representative TEM images of mitochondria from HeLa cells stably expressing GFP (control) or SLC35A4. Scale bar, 200 nm. (**C**) Quantitative analysis of mitochondrial cristae morphology based on TEM images from stable HeLa cell lines transduced with either GFP (control) or SLC35A4. Bar chart showing percentage of mitochondrial cristae categories (*n* = 53 mitochondria for control; *n* = 82 mitochondria for SLC35A4 overexpressing cells). *P* values were calculated using Fisher’s exact test based on raw counts (*P* = 0.5668). (**D**) Predicted interaction confidence scores (mean PAE) between SLC35 family transporters and various nucleotide sugar substrates, as modeled by AlphaFold3. (**E**) Correlation analysis of AlphaFold3-predicted transporter-substrate binding scores (ipTM). (**F**) Phylogenetic tree of the human SLC35A subfamily constructed from protein sequences. (**G**) Evolutionary conservation of the seven substrate-binding residues across species, mapped onto the SLC35A4 structure. (**H**) Differential gene expression profiling in SLC35A4-overexpressing versus control in HEK293T cells (*n *= 3). Volcano plot highlights mitochondrial-localized genes (red) identified by Benjamini–Hochberg-adjusted Wilcoxon rank-sum test.

SLC35A4 is a member of the nucleotide sugar transporter (NST) family, which transports nucleotide sugars into the secretory pathway in exchange for nucleotide monophosphates (Orellana et al, 2016). We confirmed its localization to the Golgi apparatus via immunostaining (Fig. 6A). Initially, we explored whether stress-induced *SLC35A4* translation influences mitochondrial cristae morphogenesis. However, neither TEM nor STED microscopy revealed alteration in cristae formation upon SLC35A4 overexpression, suggesting that SLC35A4 is acting independently of mitochondrial cristae modeling (Fig. EV6A–C).

To investigate the potential substrate preference of SLC35A4, we leveraged AlphaFold3 to predict structural interfaces between SLC35 transporters and their substrates. High-confidence predictions (interfacial predicted TM score, ipTM > 0.9, mean predicted aligned error, mean PAE < 1.6) indicate that SLC35A4 can coordinate with multiple cytidine diphosphate (CDP)/uridine diphosphate (UDP)-sugar substrates, with confidence scores comparable to those of established transporter-substrate pairs (Figs. 6B and EV6D). Correlation analysis suggested that SLC35A4’s predicted binding profile closely resembles that of SLC35A1 and SLC35A5 (Figs. 6C and EV6E), a finding corroborated by sequence alignment (Fig. EV6F) and prior reports of functional redundancy and physical association (Sosicka et al, 2017; Ury et al, 2021). In structural models of SLC35A4 with cytidine monophosphate (CMP)/ uridine monophosphate (UMP) and their corresponding diphosphate sugars, the nucleotide moiety binds deeply within the luminal pocket, and is stabilized by Lys61, Asn100, and Tyr225 via hydrogen bonds (Fig. 6D–G). Additionally, four residues (Asn99, Lys122, Ser199, and Arg279) interact with the phosphate group through electrostatic interactions and hydrogen bonding (Fig. 6F,G). These seven substrate-binding residues are evolutionarily conserved (Fig. EV6G), supporting their functional significance. Collectively, these data suggest that SLC35A4 functions as a Golgi-resident nucleotide sugar transporter, likely specialized in delivering cytosine/uridine phosphate sugars into the secretory pathway to support protein glycosylation.

Prompted by the translational activation of *SLC35A4* under stress, we next investigated its broader role in stress response regulation. Consistent with many stress-responsive proteins, SLC35A4 overexpression inhibited cell proliferation (Fig. 6H) (Kültz, 2005). To uncover the molecular pathways modulated by SLC35A4, we conducted transcriptomic profiling following its transfection. This analysis revealed significant upregulation of genes linked to the unfolded protein response (UPR) (Fig. 6I,J; Dataset EV4). Transcription factor analysis pinpointed key regulators of endoplasmic reticulum (ER) and cytosolic proteostasis—HSF1, ATF6, and XBP1—as likely orchestrators of this transcriptional shift (Fig. 6K–N) (Acosta-Alvear et al, 2025). In contrast, the transcriptional profiles of mitochondrial-localized proteins remained largely unaltered (Fig. EV6H). These data provide a foundation for understanding how SLC35A4 may contribute to glycosylation-associated proteostasis under stress, though direct experimental validation of endogenous transporter activity remains to be confirmed.

### Evolutionary analysis reveals the origin of gene symbiosis

**Figure 7:**
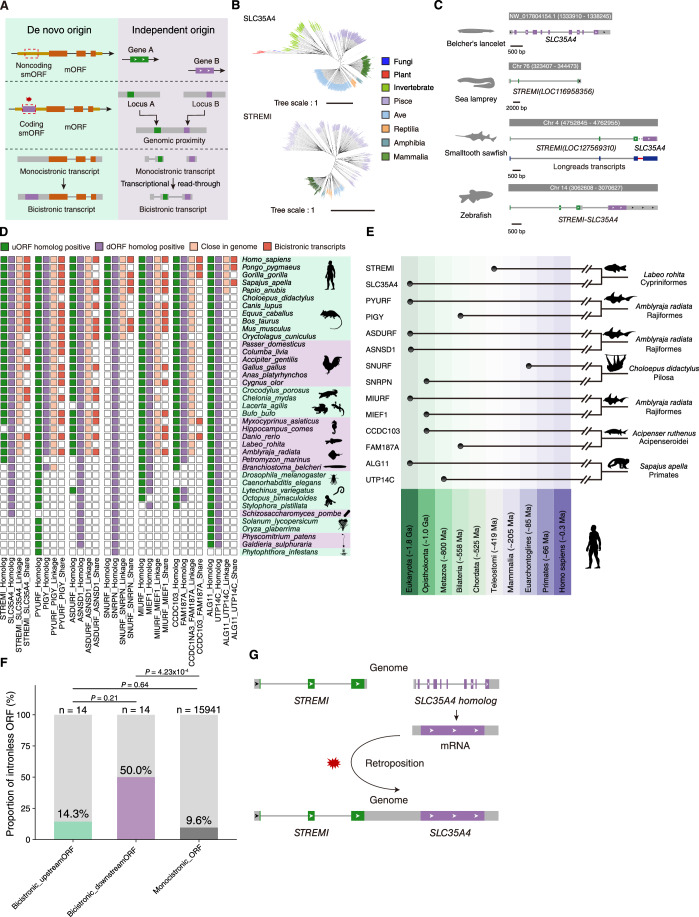
Evolutionary analysis reveals the origin of gene symbiosis. (**A**) Two hypothetical models that explain the evolutionary origin of the *STREMI*-*SLC35A4* transcript. (**B**) Phylogenetic trees of SLC35A4 and STREMI homologous colored by evolutionary clades. (**C**) Representative gene models of *STREMI* and *SLC35A4* across selected species. (**D**) Evolutionary analysis of homologs of proteins encoded by bicistronic transcripts. Presence or absence of bicistronic proteins pairs in representative eukaryotic species. Linked gene structure (candidate bicistronic transcript or adjacent separate transcripts) is indicated by a fleshcolor square. Bicistronic transcript is indicated by a red square. (**E**) Origin analysis of bicistronic proteins pairs with reference to human taxonomic group. The black dots indicate the occurrence of the corresponding homologous protein in this taxonomic group, with no later appearance than observed. (**F**) Proportion of intronless ORFs in human bicistronic and monocistronic transcripts. *P* values were calculated using pairwise Fisher’s exact tests followed by Holm-Bonferroni correction: dORF vs. monocistronic (*P* = 4.23 x 10^-4^), uORF vs. monocistronic (*P* = 0.64), uORF vs. dORF (*P* = 0.21). (**G**) Schematic of gene symbiosis through retroposition-driven co-evolution. Source data are available online for this figure.

Our findings indicate that *STREMI* and *SLC35A4*, despite sharing a bicistronic transcript that enables *STREMI*-mediated *SLC35A4* translation activation under stress, lack direct functional interdependence. To reconcile this observation, we explored the evolutionary origins of their bicistronic arrangement. The absence of sequence homology between *STREMI* and *SLC35A4* prompted two hypotheses: (1) *STREMI* emerged de novo from the 5’UTR of *SLC35A4*, or (2) transcriptional read-through fused two independently evolved genes originally situated in genomic proximity (Fig. 7A). To test these possibilities, we traced the evolutionary history of both proteins.

**Figure EV7:**
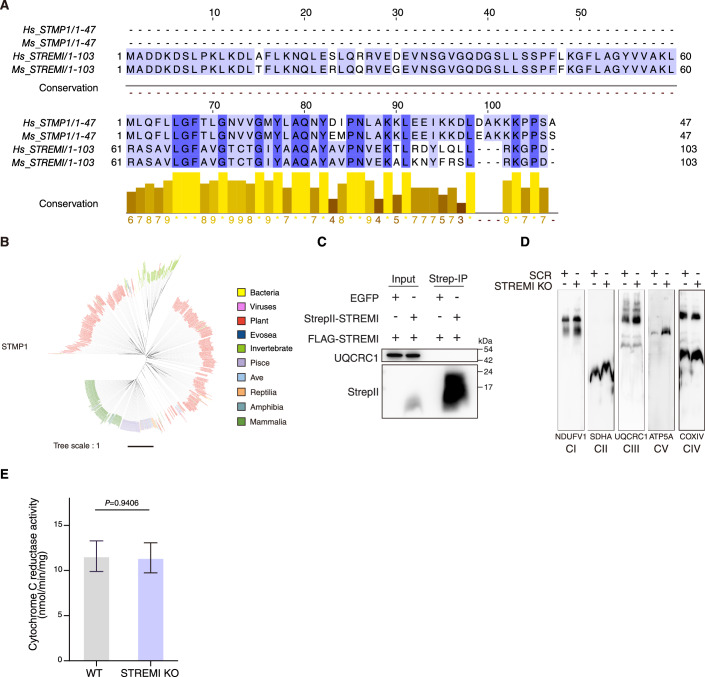
Evolutionary analysis reveals the origin of gene symbiosis. (**A**) Protein sequence alignment of human and murine STREMI and STMP1. (**B**) Phylogenetic tree of STMP1 homologs constructed using the maximum likelihood method. (**C**) Immunoprecipitation of Strep tagged STREMI, followed by immunoblotting for the CIII component UQCRC1. (**D**) Assembly of the electron transport chain (ETC) complexes analyzed by blue native-PAGE in STREMI knockout (KO) HEK293T cells. (**E**) CIII enzymatic activity in mitochondria from WT and STREMI KO HEK293T cells, normalized to total protein. Data are presented as mean ± SEM (*n* = 4). *P* values are from two-tailed unpaired *t* tests (*P* = 0.9406).

Searching for STREMI homologs, we identified significant sequence similarity between its C-terminus and STMP1, another mitochondrial microprotein (Fig. EV7A) (Bhatta et al, 2020). Phylogenetic analysis revealed that STMP1 is conserved across land plants and metazoans, whereas STREMI homologs are exclusive to vertebrates (Figs. 7B and EV7B). Despite their shared ancestry, STREMI does not appear to overlap functionally with STMP1, a known assembly factor for respiratory chain complex III (CIII) (Makarewich et al, 2022). We detected no interactions between STREMI and core CIII subunits (Fig. EV7C), nor did STREMI deficiency impair CIII assembly or activity (Fig. EV7D,E). These data suggest that STREMI diverged from an STMP1-like ancestor to assume a distinct role in vertebrate cells.

**Figure EV8:**
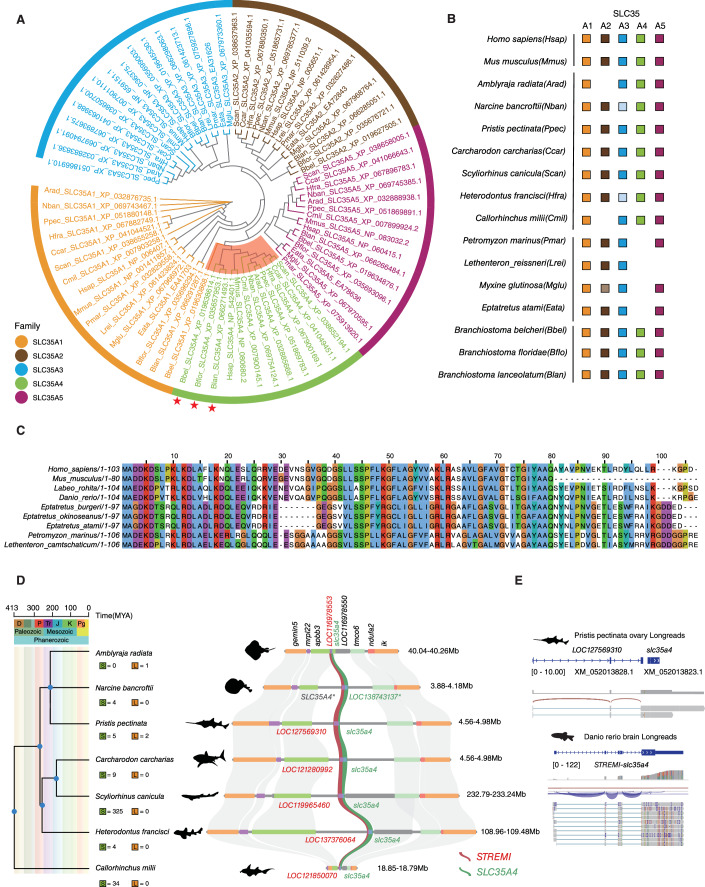
Evolutionary analysis of STREMI and SLC35A4 homologs in chordates. (**A**) Protein phylogenetic tree of SLC35 family members across lancelet species and vertebrates. Branches are color-coded by gene family, with the SLC35A4 clade highlighted; red stars indicate SLC35A4 in lancelet species. (**B**) Presence–absence matrix showing the distribution of SLC35 homologs across major chordate and vertebrate lineages. (**C**) Multiple sequence alignment of representative STREMI homologs highlighting conserved residues across species. (**D**) Conserved synteny of the *STREMI–SLC35A4* locus in representative cartilaginous fishes, integrated with species phylogeny and available RNA-seq datasets (S short-read, L long-read). Asterisks indicate likely annotation errors in NCBI genome records, in which the names of *slc35a4* and the uORF-encoded *STREMI* homolog (*LOC138743137*) appear to be swapped. (**E**) Long-read Iso-Seq evidence supporting bicistronic transcription of the *STREMI-SLC35A4* locus in *Pristis pectinata* and *Danio rerio*. Gene models and mapped reads illustrate the shared transcript structure.

In contrast, the SLC35 family of nucleotide sugar transporters is deeply conserved across fungi, plants, and metazoans (Fig. 7B). Within this family, phylogenetic analysis of protein sequences indicates that SLC35A4 orthologs were already specified in lancelets, a basally divergent extant chordate lineage (Fig. EV8A,B; Dataset EV5).

We next asked when the bicistronic transcript emerged. In lancelets, we identified *SLC35A4* orthologs but found no evidence for an associated *STREMI* ORF (Fig. 7C; Dataset EV5). In multiple species of sea lamprey and hagfish—basally divergent jawless vertebrates—we identified highly conserved STREMI orthologs (Fig. EV8C). However, in these species the annotated SLC35-family genes cluster with SLC35A1/2/3/5, and none are positioned near the STREMI locus (Fig. EV8A,B; Dataset EV5). By contrast, in jawed vertebrates, *STREMI* and *SLC35A4* reside in close genomic proximity, and bicistronic transcripts have emerged—at least in some species—as supported by long-read RNA sequencing and synteny analyses (Figs. 7C and EV8D,E). Collectively, these observations indicate that STREMI and SLC35A4 originated before the emergence of the bicistronic transcript, which was later established in jawed vertebrates.

To trace the evolutionary origin of other protein pairs encoded by human bicistronic transcripts, we conducted sequence and structural homology searches (Fig. 7D,E; Dataset EV6). In most cases, homologs of both proteins appeared earlier in evolution than the emergence of their bicistronic co-expression. We refer to this evolutionary pattern—where independently originating genes become co-transcribed in a single transcript—as “bipartite origin, bicistronic transcription.”

What genomic event might underlie this convergence? We observed that the CDS of *SLC35A4* is consistently intronless when located near *STREMI*, a trait not shared by its homologs *SLC35A1/2/3/5* (Figs. 1E and 7C). This pattern is common among bicistronic transcripts, with 14.3% of uORFs and 50% of dORFs lacking introns, compared to fewer than 10% of intronless CDSs in human monocistronic transcripts (Fig. 7F). Intronless genes frequently originate through retroposition, a process in which spliced mRNA is reverse-transcribed into cDNA and subsequently integrated into the genome (Casola and Betrán, 2017). Lacking native regulatory elements, many retroposed CDSs are transcriptionally silent and degenerate into pseudogenes (Esnault et al, 2000). However, when retroposition occurs adjacent to transcriptionally active loci, the inserted CDS may be co-transcribed with the host gene, potentially evolving into a stable, functional bicistronic transcript. Together, these findings support an evolutionary model in which *SLC35A4* and *STREMI*, originally independent genes, established a stable transcriptional “symbiosis” following a retroposition event that inserted the *SLC35A4* CDS into the *STREMI* locus (Fig. 7G).

## Discussion

In this study, we systematically investigated the prevalence, function, and evolutionary origins of human polycistronic transcripts, specifically characterizing the dual-protein-encoding transcript of *SLC35A4*. Our findings demonstrate that the uORF of *SLC35A4* encodes STREMI, a mitochondrial inner membrane-localized microprotein critically involved in mitochondrial cristae morphogenesis via direct interaction with the MIC10 subunit of the MICOS complex. Notably, this uORF not only encodes a functional microprotein but also operates as a cis-regulatory element that modulates the stress-responsive translation of the downstream *SLC35A4* coding sequence. Under conditions of cellular stress, SLC35A4 expression is upregulated, thereby enhancing proteostasis pathways, particularly those involving molecular chaperones essential for maintaining protein homeostasis.

This work expands our understanding of the functional protein-coding potential inherent to 5’UTRs. Prior independent reports, including ours, have reported protein-coding capacity of the *SLC35A4* 5’UTR (Ajala et al, 2025; Andreev et al, 2015; Rocha et al, 2024; Vanderperre et al, 2013; Yang et al, 2024; Zhang et al, 2020). Through combined biochemical and genetic analyses in cultured human cells and a mouse knockout model, we demonstrate that STREMI orchestrates cristae morphogenesis via its physical association with the MICOS complex. Using protease protection assays and split-GFP–based topology reporters, we provide direct evidence that STREMI adopts a dual-transmembrane topology within the inner mitochondrial membrane, with both termini facing the intermembrane space. These findings help resolve ongoing debates regarding STREMI’s submitochondrial localization and membrane topology (Rocha et al, 2024; Yang et al, 2024).

This topology, combined with the presence of conserved glycine-rich GxxxG motifs, promotes STREMI oligomerization with MIC10. In this configuration, STREMI emerges as a MICOS-associated factor required for optimal assembly of MICOS-related higher-order complexes. Our data further suggest that STREMI may function either as an accessory subunit or as an assembly factor for MICOS/MIB assemblies. Future high-resolution structural analyses will be important to define how STREMI engages MICOS and how it modulates MICOS assembly.

Notably, while we were preparing this manuscript, Rocha et al independently reported that loss of SLC35A4-MP/STREMI disrupts mitochondrial morphology, reduces cristae density under a high-fat diet, and impairs brown adipose thermogenesis (Rocha et al, 2025). However, a coherent molecular model explaining these phenotypes remains elusive. Our study provides a timely mechanistic complement to their findings and highlights a direct link between cristae morphogenesis and mitochondrial fatty acid oxidation—an emerging axis of physiological regulation that warrants further investigation.

Our data further highlighted the potential regulatory role of the STREMI-encoding uORF in modulating stress-responsive translation of *SLC35A4*, a member of the Golgi nucleotide sugar transporters. While direct functional assays of substrate transport remain to be performed, protein-substrate interaction modeling based on AlphaFold3 predicts that SLC35A4 preferentially transports uridine and cytidine diphosphate-linked sugar substrates, consistent with previous studies suggesting functional overlap with SLC35A1 in CDP-ribitol transport and heterodimer formation with SLC35A5 in the transport of multiple UDP-sugars (Sosicka et al, 2017; Ury et al, 2021). These substrates are essential for the glycosylation of secretory proteins—a critical process for proper folding and quality control within the secretory pathway. Given the dynamic cycling of Golgi-resident proteins with the endoplasmic reticulum (Vashist and Ng, 2004; Zhang et al, 2017), it is plausible that stress-induced SLC35A4 expression exerts broad effects on secretory proteostasis, as supported by our experimental observations. Future studies will be essential to experimentally validate these predicted transport activities and to delineate the precise mechanisms by which SLC35A4 modulates secretory protein homeostasis under stress conditions.

Beyond the molecular characterization on STREMI and SLC35A4, our evolutionary analyses shed new light on the origins and functional integration of bicistronic transcripts. By tracing the evolutionary history of STREMI and SLC35A4, we discovered that these two proteins originated independently first and converged into a single transcriptional unit through a genomic rearrangement likely driven by retroposition (Tan et al, 2016). Such rearrangements, characterized by the integration of intronless retroposed CDSs into existing transcriptional units, appear to underlie a broader evolutionary principle facilitating a process we term “gene symbiosis”. The “bipartite origin, bicistronic transcription” model exemplified by the STREMI-SLC35A4 transcript offers a conceptual framework for understanding how disparate proteins may become functionally integrated within polycistronic arrangements in vertebrate genomes.

Taken together, our study offers significant insights into the previously underappreciated complexity of eukaryotic gene expression. By demonstrating the dual functionality of a uORF in regulating mitochondrial structure and orchestrating stress-responsive translation, we underscore the adaptive advantages conferred by polycistronic transcripts in vertebrates. Understanding these intricate layers of translational and regulatory control holds potential implications for therapeutic strategies targeting mitochondrial dysfunction and stress-related disorders.

## Methods

**Table:** Reagents and tools table

Reagent/resource	Reference or source	Identifier or catalog number
**Experimental models**		
Human HEK293T (STR profiling)	Procell	Cat#CL-0005
Human HeLa (STR profiling)	Procell	Cat#CL-0101
Human U2OS (STR profiling)	Procell	Cat#CL-0236
Human HEK293T-STREMI KO	This study	N/A
Human HEK293T-OMA1 KO	This study	N/A
Human HeLa-STREMI KO	This study	N/A
Human HeLa-MIC10 KO	This study	N/A
Human HeLa-SLC35A4 OE	This study	N/A
Mouse:C57BL/6J	Shanghai Model Organisms Center	Cat#SM-001
Mouse:C57BL/6J-STREMI ^+/−^	Shanghai Model Organisms Center	Cat#NM-KO-240882
Mouse:C57BL/6J-STREMI ^−/−^	This study	N/A
TStbl3 Chemically Competent Cell	TSINGKE	Cat#TSC-C06
Rosetta (DE3) Competent Cell	TSINGKE	Cat#DLC204
DH5α competent cells	Solarbio	Cat#C1100
**Recombinant DNA**		
pLV3-pEF1a-STREMI-pCMV-miRFP670-T2A-Puro	This study	N/A
pLV-pEF1a-HA-STREMI-IRES-BSD	This study	N/A
pLV3-pCMV-FLAG-STREMI-pEF1a-Puro	This study	N/A
pLV3-pCMV-SLC35A4-pEF1a-Puro	This study	N/A
pLV3-pCMV-SLC35A4-HA-pEF1a-Puro	This study	N/A
pCMV-HA-STREMI-SLC35A4-3 × FLAG-pEF1a-Puro	This study	N/A
pCDH-pCMV-HA-STREMI (87-103AA) truncated-pEF1a-BSD	This study	N/A
pLV3-pEF1a-STREMI-mutant-L52G-A69G-C73G-pCMV-miRFP670-T2A-Puro	This study	N/A
pLV-pEF1a-STREMI-1st α- helix (11-28AA) truncated-IRES-BSD	This study	N/A
pLV-pEF1a-HA-STREMI-1st α- helix (11-28AA) truncated-IRES-BSD	This study	N/A
pLV3-pCMV-MIC10-StrepII-pEF1a-Puro	This study	N/A
pLV3-pEF1a-STREMI-GFP11-pCMV-miRFP670-T2A-Puro	This study	N/A
pLV3-pEF1a-GFP11-STREMI-pCMV-miRFP670-T2A-Puro	This study	N/A
pcDNA3.1/Hygro(+)-Cox8a-GFP1-10	This study	N/A
pcDNA3.1/Hygro(+)-LACTB-GFP1-10	This study	N/A
pcDNA3.1/Hygro(+)-GFP1-10-OMP25	This study	N/A
pLV3-pCMV-StrepII-STREMI-pEF1a-Puro	This study	N/A
pLV3-pCMV-STREMI-HA-pEF1a-Puro	This study	N/A
pLV3-pCMV-MIC10-3 × FLAG-pEF1a-Puro	This study	N/A
pLV3-pEF1a-MIC10-pCMV-miRFP670-T2A-Puro	This study	N/A
pLV3-CMV-MIC26-FLAG-pEF1a-Puro	This study	N/A
pLV3-CMV-MIC13-FLAG-pEF1a-Puro	This study	N/A
pmirGLO-PGK-Fly luc-SV40-Renilla luc	This study	N/A
pmirGLO-PGK-SLC35A4-5’UTR-Fly luc-SV40-Renilla luc	This study	N/A
pmirGLO-PGK-SLC35A4-5’UTR-ATG null-Fly luc-SV40-Renilla luc	This study	N/A
pmirGLO-PGK-SLC35A4-5’UTR-ATG null-STREMI^ATG only^-Fly luc-SV40-Renilla luc	This study	N/A
pmirGLO-PGK-SLC35A4-5’UTR-WT-STREMI^ATG mut^-Fly luc-SV40-Renilla luc	This study	N/A
pmirGLO-PGK-ATF4 uORF1 uORF2-Fly luc-SV40-Renilla luc	This study	N/A
pmirGLO-PGK-SLC35A4-5’UTR-optimal kozak-Fly luc-SV40-Renilla luc	This study	N/A
pLV3-pEF1a-SLC35A4-5’UTR-optimal kozak-pCMV-miRFP670-T2A-Puro	This study	N/A
lentiCRISPR v2-Puro	Addgene	Plasmid #52961
lentiCRISPR v2-Blast	Addgene	Plasmid #98293
lentiCRISPRv2-scr	Addgene	Plasmid #169795
pRSV-Rev	Addgene	Plasmid #12253
pMD2.G	Addgene	Plasmid #12259
pMDLg/pRRE	Addgene	Plasmid #12251
pET-21a(+)-6HIS-HsSTREMI Ecoli Opti	This study	N/A
**Antibodies**		
Rabbit polyclonal anti-STREMI(WB 1:1000, IF 1:500)	This study	N/A
Rabbit polyclonal anti-DYKDDDDK(WB 1:5000)	ProteinTech	Cat#20543-1-AP RRID:AB_11232216
Mouse monoclonal anti-FLAG(WB 1:5000)	Sigma	Cat#F1804-50UG RRID:AB_262044
Rabbit monoclonal anti-GAPDH(WB 1:5000)	Cell Signaling Technology	Cat#2118 RRID:AB_561053
Mouse monoclonal anti-Tubulin(WB 1:5000)	ProteinTech	Cat#66031-1-Ig RRID:AB_11042766
Rabbit polyclonal anti-SDHA(WB 1:5000)	AB Clonal	Cat#A2594 RRID:AB_2764479
Rabbit polyclonal anti-TOM20(WB 1:5000, IF 1:500)	ProteinTech	Cat#11802-1-AP RRID:AB_2207530
Rabbit polyclonal anti-UQCRC1(WB 1:5000)	AB Clonal	Cat#A3339 RRID:AB_2765058
Mouse monoclonal anti-HA(WB 1:5000, IF 1:500)	Santa Cruz	Cat#SC-7392 RRID:AB_627809
Mouse monoclonal anti-StrepII(WB 1:5000)	Biodragon	Cat#B1195
Rabbit polyclonal anti-Mitofilin(WB 1:5000, IF 1:500)	ProteinTech	Cat#10179-1-AP RRID:AB_2127193
Rabbit polyclonal anti-MIC19(WB 1:5000)	AB Clonal	Cat#A19959 RRID:AB_2862868
Mouse monoclonal anti-MIC26(WB 1:5000)	Invitrogen	Cat#MA5-15493, RRID:AB_10982238
Sam50 Polyclonal antibody(WB 1:2000)	ProteinTech	Cat#20824-1-AP RRID: AB_10695190
OMA1 Polyclonal antibody(WB 1:2000)	ProteinTech	Cat#17116-1-AP RRID: AB_2299053
STAR ORANGE anti-Mouse(STED 1:250)	Abberior	Cat#STRED-1001
STAR RED anti-Rabbit(STED 1:250)	Abberior	Cat#STORANGE-1002
Rabbit polyclonal anti-MTCH2(WB 1:2500)	ProteinTech	Cat#16888-1-AP RRID:AB_2266733
Rabbit polyclonal anti-NDUFV1(WB 1:5000)	ProteinTech	Cat#11238-1-AP RRID:AB_2149040
Rabbit polyclonal anti-COX IV(WB 1:5000)	ProteinTech	Cat#11242-1-AP RRID:AB_2085278
Mouse monoclonal anti-ATP5A	Santa Cruz	Cat#SC-136178 RRID:AB_627809
Rabbit polyclonal anti- GOLGA2/GM130	ProteinTech	Cat#11308-1-AP RRID:AB_2115327
Rabbit polyclonal anti-MIC10(WB 1:5000)	Affinity	Cat#DF14761
HRP-conj Donkey Anti-Mouse IgG (H + L) (WB 1:10000)	Jackson ImmunoResearch	Cat#715-035-150 RRID:AB_2340770
HRP-conj Goat Anti-Rabbit IgG (H + L) (WB 1:10000)	Jackson ImmunoResearch	Cat#111-035-003 RRID:AB_2313567
Donkey anti-Mouse IgG (H + L) Highly Cross-Adsorbed Secondary Antibody, Alexa Fluor 488(IF 1:1000)	Invitrogen	Cat#A-21202 RRID:AB_141607
Donkey anti-Rabbit IgG (H + L) Highly Cross-Adsorbed Secondary Antibody, Alexa Fluor 568(IF 1:1000)	Invitrogen	Cat#A-10042 RRID:AB_2535792
**Oligonucleotides and other sequence-based reagents**		
STREMI gRNA #1-(5’GTAGAAGACGAAGTCAACAG3’)	This study	N/A
STREMI gRNA #2-(5’GAATCCCTTGAGGAACGGGG3’)	This study	N/A
STREMI mouse gDNA-(5’GGAGCGCCTACAGCAGCGTG3′)	This study	N/A
MIC10 gRNA-(5’CGACCGCATCCGCCAGACAC3’)	This study	N/A
scramble gDNA-(5’GCTTAGTTACGCGTGGACGA3’)	Suzuki et al, 2016	N/A
OMA1 gRNA-(5’ACCGGAGCAGCTTGAAACCG3’)	This study	N/A
STREMI-A4 qPCR primer1-F(5’GGCTTTGCTGTGGGCACC3’)	This study	N/A
STREMI-A4 qPCR primer1-R(5’AGGGATTGCAACACGTTTGGG3’)	This study	N/A
STREMI-A4 qPCR primer2-F(5’TGCTGACTGAGCTGACCAAG3’)	This study	N/A
STREMI-A4 qPCR primer2-R(5’ACACTGACGACAAGCCTGAG3’)	This study	N/A
**Chemicals, enzymes and other reagents**		
Digitonin	Biosynth	Cat#D-3203
Lipo8000	Beyotime	Cat#C0533
Anti-FLAG magnetic beads	Selleck	Cat#B26101
Strep-Tactin® Sepharose® resin	IBA	Cat#2-1201-002
Anti-FLAG® M2 Affinity Gel, purified immunoglobulin, buffered aqueous glycerol solution	Sigma-Aldrich	Cat#A2220
3X FLAG® Peptide	Beyotime	Cat#P9801
Protease inhibitor cocktail for general use, 100X	Beyotime	Cat#P1005
PMSF	Roche	Cat#10837091001
Sucrose	Aladdin	Cat# S112234
Tris Base	Aladdin	Cat# T591027
Bis-Tris	Aladdin	Cat# B105639
MOPS	Aladdin	Cat# M105133
SDS	Sangon	Cat# A600485
RIPA Lysis Buffer	Beyotime	Cat# P0013C
Glycerol	Sangon	Cat# A600232
Triton™ X-100	Sigma-Aldrich	Cat#T8787
FCCP	MCE	Cat#HY-100410
Oligomycin	MCE	Cat#HY-N6782
Rotenone	Aladdin	Cat#R105076
Antimycin A	Maokangbio	Cat#MS0070
Sodium carbonate, anhydrous	Sangon	Cat#A610615
Pierce™ Trypsin Protease, MS Grade	Thermo Fisher Scientific	Cat#90057
Proteinase K	Beyotime	Cat#ST533
PK Mito orange	Genvivo	Cat#PKMO-2
MitoTracker™ Red CMXRos	Thermo Fisher Scientific	Cat#M7512
4% Paraformaldehyde	Solarbio	Cat#P1110
Seahorse XF DMEM medium, pH 7.4	Agilent	Cat#103575-100
Seahorse XF Calibrant	Agilent	Cat#100840-000
Glutamine	Gibco	Cat#A2916801
D-desthiobiotin	Merck	Cat#71610-M
Verapamil	Macklin	Cat#V820460
Glutaraldehyde	Sinopharm	Cat#30092436
GoldBand Plus 3-color Regular Range Protein Marker(8-180 kDa)	Yeasen	Cat#20350ES72
BeyoColor™ Color Prestained Protein Ladder (6.5-270kD)	Beyotime	Cat#P0071
NotI-HF restriction enzyme	NEB	Cat#R3189L
XhoI restriction enzyme	NEB	Cat#R0146S
Quick CIP phosphatase	NEB	Cat#M0525S
BamHI-HF restriction enzyme	NEB	Cat#R3136S
EcoRI-HF restriction enzyme	NEB	Cat#R3101S
SalI-HF restriction enzyme	NEB	Cat#R3138S
NheI-HF restriction enzyme	NEB	Cat#R3131S
NdeI restriction enzyme	NEB	Cat#R0111V
DpnI restriction enzyme	NEB	Cat#R0176V
T4 DNA ligase	NEB	Cat#M0202S
BsaI-HF®v2 restriction enzyme	NEB	Cat#R3733V
BbsI-HF restriction enzyme	NEB	Cat#R3539S
XbaI restriction enzyme	NEB	Cat#R0145S
ClaI restriction enzyme	NEB	Cat#R0197S
AvrII restriction enzyme	NEB	Cat#R0174S
MluI-HF restriction enzyme	NEB	Cat#R3198S
SbfI-HF restriction enzyme	NEB	Cat#R3642S
AsiSI restriction enzyme	NEB	Cat#R0630S
2 × Phanta Flash Master Mix	Vazyme	Cat# P510
Taq pro Universal SYBR qPCR Master Mix	Vazyme	Cat#Q-712
**Software**		
Image Lab	Bio-rad	https://www.bio-rad.com/zh-cn/product/image-lab-software
GraphPad Prism 10.4.0	GraphPad	https://www.graphpad.com
Fiji 2.9.0	Schindelin et al, 2012	https://fiji.sc/
DeepLoc 2.0	DTU Health Tech	https://services.healthtech.dtu.dk/services/DeepLoc-2.0/
DeepTMHMM (1.0.42)	DTU Health Tech	https://dtu.biolib.com/DeepTMHMM/
ORFfinder (v0.4.3)	Github	https://github.com/Augustpan/NCBI_ORFfinder
InterProScan (v5.69-101.0)	Jones et al, 2014	https://github.com/ebi-pf-team/interproscan
PhyloCSF + +	Pockrandt et al, 2022	https://github.com/cpockrandt/PhyloCSFpp
**Other**		
Enhanced BCA Protein Assay Kit	Beyotime	Cat#P0010
ClonExpress Ultra One Step Cloning Kit	Vazyme	Cat#C115-02
FastPure® Cell/Tissue DNA Isolation Mini Kit	Vazyme	Cat#DC102-01
Seahorse XFe96/XF Pro FluxPak Mini	Agilent	Cat#103793-100
FastPure Cell/Tissue Total RNA Isolation Kit	Vazyme	Cat#RC101-01
TIANprep Mini Plasmid Kit	TIANGEN	Cat#DP103
FastPure EndoFree Plasmid Maxi Kit	Vazyme	Cat#DC202-01
HiScript III All-in-one RT SuperMix Perfect for qPCR	Vazyme	Cat#R333-01
HiScript III 1st Strand cDNA Synthesis Kit ( + gDNA wiper)	Vazyme	Cat#R312-01
Dual Luciferase Reporter Gene Assay Kit	Yeasen	Cat#11402ES60
Cell Counting Kit (CCK-8)	Yeasen	Cat#40203ES60

### Identification of candidate bicistronic transcripts

The human reference genome (GRCh38) and corresponding Ensembl canonical transcripts were downloaded from the Ensembl database (Dyer et al, 2025). Only canonical transcripts with both transcript_type and gene_type annotated as protein_coding were retained for analysis. Open reading frames (ORFs) were predicted for each transcript sequence using NCBI ORFfinder (v0.4.3) (Rombel et al, 2002), using the standard genetic code for nuclear genes (-g 1) and allowing both canonical (“ATG”) and alternative initiation codons as start sites (-s 1), with a minimum ORF length of 90 nucleotides (-ml 90).

Translated amino acid sequences were searched against Pfam, Panther, and Gene3D databases using InterProScan (v5.69-101.0) (Quevillon et al, 2005). Sequences positive in protein family search were searched against the UniRef90 database using MMseqs2 (v15.6f452) (Steinegger and Söding, 2017). Only ORFs with an alignment coverage ≥ 0.8 were retained as valid candidates. Transcripts containing two or more valid ORFs were selected as candidate bicistronic transcripts.

### Identification of high-confidence bicistronic transcripts

Candidate bicistronic transcripts were validated using long-read RNA sequencing (LrRNA-seq) data from the ENCODE project (accession: ENCFF373TKM) (Kazachenka et al, 2018). Based on raw sequencing reads, we determined whether both ORFs were co-located on the same transcript and calculated per-ORF coverage accordingly. Only transcripts with ORF-normalized read coverage score showing high coverage (≥ 0.7) were retained. PhyloCSF scores were calculated using PhyloCSF ++ (v1.2.0) based on the UCSC hg38 Human: Multiz Alignments of 100 Vertebrates (Pockrandt et al, 2022). For each ORF within a transcript, scores were extracted using custom scripts in combination with the bigWigSummary tool. ORFs with positive scores were labeled as PhyloCSF Positive, and the number of such ORFs was counted per transcript.

Ribosome profiling (Ribo-seq) signal tracks from GWIPS-viz (hg38) were first used to assess translational potential for all candidate ORFs. For each ORF, the mean ribosome footprint signal across its exonic genomic span was calculated using bigWigSummary. ORFs with a non-zero average ribosome occupancy were considered to exhibit translational potential and were retained for downstream analyses. To obtain statistically supported evidence of translation, publicly available human Ribo-seq datasets from PRJEB28810 were analyzed, including samples from brain (cerebrum, *n* = 3), liver (*n* = 3), and testis (*n* = 3). Ribosome footprint reads were independently analyzed using PRICE and RiboCode. Predicted ORFs were matched to candidate ORFs based on genomic coordinates, strand, and exon structure, prioritizing exact matches or in-frame internal initiation events with minimal start or end offsets. The corresponding *P* values were used to quantify translational support. ORFs supported by a *P* value ≤ 1 ×  10^−5^ in either PRICE or RiboCode were considered to show strong evidence of translation.

Transcripts were defined as high-confidence bicistronic transcripts if they met all of the following criteria: both ORFs were co-located on the same transcript as supported by LrRNA-seq data; the transcript contained at least two ORFs with positive PhyloCSF scores; and at least two ORFs exhibited Ribo-seq translation signals meeting the thresholds described above.

### Plasmid construction

DNA constructs utilized in this study are detailed in the key resources table. Amplified DNA fragments were derived from genomic DNA or cDNA of HEK293T cells. The vector backbones were digested with specific restriction enzymes in the key resources table to prepare them for ligation. DNA fragments were ligated into the digested vector using a recombination-based cloning approach (Vazyme, C115-02), and the ligation product was transformed into bacteria. Guide RNA plasmids were constructed using the LentiCRISPR v2 backbone as described previously. STREMI truncation mutants were assembled via T4 DNA ligase (Vazyme, N102-01).

### Transfection and lentivirus transduction

Lipo8000 (Beyotime, C0533) was used to transfect plasmids into cells at a ratio of 1:1.6 (DNA [μg]: Lipo8000 [μL]) when cell confluency reached 60-70%, following the manufacturer’s instructions.

Lentiviral particles were produced via co-transfecting HEK293T cells with lentiviral and packaging plasmids using Lipo8000. Viral supernatant was collected 48-72 h post-transfection. For transduction, target cells were incubated with lentiviral supernatant supplemented with 8 μg/mL polybrene (Santa Cruz, sc-134220). Infected cells were subsequently selected with puromycin (1 μg/mL; Beyotime, ST551) or blasticidin (10 μg/mL; Beyotime, HY-K1054) to establish stable cell lines.

### Antibody generation

Custom polyclonal antibodies against human STREMI were generated by immunizing rabbits with a synthetic peptide spanning the N-terminal residues 2–13 (sequence: ADDKDSLPKLKD-C), where an engineered cysteine was added at position 14 to enable conjugation to keyhole limpet hemocyanin (KLH) and antigen affinity purification. Antiserum produced by HuaBio (Hangzhou, China) was validated for immunoblotting.

### Animals

All mouse experiments and housing protocols were approved by the Zhejiang University Institutional Animal Care and Use Committee (Approval No. ZJU20240378) and were conducted in strict accordance with the Guidelines for Ethical Review of the Welfare of Experimental Animals (GB/T 35892‑2018), the Laboratory animal–Guidelines for euthanasia (GB/T 39760‑2021), and Laboratory animals—General requirements for animal experiment (GB/T 35823‑2018). Mice were group-housed in the Zhejiang University Laboratory Animal Center under a 12-h light/12-h dark cycle, with ad libitum access to standard rodent chow and tap water. Wild-type C57BL/6J mice were obtained from the Shanghai Model Organisms Center, which also generated the heterozygous C57BL/6J-STREMI^+/–^ mouse line used in this study.

For the cold exposure experiment, mice were singly housed and relocated to a dedicated mice breeding incubator (Ningbo Jiangnan Instrument Factory, RXM-358B) with a controlled ambient temperature of 4 °C. Rectal core temperature was assessed at predetermined time points using a rectal probe (ZHONGJIAOJIANYI, TH-212).

### Isolation of mitochondria

Cells were trypsinized and collected in centrifuge tube, followed by two washes in PBS at 4 °C. Homogenization buffer (200 mM sucrose, 10 mM Tris-MOPS, 1 mM EGTA-Tris, pH 7.4), supplemented with 1× protease inhibitor cocktail (PIC, Beyotime, P1005) and 1 mM PMSF, was used to resuspend cells on ice for 10 min. The cell suspension was homogenized by a Dounce tissue grinder (Merck, D8938-1SET), and spun at 1000×*g* for 10 min at 4 °C. The supernatant was transferred to a clean tube and then crude mitochondria were pelleted at 12,000×*g* for 15 min at 4 °C.

### SDS–PAGE and immunoblotting

Cell or mitochondrial pellets were treated with lysis buffer (RIPA, 1 × PIC and 2 mM PMSF) on ice for 10 min, with vortexing every 3 min. The supernatant was obtained by centrifugation at 20,000 × *g* for 10 min and quantified using the enhanced BCA protein assay kit (Beyotime, P0010) according to the manufacturer’s instructions. Subsequently, the normalized protein samples were separated on SDS–PAGE gels and transferred to PVDF membranes (Millipore, ISEQ00010). The membranes were blocked in 5% nonfat milk in TBST (150 mM NaCl, 20 mM Tris base, 0.05% Tween 20, pH 7.4) for 1 h at room temperature (RT). They were then probed with the indicated primary antibodies overnight at 4 °C on a roller.

The antibodies and relevant dilutions are available in key resources table. After four washes with TBST, the membranes were incubated with HRP-conjugated secondary antibodies against mouse and rabbit (1:10,000; Jackson ImmunoResearch) for 1 h at RT on a shaker, followed by four additional washes with TBST. Chemiluminescence was detected using ECL reagent (Beyotime, P0018M) and visualized with a Chemiluminescence Imager (Tanon, 5200).

### Immunogold labeling and electron microscopy

HeLa cells were initially fixed at 4 °C overnight in a mixture of 4% paraformaldehyde and 0.1% glutaraldehyde prepared in 0.1 M phosphate buffer (PB) to preserve ultrastructure and antigenicity. Following fixation, excess aldehydes were neutralized by incubation with 50 mM glycine. Cells were then permeabilized using 0.1% Triton X-100 in PB and blocked with 0.1% BSA to reduce non-specific binding prior to antibody staining. The samples were incubated with an anti-STREMI primary antibody overnight at 4 °C, followed by incubation with a nanogold-conjugated Fab’ goat anti-rabbit IgG secondary antibody (Nanoprobes) overnight at 4 °C. After thorough phosphate buffer washes, samples were further stabilized in 2.5% glutaraldehyde, rinsed with deionized water, and equilibrated in sodium citrate buffer (pH 7.0) before silver enhancement, which was carried out for 2 min under light-protected conditions. The labeled cells were post-fixed with 1% osmium tetroxide, contrasted with 2% uranyl acetate, dehydrated through a graded ethanol series followed by acetone, and embedded in EPON 812 resin. Ultrathin sections ( ~ 90 nm) were prepared and imaged using a Tecnai G2 Spirit transmission electron microscope (Thermo Fisher Scientific).

### Immunostaining

HeLa cells were cultured in 35-mm glass-bottom dishes (CELLVIS, D35-20-1.5-N) overnight. After washing by PBS once, cells were fixed by 4% PFA (Solarbio, P1110) at RT for 20 min, followed by three washes of PBS. Cells were permeabilized and blocked in PBS containing 0.3% Triton X-100, 3% BSA, and 2% donkey serum for 1 h at RT. Samples were incubated with primary antibody diluted in staining buffer (0.1% Triton X, 3% BSA, 2% donkey serum in PBS) overnight at 4 °C. After washing the samples three times with washing buffer (PBS supplemented with 0.1% Triton X), fluorescent secondary antibodies were diluted in staining buffer (1:1000) and covered samples for 1 h at RT. Following three washes with washing buffer, cells were incubated with DAPI (500 ng/mL; Aladdin, D489987) for 5 min. All samples were imaged using an Olympus FV3000 laser confocal microscope with a 100 × oil lens.

### Seahorse mitostress test

Before seeding cells, 0.01% poly-L-lysine hydrobromide was used to treat Seahorse XF96 cell culture microplates (Agilent, 103793-100). 293 T cells were seeded at a density of 40,000 cells per well in microplates and allowed to adhere overnight in culture media at 37 °C with 5% CO_2_. The XFe96 sensor cartridge was hydrated with distilled water overnight at 37 °C without CO_2_. On the day of the assay, the cartridge was calibrated using pre-warmed Seahorse XF Calibrant solution and cells were incubated in serum-free, bicarbonate-free DMEM supplemented with 10 mM glucose, 2 mM glutamine, and 1 mM pyruvate for 1 h at 37 °C without CO_2_. The mitochondrial stress assay was performed using the Seahorse XFe96 Analyzer with sequential injections of 2.5 μM oligomycin, 1 μM FCCP, and a mixture of 1 μM rotenone and 1 μM antimycin A. OCR was measured at specified intervals before and after each injection. Protein quantification was performed post-assay using the BCA method to normalize OCR values.

### Immunoprecipitation

Mitochondria (1.8 mg of protein) were isolated from HEK293T cells overexpressing GFP and either FLAG- or StrepII-tagged proteins, solubilized in 1.2 mL immunoprecipitation (IP) buffer (150 mM NaCl, 10% glycerol, 1 mM EDTA, 25 mM Tris-HCl, pH 7.4) containing 1% digitonin, 1 mM PMSF, and 1× protease inhibitor cocktail (PIC), and incubated at 4 °C for 1 h with gentle rotation. Anti-FLAG magnetic beads (Selleck, B26101) or Strep-Tactin resin beads (Iba Lifesciences, 2-1201-002) were pre-washed twice with IP buffer supplemented with 0.1% digitonin. After solubilization, lysates were centrifuged (20,000×*g*, 15 min, 4 °C) to remove insoluble material. The supernatant was incubated with the washed FLAG or Strep-Tactin beads for 3 h at 4 °C with continuous rotation. Beads were washed extensively and eluted with 40 μL of 0.5 mg/mL 3×FLAG peptide in IP buffer containing 0.1% digitonin (for FLAG beads) or 50 mM D-biotin in elution buffer (20 mM Tris-HCL, 150 mM NaCl, pH 8) containing 0.1% digitonin (for Strep-Tactin beads). Eluates were analyzed by SDS–PAGE followed by immunoblotting.

### Mass spectrometry

Proteins purified via Strep-Tactin immunoprecipitation (as described above) were denatured in 30 μL SDT buffer (4% SDS, 100 mM DTT, 150 mM Tris-HCl, pH 8.0) and purified by ultrafiltration (10 kDa cutoff, Microcon; Merck, MRCF0R010) using UA buffer (8 M urea, 150 mM Tris-HCl, pH 8.0). Reduced cysteines were alkylated with 100 mM iodoacetamide in UA buffer (30 min, dark), followed by sequential washes with UA buffer and 25 mM NH₄HCO₃. Proteins were digested with sequencing-grade trypsin (1:50 w/w, Promega, V5111) in 25 mM NH₄HCO₃ (16 h, 37 °C). Peptides were desalted using C18 StageTips (3 mL bed volume, Sigma, S6682-40PAK), dried by vacuum centrifugation, and resuspended in 0.1% formic acid.

LC–MS/MS analysis was performed on a Q Exactive HF-X mass spectrometer (Thermo Scientific, IQLAAEGAAPFADBMBCX) coupled to an Easy-nLC 1200 system. Peptides were separated on a 50 cm analytical column (75 μm ID, 1.9-μm C18 beads; Thermo Scientific, ES903) with a 110 min gradient of 2–40% buffer B (80% acetonitrile/0.1% formic acid) at 300 nL/min. Full MS scans (300–1800 *m/z*) were acquired at 70,000 resolution (AGC target 1 × 10^6^, max injection time 50 ms), followed by HCD fragmentation of the top 20 ions (NCE 27%, resolution 17,500, AGC 1 × 10^5^, dynamic exclusion 30 s).

The raw mass spectrometry data were analyzed using Thermo Fisher Scientific’s Proteome Discoverer software (v2.2). Protein identification was performed against the UniProt human reference proteome database (reviewed) containing 20,214 entries, using default search parameters. The experimental design included two groups, STREMI and EGFP, each comprising three biological replicates. Enrichment analysis was conducted using the limma package (v3.20) in R. Linear models were fitted to the data using the ‘lmFit‘ function, followed by empirical Bayes moderation with the ‘eBayes‘ function to compute *P* values and log_2_ fold changes for each protein. Volcano plots were generated to visualize the differential expression results using the ggvolcano package (v0.1.4, https://github.com/BioSenior/ggVolcano) in R.

### Transmission electron microscopy

HeLa and 293 T cells were cultured in tissue culture dishes until reaching 80% confluency and trypsinized. The cells were washed with PBS containing 5% FBS, pelleted by centrifugation at 728×*g*, and subsequently washed once with PBS. Cell pellets were fixed overnight at 4 °C in 2.5% glutaraldehyde in PBS, followed by three washes with PBS. For tissue samples, fresh tissues were dissected into 1 mm³ cubes and immediately fixed in 2.5% glutaraldehyde in PBS at 4 °C overnight, followed by three washes with PBS. The fixed cell pellets and tissue samples were then stained with 1% osmium tetroxide at RT (1 h for cells, 1.5 h for tissues) and washed three times with deionized water. To ensure optimum contrast, samples were treated with 2% uranyl acetate for 30 min at RT. Subsequently, samples were dehydrated through a series of ethanol solutions (50%, 70%, and 90% for 15 min each, followed by two washes in 100% ethanol for 20 min at RT). Further dehydration was carried out using 100% acetone twice for 20 min each at RT. The samples were sequentially infiltrated with a 1:1 (vol/vol) mixture of acetone and epoxy resin for 2 h, followed by 100% epoxy resin for 48 h at 37 °C for polymerization. Thin sections (100 nm) were prepared and supported on copper grids. Post-staining was performed using Sato lead solution for 1 min, and the stained sections were imaged using a Thermo Scientific Talos L120C microscope.

Quantification of mitochondrial ultrastructure was performed on anonymized and randomly coded images. Two independent investigators, blinded to sample identity and experimental group allocation, conducted the measurements.

### STED microscopy of mitochondria

For live-cell imaging, HeLa cells were stained with 200 nM PK Mito Orange at 37 °C and 5% CO₂ for 30 min, followed by two washes with prewarmed DMEM complete media (37 °C). For fixed-cell imaging, schemes were implemented as previously described (Chen et al, 2024). Briefly, HeLa cells were fixed with 2% pre-warmed (37 °C) Glutaraldehyde (GA) for 20 s, replaced with prewarmed 4% PFA in PBS for 8 min at RT, and then washed thrice with PBS. Cells were permeabilized with 0.25% Triton X-100 in PBS and blocked using PBS containing 0.01% Triton X-100, 3% BSA, and 2% FBS, followed by an additional wash with PBS. Following blocking, cells were incubated with primary antibodies in staining buffer (0.01% Triton X-100, 3% BSA, 2% FBS in PBS) overnight at 4 °C and washed thrice with PBS. Secondary antibody incubation was carried out at RT for 1 h, followed by three washes with PBS. The samples were subsequently prepared for STED imaging.

Images were acquired using a Facility Line microscope (Abberior Instruments) equipped with an Olympus UPlanXAPO 60× oil/NA1.42 objective. PK Mito Orange was excited at a wavelength of 561 nm, and STED imaging was performed using a pulsed depletion laser at 775 nm wavelength with a gating of 1 to 7 ns and dwell time of 5 μs. Pixel sizes of 20 nm were utilized for STED nanoscopy, with each line scanned three times (line accumulations).

### Quantitative real-time PCR (qRT–PCR)

Total RNA was isolated from cells using RNA Isolation Kit (Vazyme, RC101-01). For NMD inhibition experiments, cells were treated with the NMD inhibitor NMDI-14 (MCE, HY-111374) at a final concentration of 5 μM for 12 h prior to RNA extraction. qRT–PCR was performed using HiScript III All-in-one RT SuperMix (Vazyme, R333-01) and Taq pro Universal SYBR qPCR Master Mix (Vazyme, Q712-02) according to the manufacturer’s instructions. Data were analyzed and normalized relative to GAPDH expression values. Primers for qPCR assays in this study have been deposited in the Reagents and tools table.

### Sodium carbonate extraction

Sodium carbonate extraction was performed following established protocols with minor modifications (Fujiki et al, 1982). Mitochondria were isolated using homogenization buffer and centrifuged at 12,000×*g* for 15 min to obtain the pellet. The pellet was then resuspended in 0.1 M Na_2_CO_3_ at pH 10.5, 11, or 11.5, or in isotonic buffer containing 1% Triton X-100 and 1% digitonin, and incubated at 4 °C for 1 h. The suspension was subjected to ultracentrifugation at 100,000× *g* for 30 min in thick-wall polycarbonate tubes (Beckman, 343778) using a MLA-150 rotor at 4 °C. The supernatant was collected, and the pellet was resuspended in the same volume of buffer. Both the supernatant and pellet fractions were analyzed by western blotting.

### Protease sensitivity assay

Protein topology was assessed through proteolysis assays following established methodologies (Zhang et al, 2023). After isolation of mitochondria from HEK293T cells using homogenization buffer containing 1 × PIC, the mitochondrial pellet was resuspended in homogenization buffer without PIC at a protein concentration of 1 mg/mL. Mitochondrial membranes were solubilized with varying concentrations of digitonin (0, 0.02%, 0.04%, 0.06%, 0.08%, 0.1%, 0.15%, 0.2%) or 1% Triton X-100. Proteinase K (Thermo Fisher Scientific, ST533) was added at a concentration of 100 μg/mL to samples treated on ice with detergent for 10 min, followed by incubation on ice for an additional 30 min. An additional sample without digitonin and Proteinase K was included. PMSF was freshly prepared and added to terminate digestion at a concentration of 8 mM.

### Mitochondrial split-GFP localization

GFP1-10 was targeted using: (1) the N terminus (68 aa) of mouse LACTB (intermembrane space), (2) the presequence (29 aa) of human COX8A (matrix), or (3) the C terminus (39 aa) of human OMP25 (outer membrane), as we previously described (Zhang et al, 2023). STREMI, fused to GFP11 at its N or C terminus, was co-expressed pairwise with these constructs in HeLa cells. Compartment-specific reconstitution was monitored by confocal microscopy.

### Protein stability analysis by cycloheximide chase

HEK293T cells were transiently transfected with constructs encoding STREMI mutants. At 48 h post-transfection, protein synthesis was inhibited by treating cells with 50 μg/mL cycloheximide for 0 and 8 h. At each time point, cells were harvested and protein levels were analyzed by immunoblotting. Band intensities were quantified using ImageLab software (Bio-Rad) to assess protein degradation kinetics.

### Expression and purification of STREMI

STREMI was expressed and purified using a protocol adapted from the previous study (He et al, 2024). The pET21a (+) plasmid encoding STREMI with 6×His tag was synthesized by General BIOL and transformed into *E. coli* Rosetta (DE3) cells. A single colony was inoculated into 5 mL of LB medium containing 100 μg/mL ampicillin and cultured overnight at 37 °C with shaking (250 rpm). The culture was then used to inoculate 100 mL of LB medium containing the same antibiotic and incubated overnight at 37 °C. The culture was scaled up to 1 L of LB medium with antibiotic and grown at 37 °C with shaking until the OD_600_ reached 0.4. Growth was continued until OD_600_ reached 0.9–1, after which protein expression was induced with 500 μM IPTG for 6 h at 37 °C with shaking. Cells were harvested by centrifugation at 5000 rpm for 6 min at 4 °C, resuspended in 80 mL of ice-cold PBS containing 0.002% PMSF, 1 mg/mL lysozyme, and 5 μg/mL DNase I, and incubated for 30 min at 4 °C. The suspension was subjected to sonication using a microprobe with a 115-μm tip, applying 300 W output for 3 s, with 5-second intervals, repeated for a total of 20 min.The homogenate was centrifuged at 12,000×*g* for 15 min at 4 °C, and the supernatant was filtered through a 0.45 μm filter. The filtrate was then loaded onto a 5 mL HisTrap Excel column equilibrated with buffer A (PBS containing 20 mM imidazole and 0.05% Triton X). The column was washed with 25 mL of buffer A and eluted with a 0–60% gradient of buffer B (PBS containing 500 mM imidazole and 0.05% Triton X) over 20 column volumes. Fractions exhibiting high absorbance at 280 nm were pooled and concentrated using a 10 kDa MWCO centrifugal concentrator. The concentrate was then subjected to size-exclusion chromatography on a Superose 6 Increase 10/300 GL column using SEC buffer (20 mM Tris pH 7.4, 50 mM NaCl, 0.002% PMSF, and 0.015% GDN). Following confirmation via SDS–PAGE and Coomassie staining, the purified STREMI was aliquoted and stored for downstream functional studies.

### Mass photometry

Mass photometry measurements were conducted at RT employing a Refeyn TwoMP mass photometer (Refeyn Ltd). Sample analysis was performed in six-chambered silicone gaskets mounted on sample carrier slides. Purified STREMI protein was diluted to a concentration of 20 nM using phosphate-buffered saline as the experimental buffer. The measurement consisted of 60 s continuous video acquisition. Raw interferometric scattering data were processed through the Refeyn-provided software. The resulting mass distributions were statistically analyzed through Gaussian peak fitting and visualized as normalized frequency distributions.

### Untargeted metabolomics analysis

Metabolites were extracted from 20 mg of mice BAT using 400 μL methanol/water (7:3, v/v) solution containing internal standards, followed by vortexing and incubation on ice. After centrifugation at 4 °C, the supernatant was collected, further clarified by cooling and centrifugation, and aliquots were subjected to LC–MS analysis. Chromatographic separation was performed on a Waters ACQUITY Premier HSS T3 column (1.8 μm, 2.1 × 100 mm) at 40 °C with a flow rate of 0.4 mL/min. Mobile phase A consisted of water with 0.1% formic acid, and mobile phase B consisted of acetonitrile with 0.1% formic acid. A gradient elution was applied, and samples were analyzed in both positive and negative electrospray ionization modes. Mass spectrometry data were acquired using a Q Exactive platform with full-scan MS over an *m/z* range of 70–1000 at 60,000 resolution, combined with data-dependent MS/MS acquisition.

Raw LC–MS data were processed for peak detection, alignment, and normalization prior to statistical analysis. Metabolite intensities were log2-transformed and mean-centered to reduce systematic variation. Differential metabolites between the two groups were identified based on variable importance in projection (VIP > 1) derived from orthogonal partial least squares discriminant analysis (OPLS–DA) and statistical significance determined by Student’s *t* test (*P* < 0.05). Model robustness was evaluated using permutation testing (200 permutations) to minimize overfitting. Heatmaps and volcano plots were generated in R to visualize differential metabolite patterns.

### Dual-luciferase reporter assay

HEK293T cells were seeded in 24-well tissue culture dishes and transfected with pMIRGLO vectors. Cell extracts were prepared 48 h after transfection, and luciferase activity was measured with the Dual Luciferase Reporter Gene Assay Kit (YEASEN, 11402ES60) according to the manufacturer’s instructions.

### CCK-8 assay

HEK293T cells were seeded in 96-well tissue culture dishes at a density of 1 × 10⁴ cells/well. 10 μL of CCK-8 reagent (40203ES60) was added to each well after 48 h or 72 h transfection, and OD_450_ was recorded after 1 h incubation in 37 °C. Cell viability was calculated as:

$${V}=\frac{{{{{\rm{OD}}}}}_{{{{\rm{treat}}}}}-{{{{\rm{OD}}}}}_{{{{\rm{blank}}}}}}{{{{{\rm{OD}}}}}_{{{{\rm{control}}}}}-{{{{\rm{OD}}}}}_{{{{\rm{blank}}}}}}$$

### RNA sequencing analysis of SLC35A4 overexpression in HEK293T cells

HEK293T cells were transiently transfected with either an empty vector or SLC35A4 overexpression plasmid. At 48 h post-transfection, cells were harvested (5 × 10⁶ cells per condition), and total RNA was isolated using the FastPure Cell/Tissue Total RNA Isolation Kit (Vazyme, RC101-01) with on-column DNase I digestion. RNA integrity was verified by Bioanalyzer 2100 (Agilent, RNA Integrity Number [RIN] > 9.0). Polyadenylated mRNAs were enriched through two rounds of hybridization with oligo(dT) magnetic beads. The purified mRNA was fragmented into 200-300 bp fragments using divalent cations under elevated temperature conditions, followed by first-strand cDNA synthesis with random hexamers and reverse transcriptase. Second-strand cDNA was generated using NEBNext Ultra RNA Library Prep Kit for Illumina (NEB, E7530). The resulting double-stranded cDNA underwent end repair, dA-tailing, and ligation with Illumina-compatible adapters. Adapter-ligated products were purified using AMPure XP Beads (Beckman Coulter, A63881) at 1.0 × ratio, followed by 8-cycle PCR amplification. Quality-controlled libraries were sequenced on the Illumina NovaSeq 6000 platform (Astrocyte Technology) with paired-end 150 bp configuration.

The raw reads in fastq format underwent quality control via FastQC (v0.12.1) with MultiQC (v1.16) visualization to assess base-level errors and adapter contamination (Ewels et al, 2016). Clean reads were obtained by removing adapter sequences and low-quality bases using Trim Galore (v0.6.10, https://github.com/FelixKrueger/TrimGalore) under stringent thresholds (Phred score cutoff: 20, minimum read length: 36 bp, sliding window quality evaluation: 4-bp). Read alignment was performed against the Homo sapiens GRCh38 reference genome using HISAT2 (v2.2.1) in splice-aware mode with default mismatch parameters (Kim et al, 2019). Gene-level quantification was conducted through featureCounts (v2.0.6) from the Subread package, requiring both paired-end reads to map unambiguously to exonic regions (--pairwise flag) as annotated in GENCODE v44 (Liao et al, 2014).

Differential expression analysis between experimental groups was performed using DESeq2 (v1.40.2) with default dispersion estimation parameters (Love et al, 2014). Genes were classified as differentially expressed when meeting dual thresholds (adjusted *P* < 0.001 via Benjamini–Hochberg correction and absolute log_2_ fold-change >1.5). Enrichment analysis for Gene Ontology (GO) terms was conducted via the clusterProfiler (v4.8.1) R package using biological process annotations(Yu et al, 2012). Transcriptional regulator activity was predicted using the decoupleR (v2.8.0) R package with the dorothea TF-target interaction database (Badia et al, 2022).

### Phylogenetic tree construction of SLC35A4 and STREMI genes

Homologous protein sequences of SLC35A4 and STREMI were retrieved using the NCBI BLASTp tool against the non-redundant (nr) protein database with an E-value threshold of 1 × 10^−5^. Multiple sequence alignment was conducted using MAFFT (v7.505) (Katoh et al, 2002), and poorly aligned regions were removed using trimAl (v1.4.rev15) with default parameters (Capella-Gutiérrez et al, 2009). Phylogenetic trees were inferred using IQ-TREE (v2.1.4) with 1000 ultrafast bootstrap replicates to assess branch support (Minh et al, 2020). Taxonomic hierarchy information for each species was obtained via the NCBI Entrez API, and the final trees were visualized using iTOL (Letunic and Bork, 2021).

To identify SLC35A4 orthologs, we downloaded reference protein sequences for solute carrier family 35 member A4 from OrthoDB at the Vertebrata level (orthogroup 757497at7742). We then used BLASTp (BLAST v2.9.0) to search predicted proteomes derived from the genomes listed in Dataset EV4, with an E-value cutoff of 1 × 10^−5^. In parallel, we performed HMMER searches using the Pfam domain PF04142 with an E-value threshold of 1 × 10^−5^. Only sequences supported by both BLASTp and HMMER were retained as candidate SLC35A4 proteins. Because these searches also retrieved other SLC35A family members, we constructed a protein family phylogeny in IQ-TREE to identify candidates that cluster most closely with annotated SLC35A4 orthologs.

For comprehensive identification of STREMI homologs in lamprey and hagfish, we performed BLASTN and TBLASTN searches using the coding sequence (XM_032980917.2) and protein sequence (XP_032836808.1) of *Petromyzon marinus* as queries.

### Evolutionary analysis of bicistronic transcripts across species

To assess the evolutionary conservation of bicistronic transcript structures, we performed tblastn searches using human bicistronic ORF pairs against RefSeq genome assemblies of target species. We examined whether homologous regions were located adjacent. Gene structure annotations from each species were integrated to confirm whether the homologous ORF pairs were transcribed as bicistronic transcripts.

### Analysis of native protein complexes

Blue native electrophoresis was performed as published with minor modifications (Wittig et al, 2006). Isolated mitochondria pellets were solubilized using solubilization buffer B (50 mM imidazole/HCl, 500 mM 6-aminohexanoic acid, 1 mM EDTA, pH 7) containing digitonin at a ratio of 4 mg/g (digitonin/mitochondrial protein) and 1 × PIC for 20 min on ice. The samples were centrifuged at 20,000×*g* for 15 min at 4 °C, and the lysate was quantified using a BCA assay and normalized accordingly. Prior to loading, 0.125% Brilliant Blue G (G250, Sangon, A600038-0025) and 10% glycerol were added to the lysate, which was then loaded onto a 3–12% Native PAGE gradient gel. Electrophoresis was performed at 150 V for 30 min and 250 V for 1 h, using dark blue and light blue running buffers, respectively, at 4 °C. Proteins were transferred to PVDF membranes (Millipore, ISEQ00010). The membranes were fixed with 8% acetic acid for 10 min, followed by three washes with water. After drying, the membranes were blocked with 5% milk in TBST for 1 h at RT, then incubated with the primary antibody for 1 h at RT. The remaining steps followed standard western blotting protocols.

For second-dimension separation, BN-PAGE gel lanes were excised and incubated in an SDS-containing buffer (1% SDS) at 37 °C for 15 min. The gel strips were then placed horizontally on top of SDS–PAGE gels after removal of the central stacking gel wells, while wells at both sides were retained for loading molecular weight markers. Proteins were subsequently resolved by SDS–PAGE and analyzed by immunoblotting as described above.

### Assessment of CIII activity

Mitochondrial Complex III activity was assayed spectrophotometrically by monitoring the reduction of oxidized cytochrome c at 550 nm, following a modified protocol (Spinazzi et al, 2012). Briefly, isolated mitochondria (250 µg/mL protein) were pre-incubated at RT in a reaction mixture containing 50 mM NaN₃ (to inhibit Complex IV) and 500 µM freshly reduced decylubiquinol (DQH₂, prepared via potassium borohydride reduction). The baseline absorbance at 550 nm was recorded for 5 min. The reaction was initiated by adding 50 µM oxidized cytochrome c, and the absorbance increase was monitored for 5 min. Control reactions included 0.01 mg/mL antimycin A to determine non-specific activity, while the experimental group received equivalent DMSO.

### Statistical analysis

Data analysis and visualization were conducted using GraphPad Prism (v10.4.0) and the ggplot2 package in R, with experimental sample sizes explicitly annotated in corresponding figure legends. Quantitative results are presented as mean ± standard error of the mean (SEM). All tests were two-tailed with a significance threshold of 0.05. For all comparisons, statistical significance was determined using appropriate tests, as specified in the figure legends. Box plot elements were configured as follows: the central horizontal line indicates the median (50th percentile); the box spans from the 25th (Q1) to 75th percentile (Q3); whiskers extend to the absolute minimum and maximum values within the dataset, intentionally retaining all observed data points.

## Supplementary information

Peer Review File

Dataset EV1

Dataset EV2

Dataset EV3

Dataset EV4

Dataset EV5

Dataset EV6

Source data Fig. 1

Source data Fig. 2

Source data Fig. 3

Source data Fig. 4

Source data Fig. 5

Source data Fig. 6

Source data Fig. 7

Expanded View Figures

## Data Availability

Raw proteomics data have been deposited in OMIX at the National Genomics Data Center (NGDC), China National Center for Bioinformation/Beijing Institute of Genomics, Chinese Academy of Sciences under accession number OMIX009810. Raw metabolomics data have also been deposited in OMIX under accession number OMIX015204. Raw RNA-seq data are available in the Genome Sequence Archive under accession number HRA010978 at the National Genomics Data Center. Scripts for data analysis have been deposited in figshare (10.6084/m9.figshare.31899904). The source data of this paper are collected in the following database record: biostudies:S-SCDT-10_1038-S44319-026-00783-8.
