# Supplementary figures and images for: STREMI: a dual-function upstream ORF-encoded regulator of mitochondrial cristae architecture (part 1 of 2)
Source: EMBO Rep. 2026 May 2;27(12):3303–39. doi: 10.1038/s44319-026-00783-8 (PMC13303939; doi:10.1038/s44319-026-00783-8)

Source Data Fig. 2

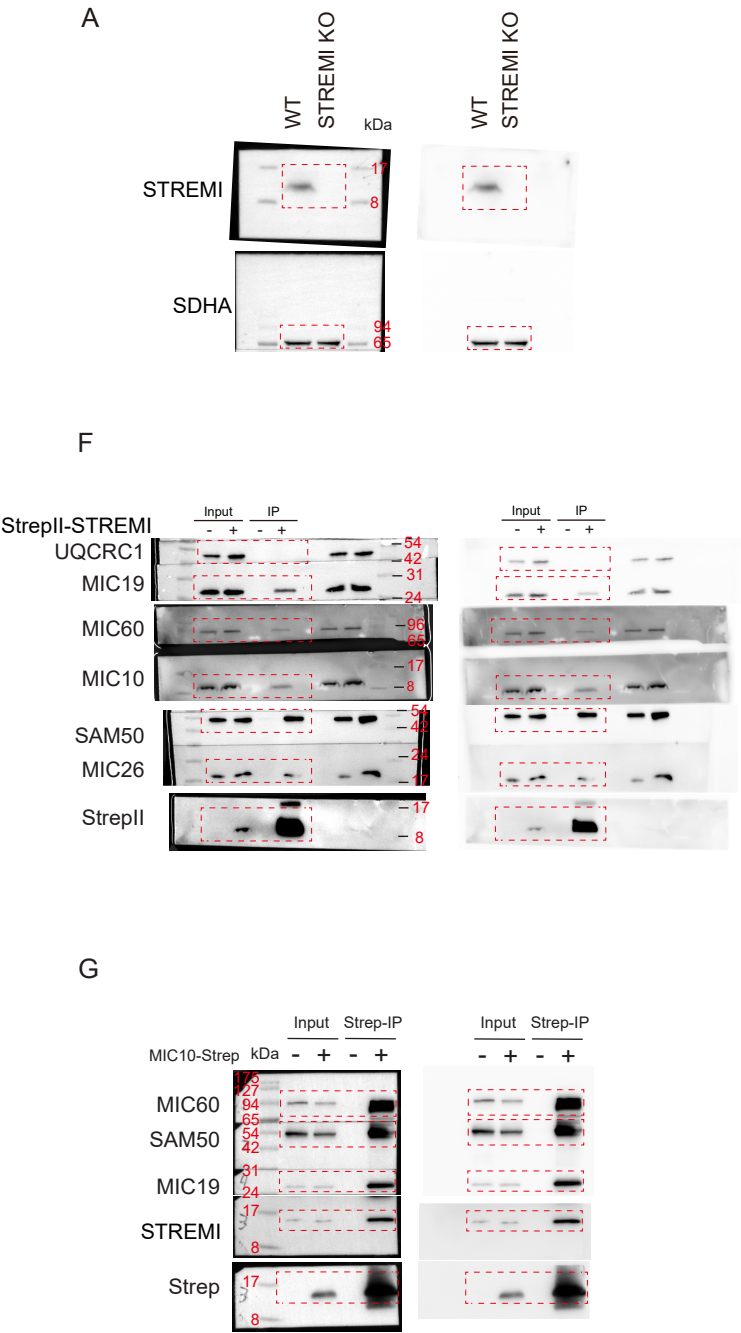

Supplement: Supplementary file 9 — Source data Fig. 2 [file 44319_2026_783_MOESM9_ESM.zip › Figure2/Figure_2_A_F_G_(Cropped_area_and marker).pdf]

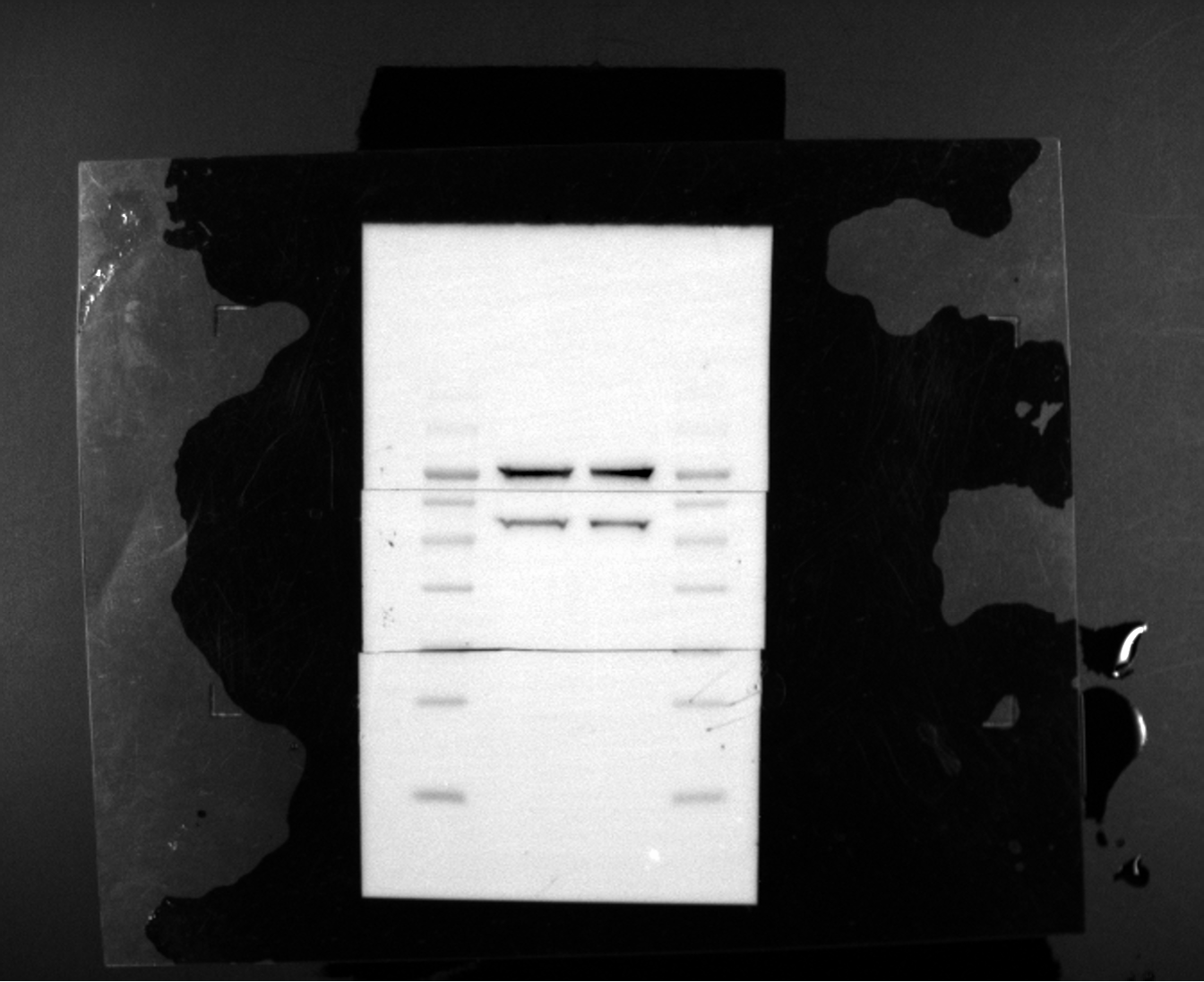

Supplement: Supplementary file 9 — Source data Fig. 2 [file 44319_2026_783_MOESM9_ESM.zip › Figure2/2A/Figure_2A- SDHA-merge_Raw_data.tif]

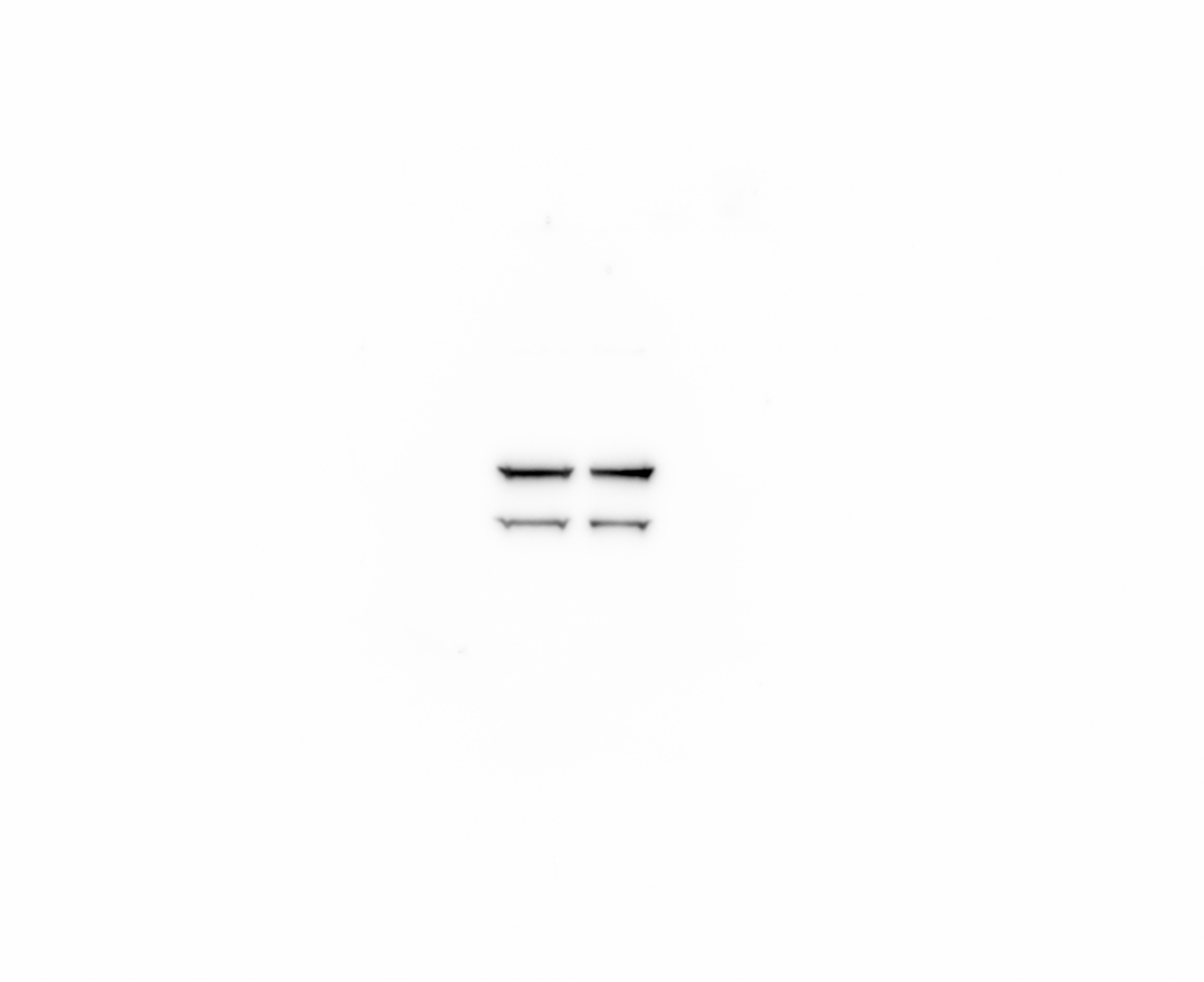

Supplement: Supplementary file 9 — Source data Fig. 2 [file 44319_2026_783_MOESM9_ESM.zip › Figure2/2A/Figure_2A- SDHA-Raw_data.tif]

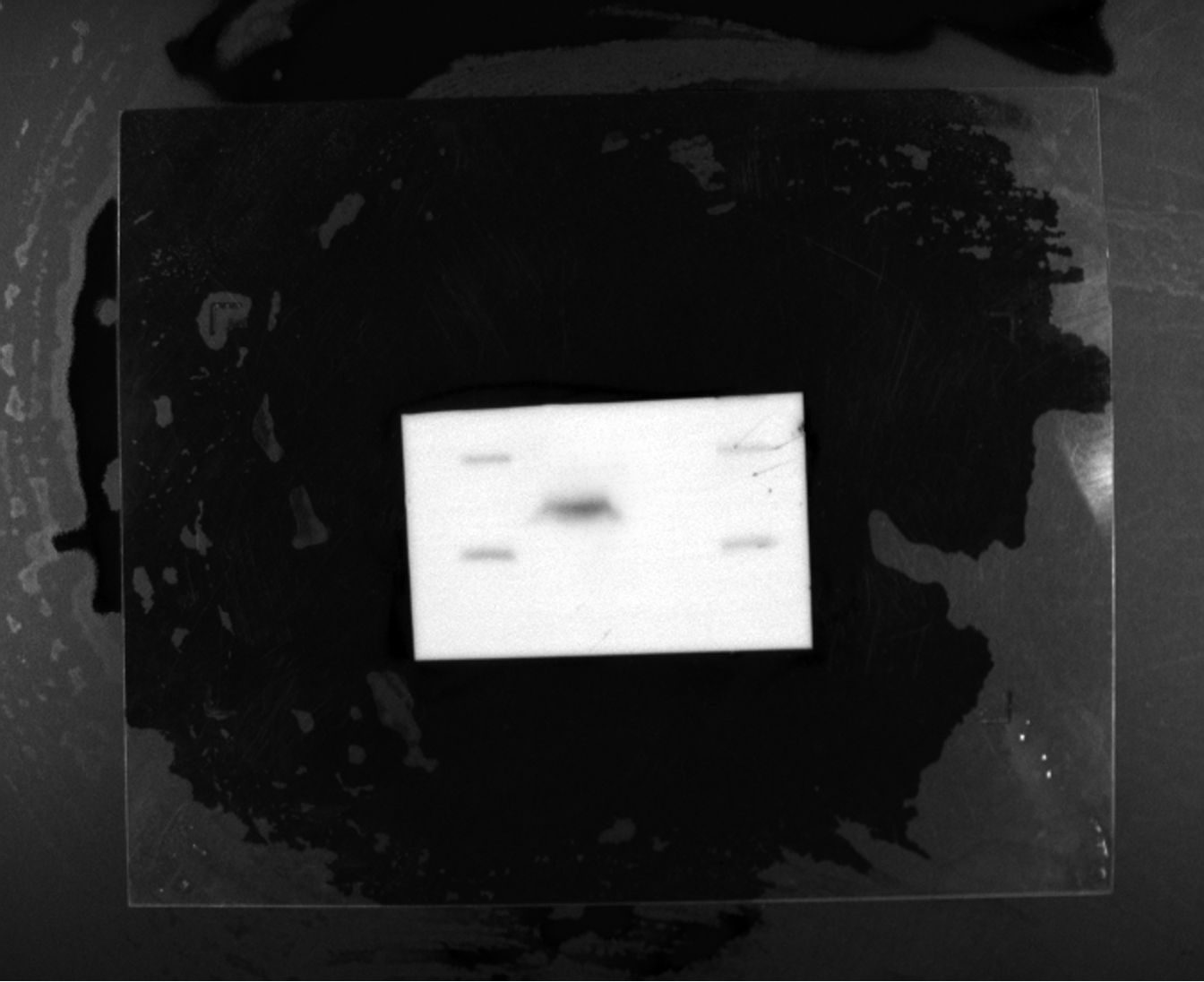

Supplement: Supplementary file 9 — Source data Fig. 2 [file 44319_2026_783_MOESM9_ESM.zip › Figure2/2A/Figure_2A- STREMI-merge_Raw_data.tif]

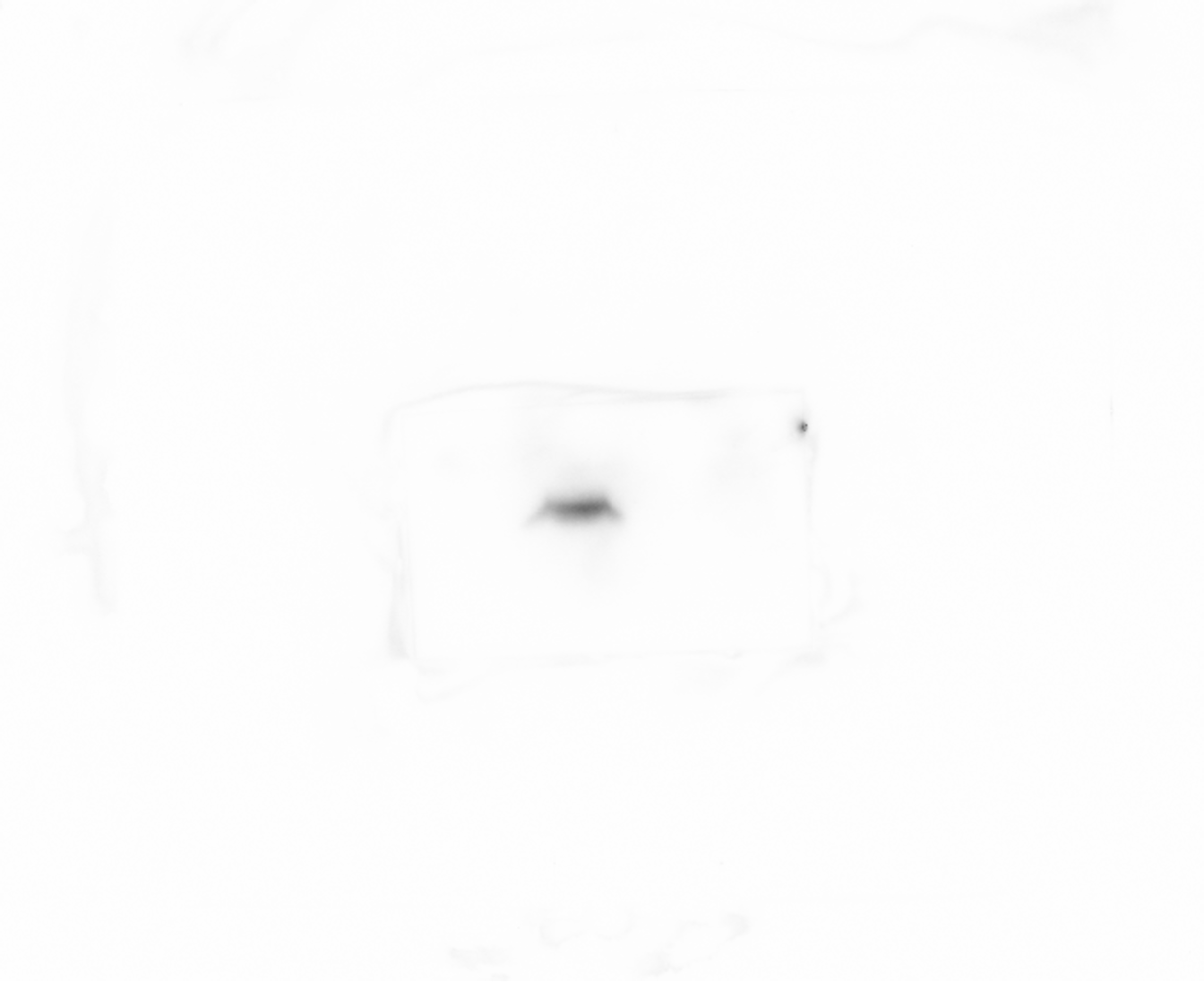

Supplement: Supplementary file 9 — Source data Fig. 2 [file 44319_2026_783_MOESM9_ESM.zip › Figure2/2A/Figure_2A- STREMI-Raw_data.tif]

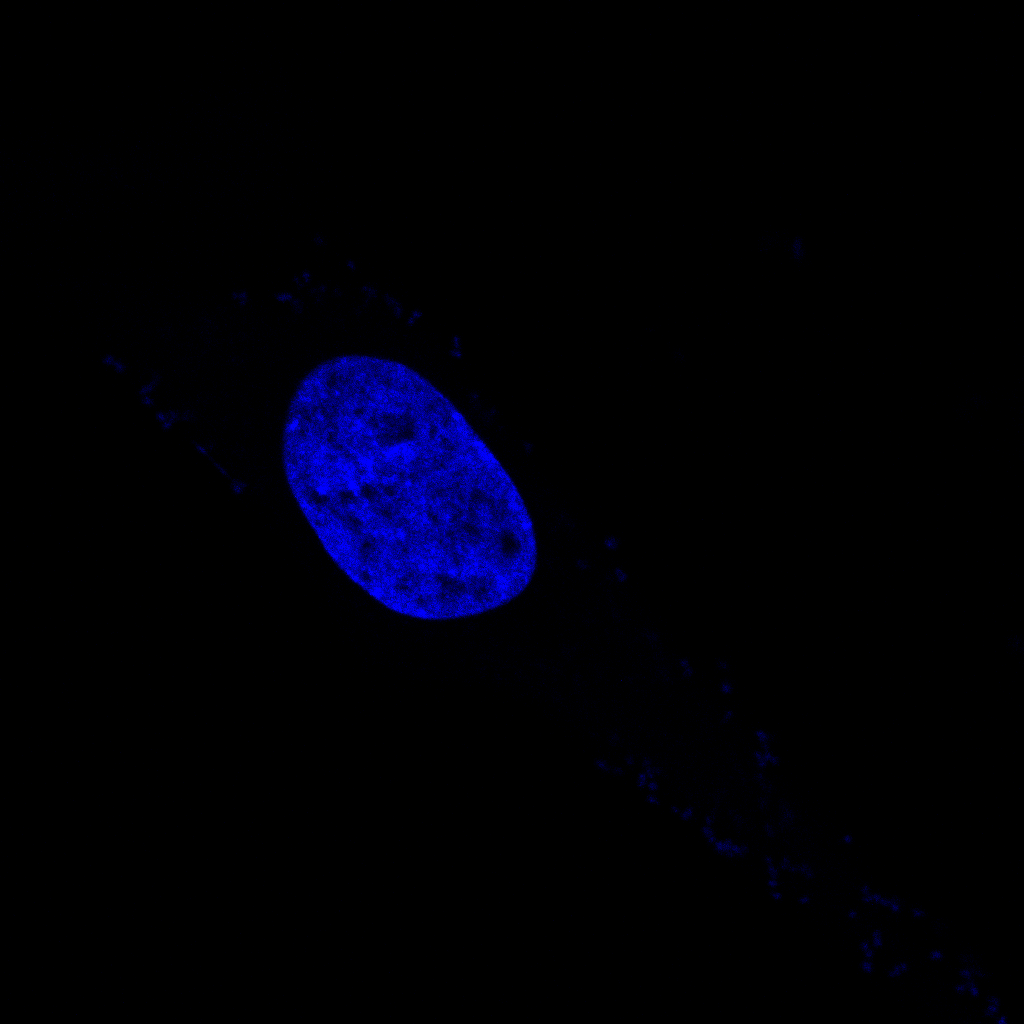

Supplement: Supplementary file 9 — Source data Fig. 2 [file 44319_2026_783_MOESM9_ESM.zip › Figure2/2B/DAPI.tif]

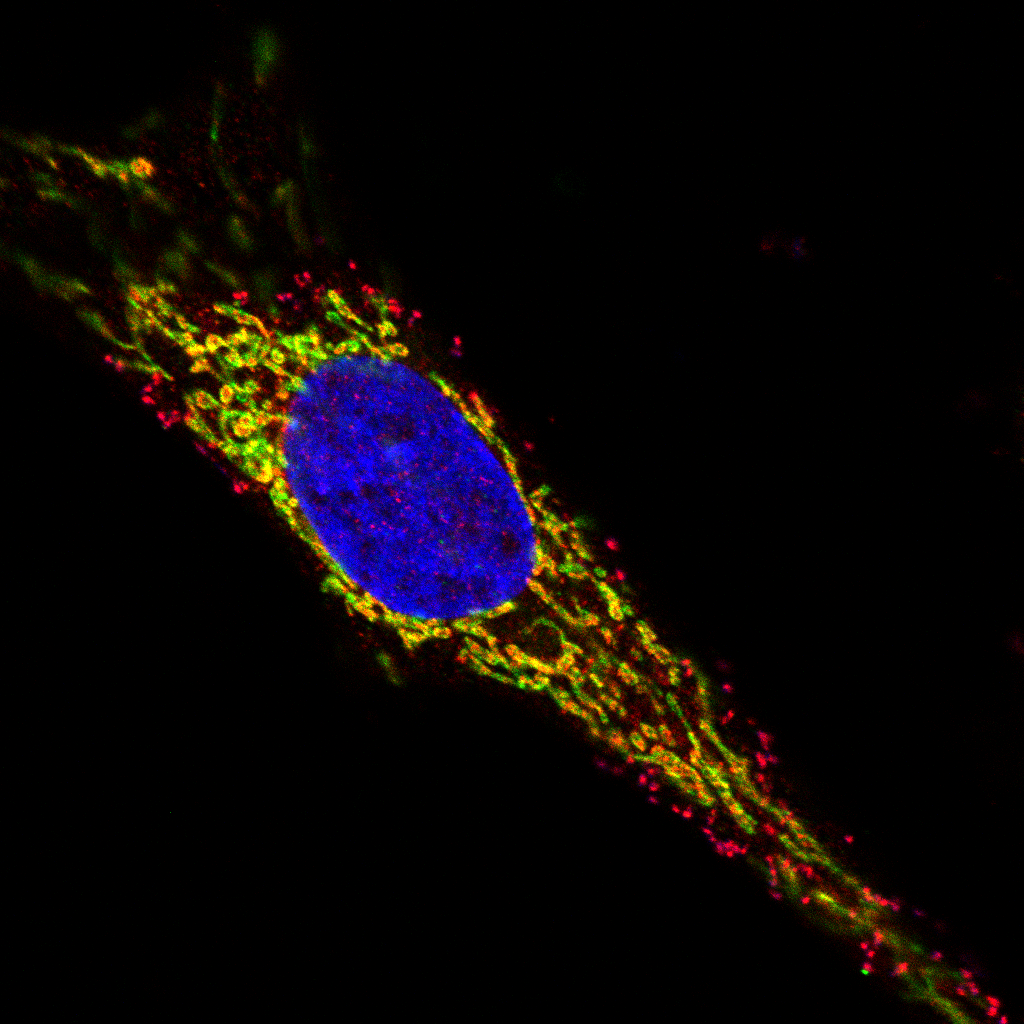

Supplement: Supplementary file 9 — Source data Fig. 2 [file 44319_2026_783_MOESM9_ESM.zip › Figure2/2B/Merge.tif]

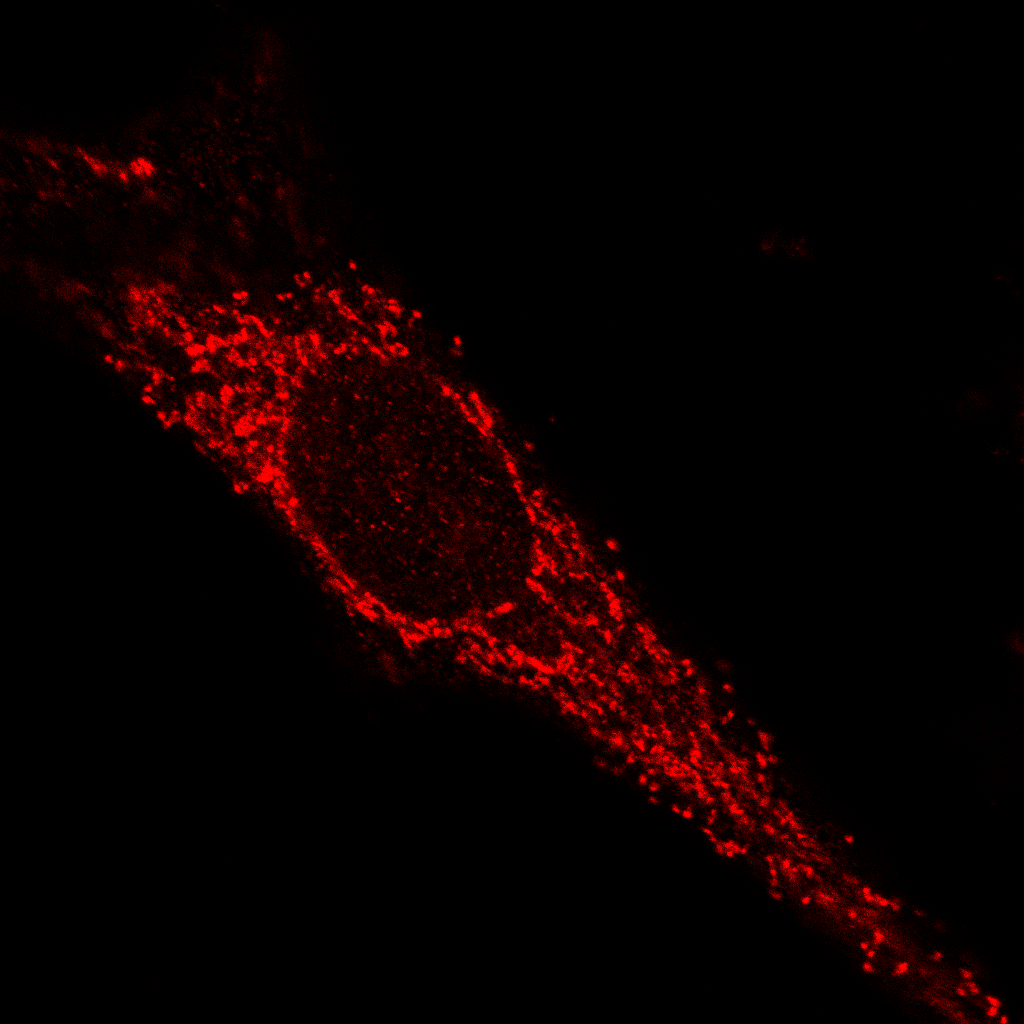

Supplement: Supplementary file 9 — Source data Fig. 2 [file 44319_2026_783_MOESM9_ESM.zip › Figure2/2B/STREMI.tif]

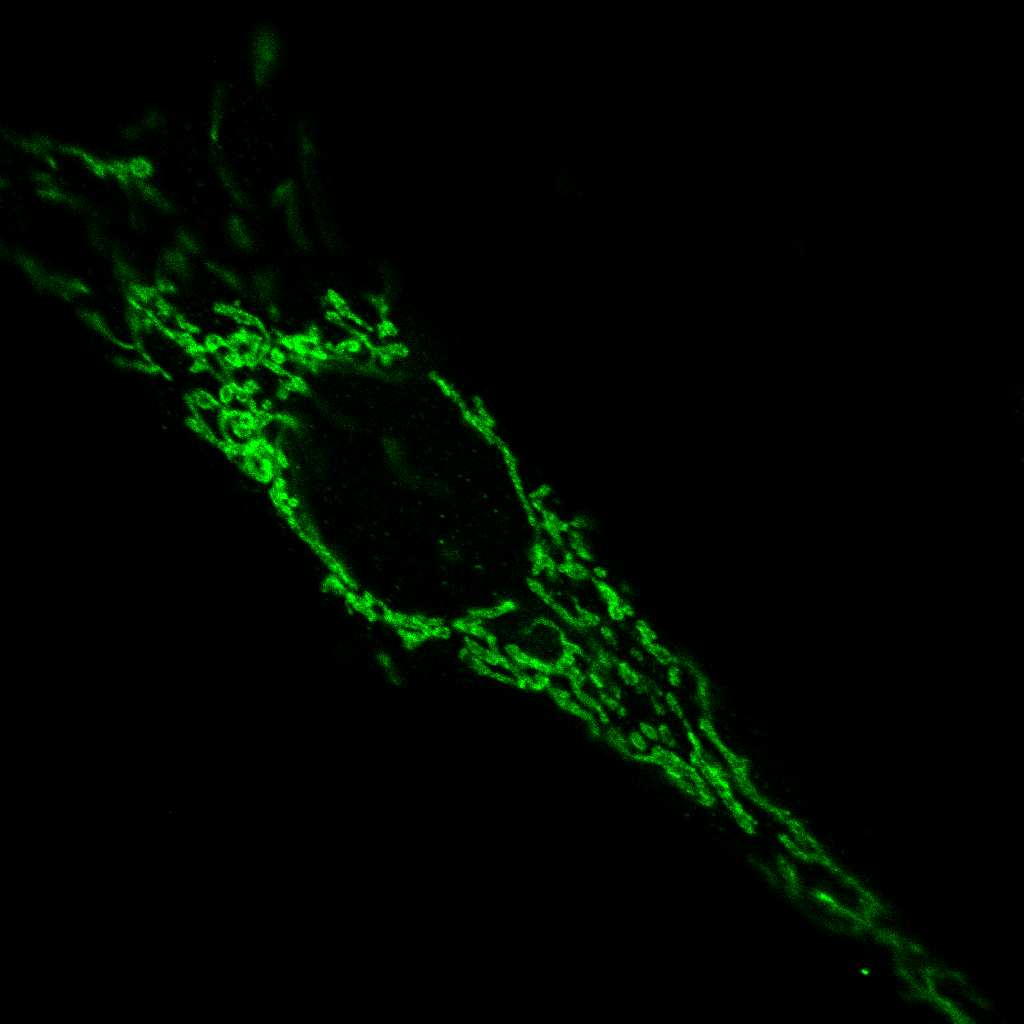

Supplement: Supplementary file 9 — Source data Fig. 2 [file 44319_2026_783_MOESM9_ESM.zip › Figure2/2B/TOM20.tif]

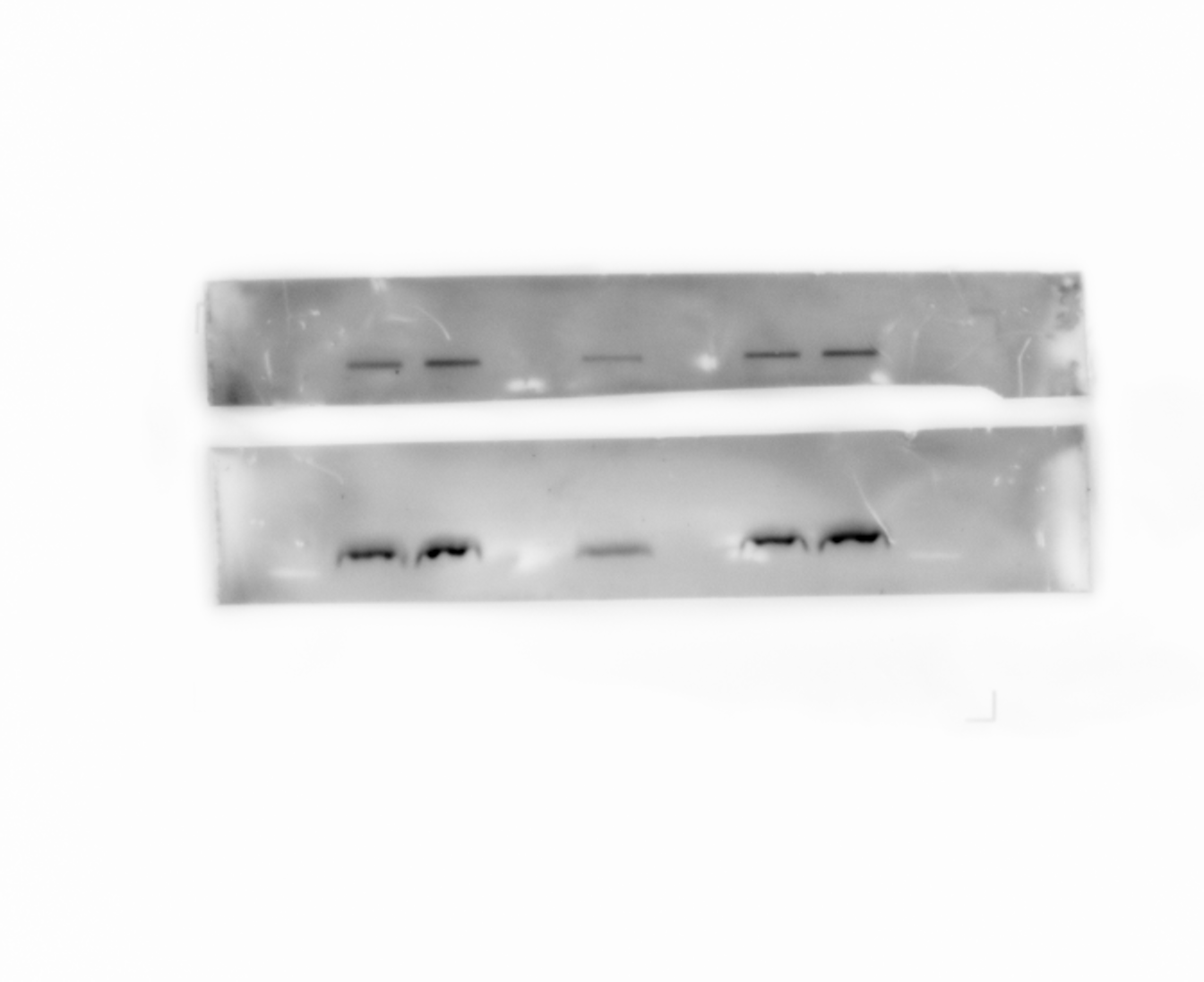

Supplement: Supplementary file 9 — Source data Fig. 2 [file 44319_2026_783_MOESM9_ESM.zip › Figure2/2F/MIC60-MIC10--RAW.Tif]

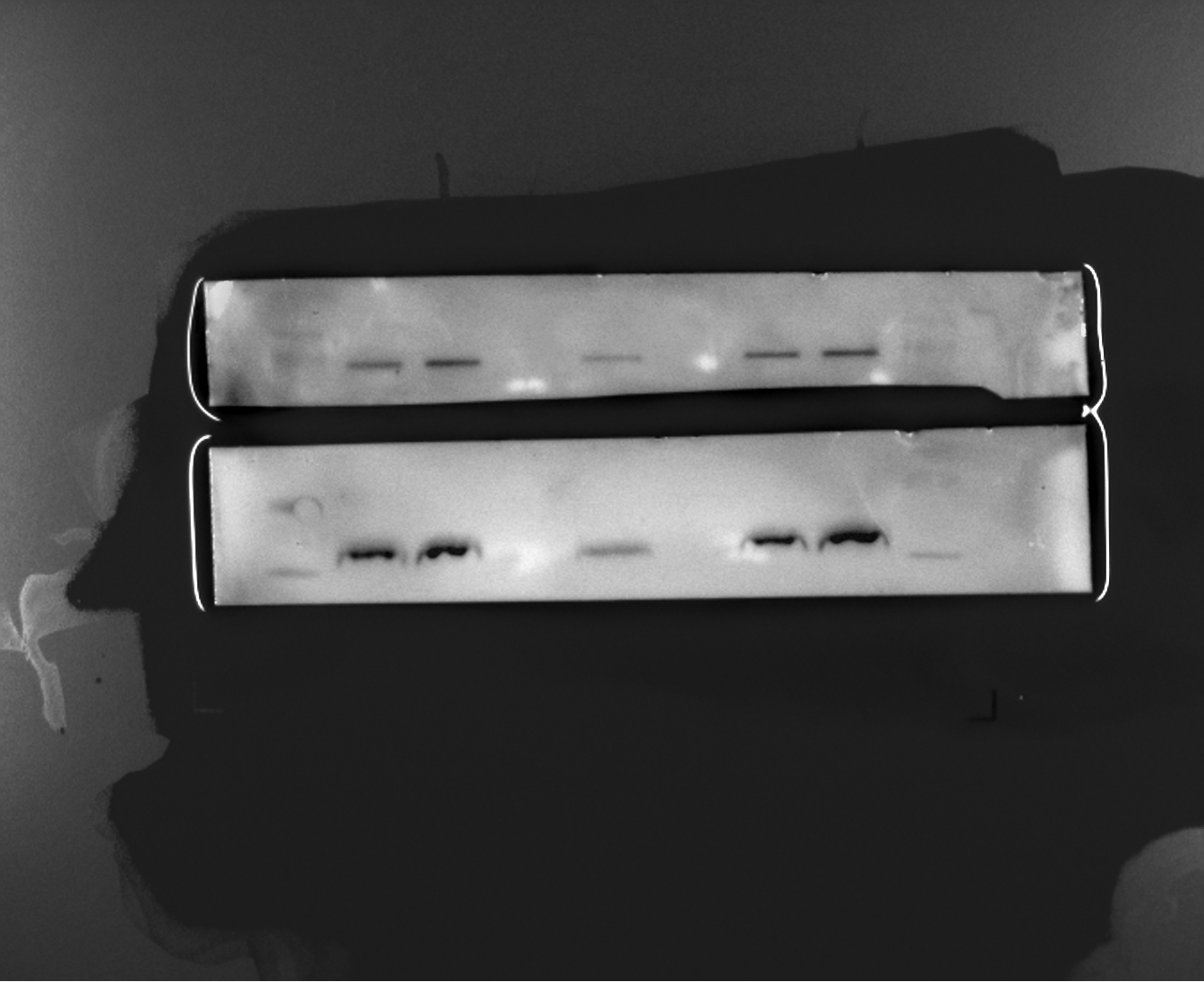

Supplement: Supplementary file 9 — Source data Fig. 2 [file 44319_2026_783_MOESM9_ESM.zip › Figure2/2F/MIC60-MIC10-merge.tif]

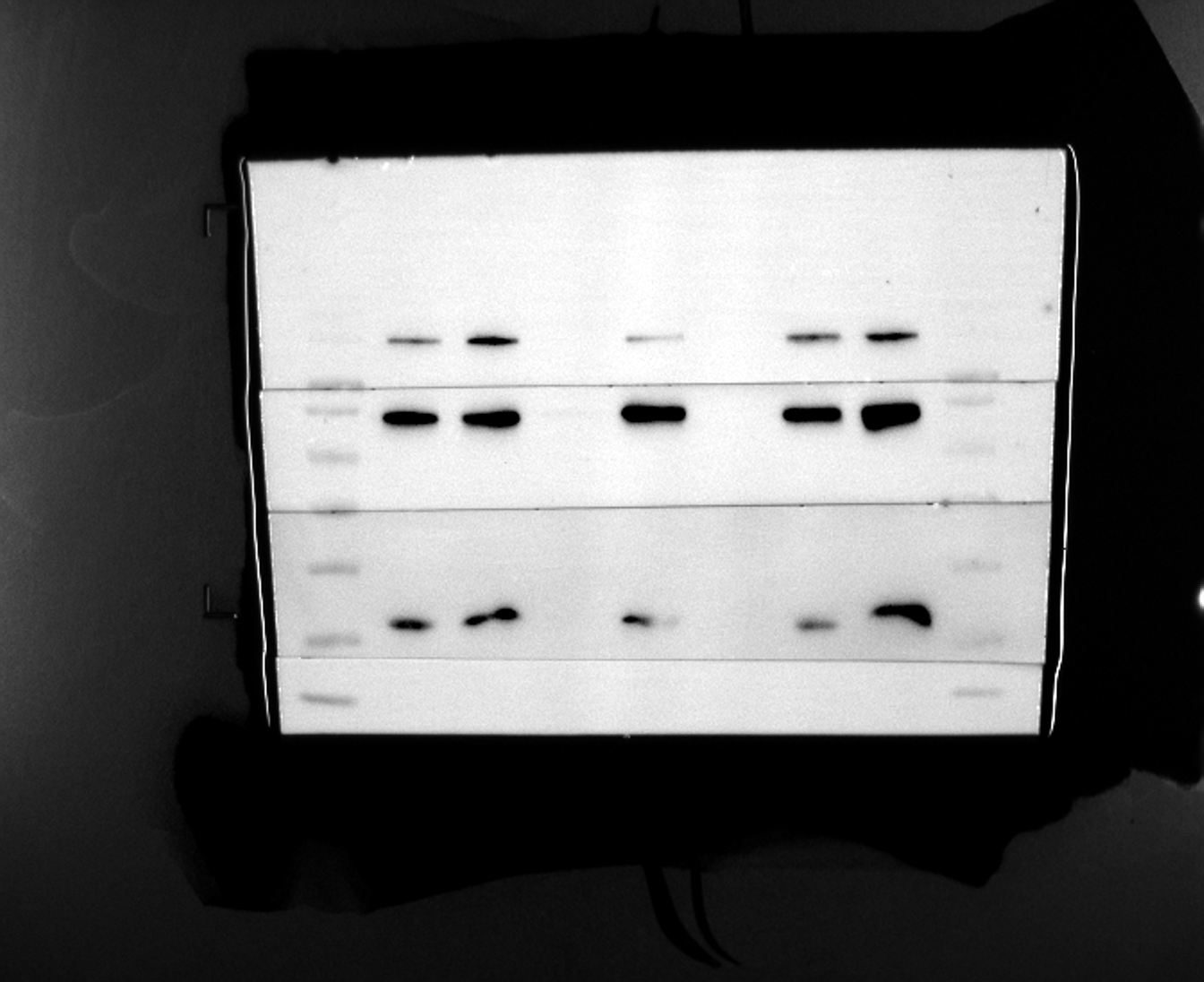

Supplement: Supplementary file 9 — Source data Fig. 2 [file 44319_2026_783_MOESM9_ESM.zip › Figure2/2F/SAM50-MIC26-MERGE.Tif]

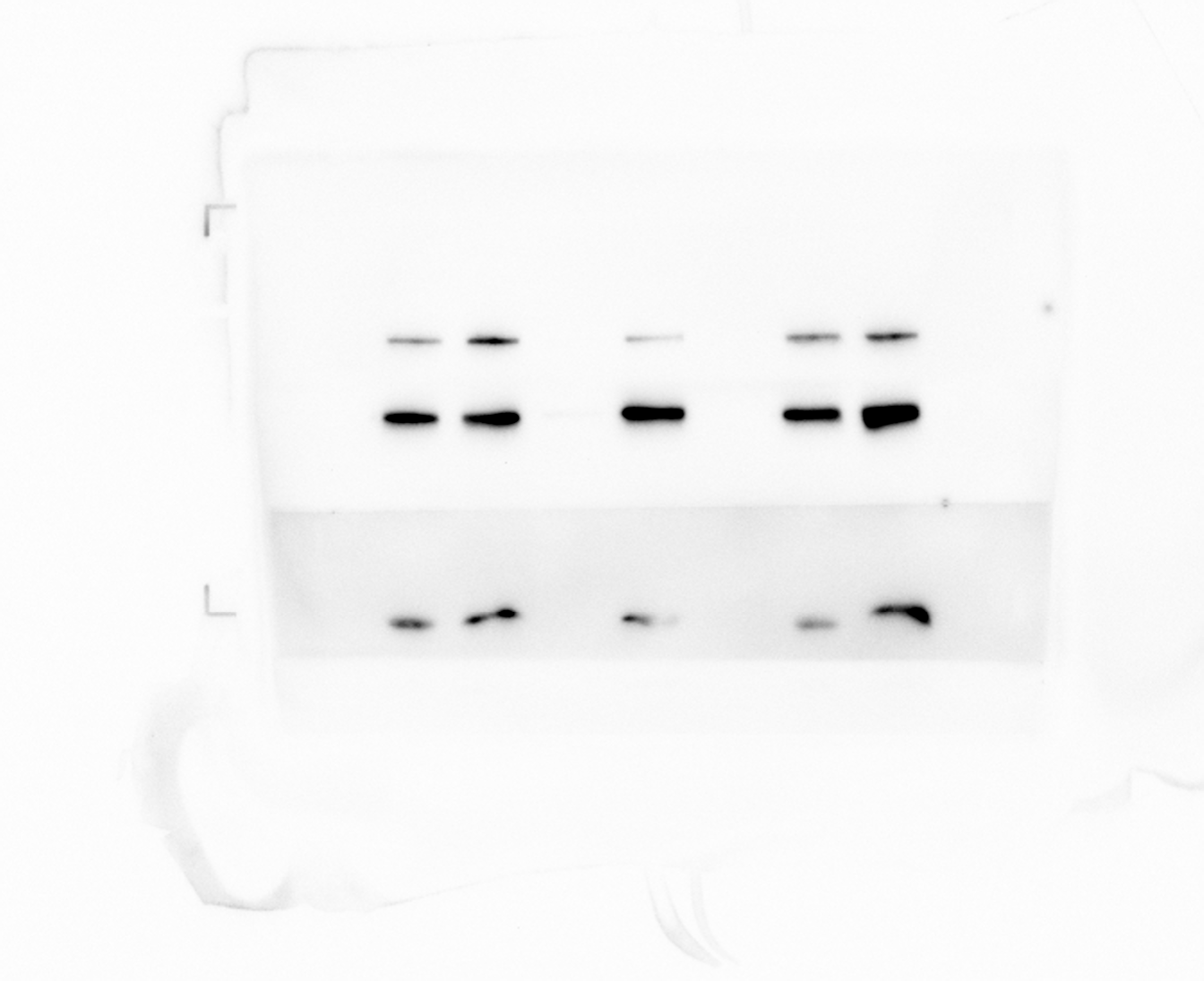

Supplement: Supplementary file 9 — Source data Fig. 2 [file 44319_2026_783_MOESM9_ESM.zip › Figure2/2F/SAM50-MIC26-RAW.tif]

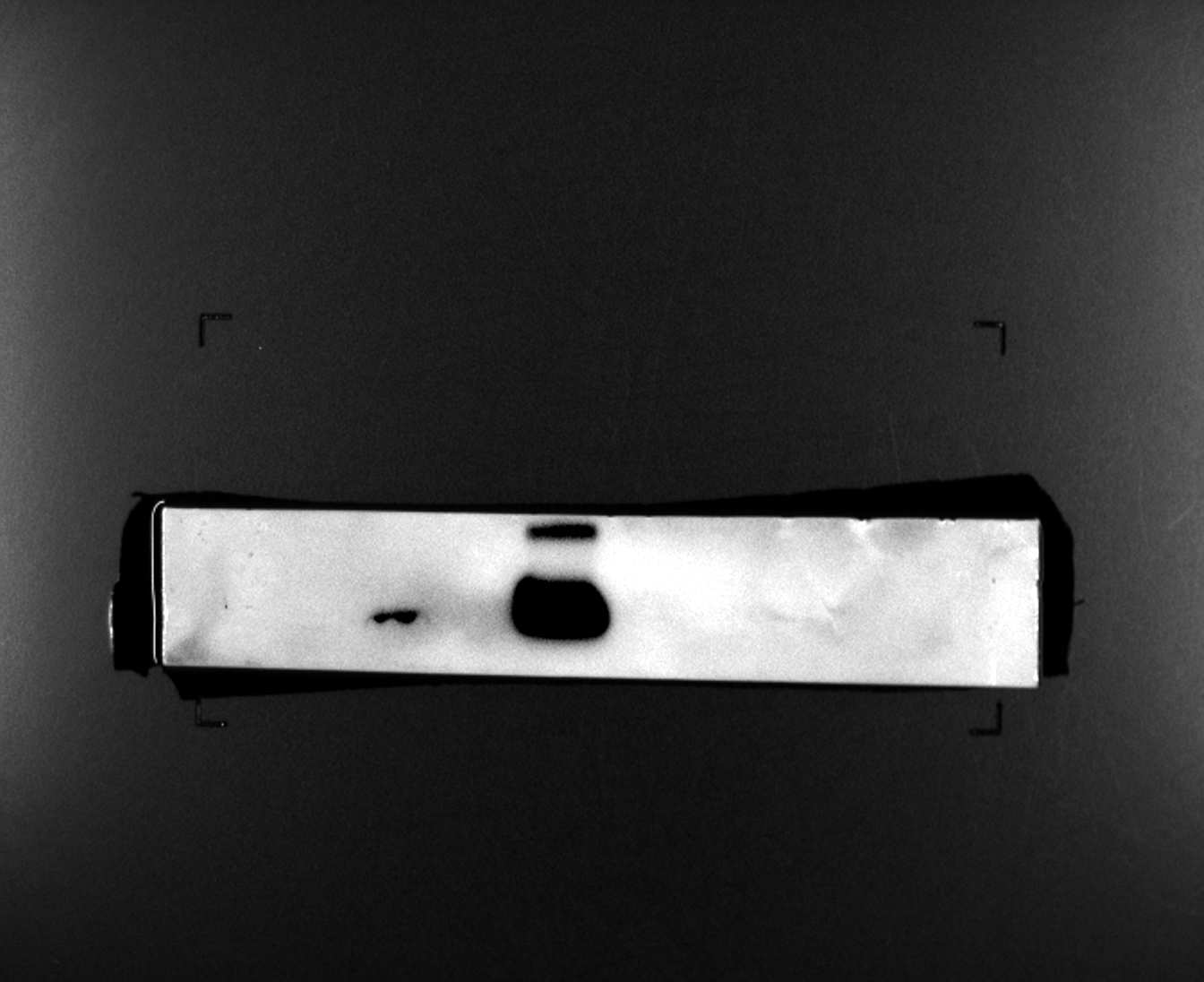

Supplement: Supplementary file 9 — Source data Fig. 2 [file 44319_2026_783_MOESM9_ESM.zip › Figure2/2F/StrepII-Merge.Tif]

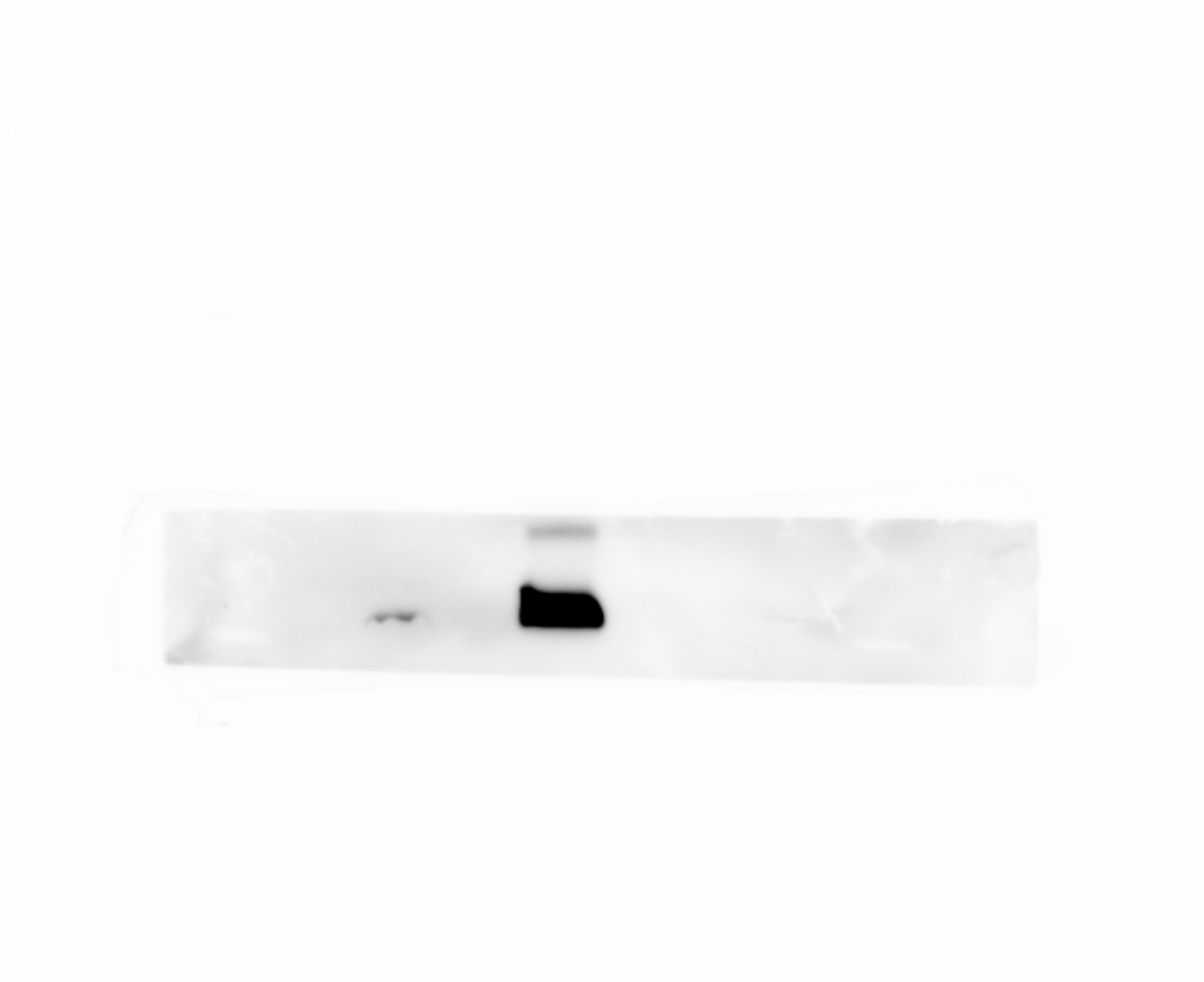

Supplement: Supplementary file 9 — Source data Fig. 2 [file 44319_2026_783_MOESM9_ESM.zip › Figure2/2F/StrepII-Raw.Tif]

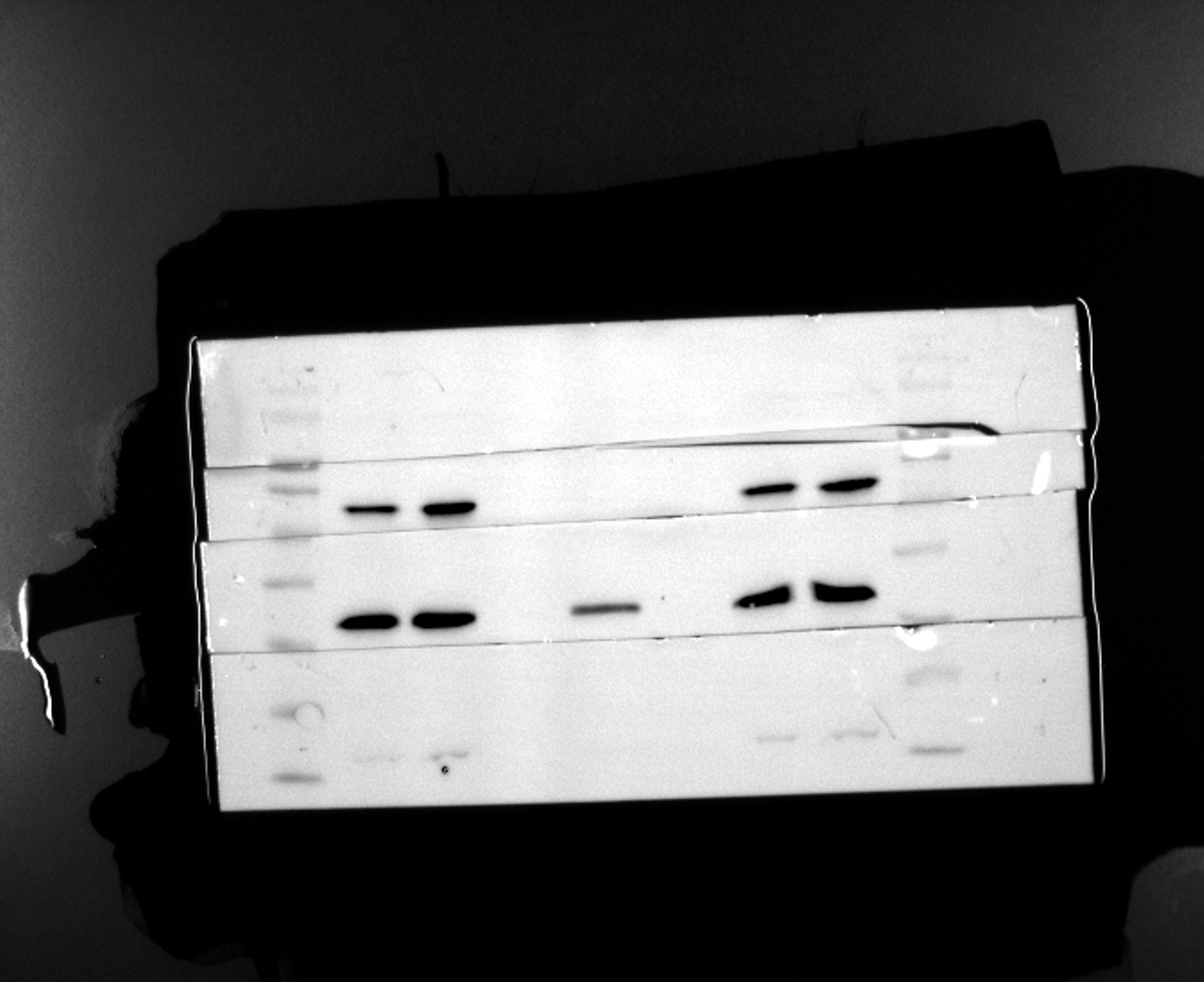

Supplement: Supplementary file 9 — Source data Fig. 2 [file 44319_2026_783_MOESM9_ESM.zip › Figure2/2F/UQCRC1-MIC19-MERGE.Tif]

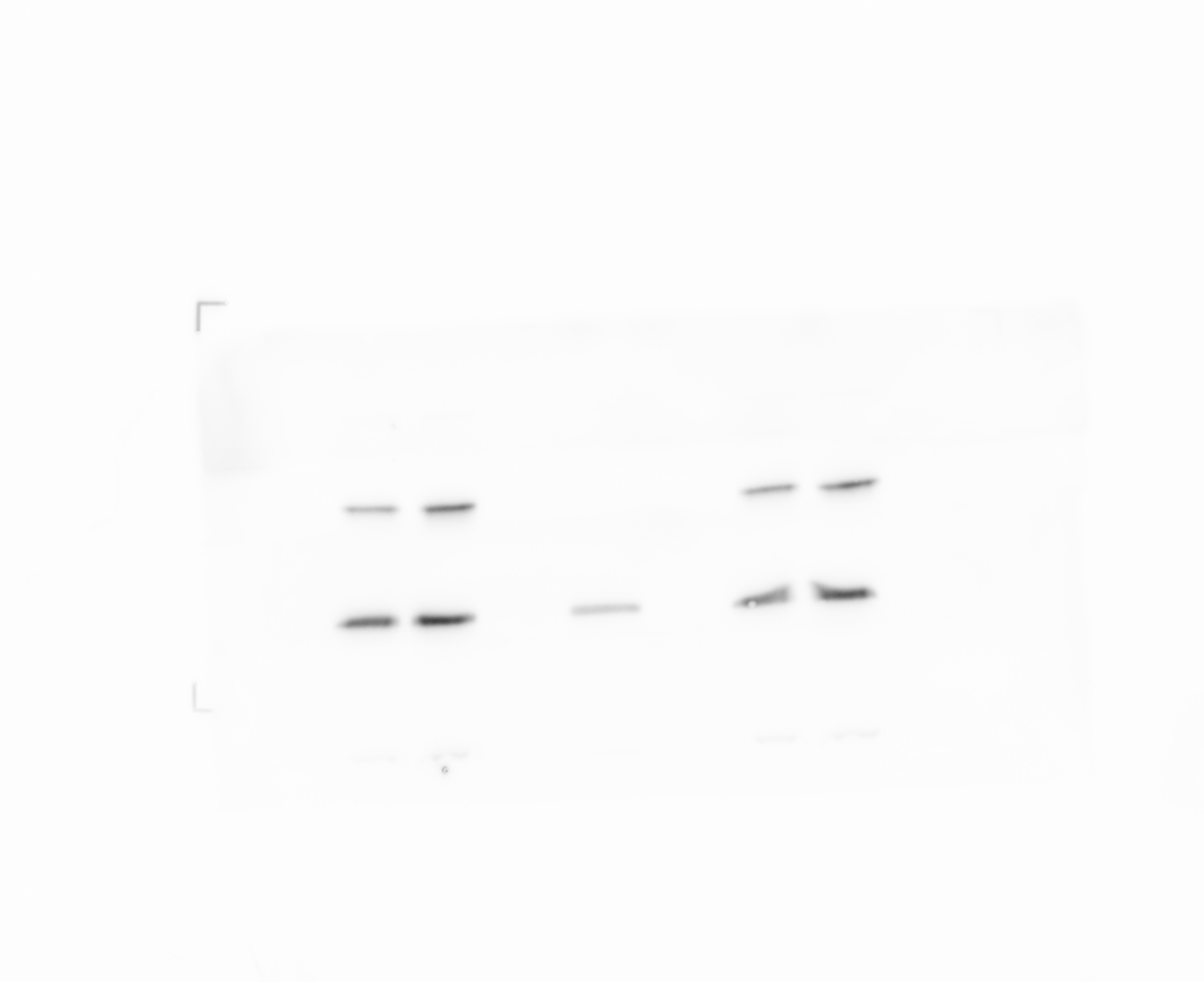

Supplement: Supplementary file 9 — Source data Fig. 2 [file 44319_2026_783_MOESM9_ESM.zip › Figure2/2F/UQCRC1-MIC19-RAW.Tif]

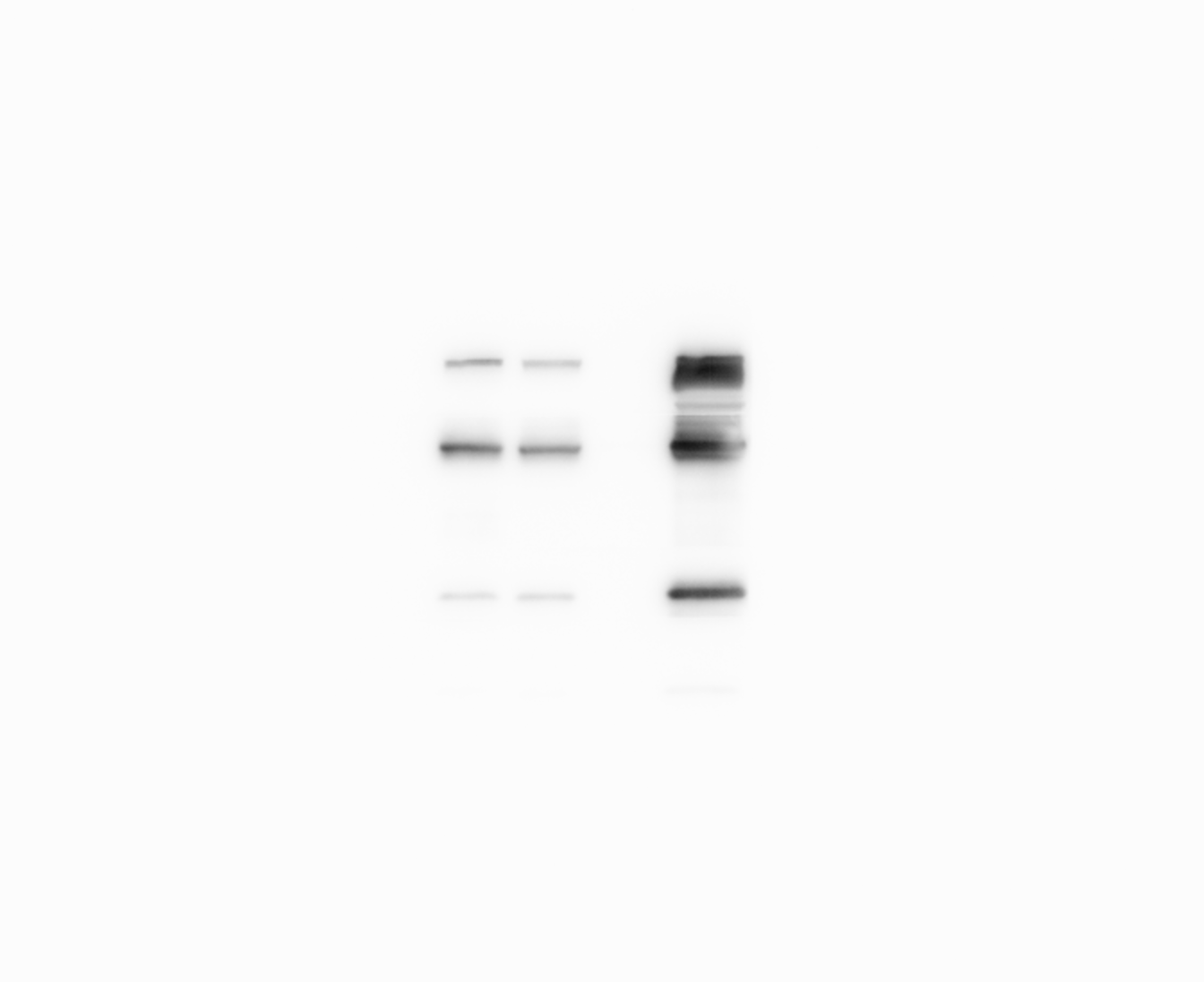

Supplement: Supplementary file 9 — Source data Fig. 2 [file 44319_2026_783_MOESM9_ESM.zip › Figure2/2G/Figure_2G- MIC60_SAM50_MIC19_Raw_data.Tif]

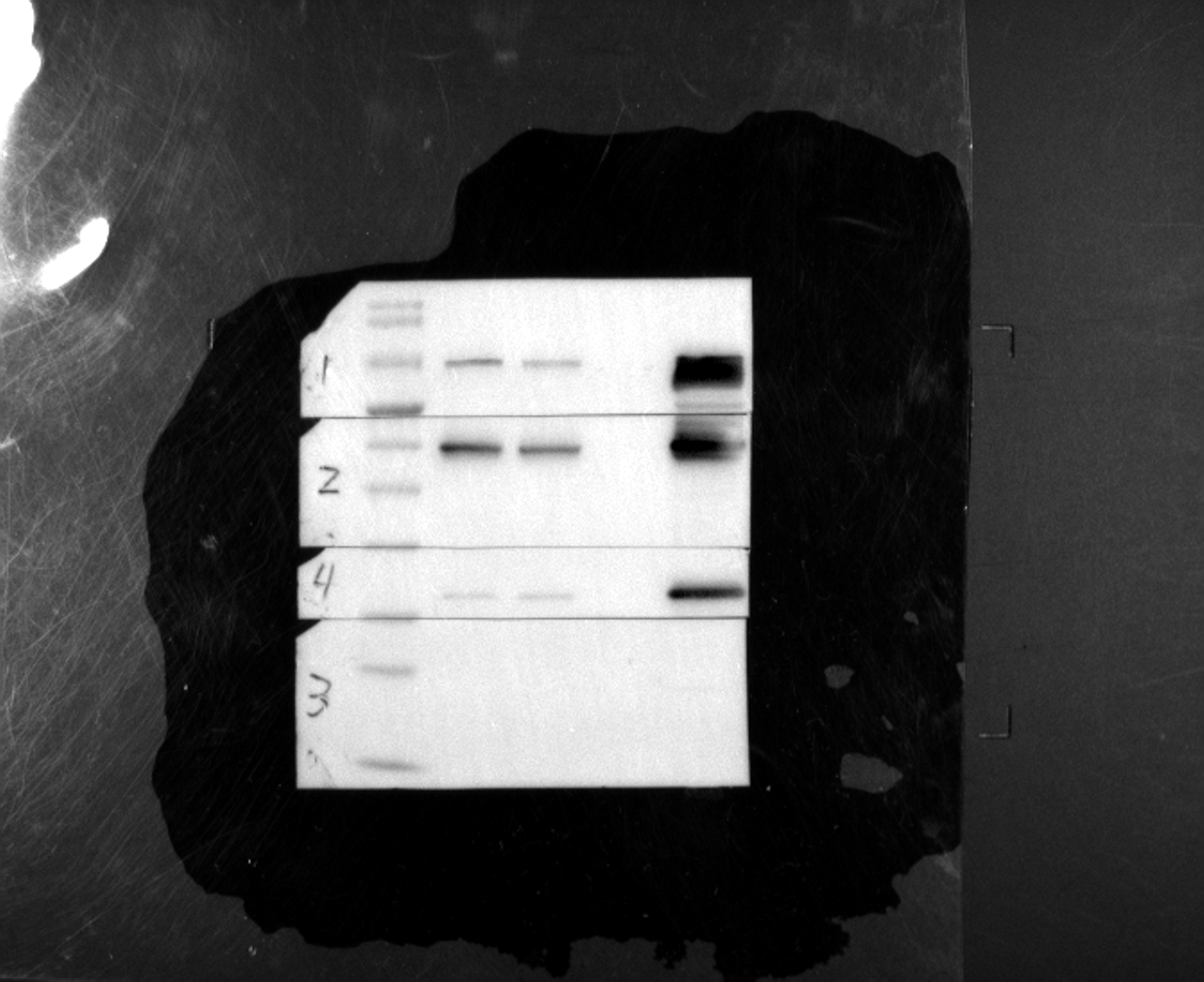

Supplement: Supplementary file 9 — Source data Fig. 2 [file 44319_2026_783_MOESM9_ESM.zip › Figure2/2G/Figure_2G-MIC60_SAM50_MIC19-Merge_data.Tif]

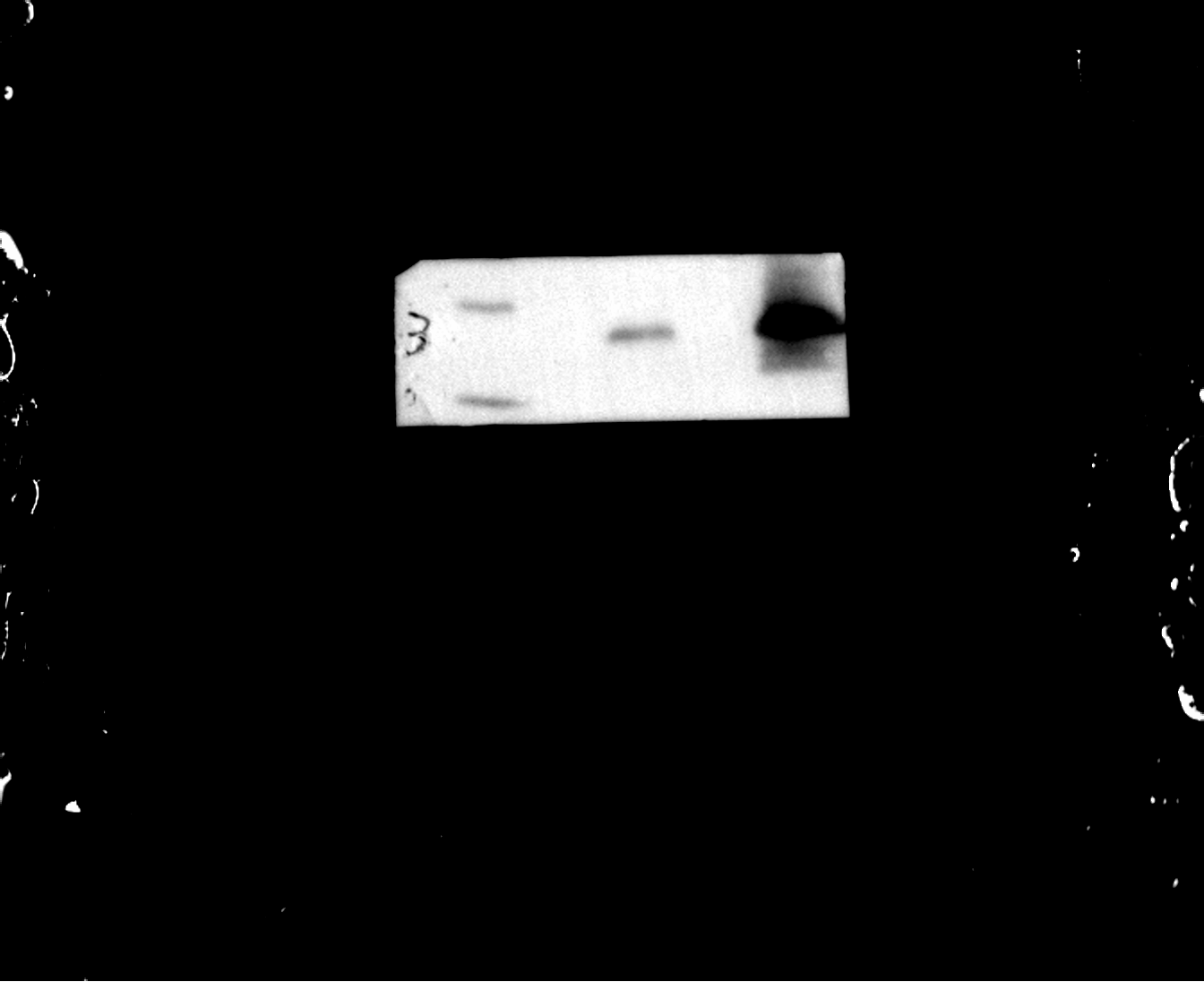

Supplement: Supplementary file 9 — Source data Fig. 2 [file 44319_2026_783_MOESM9_ESM.zip › Figure2/2G/Figure_2G-STREP-Merge_data.tif]

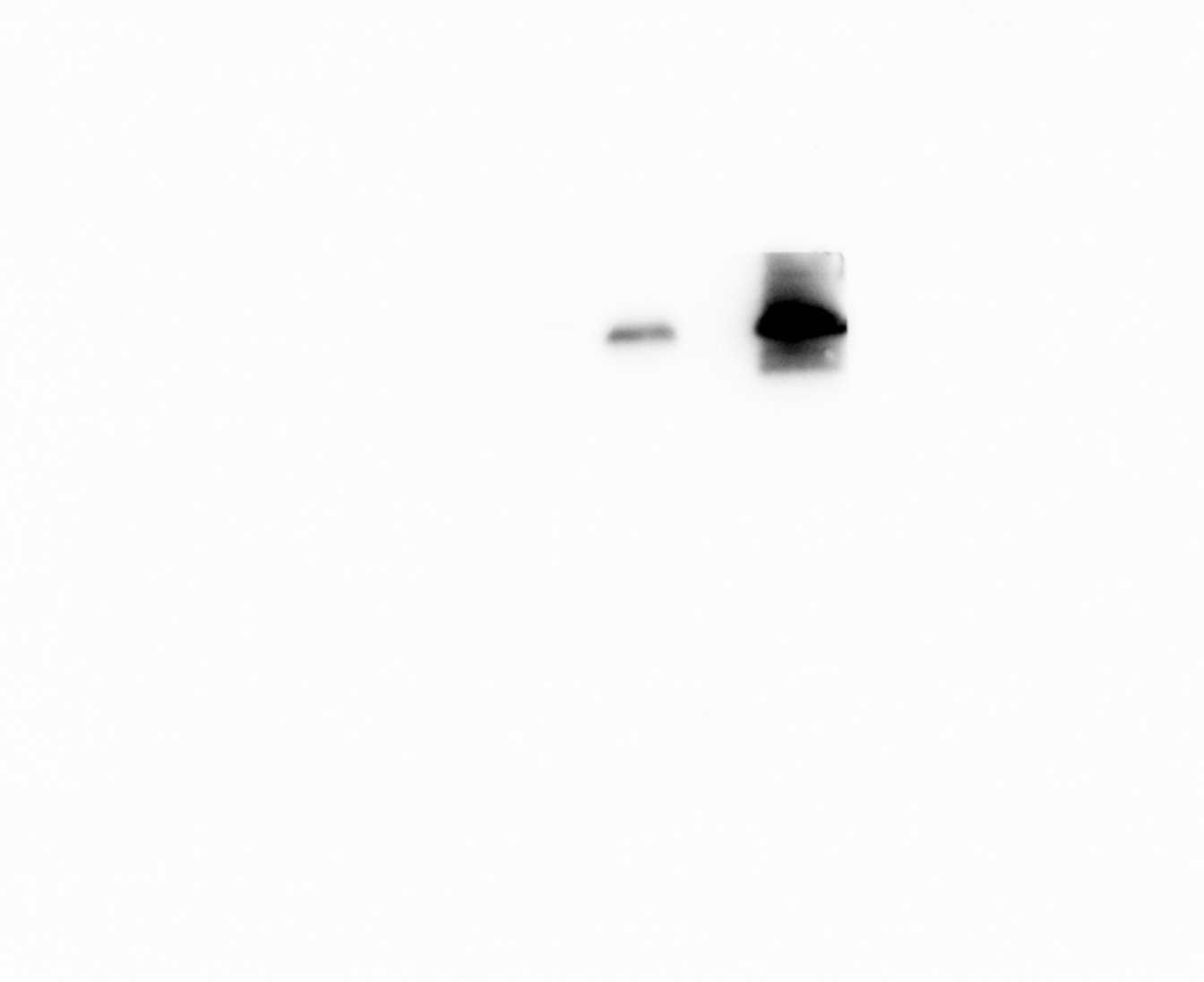

Supplement: Supplementary file 9 — Source data Fig. 2 [file 44319_2026_783_MOESM9_ESM.zip › Figure2/2G/Figure_2G-STREP-Raw_data.tif]

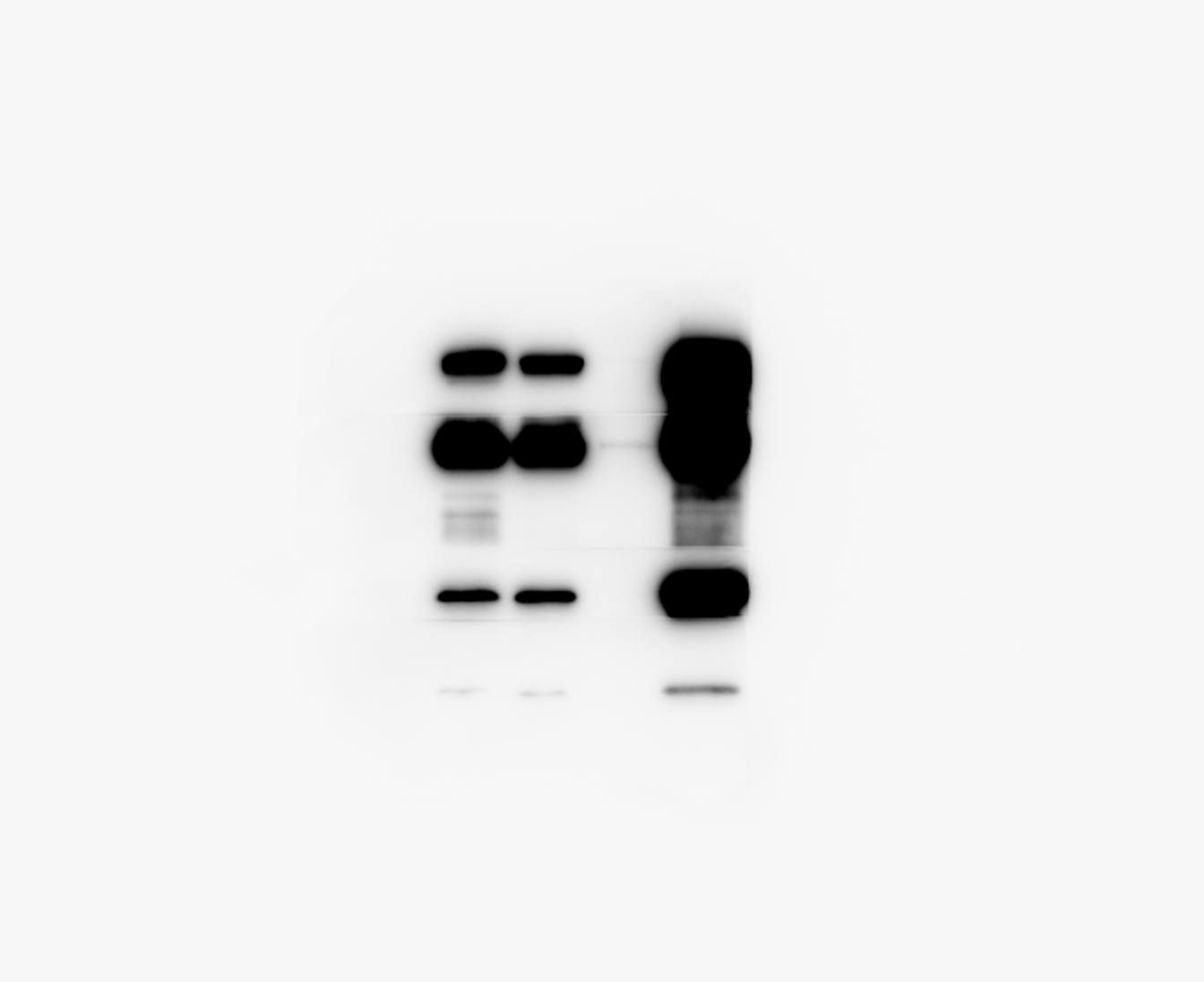

Supplement: Supplementary file 9 — Source data Fig. 2 [file 44319_2026_783_MOESM9_ESM.zip › Figure2/2G/Figure_2G-STREMI-Raw_data.Tif]

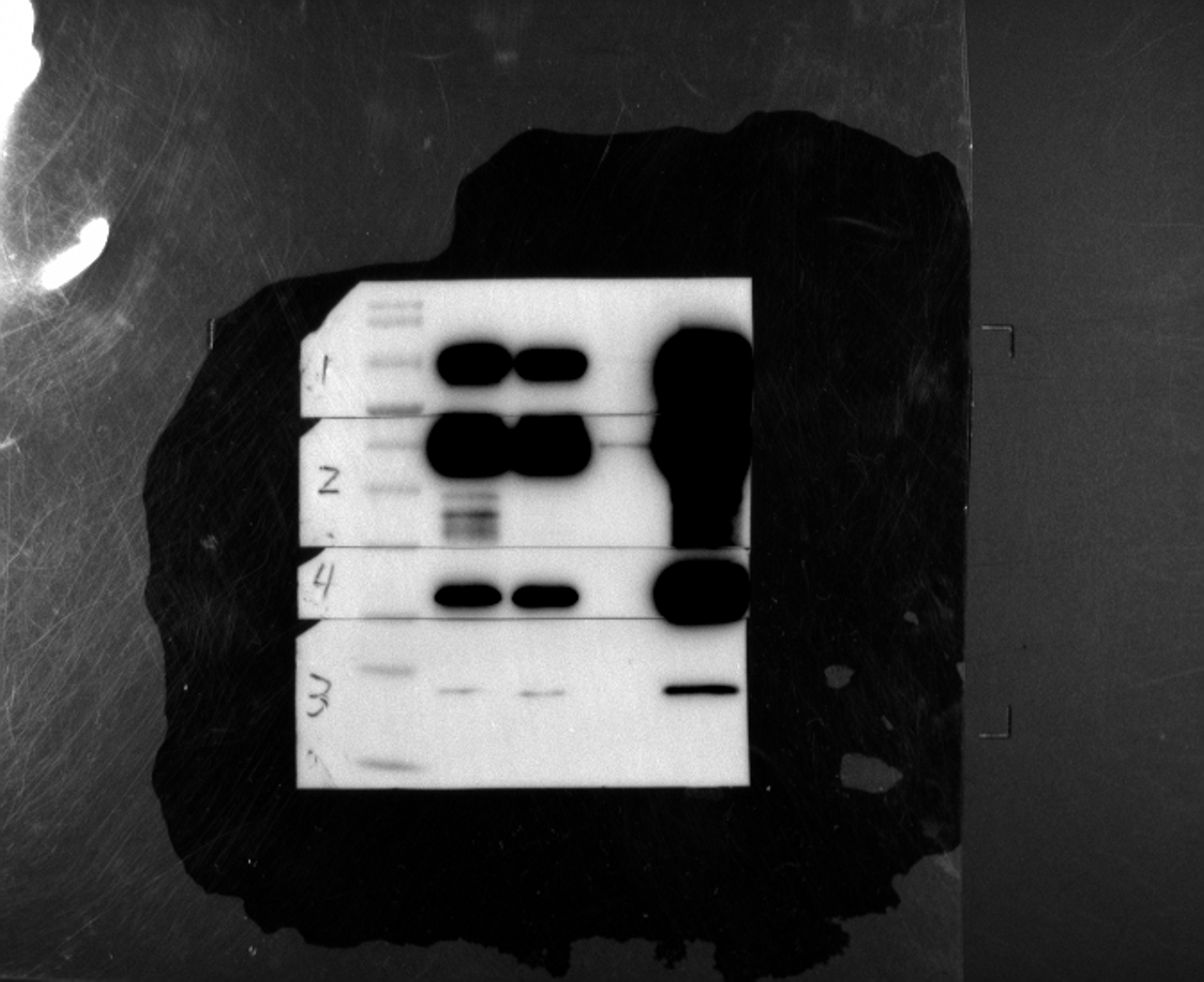

Supplement: Supplementary file 9 — Source data Fig. 2 [file 44319_2026_783_MOESM9_ESM.zip › Figure2/2G/Figure_2G-STREMI-Merge_data.Tif]

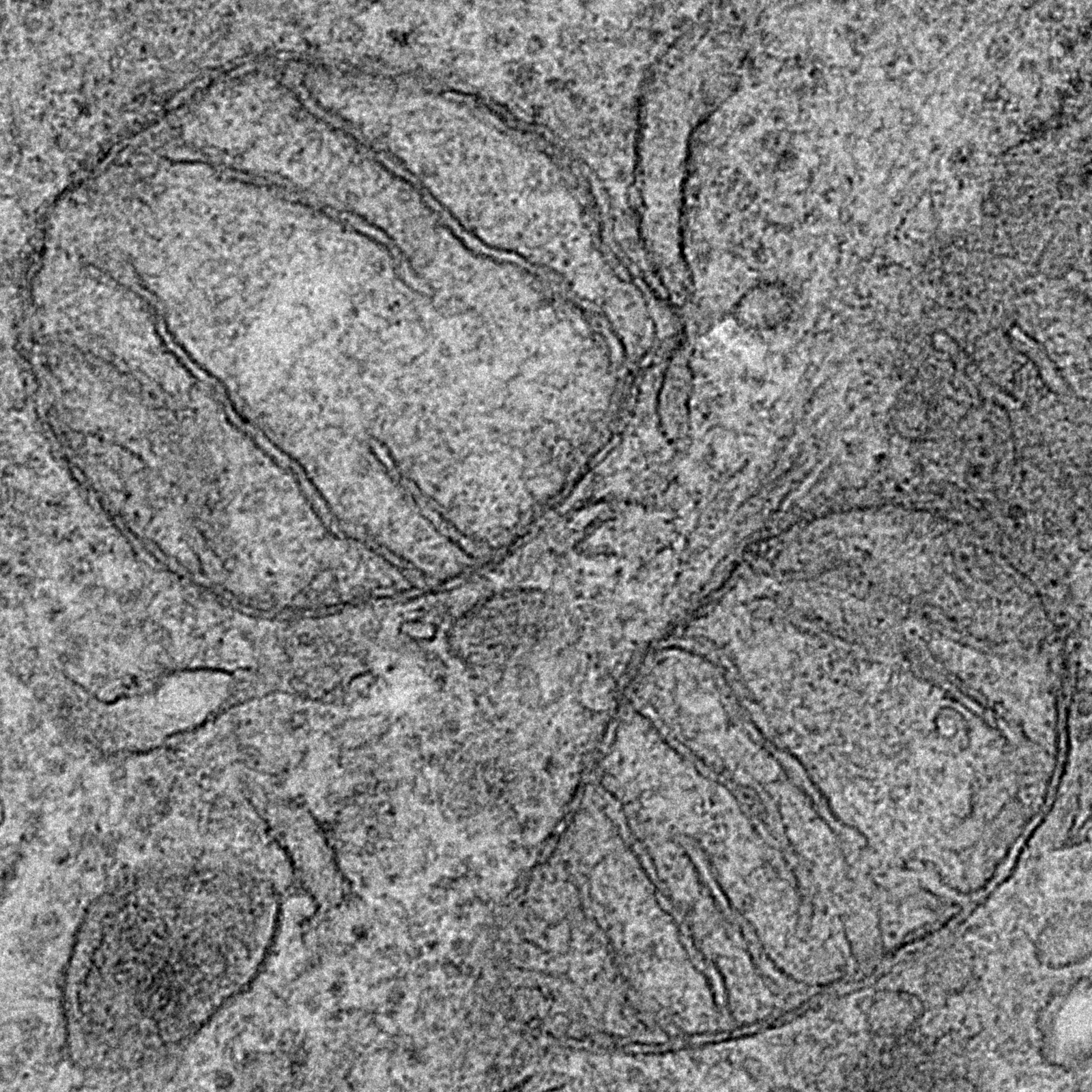

Supplement: Supplementary file 9 — Source data Fig. 2 [file 44319_2026_783_MOESM9_ESM.zip › Figure2/2J/SCR-TEM_data.tif]

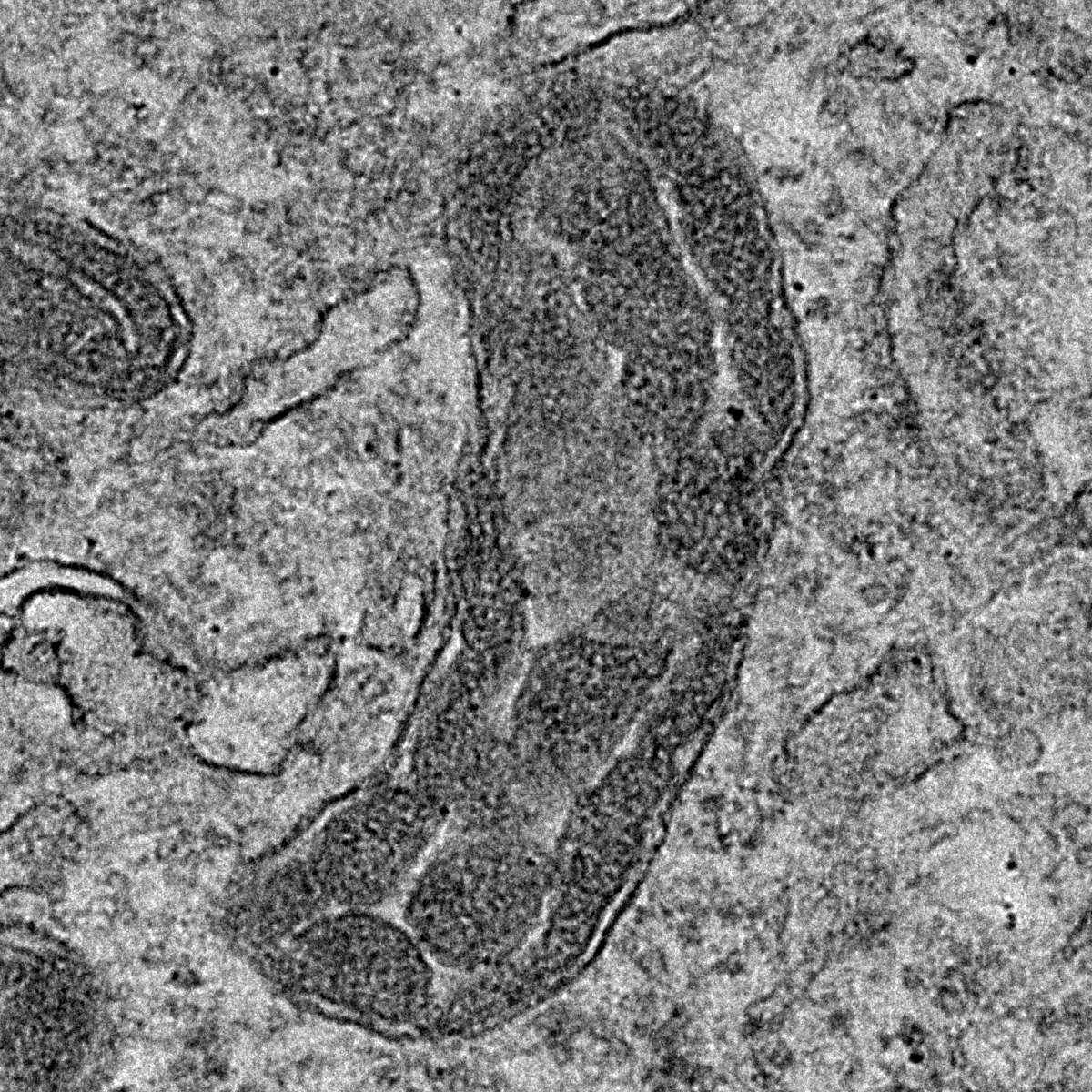

Supplement: Supplementary file 9 — Source data Fig. 2 [file 44319_2026_783_MOESM9_ESM.zip › Figure2/2K/STREMI_KO-Irregular_TEM_data.tif]

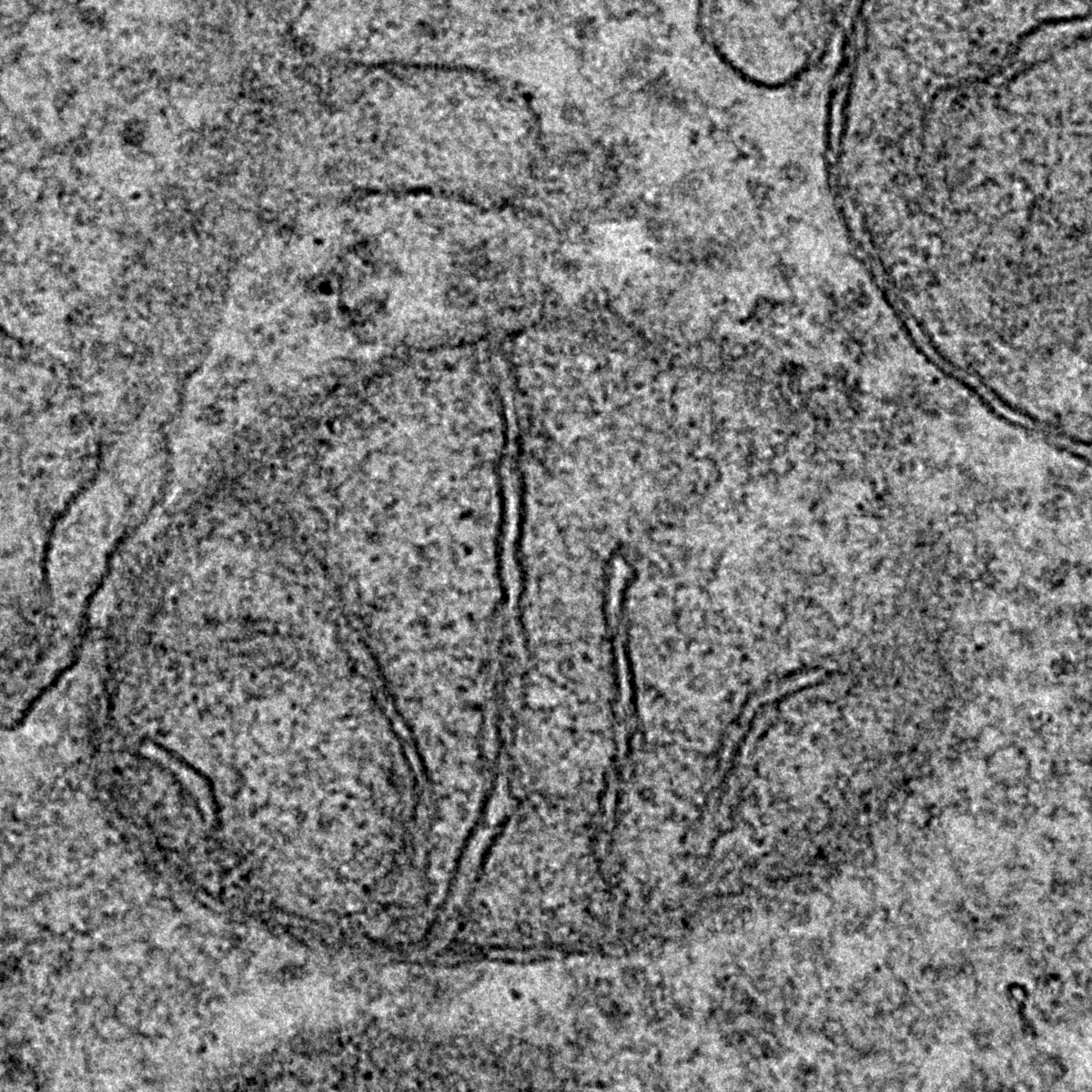

Supplement: Supplementary file 9 — Source data Fig. 2 [file 44319_2026_783_MOESM9_ESM.zip › Figure2/2K/STREMI_KO-Lamellar_TEM_data.tif]

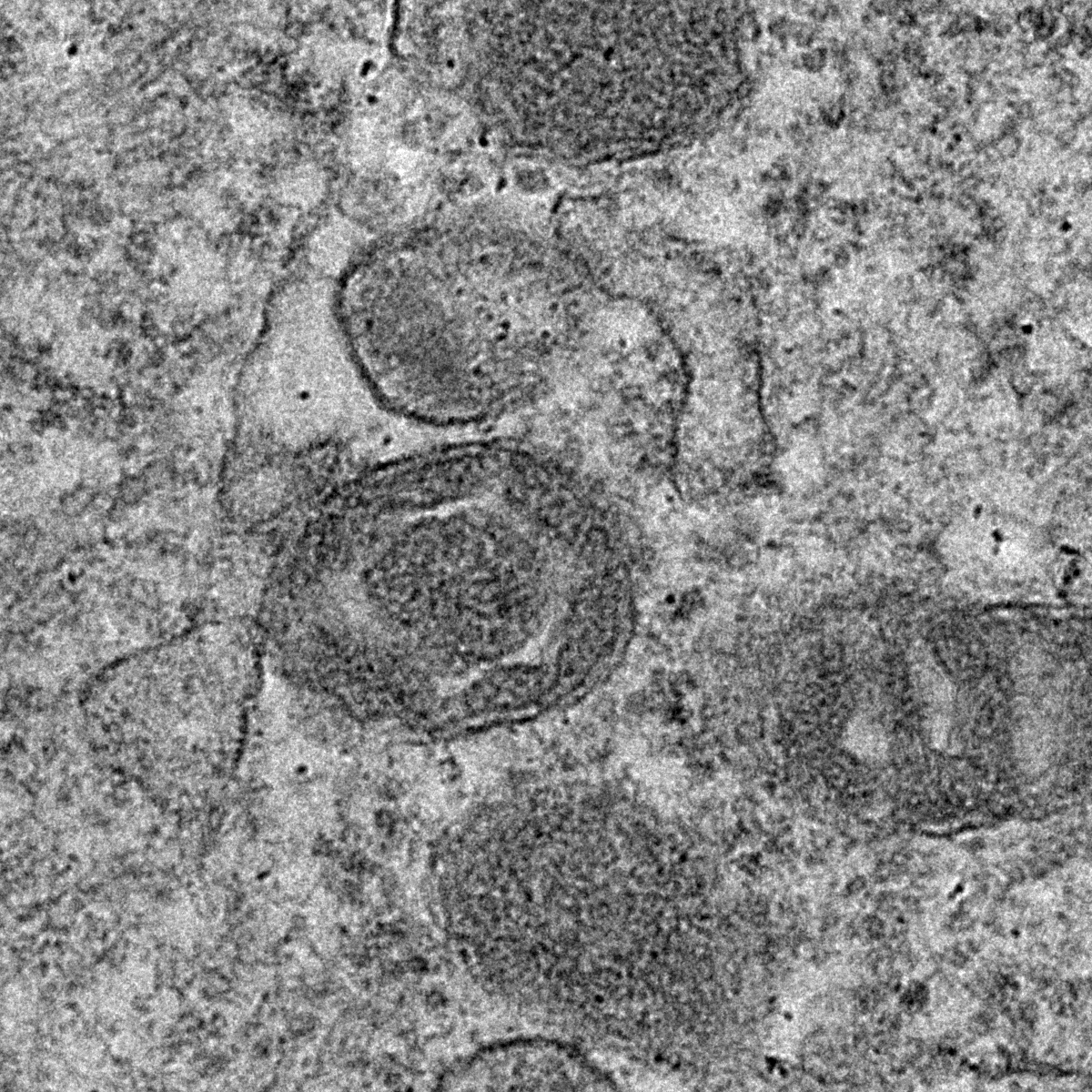

Supplement: Supplementary file 9 — Source data Fig. 2 [file 44319_2026_783_MOESM9_ESM.zip › Figure2/2K/STREMI_KO-Onion-shaped_TEM_data.tif]

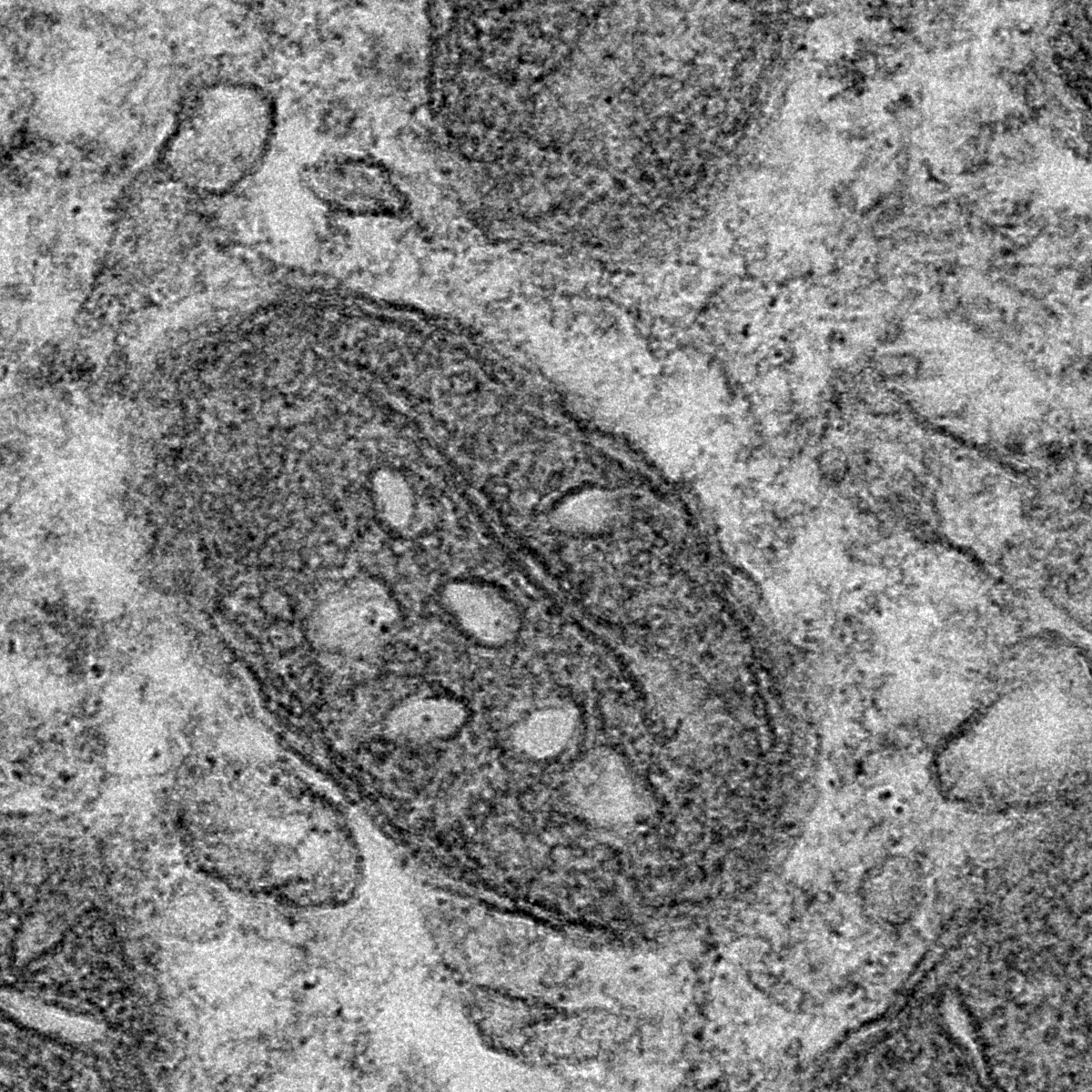

Supplement: Supplementary file 9 — Source data Fig. 2 [file 44319_2026_783_MOESM9_ESM.zip › Figure2/2K/STREMI_KO-Swollen_TEM_data.tif]

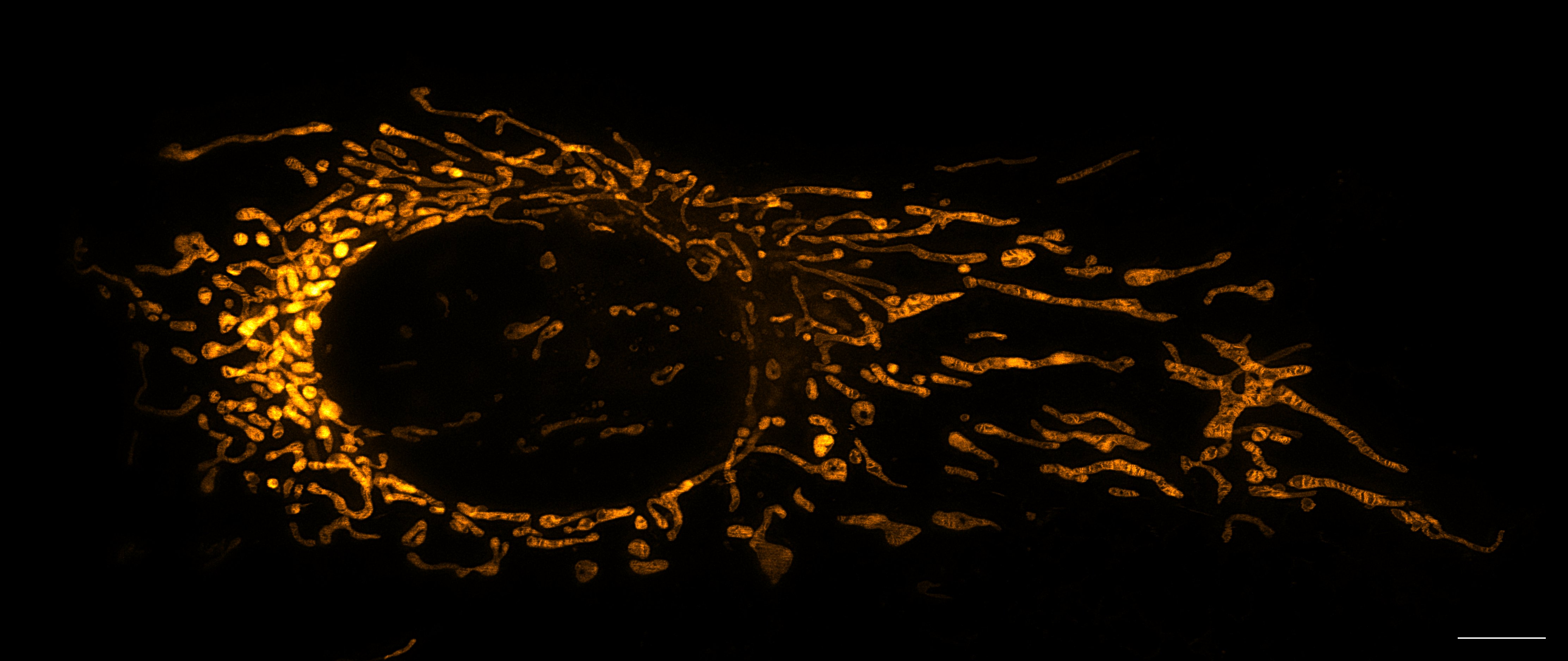

Supplement: Supplementary file 9 — Source data Fig. 2 [file 44319_2026_783_MOESM9_ESM.zip › Figure2/2N/HeLa_SCR_5um_scale bar.jpg]

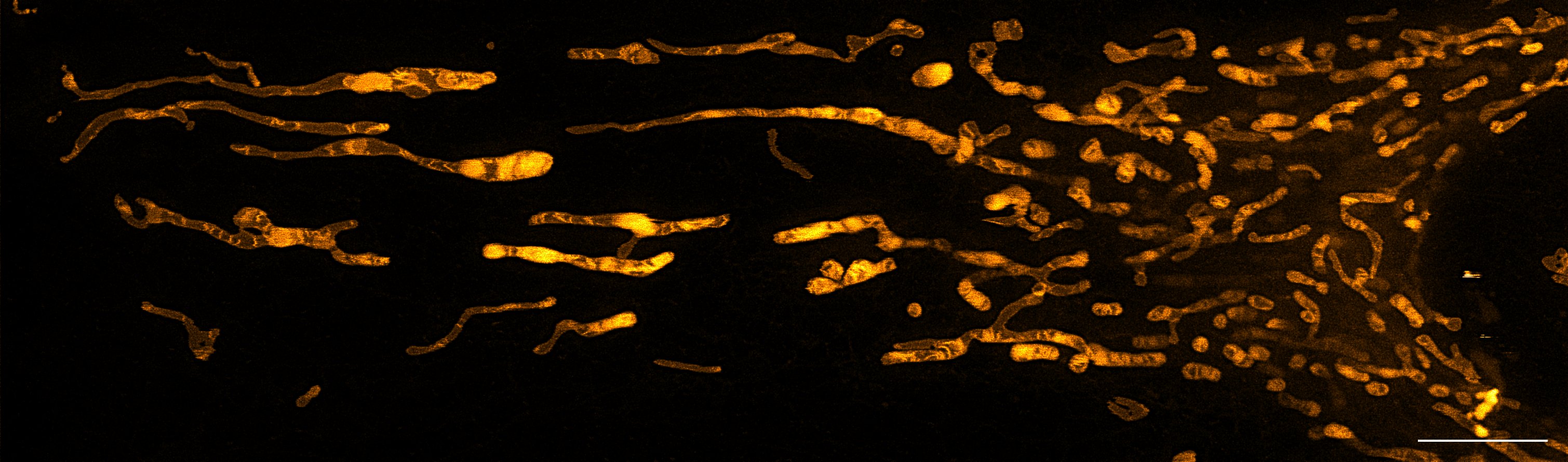

Supplement: Supplementary file 9 — Source data Fig. 2 [file 44319_2026_783_MOESM9_ESM.zip › Figure2/2N/HeLa_STREMI KO_5um_scale bar.jpg]

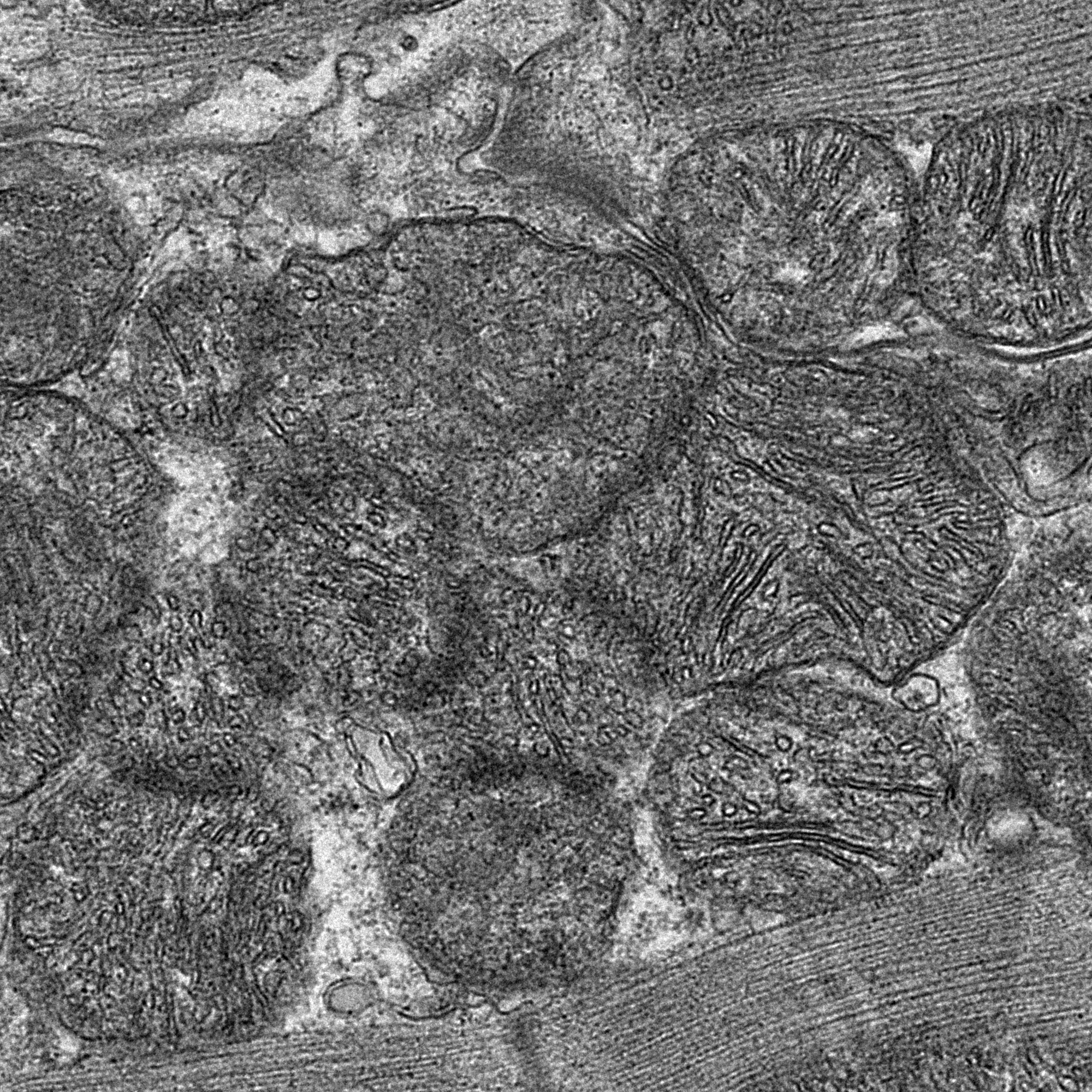

Supplement: Supplementary file 10 — Source data Fig. 3 [file 44319_2026_783_MOESM10_ESM.zip › Figure3/3B/STREMI_KO_TEM_data.tif]

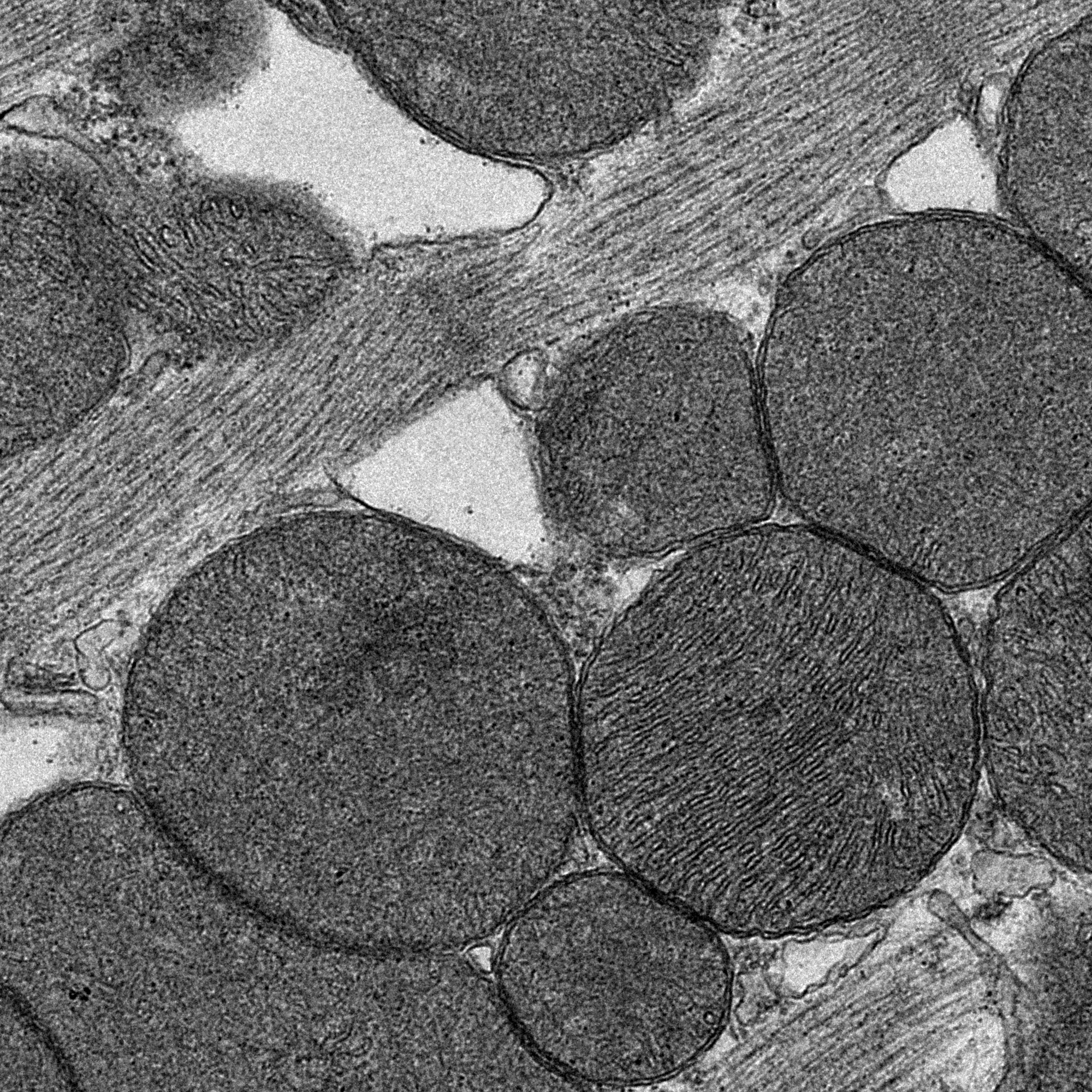

Supplement: Supplementary file 10 — Source data Fig. 3 [file 44319_2026_783_MOESM10_ESM.zip › Figure3/3B/WT_TEM_data.tif]

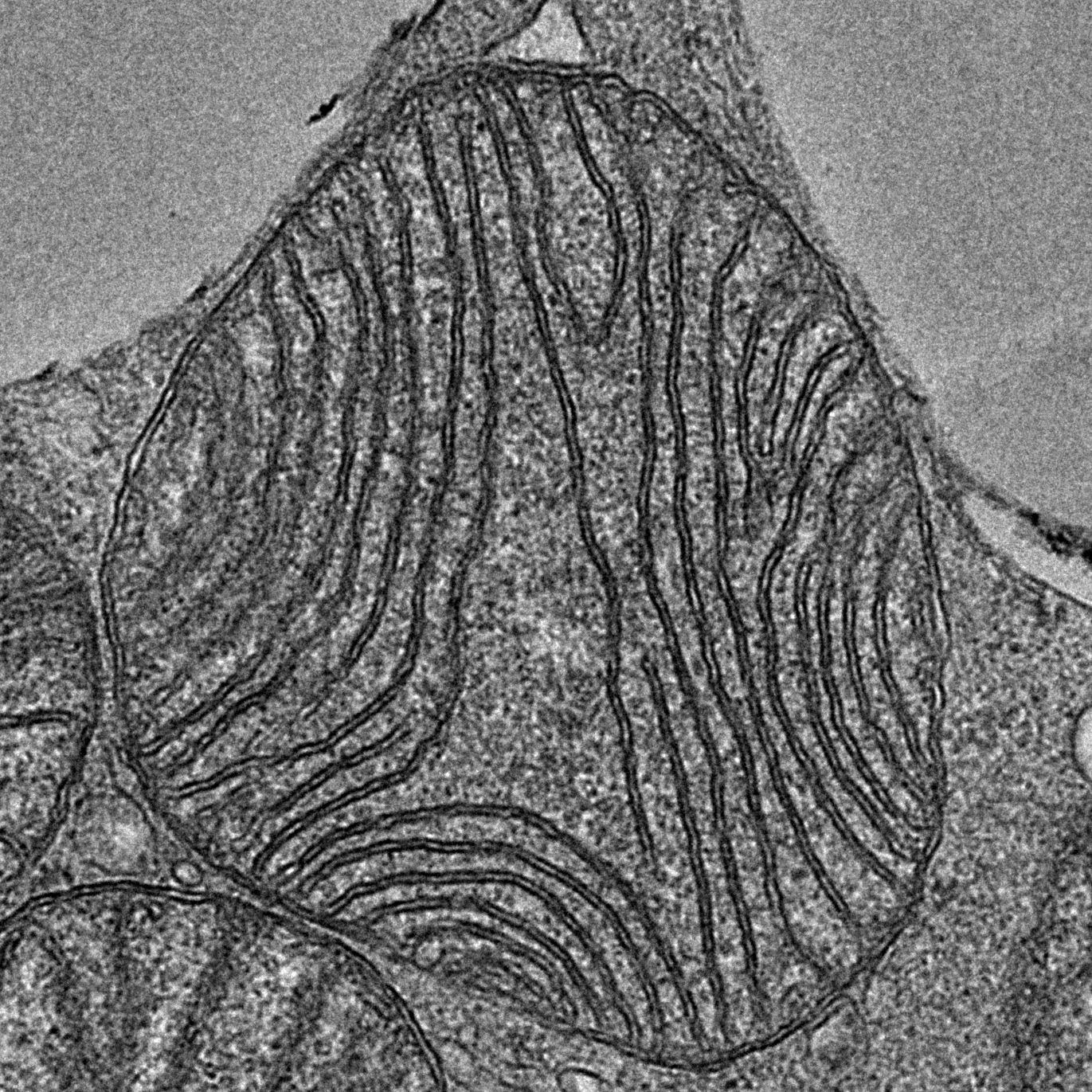

Supplement: Supplementary file 10 — Source data Fig. 3 [file 44319_2026_783_MOESM10_ESM.zip › Figure3/3D/WT_TEM_data-1.tif]

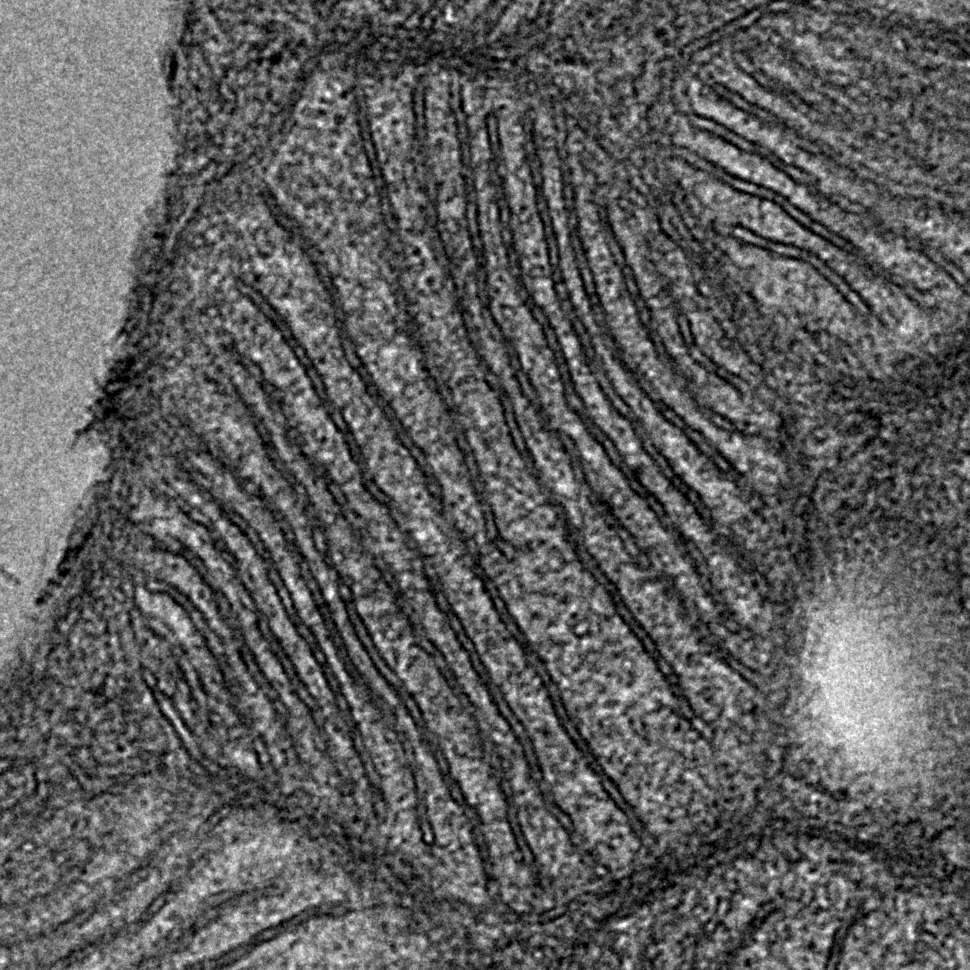

Supplement: Supplementary file 10 — Source data Fig. 3 [file 44319_2026_783_MOESM10_ESM.zip › Figure3/3D/WT_TEM_data-2.tif]

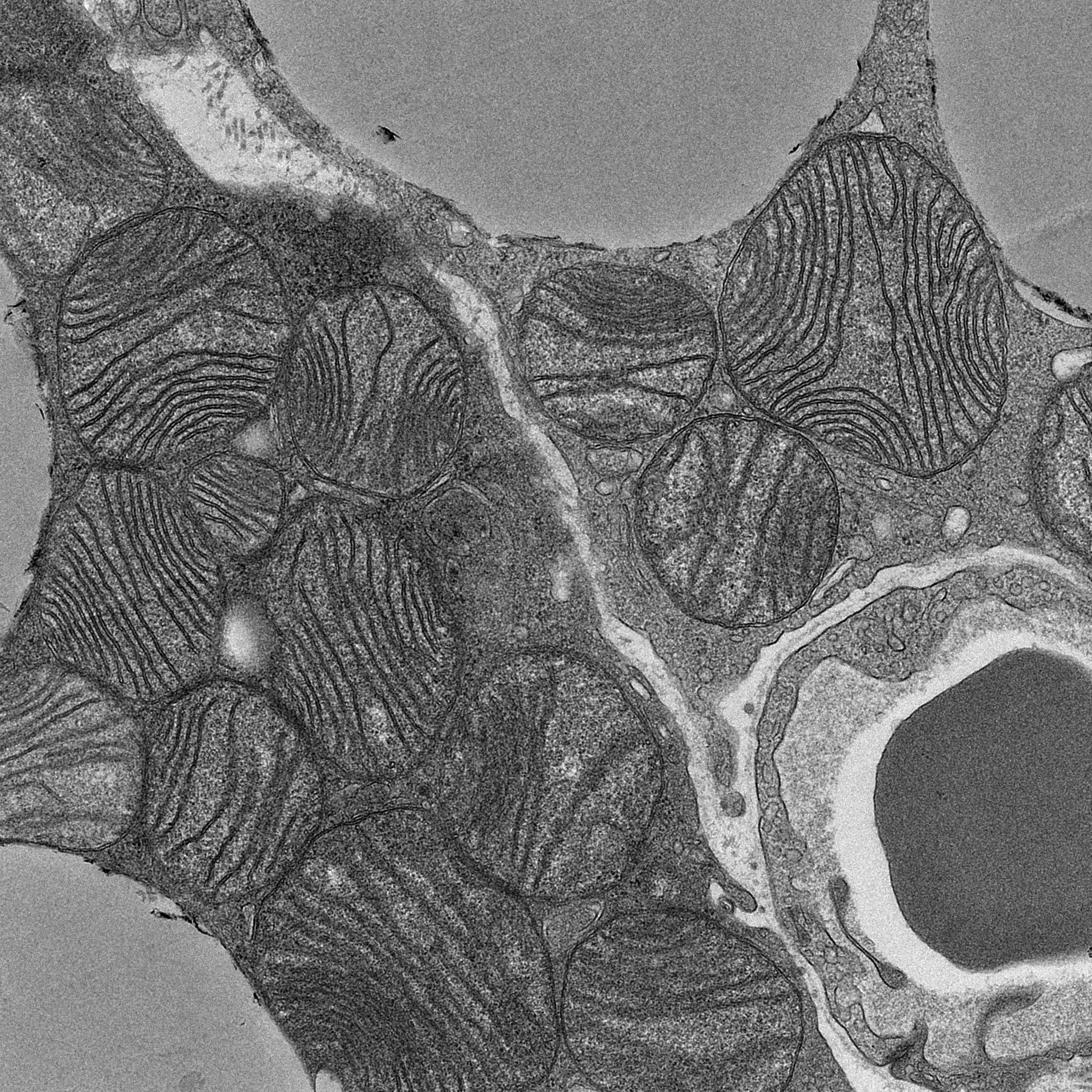

Supplement: Supplementary file 10 — Source data Fig. 3 [file 44319_2026_783_MOESM10_ESM.zip › Figure3/3D/WT_TEM_data.tif]

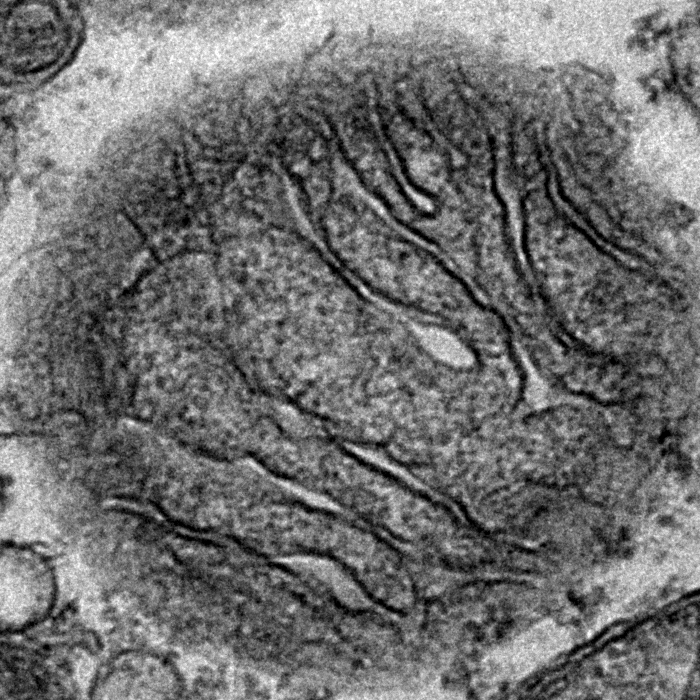

Supplement: Supplementary file 10 — Source data Fig. 3 [file 44319_2026_783_MOESM10_ESM.zip › Figure3/3E/STREMI_KO_TEM_data-1.tif]

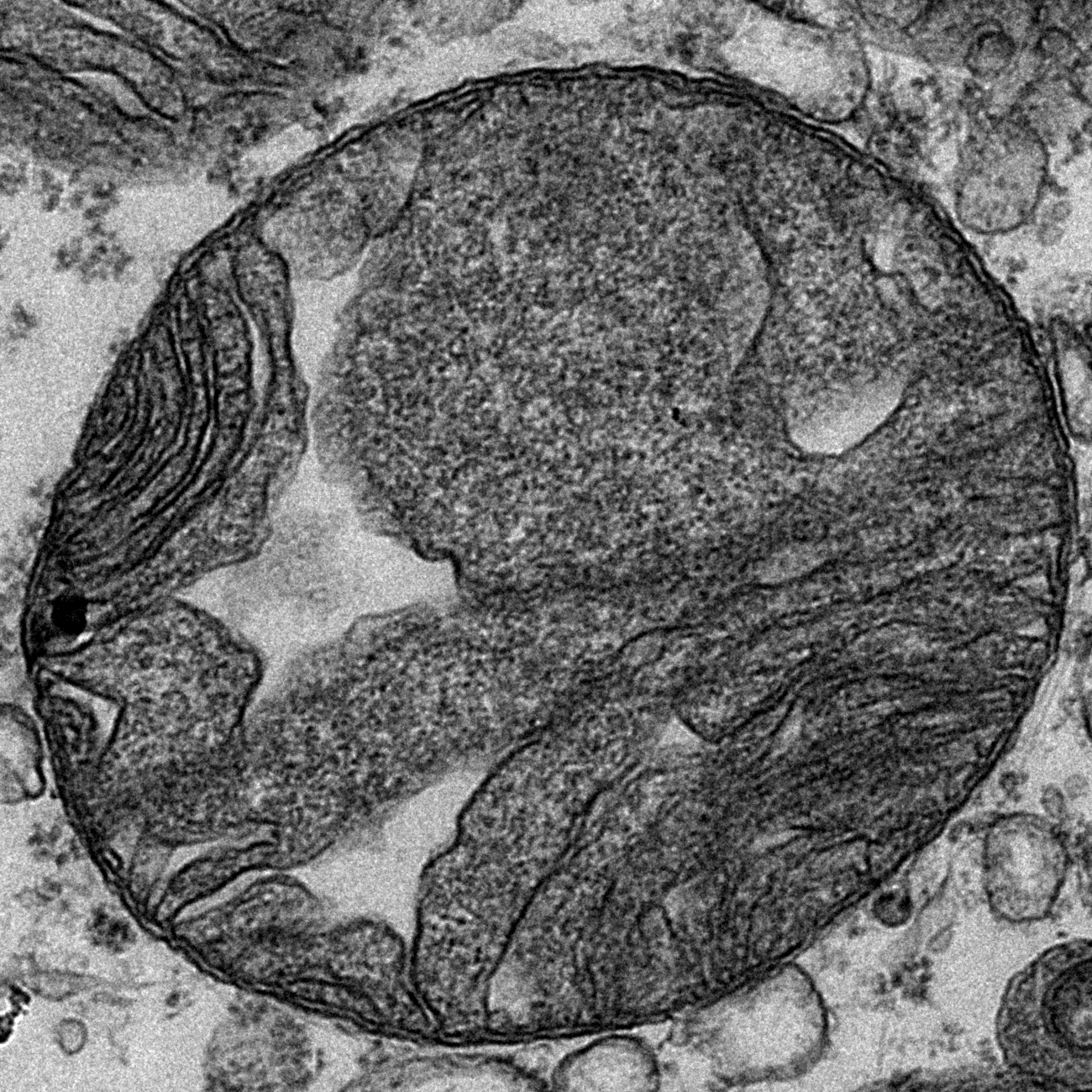

Supplement: Supplementary file 10 — Source data Fig. 3 [file 44319_2026_783_MOESM10_ESM.zip › Figure3/3E/STREMI_KO_TEM_data-2.tif]

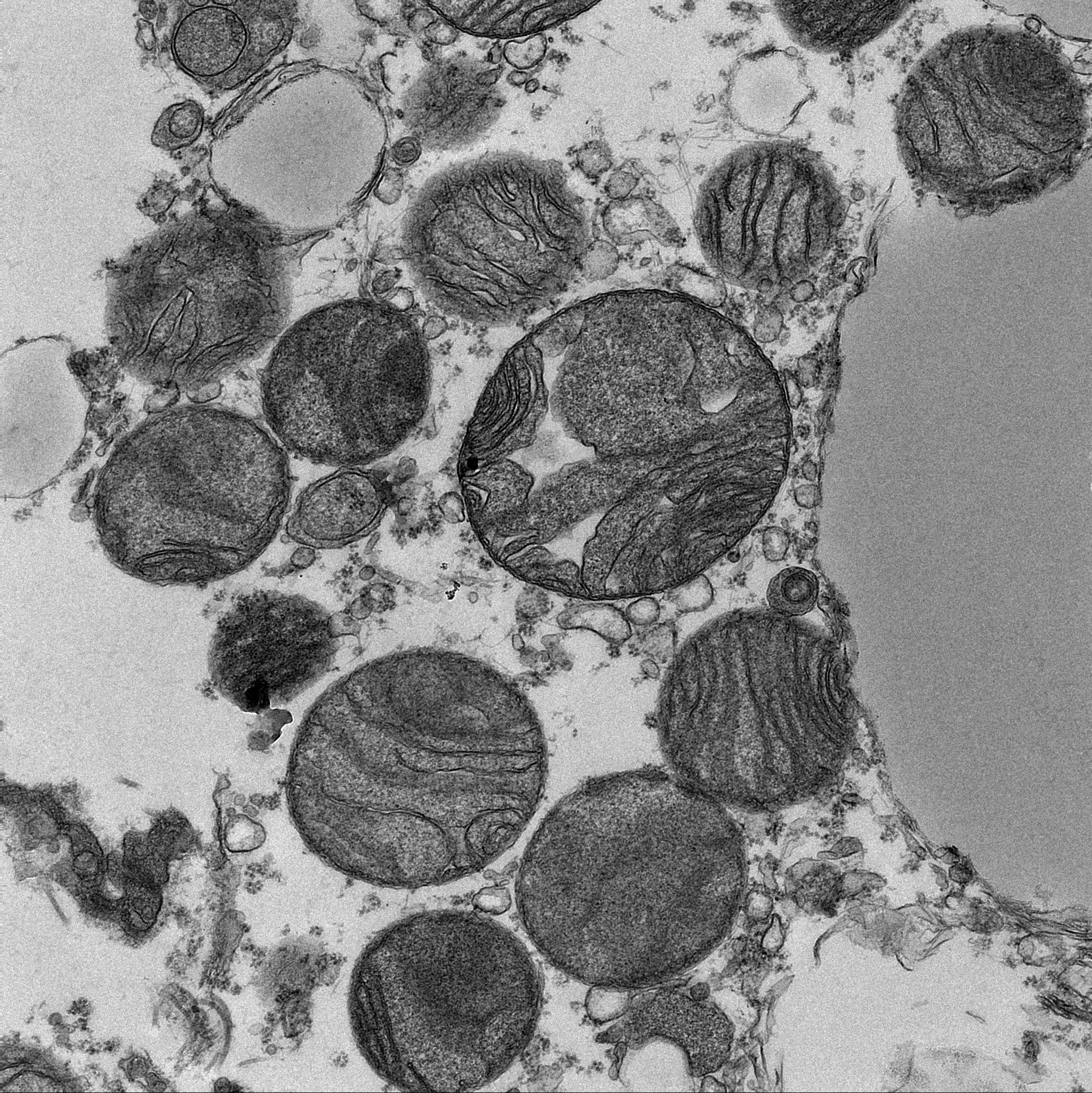

Supplement: Supplementary file 10 — Source data Fig. 3 [file 44319_2026_783_MOESM10_ESM.zip › Figure3/3E/STREMI_KO_TEM_data.tif]

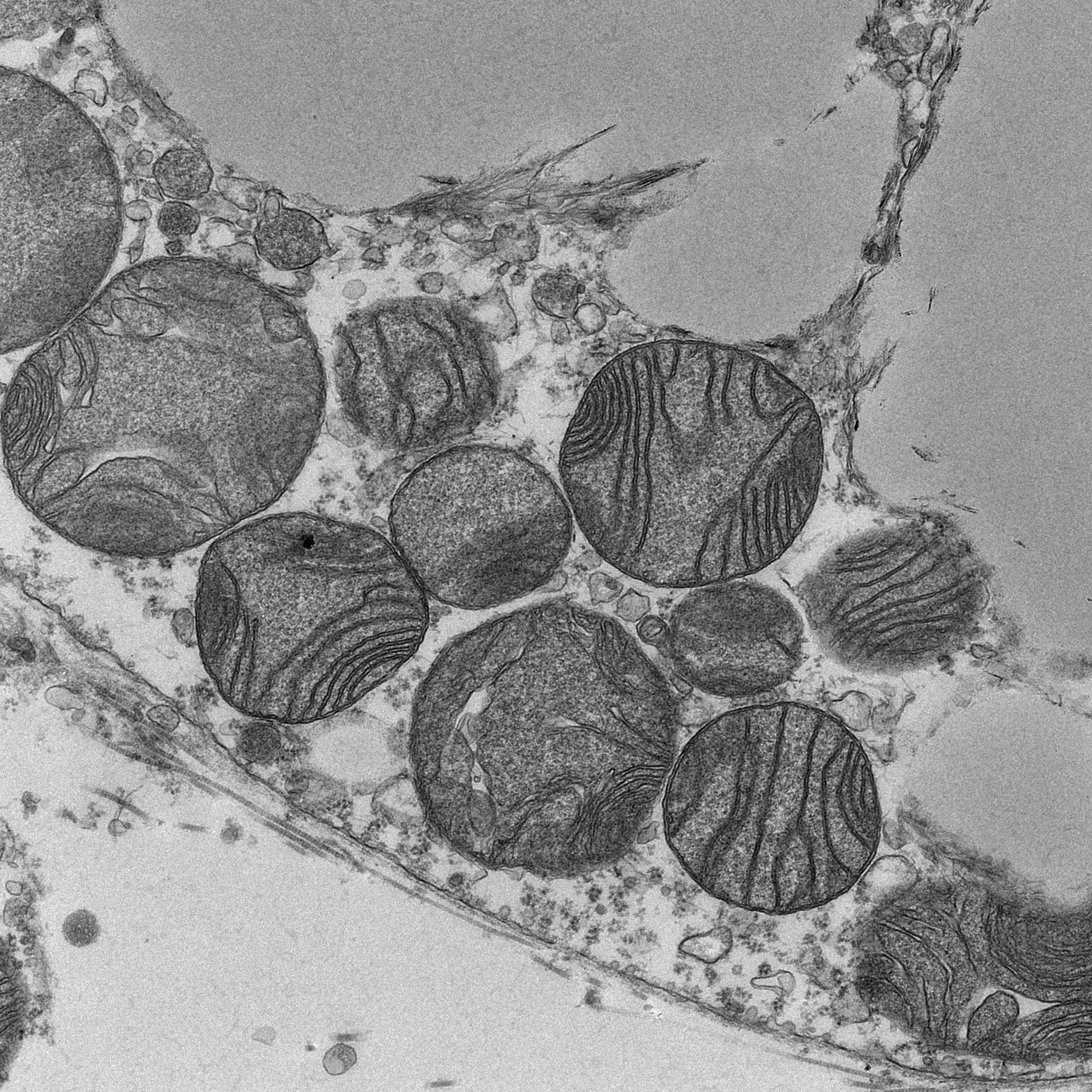

Supplement: Supplementary file 10 — Source data Fig. 3 [file 44319_2026_783_MOESM10_ESM.zip › Figure3/3H/STREMI_KO_TEM_data.tif]

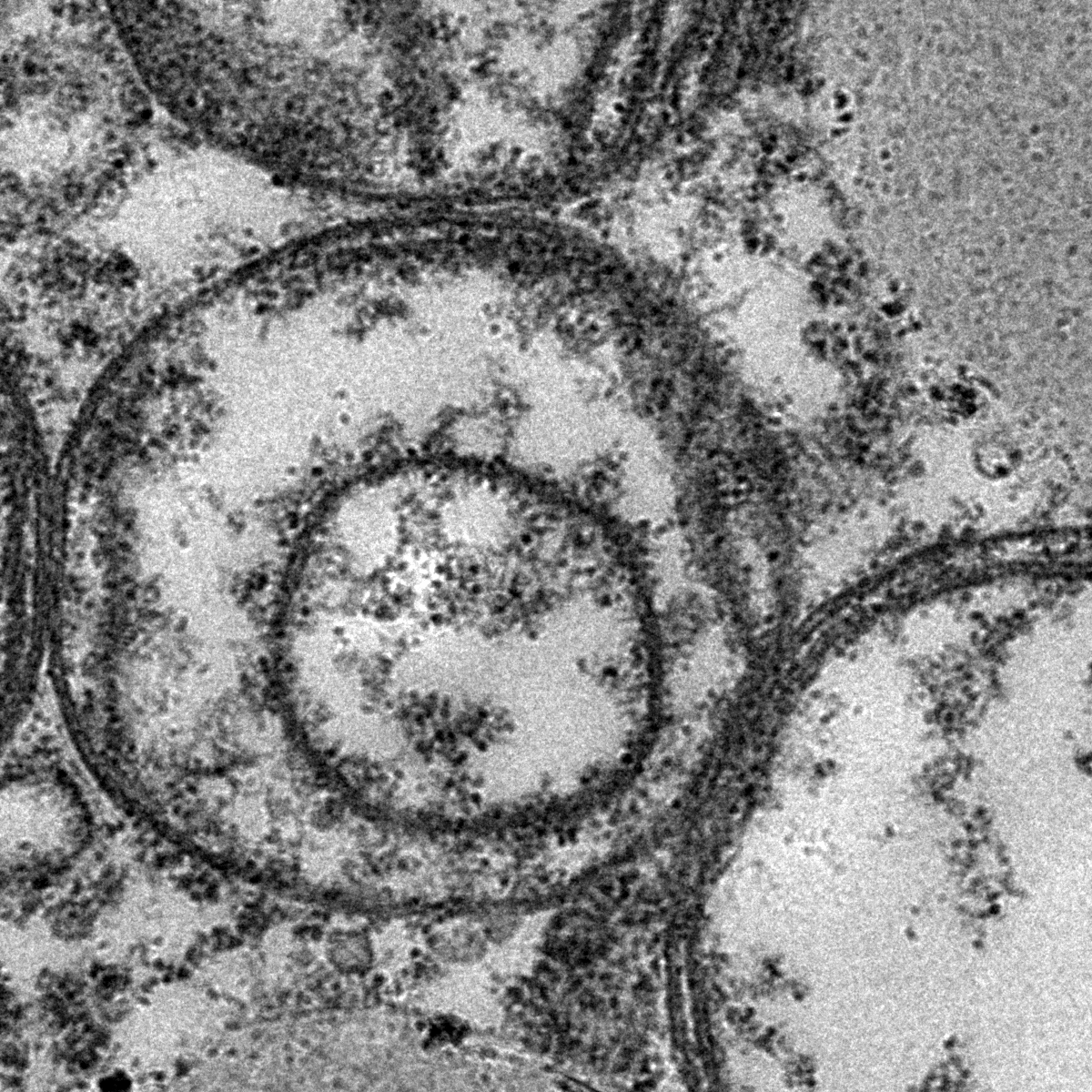

Supplement: Supplementary file 10 — Source data Fig. 3 [file 44319_2026_783_MOESM10_ESM.zip › Figure3/3L/STREMI_KO_coldstress_TEM_data-1.tif]

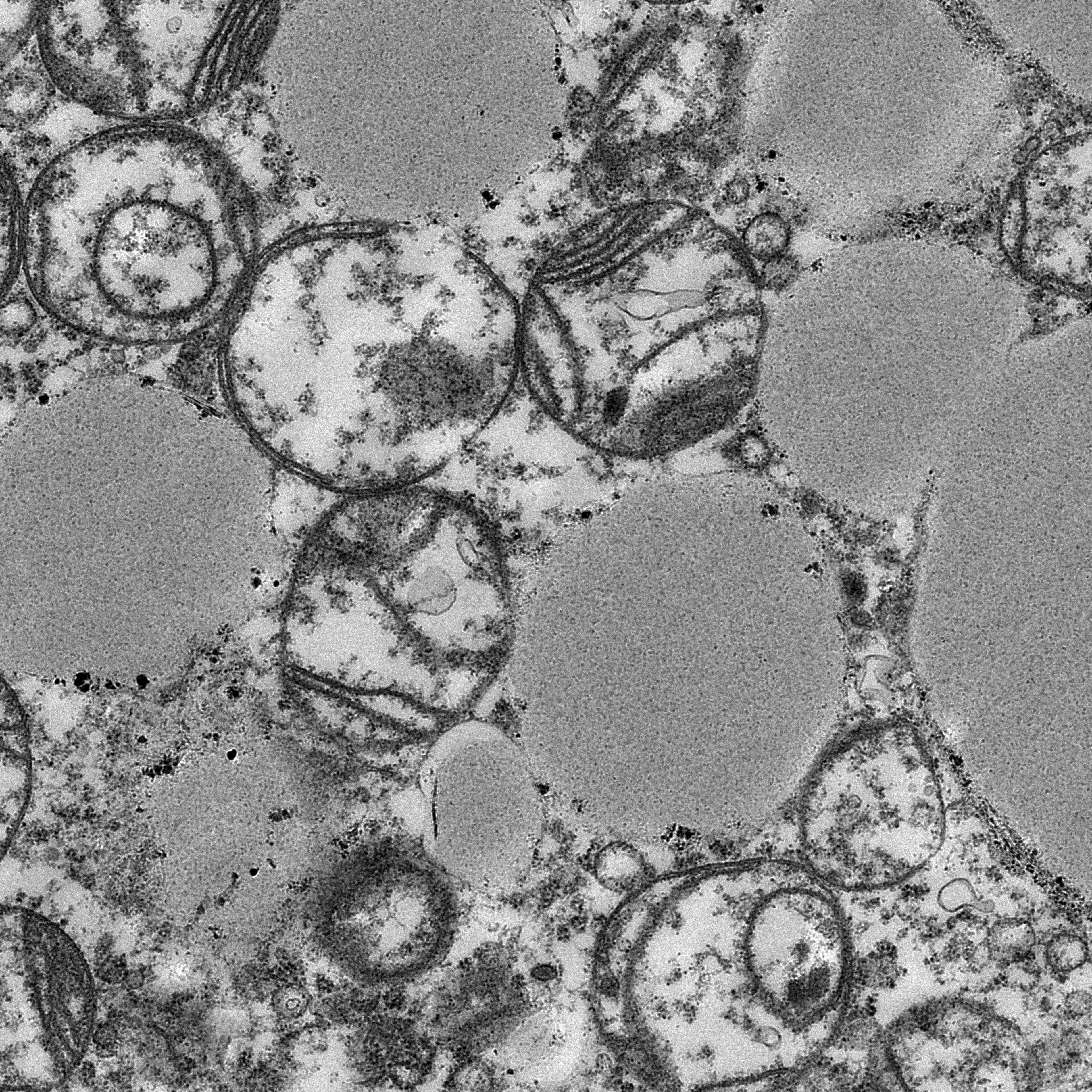

Supplement: Supplementary file 10 — Source data Fig. 3 [file 44319_2026_783_MOESM10_ESM.zip › Figure3/3L/STREMI_KO_coldstress_TEM_data.tif]

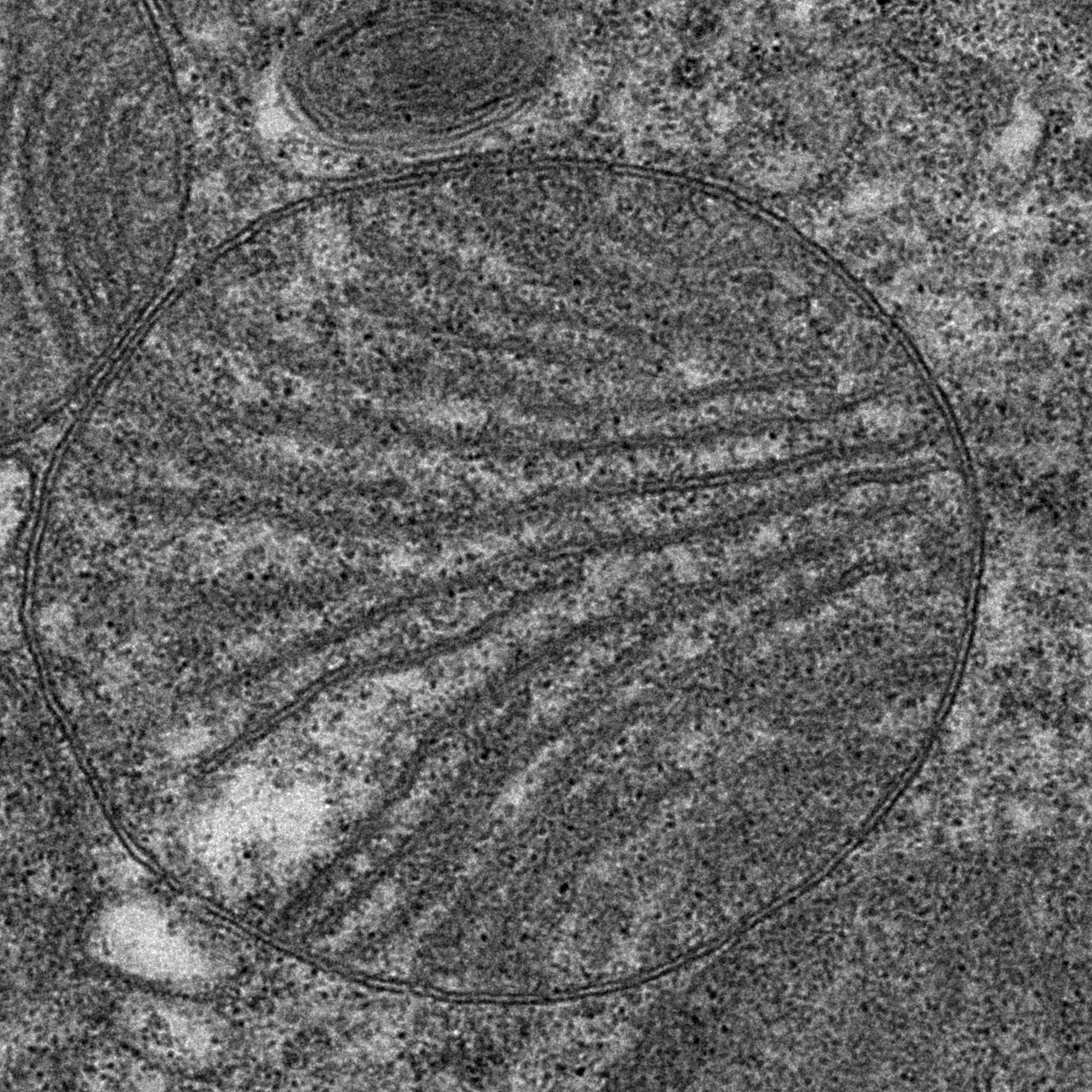

Supplement: Supplementary file 10 — Source data Fig. 3 [file 44319_2026_783_MOESM10_ESM.zip › Figure3/3L/WT_coldstress_TEM_data-1.tif]

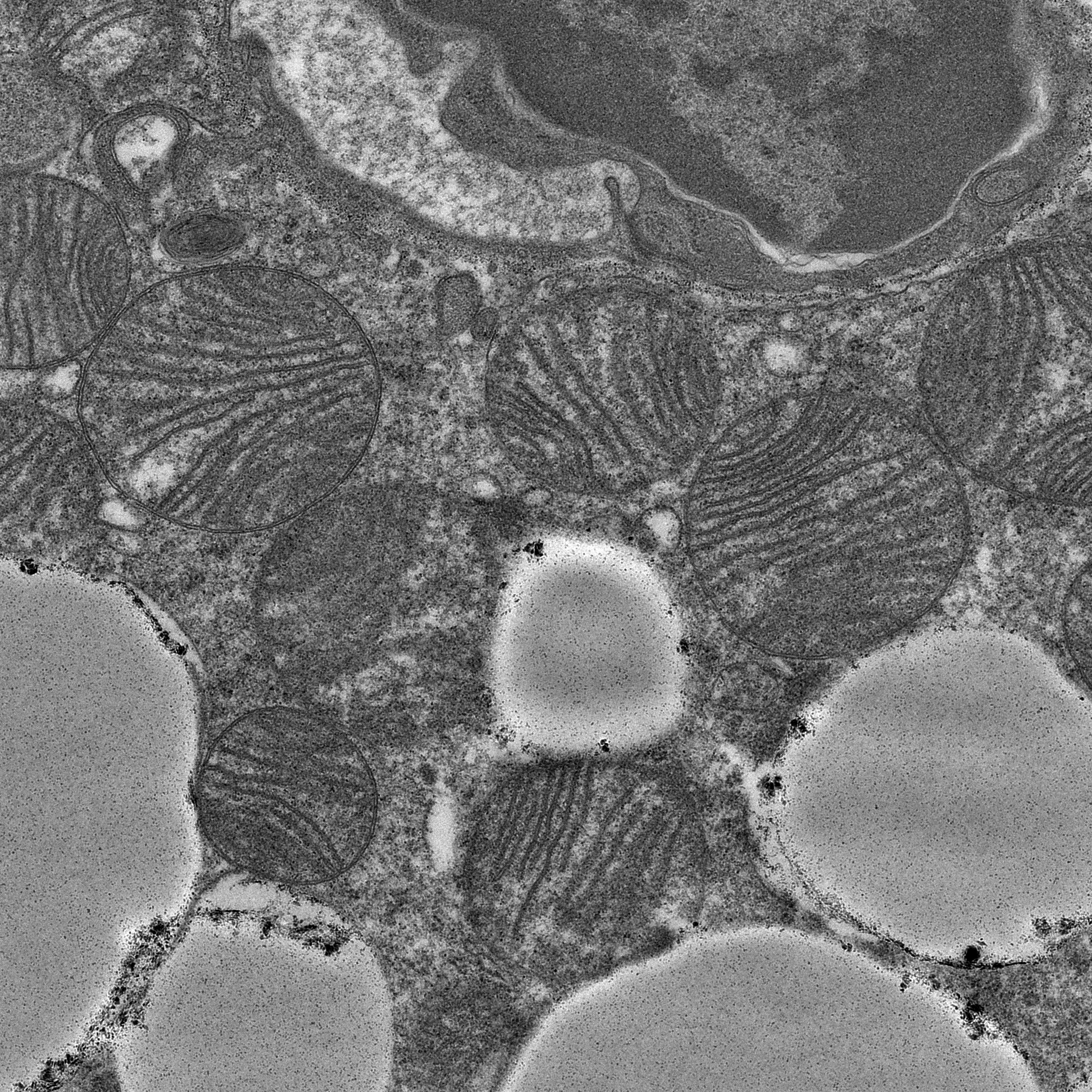

Supplement: Supplementary file 10 — Source data Fig. 3 [file 44319_2026_783_MOESM10_ESM.zip › Figure3/3L/WT_coldstress_TEM_data.tif]

Source Data Fig. 4

A

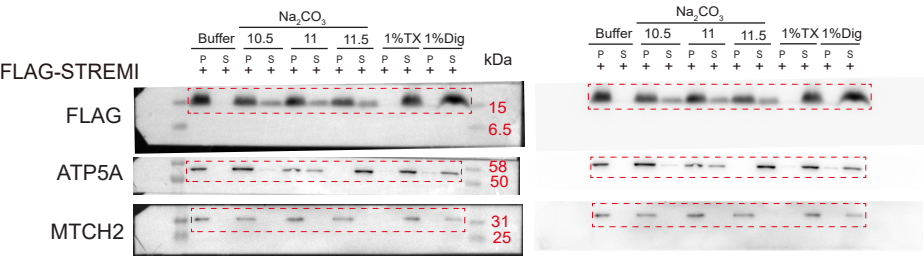

B

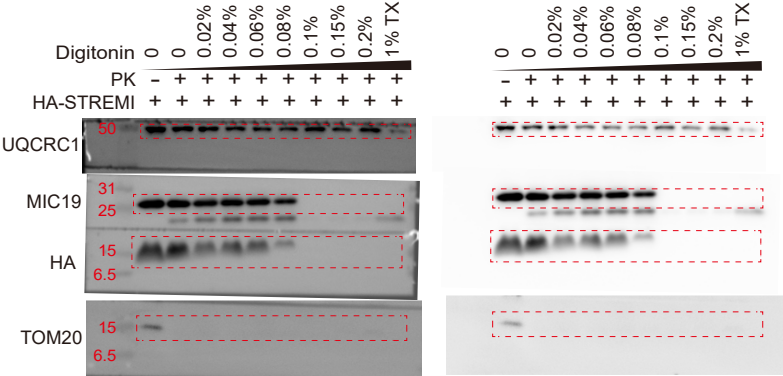

C

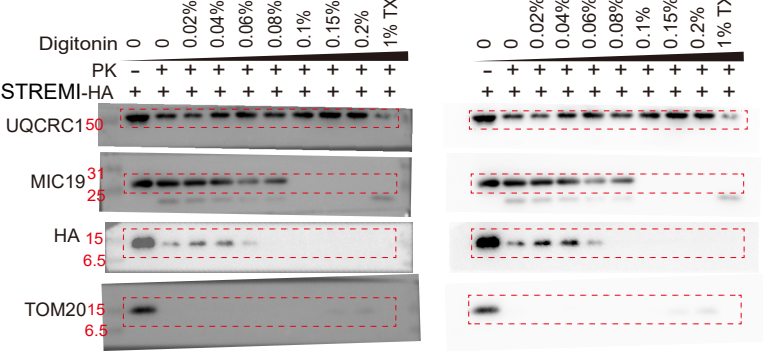

H

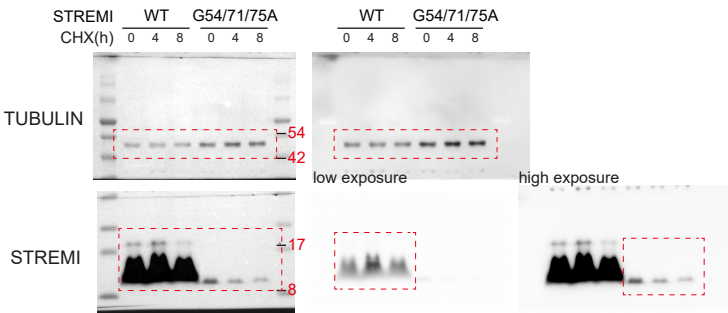

J

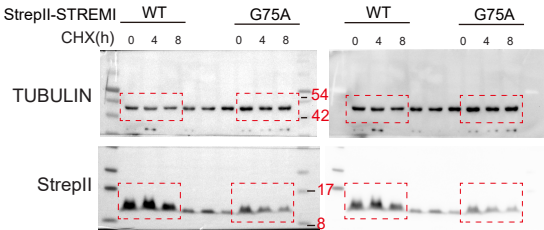

L

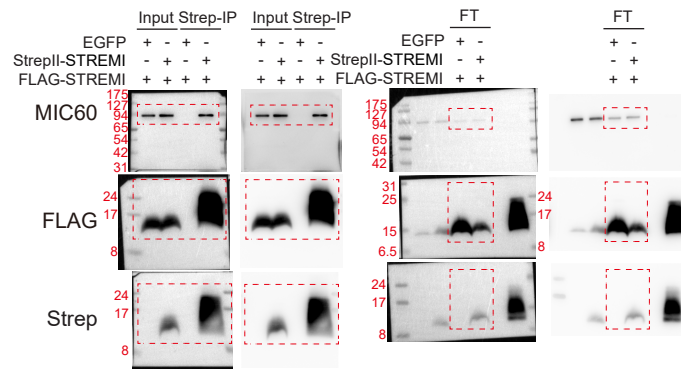

M

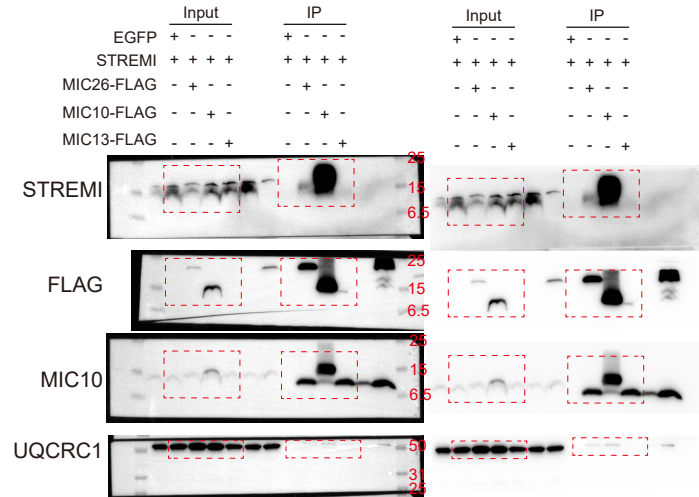

N

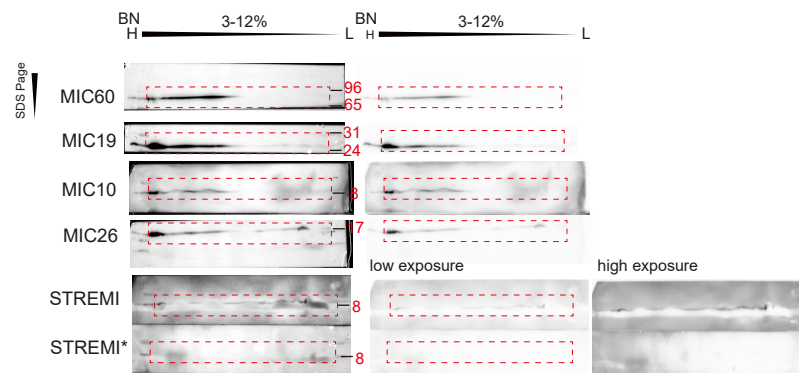

O

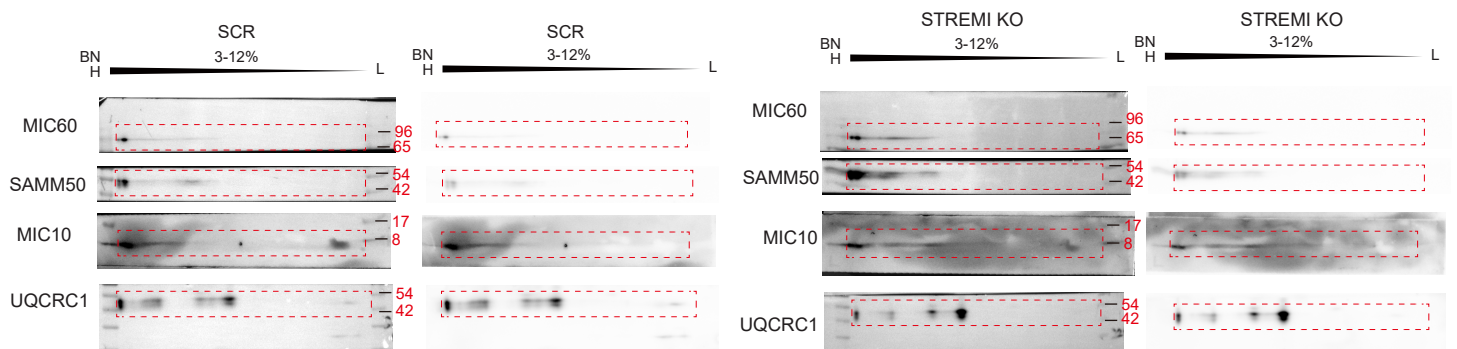

Supplement: Supplementary file 11 — Source data Fig. 4 [file 44319_2026_783_MOESM11_ESM.zip › Figure4/Figure_4_A_B_C_H_J_L-O(Cropped_area_and marker).pdf]

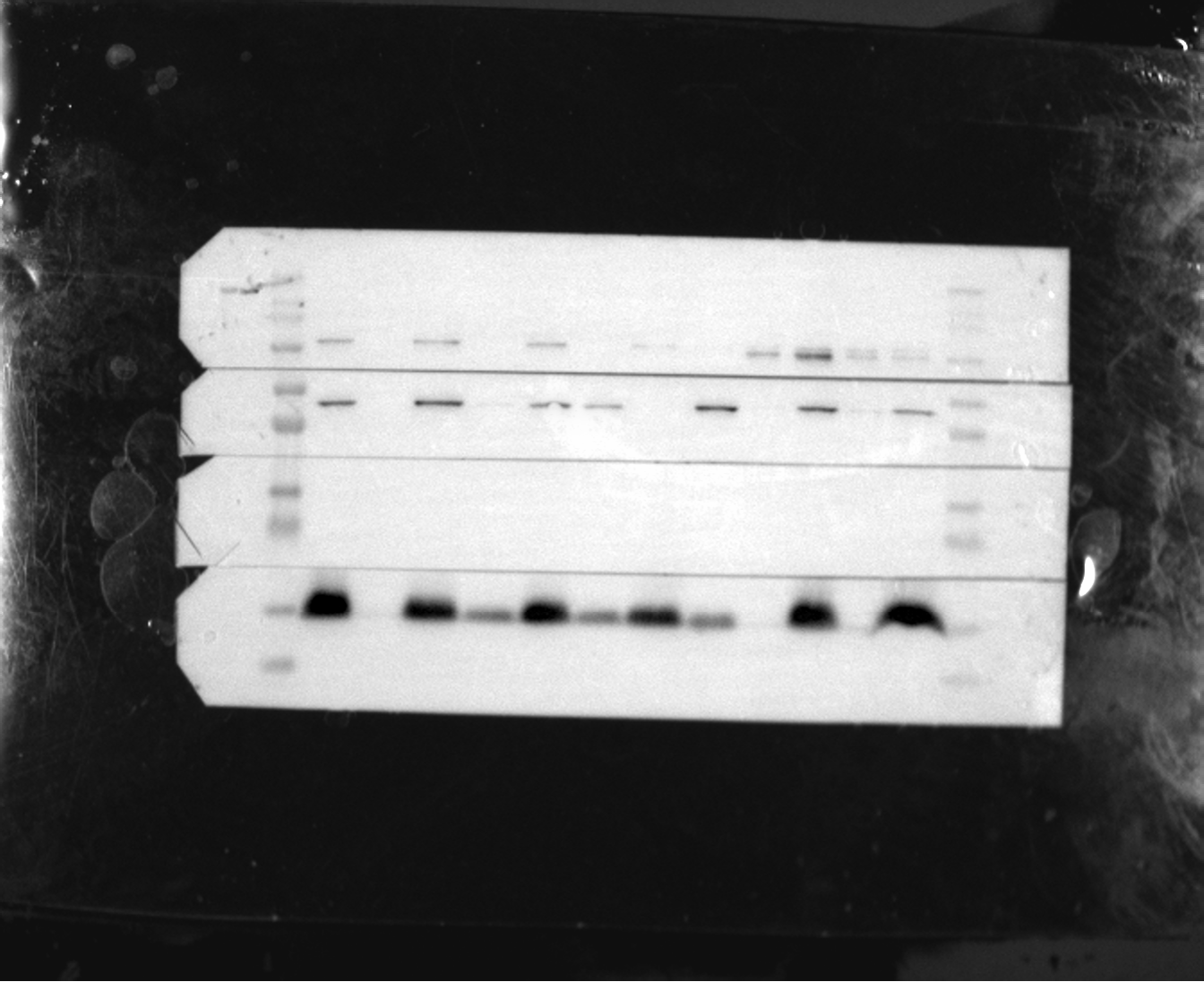

Supplement: Supplementary file 11 — Source data Fig. 4 [file 44319_2026_783_MOESM11_ESM.zip › Figure4/4A/Figure_4A- ATP5A_FLAG-Merge_data.Tif]

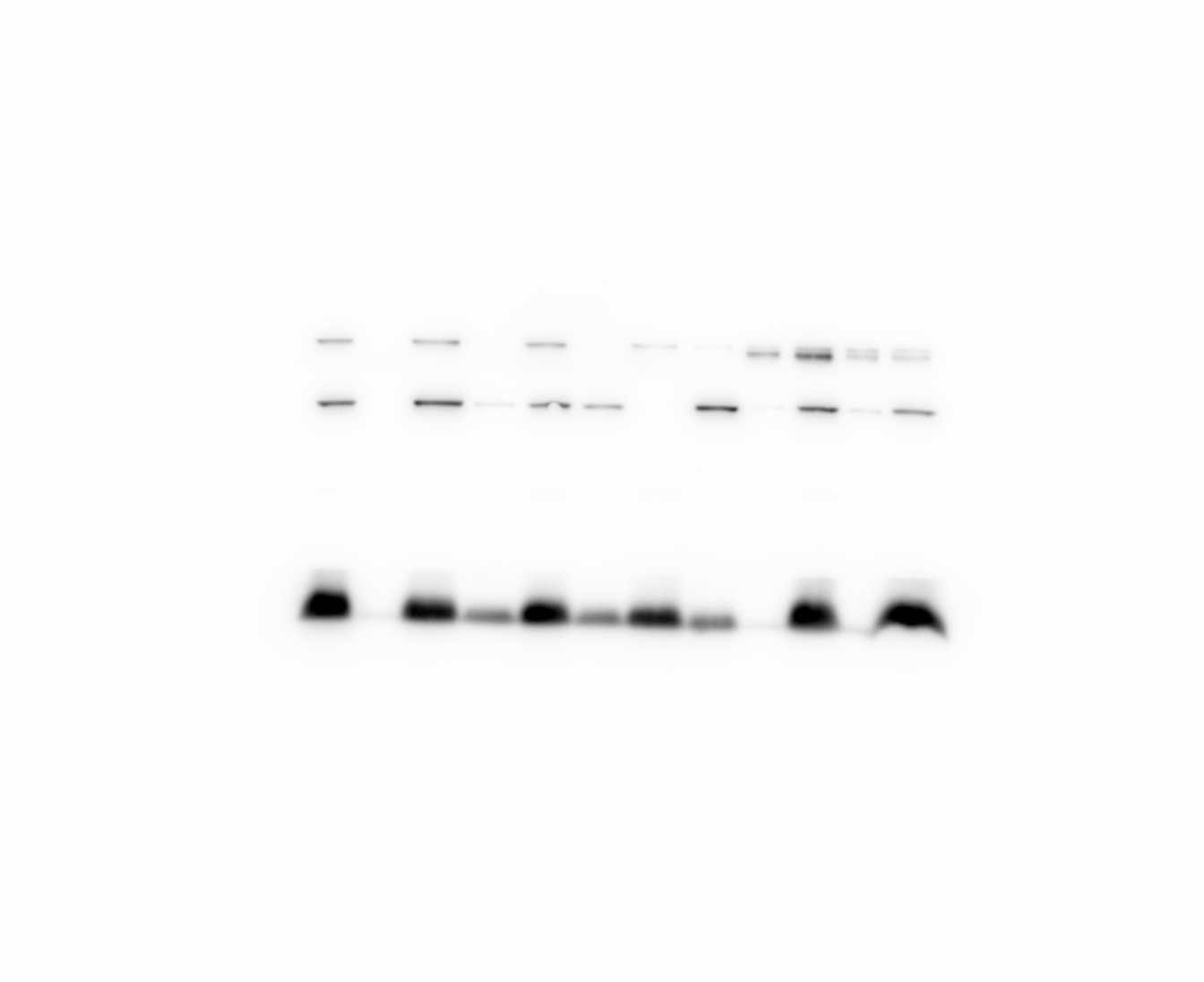

Supplement: Supplementary file 11 — Source data Fig. 4 [file 44319_2026_783_MOESM11_ESM.zip › Figure4/4A/Figure_4A- ATP5A_FLAG-Raw_data.Tif]

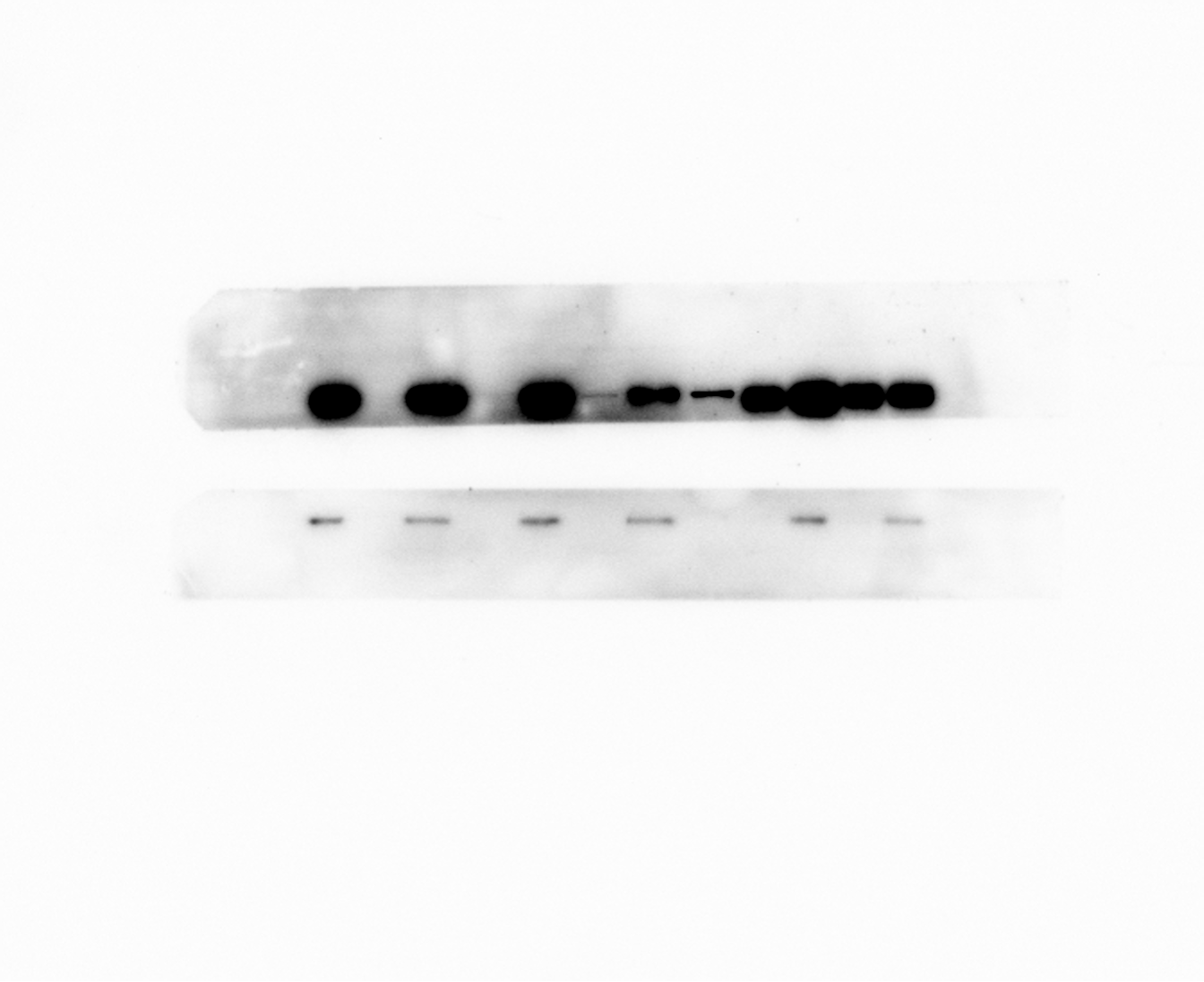

Supplement: Supplementary file 11 — Source data Fig. 4 [file 44319_2026_783_MOESM11_ESM.zip › Figure4/4A/Figure_4A-MTCH2-Merge_data.tif]

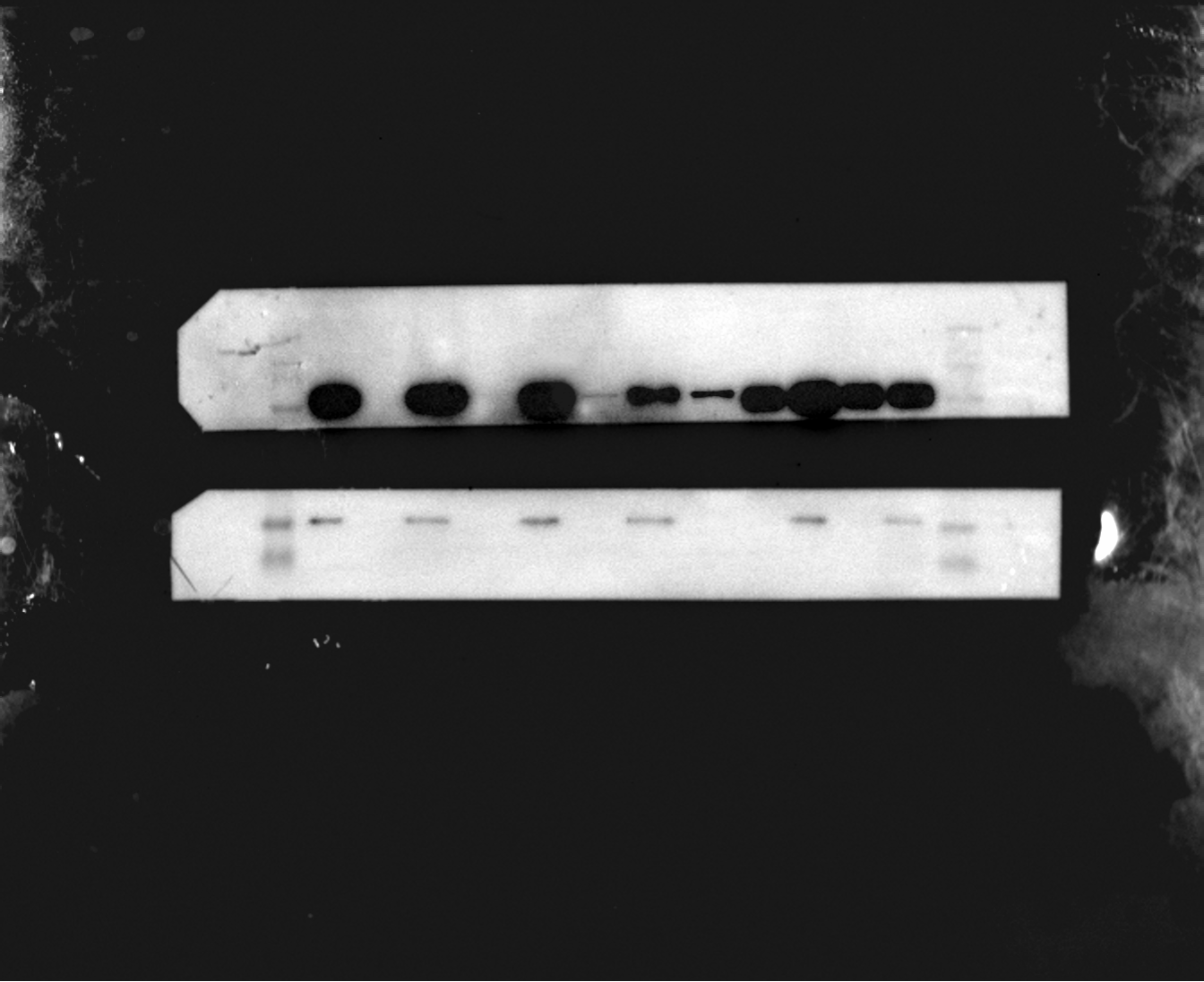

Supplement: Supplementary file 11 — Source data Fig. 4 [file 44319_2026_783_MOESM11_ESM.zip › Figure4/4A/Figure_4A-MTCH2-Raw_data.tif]

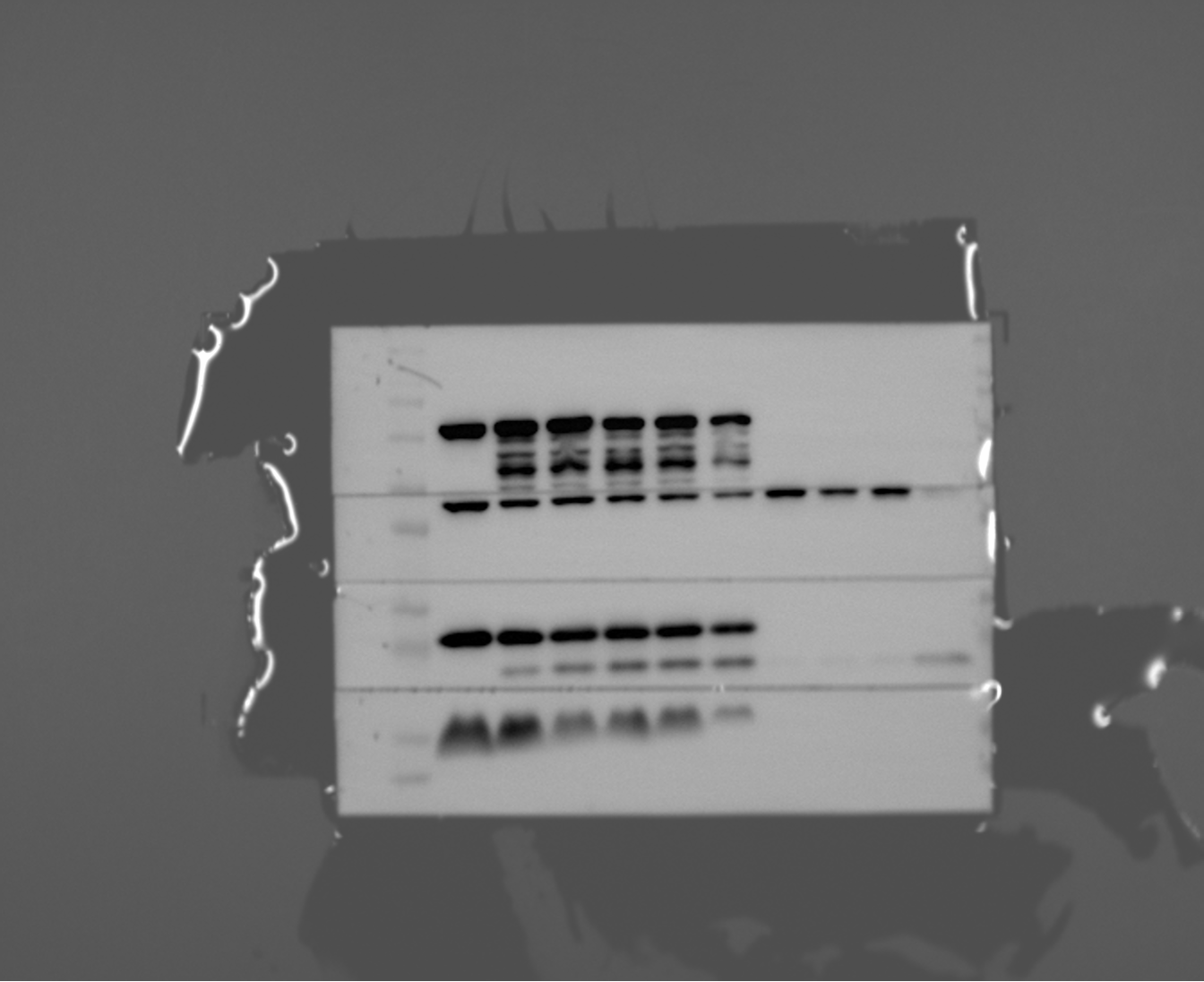

Supplement: Supplementary file 11 — Source data Fig. 4 [file 44319_2026_783_MOESM11_ESM.zip › Figure4/4B/Figure_4B-MIC19_HA-Merge_data.Tif]

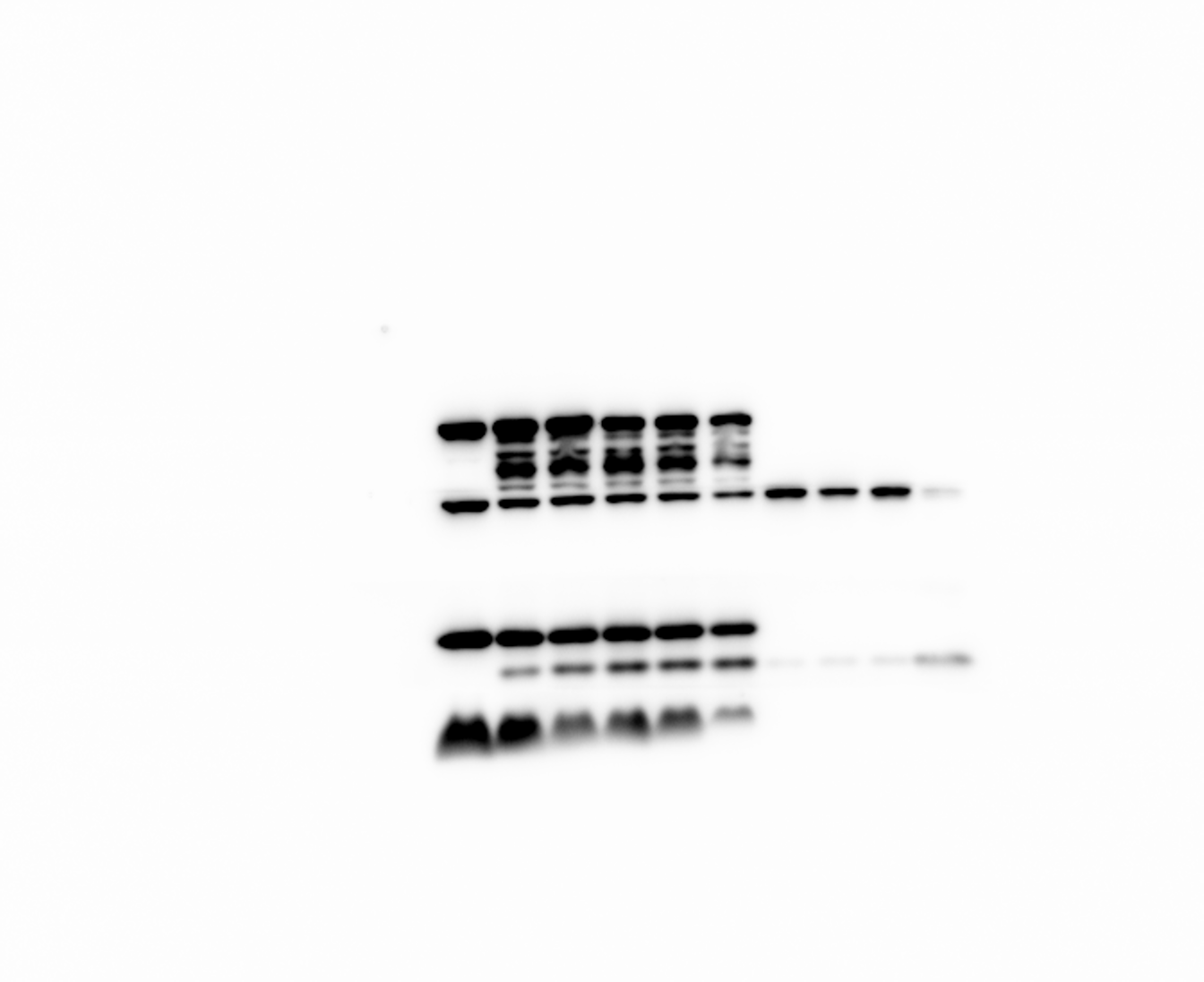

Supplement: Supplementary file 11 — Source data Fig. 4 [file 44319_2026_783_MOESM11_ESM.zip › Figure4/4B/Figure_4B-MIC19_HA-Raw_data.Tif]

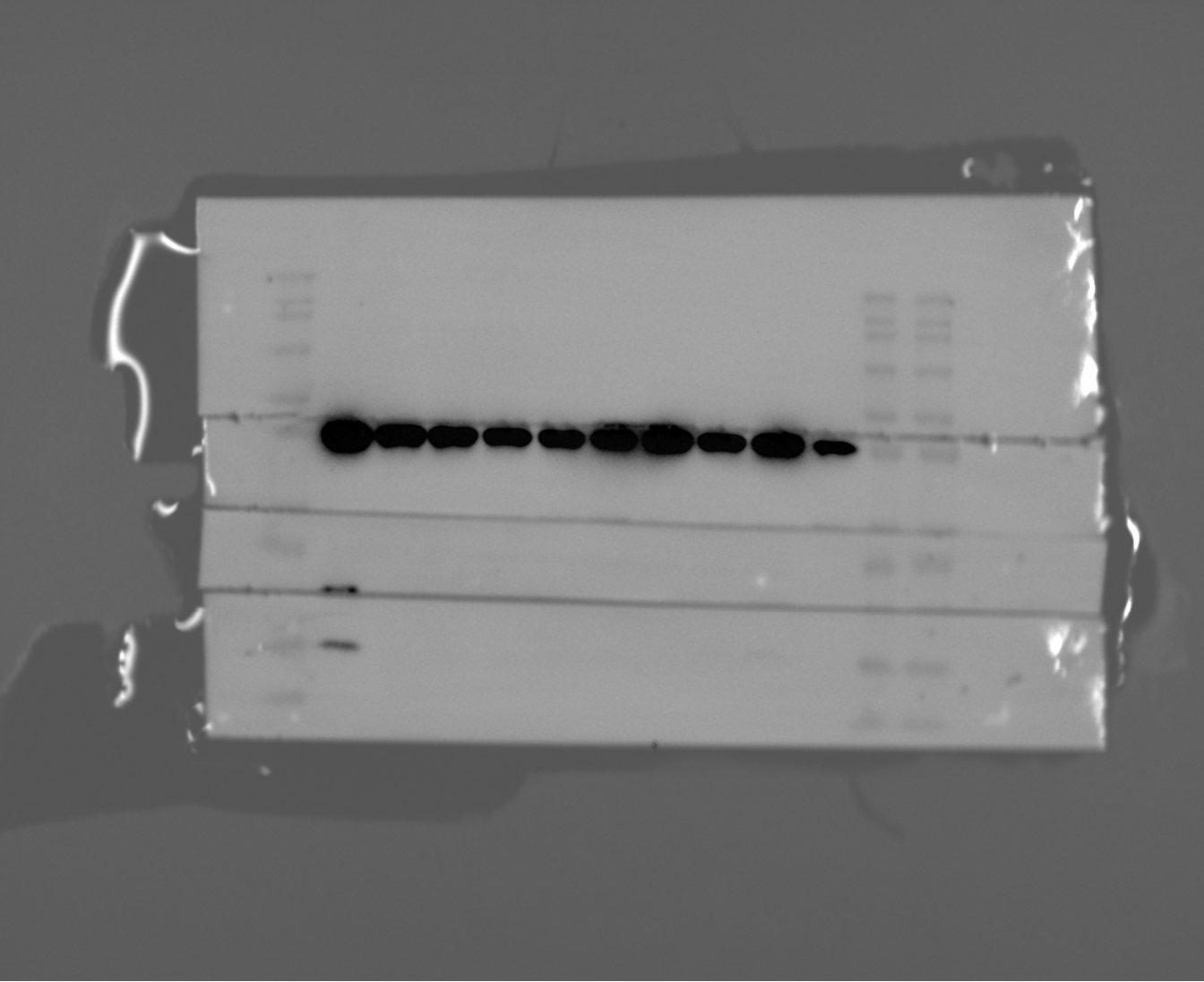

Supplement: Supplementary file 11 — Source data Fig. 4 [file 44319_2026_783_MOESM11_ESM.zip › Figure4/4B/Figure_4B-TOM20-Merge_data.Tif]

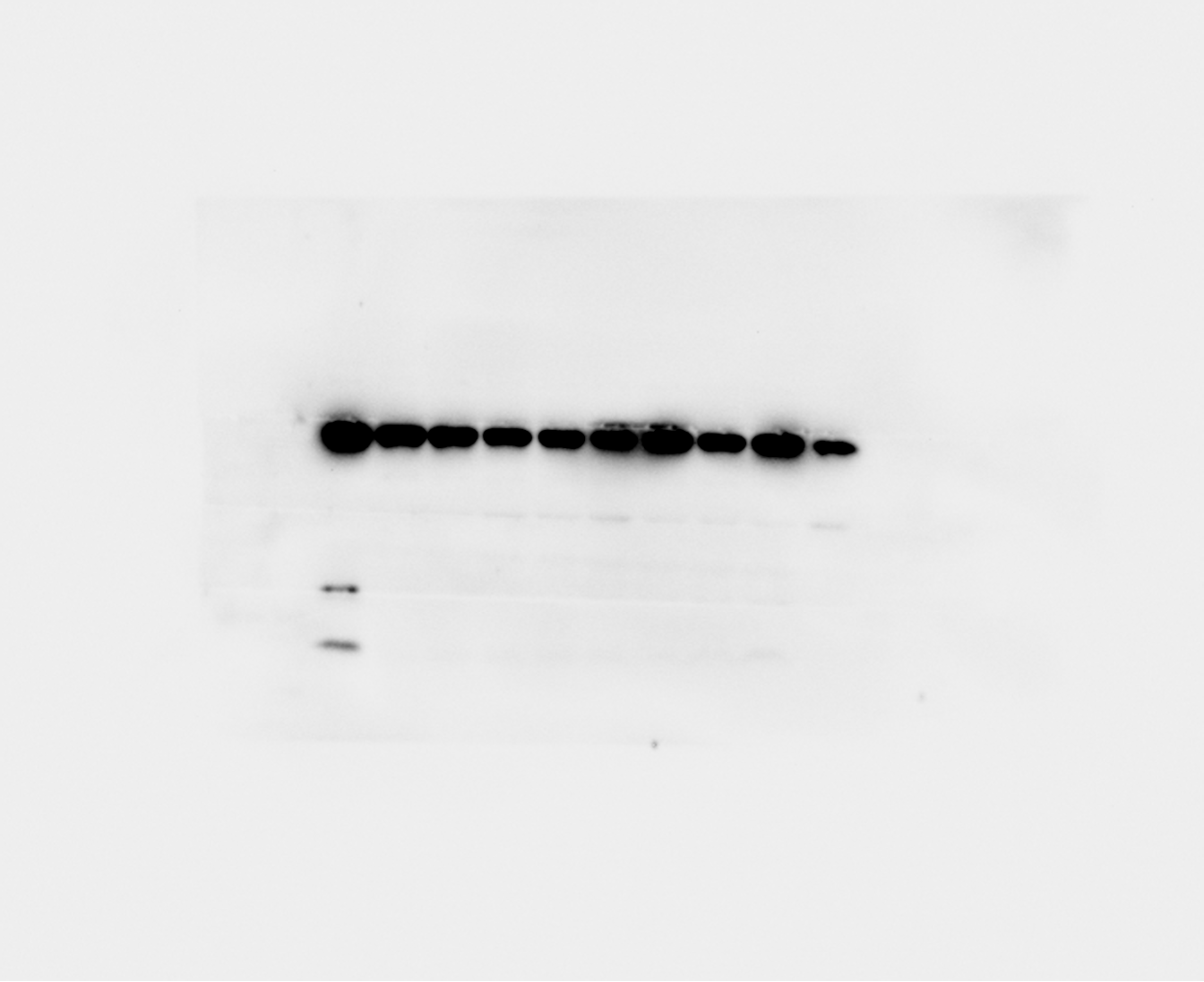

Supplement: Supplementary file 11 — Source data Fig. 4 [file 44319_2026_783_MOESM11_ESM.zip › Figure4/4B/Figure_4B-TOM20-Raw_data.tif]

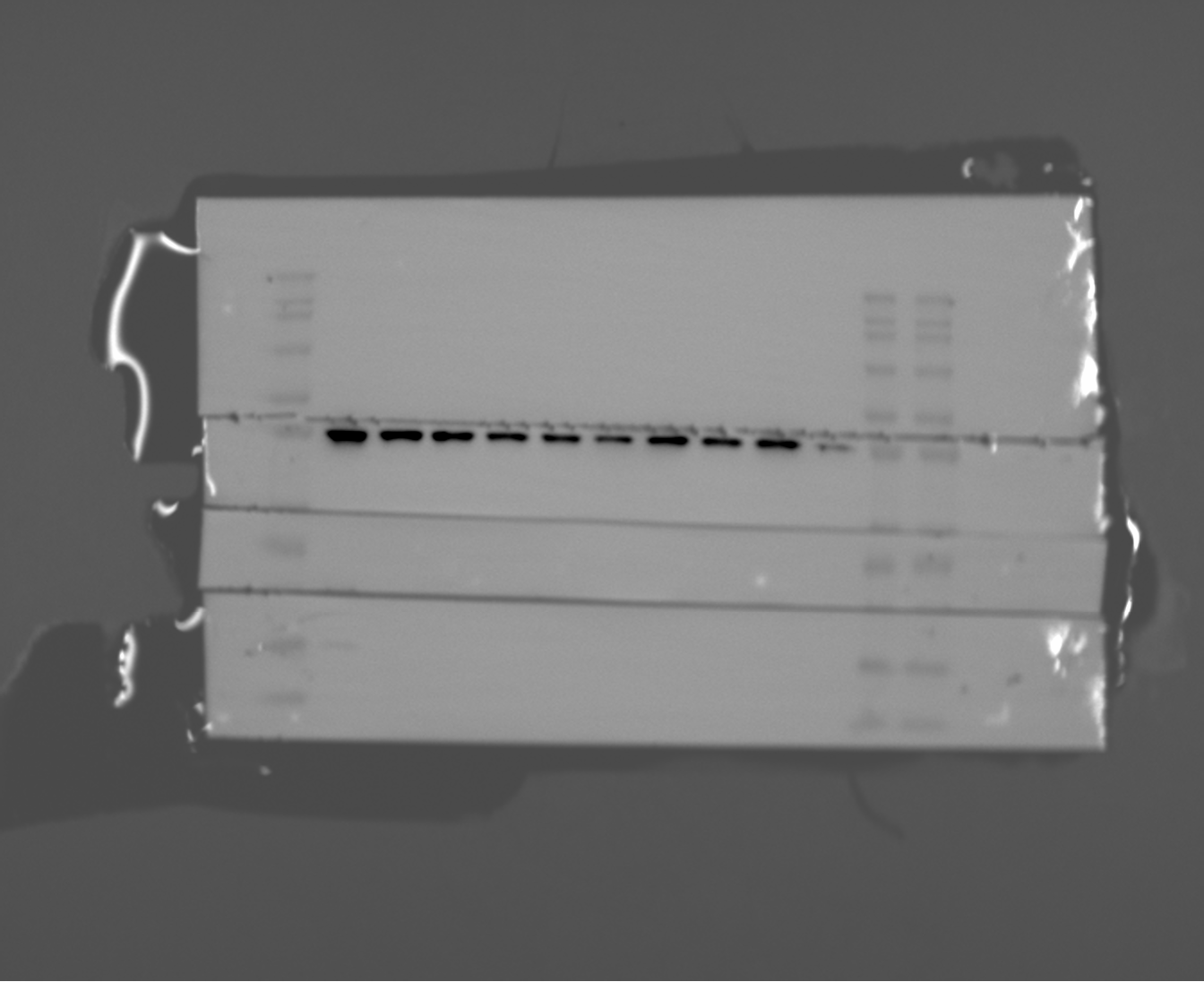

Supplement: Supplementary file 11 — Source data Fig. 4 [file 44319_2026_783_MOESM11_ESM.zip › Figure4/4B/Figure_4B-UQCRC1-Merge_data.Tif]

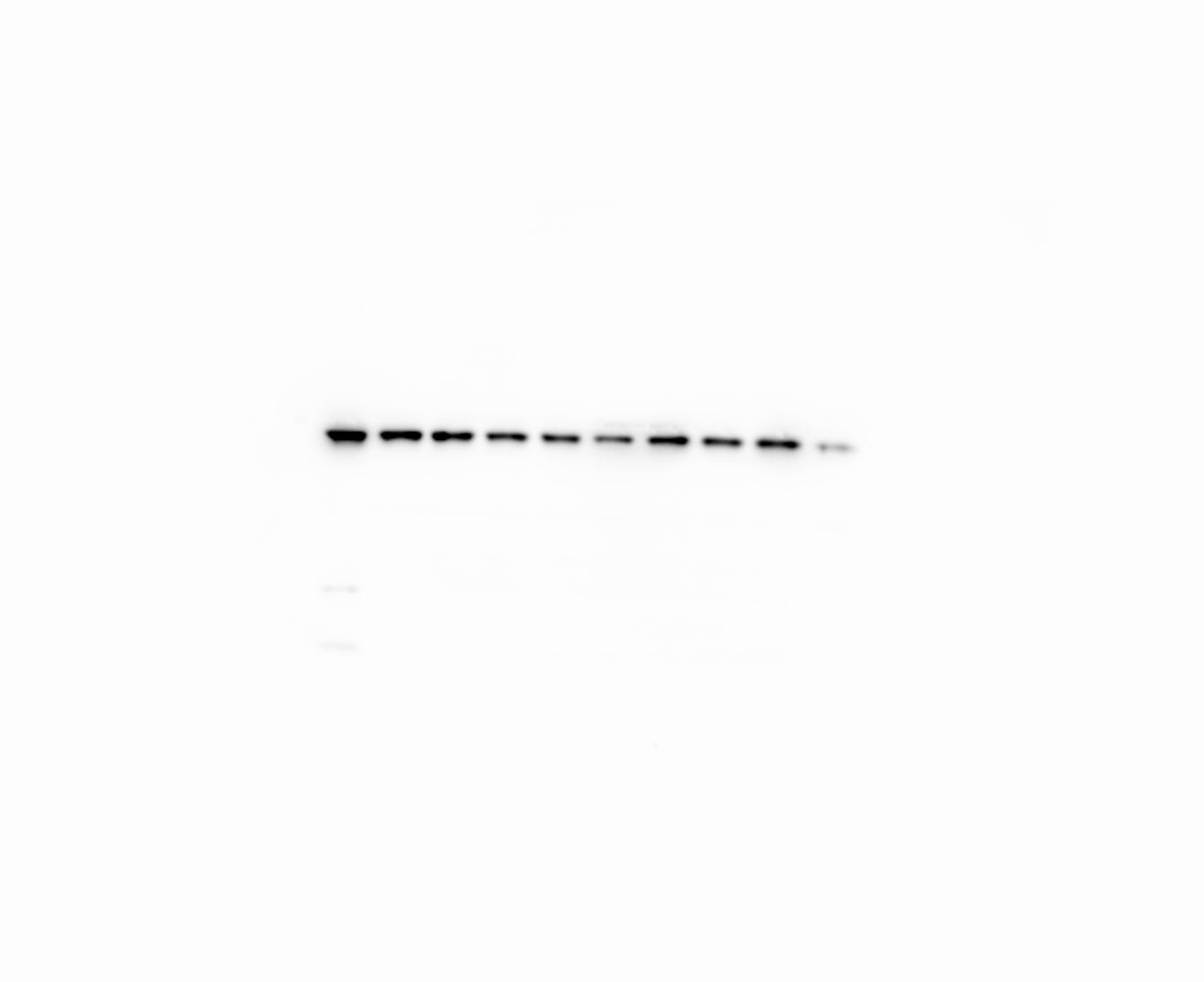

Supplement: Supplementary file 11 — Source data Fig. 4 [file 44319_2026_783_MOESM11_ESM.zip › Figure4/4B/Figure_4B-UQCRC1-Raw_data.Tif]

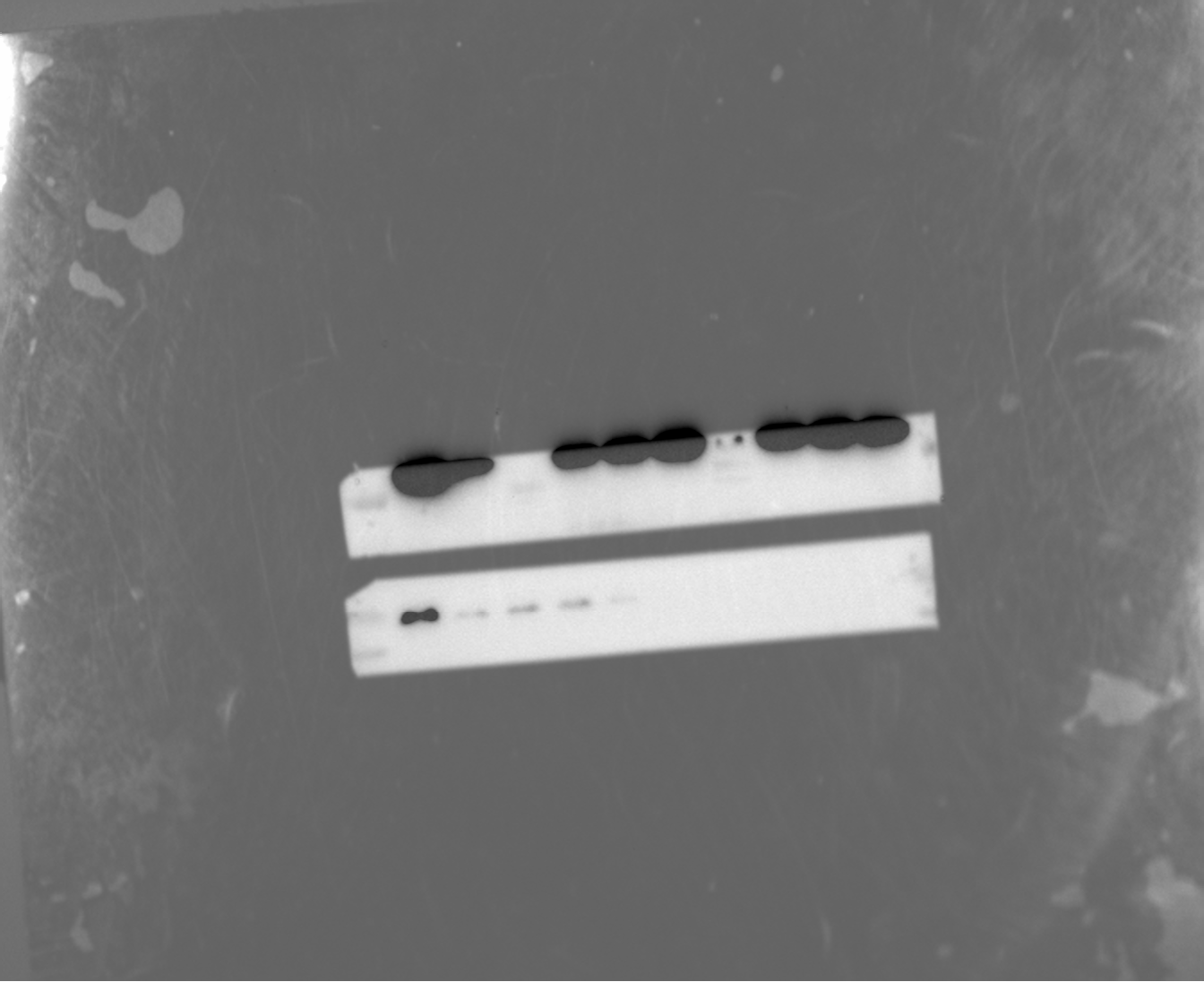

Supplement: Supplementary file 11 — Source data Fig. 4 [file 44319_2026_783_MOESM11_ESM.zip › Figure4/4C/Figure_4C-HA-Merge_data.tif]

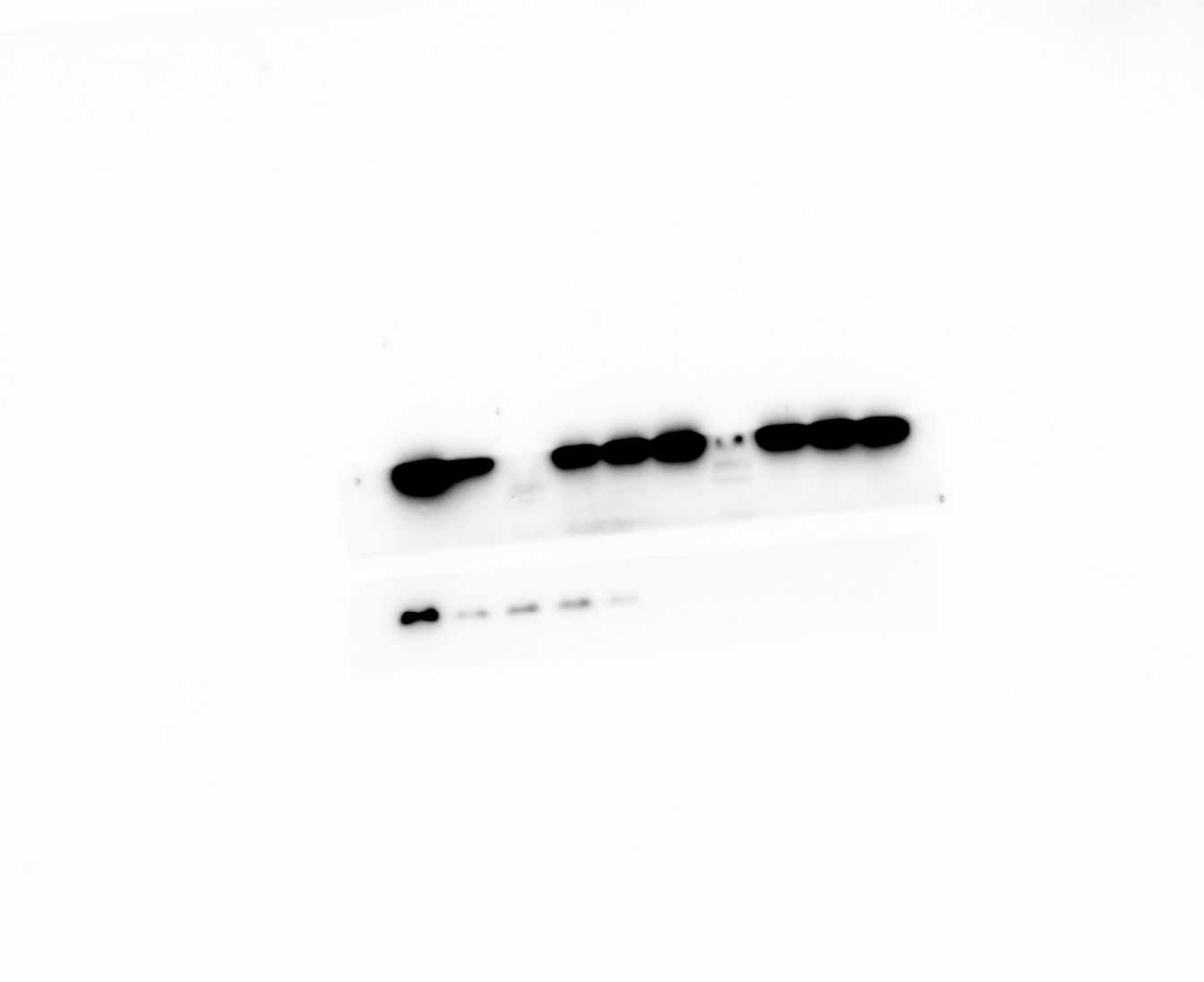

Supplement: Supplementary file 11 — Source data Fig. 4 [file 44319_2026_783_MOESM11_ESM.zip › Figure4/4C/Figure_4C-HA-Raw_data.tif]

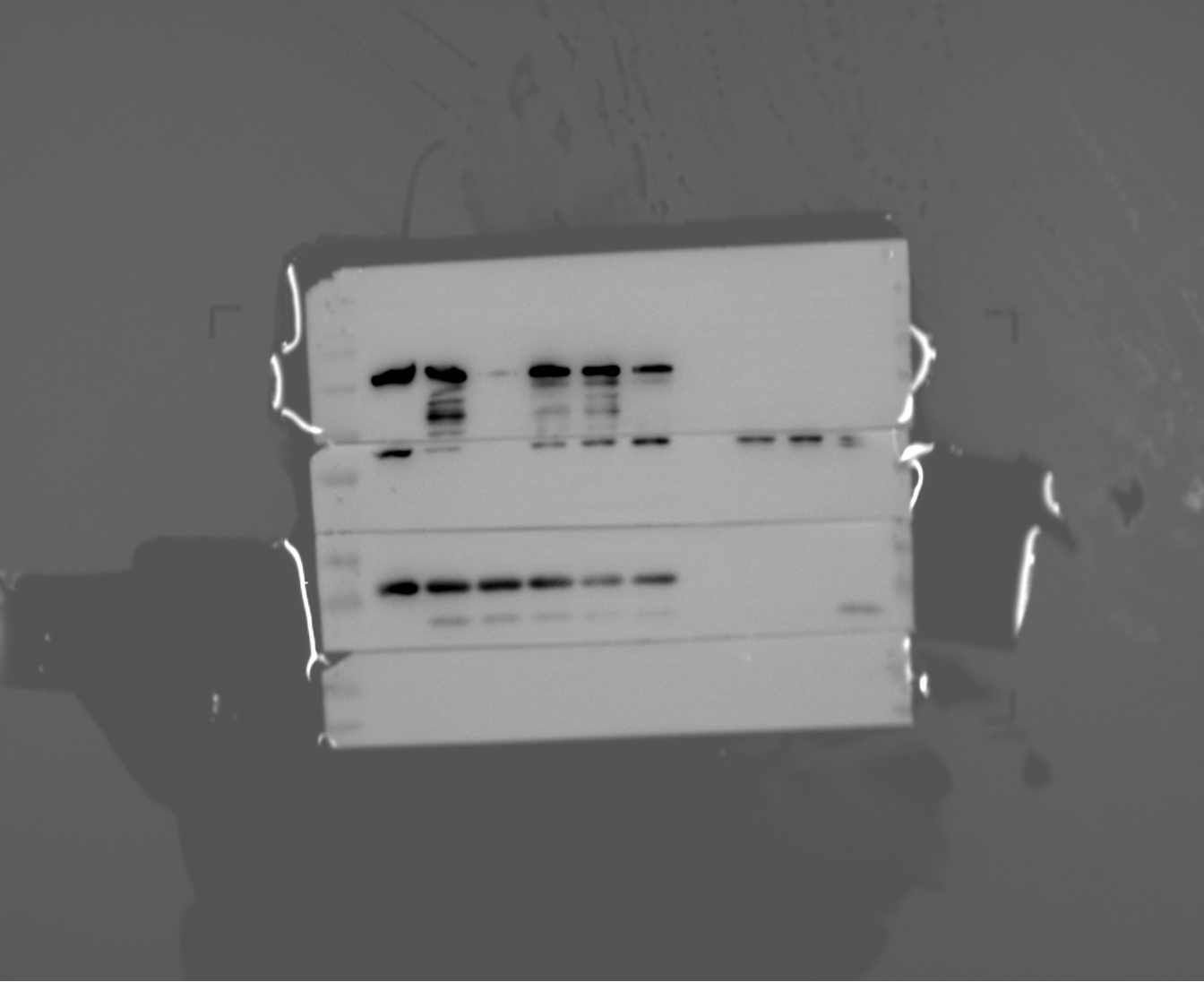

Supplement: Supplementary file 11 — Source data Fig. 4 [file 44319_2026_783_MOESM11_ESM.zip › Figure4/4C/Figure_4C-MIC19-Merge_data.tif]

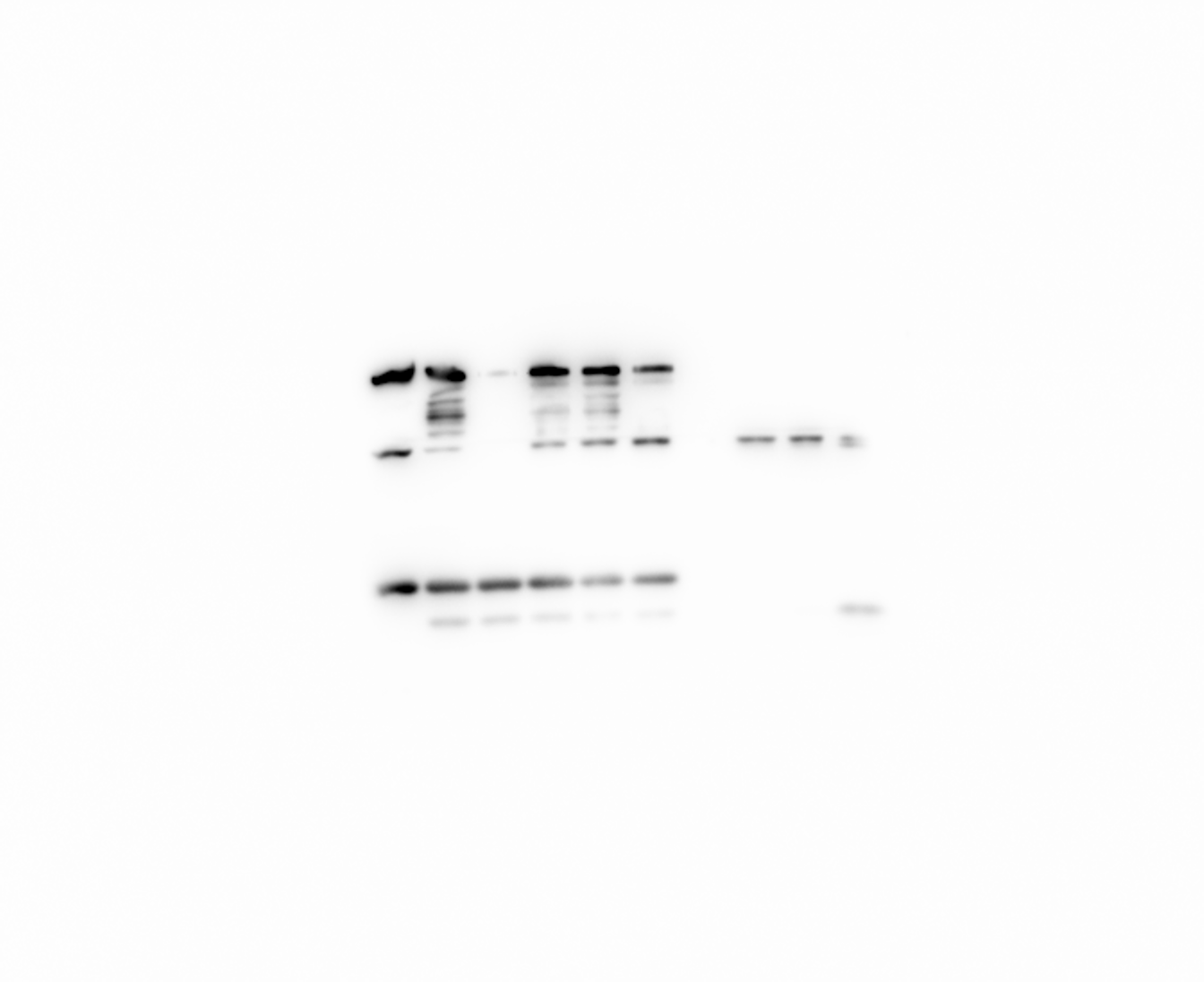

Supplement: Supplementary file 11 — Source data Fig. 4 [file 44319_2026_783_MOESM11_ESM.zip › Figure4/4C/Figure_4C-MIC19-Raw_data.Tif]

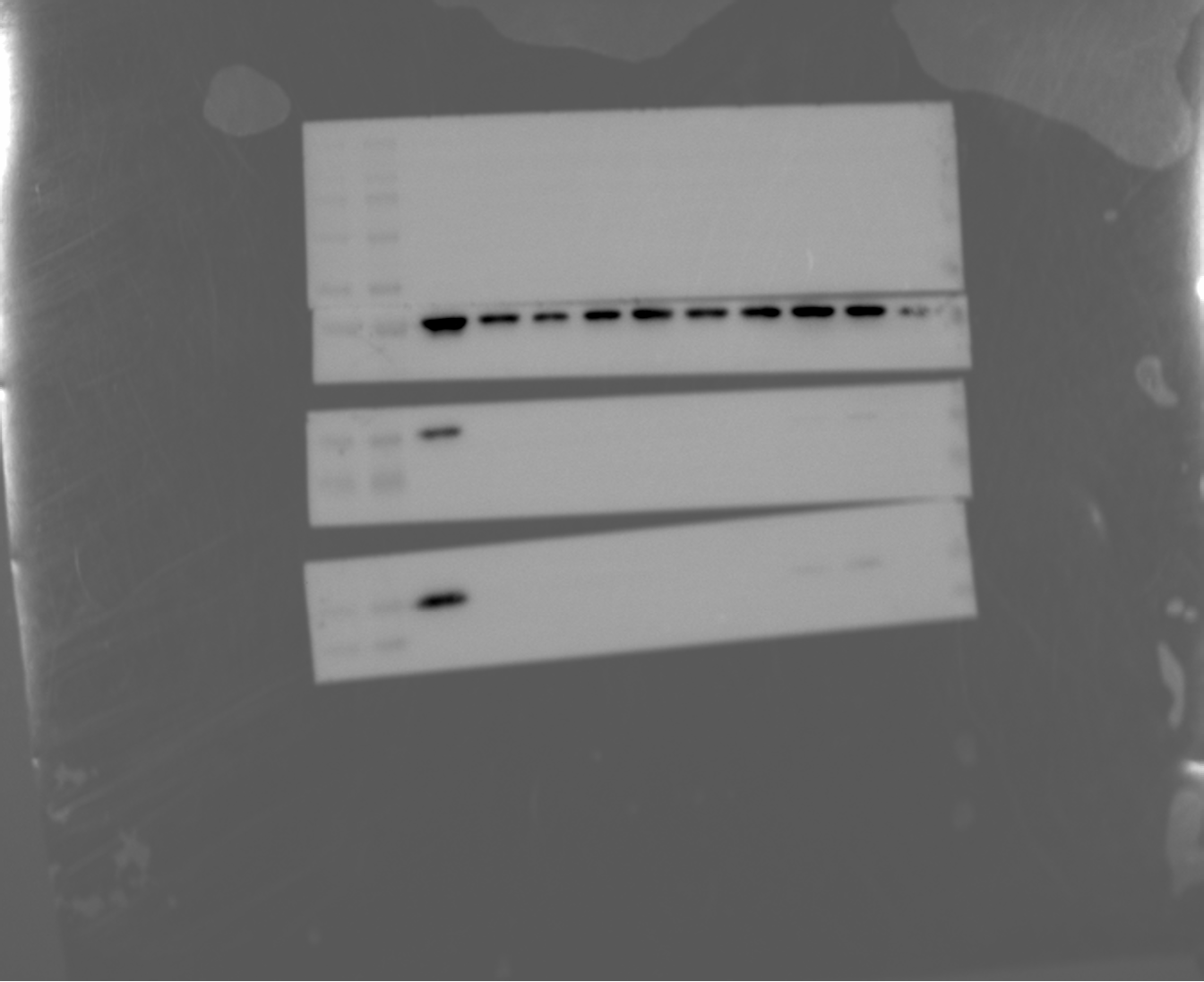

Supplement: Supplementary file 11 — Source data Fig. 4 [file 44319_2026_783_MOESM11_ESM.zip › Figure4/4C/Figure_4C-UQCRC1_TOM20-Merge_data.tif]

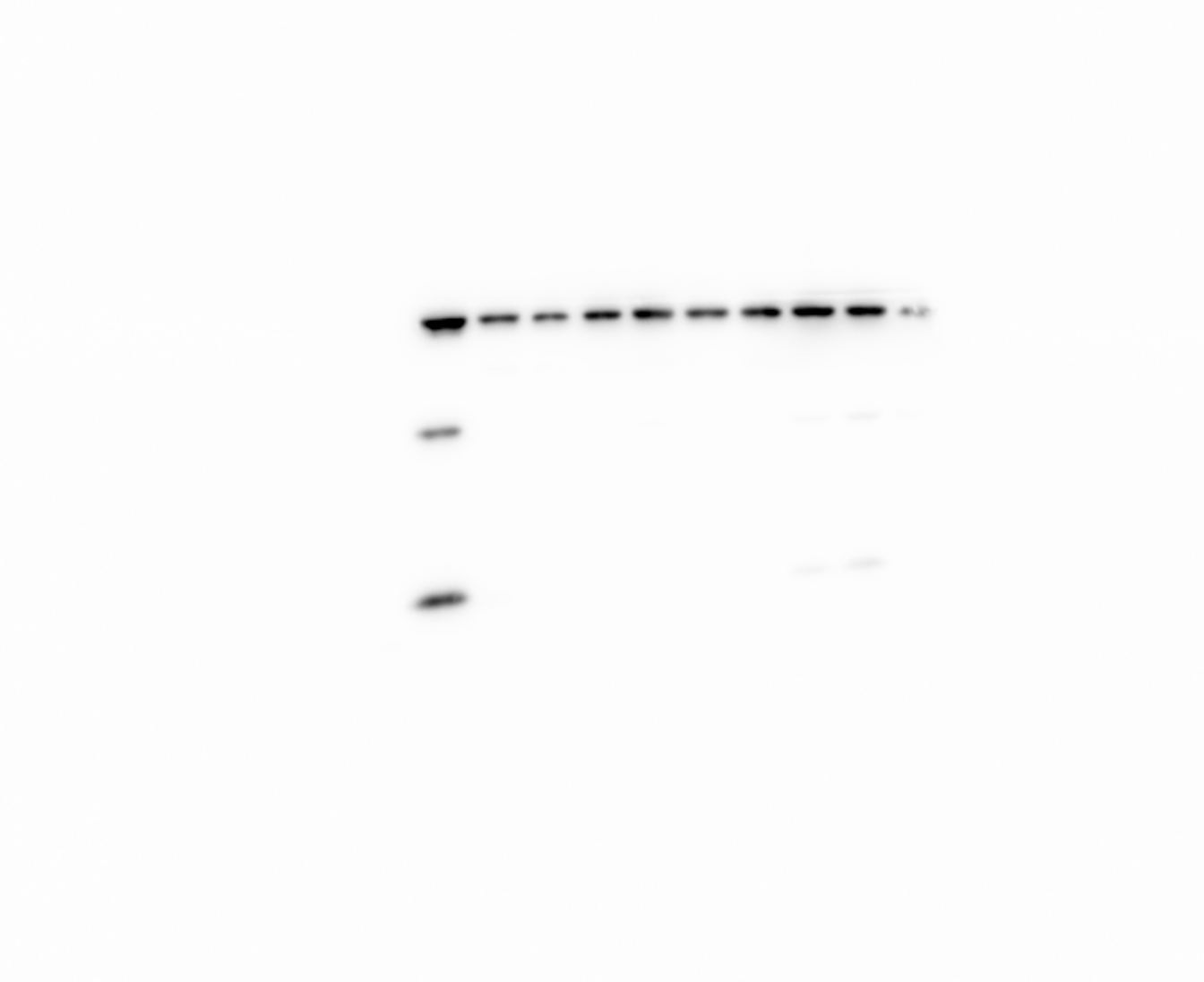

Supplement: Supplementary file 11 — Source data Fig. 4 [file 44319_2026_783_MOESM11_ESM.zip › Figure4/4C/Figure_4C-UQCRC1_TOM20-Raw_data.Tif]

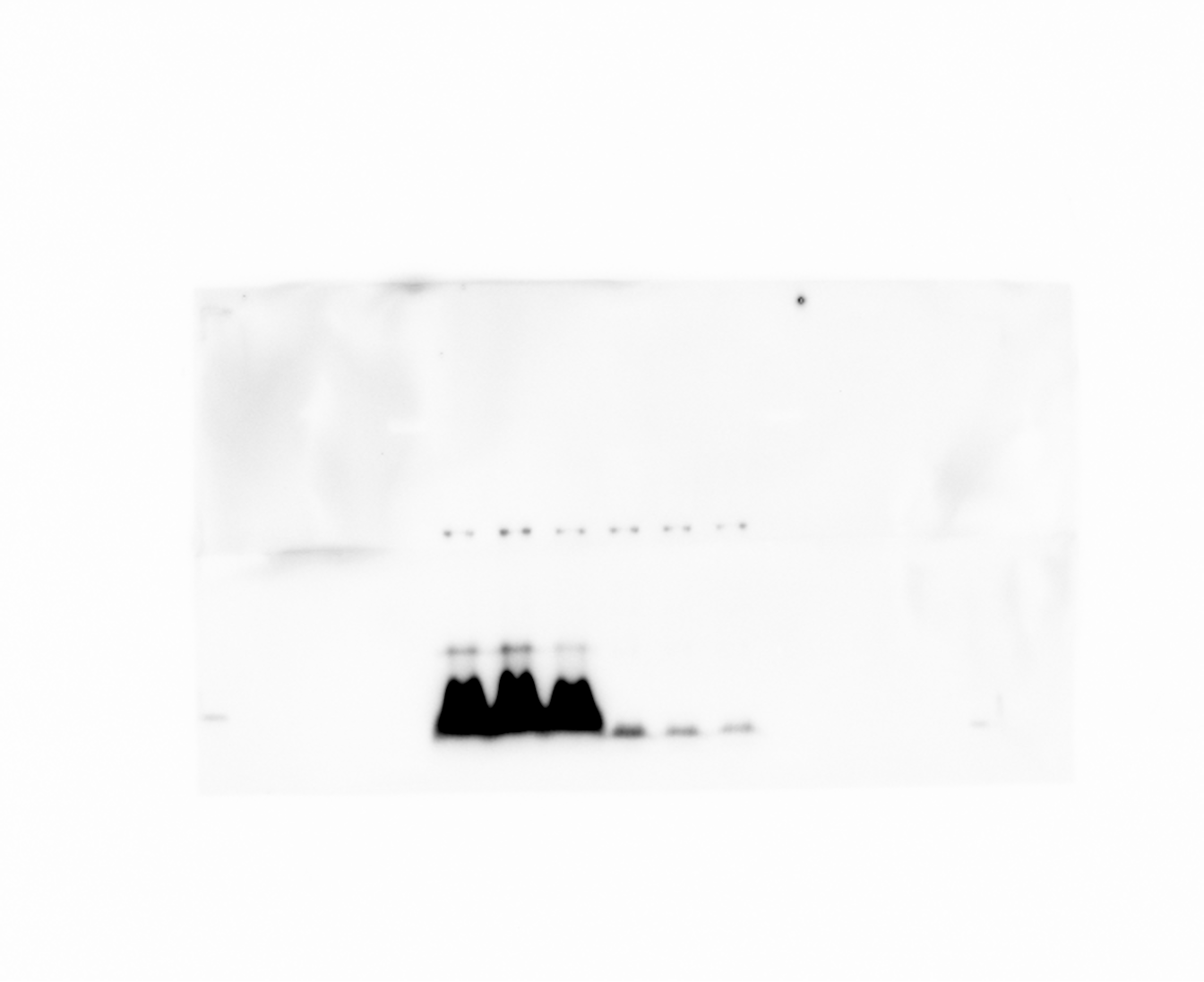

Supplement: Supplementary file 11 — Source data Fig. 4 [file 44319_2026_783_MOESM11_ESM.zip › Figure4/4H/STREMI-High exposure-raw.Tif]

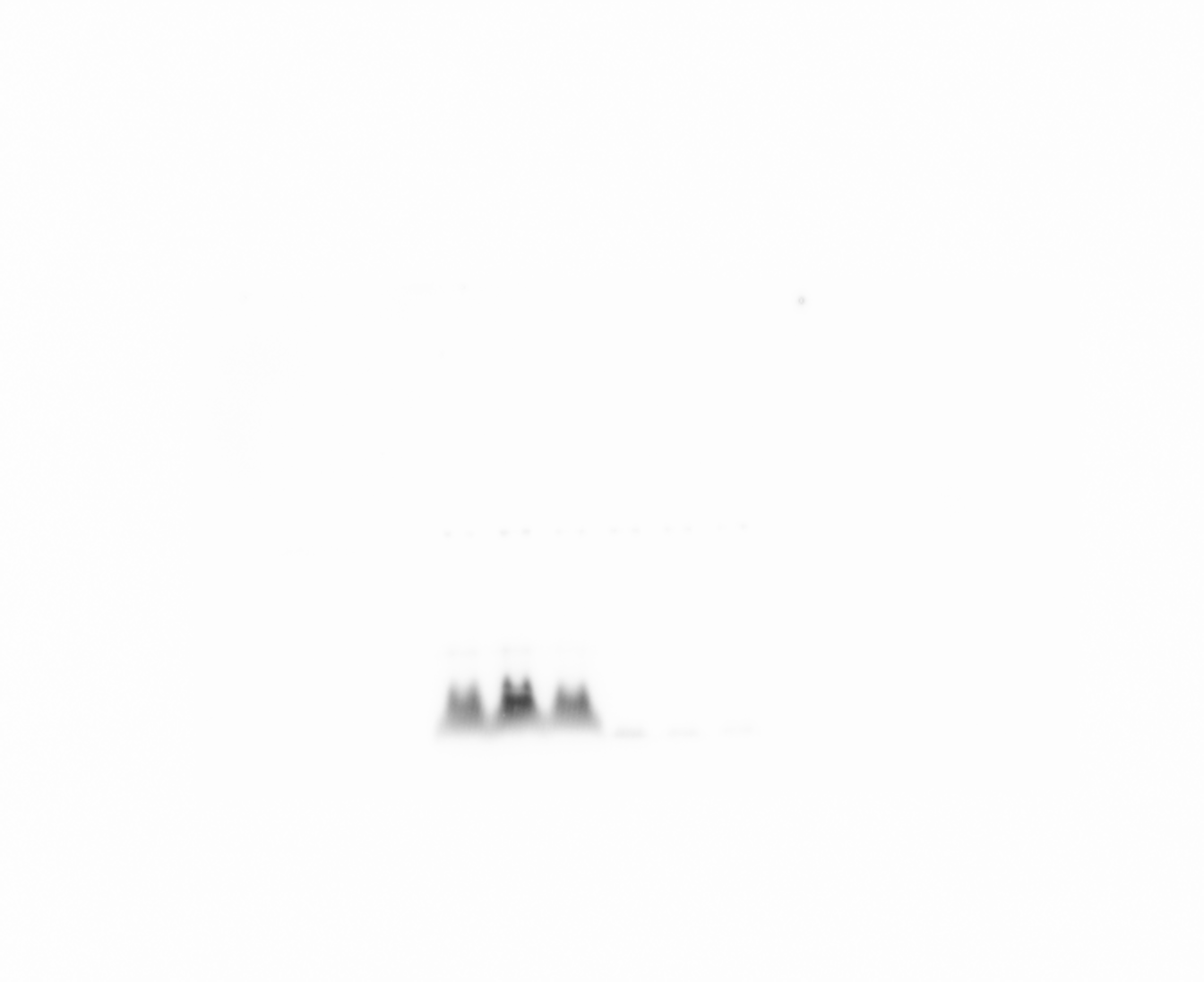

Supplement: Supplementary file 11 — Source data Fig. 4 [file 44319_2026_783_MOESM11_ESM.zip › Figure4/4H/STREMI-Low exposure-raw.Tif]

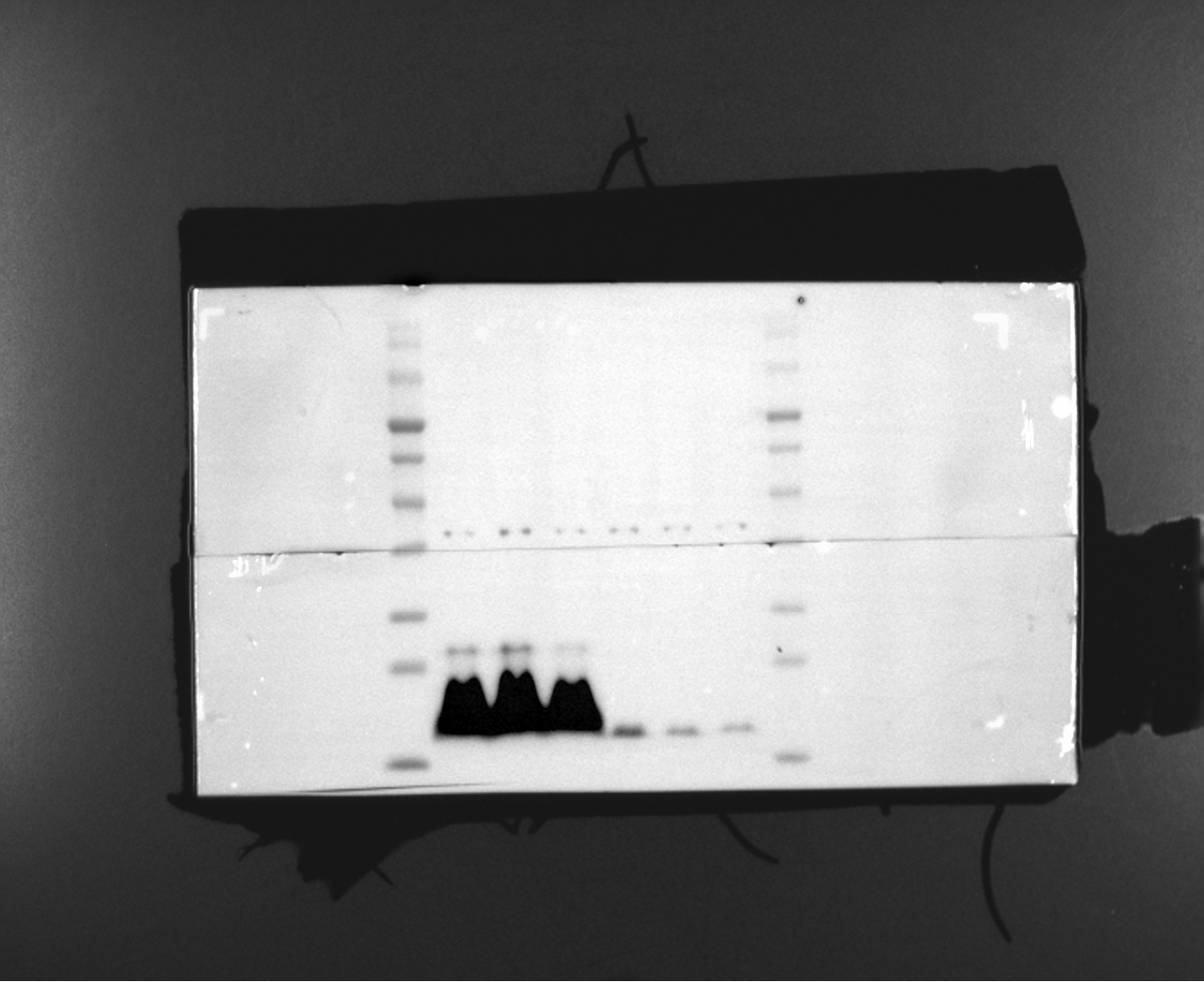

Supplement: Supplementary file 11 — Source data Fig. 4 [file 44319_2026_783_MOESM11_ESM.zip › Figure4/4H/STREMI-Merge.Tif]

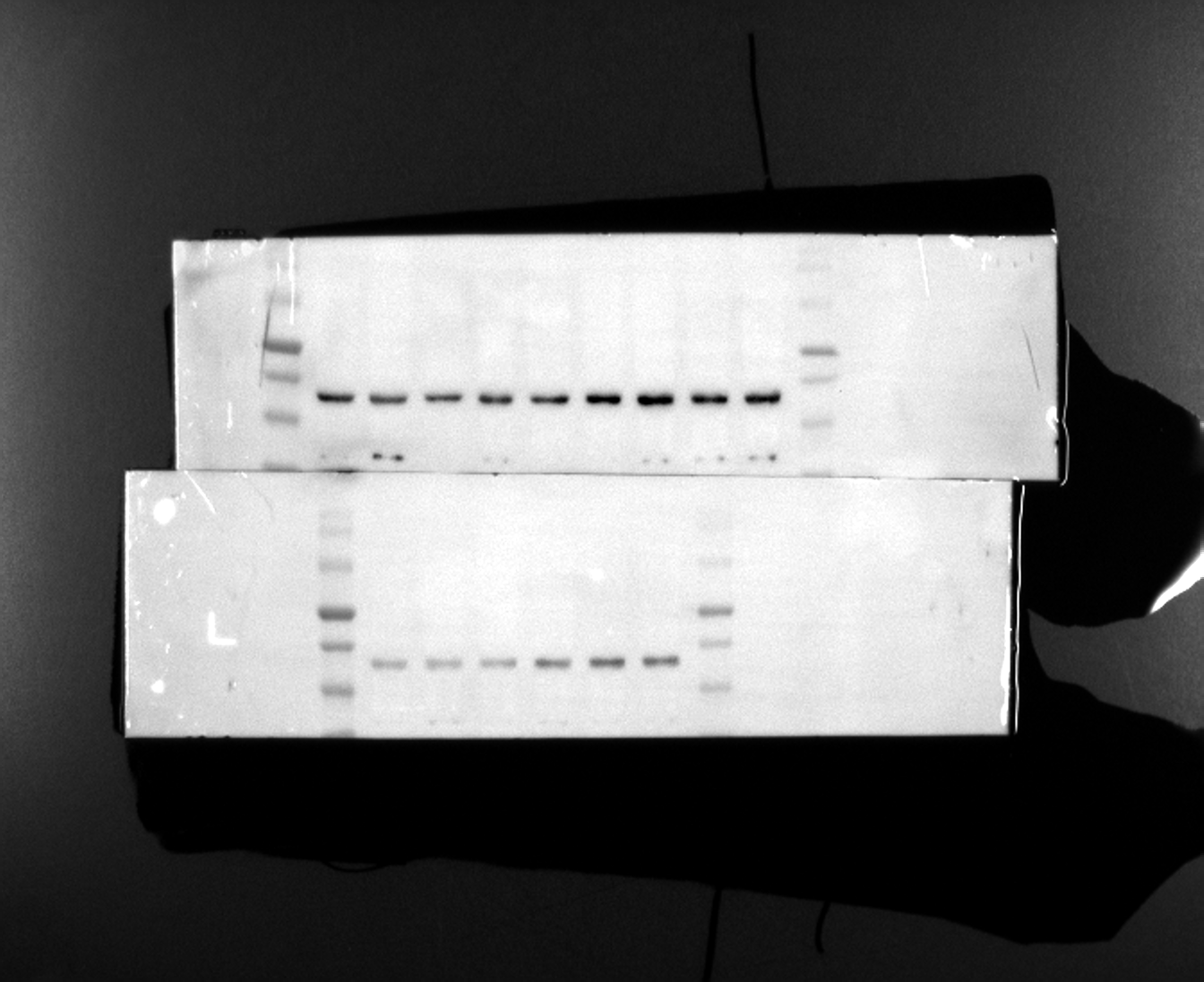

Supplement: Supplementary file 11 — Source data Fig. 4 [file 44319_2026_783_MOESM11_ESM.zip › Figure4/4H/TUBULIN-Merge.Tif]

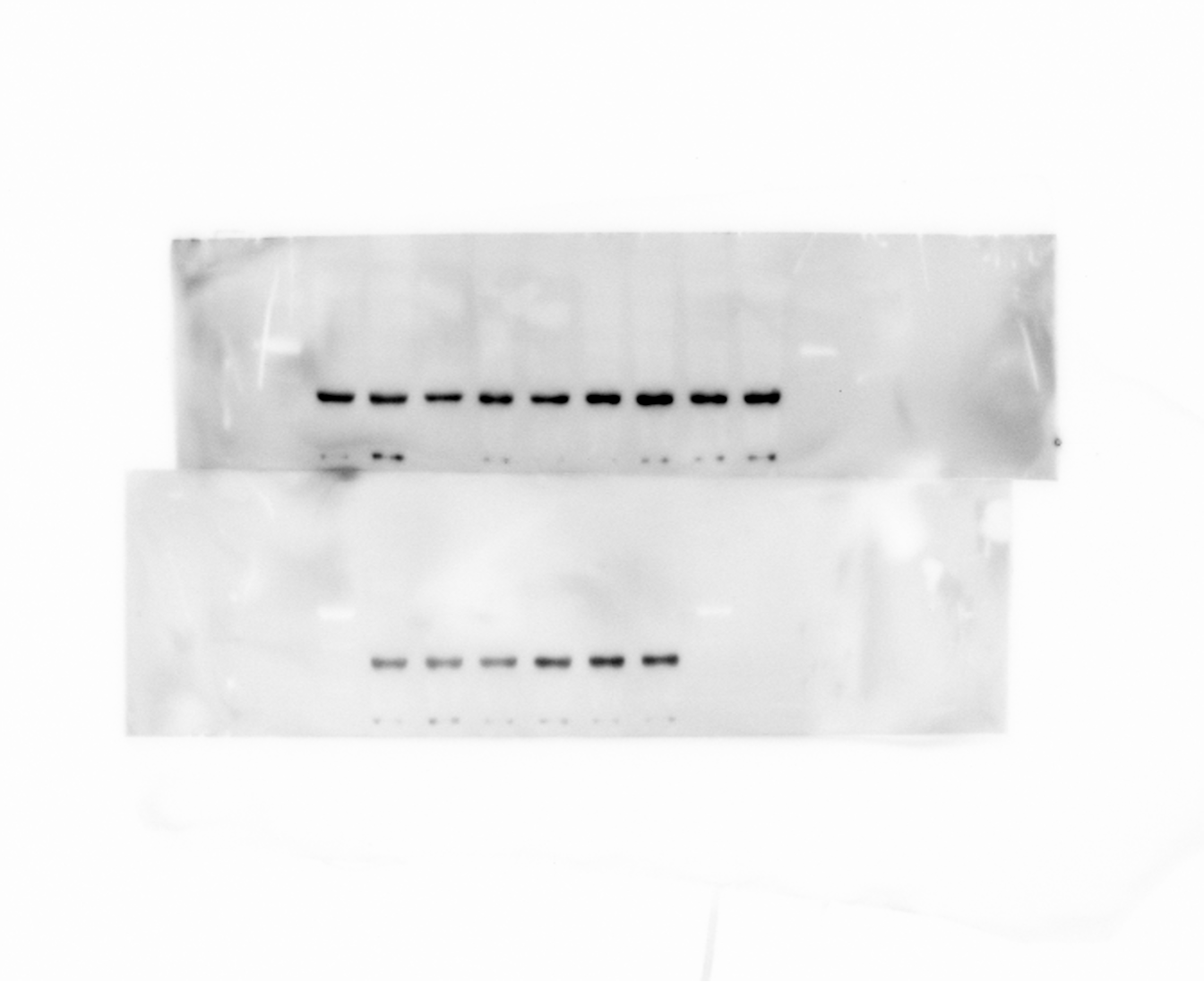

Supplement: Supplementary file 11 — Source data Fig. 4 [file 44319_2026_783_MOESM11_ESM.zip › Figure4/4H/TUBULIN-Raw.Tif]

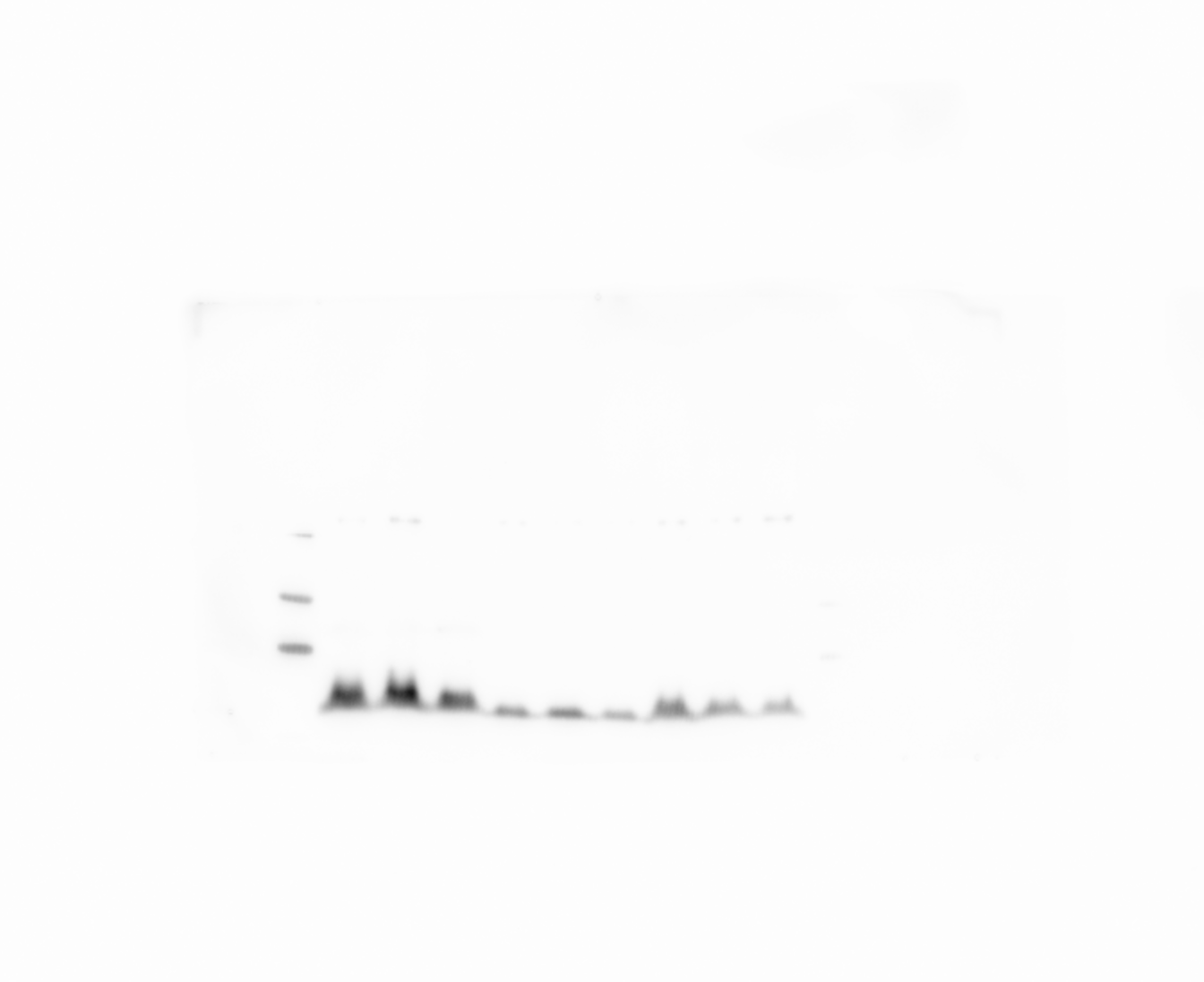

Supplement: Supplementary file 11 — Source data Fig. 4 [file 44319_2026_783_MOESM11_ESM.zip › Figure4/4J/StrepII-high exposure-Raw.Tif]

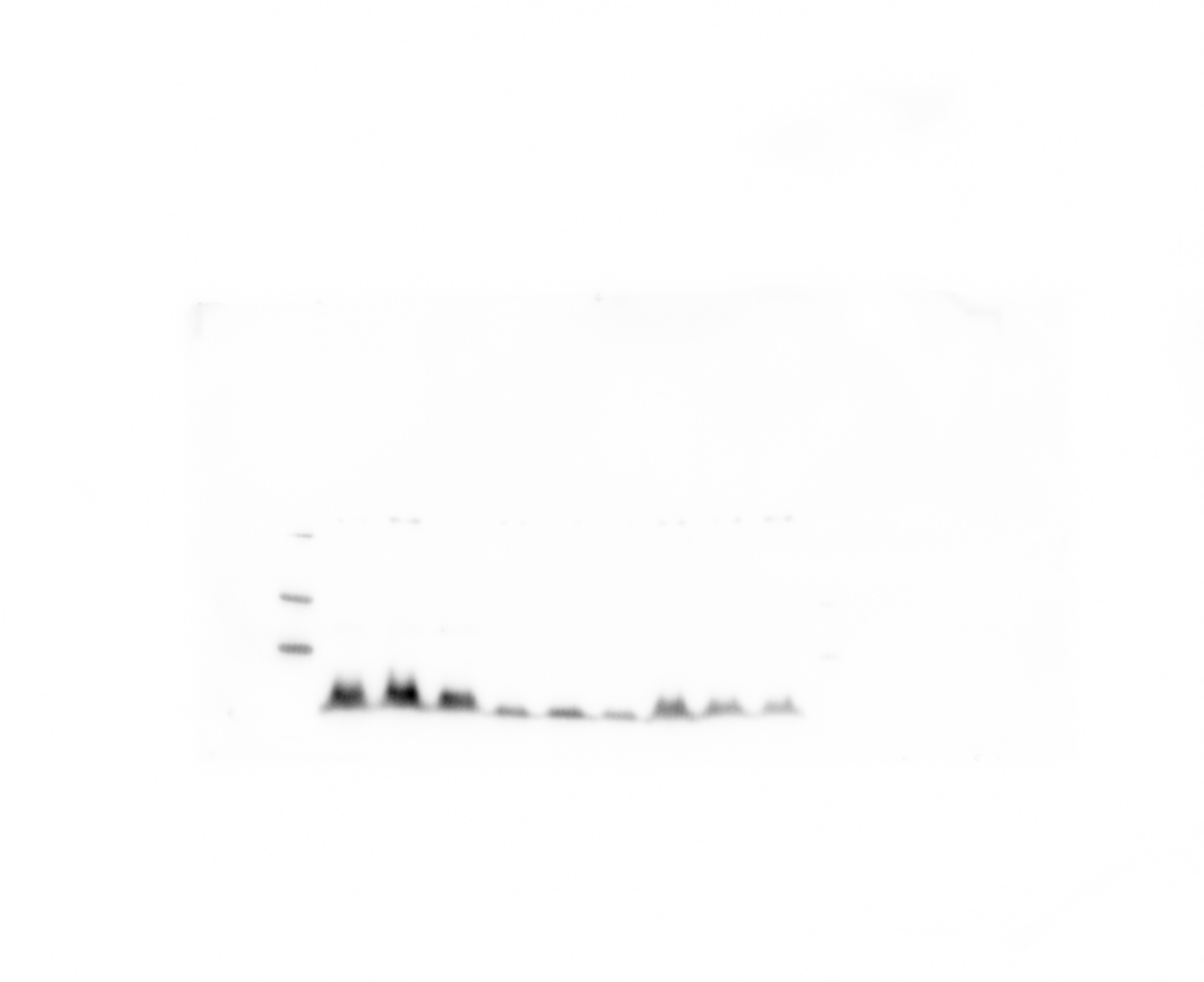

Supplement: Supplementary file 11 — Source data Fig. 4 [file 44319_2026_783_MOESM11_ESM.zip › Figure4/4J/StrepII-low exposure-Raw.Tif]

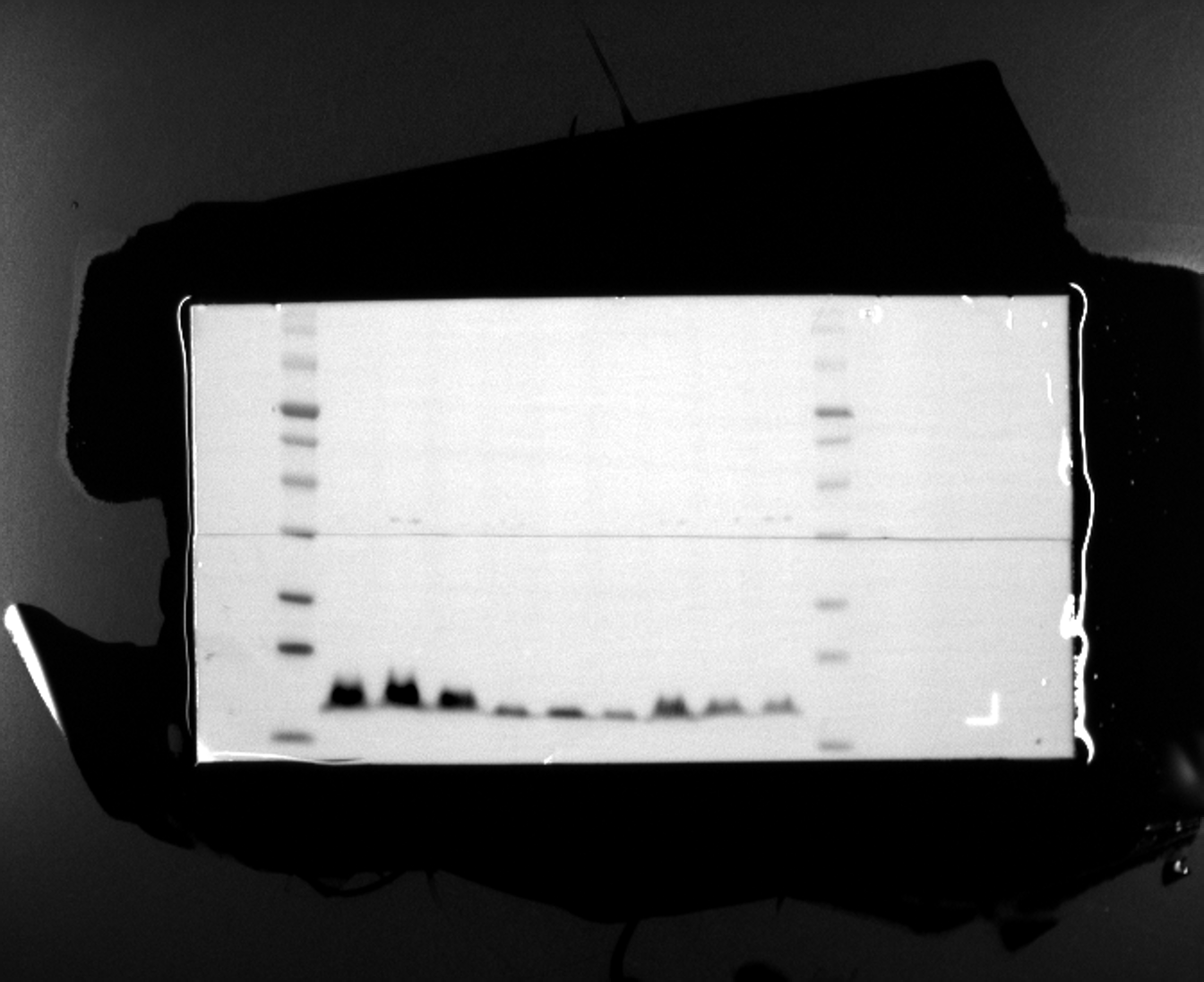

Supplement: Supplementary file 11 — Source data Fig. 4 [file 44319_2026_783_MOESM11_ESM.zip › Figure4/4J/StrepII-Merge.Tif]

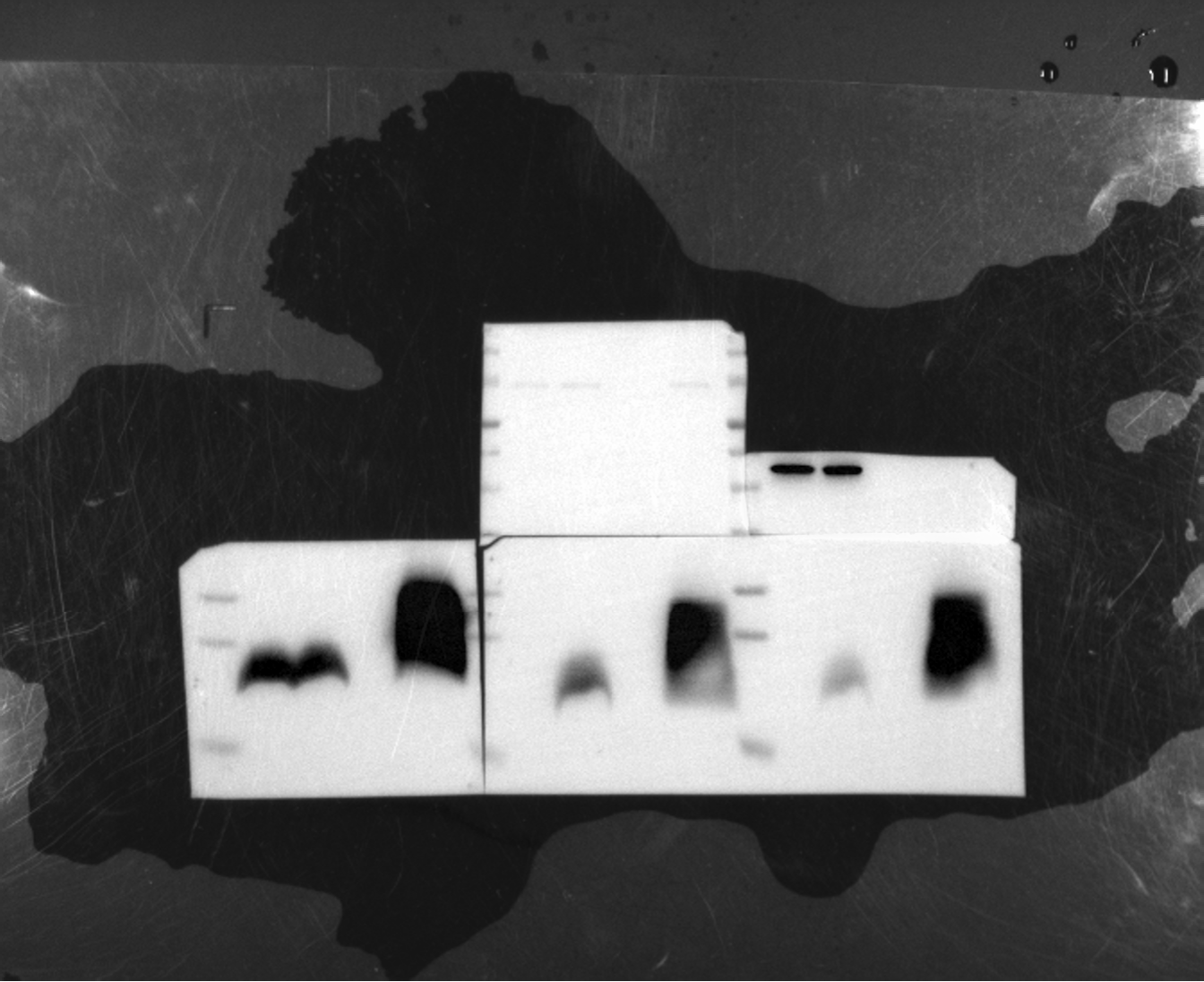

Supplement: Supplementary file 11 — Source data Fig. 4 [file 44319_2026_783_MOESM11_ESM.zip › Figure4/4L/Figure_4B-FLAG_Strep-Merge_data.Tif]

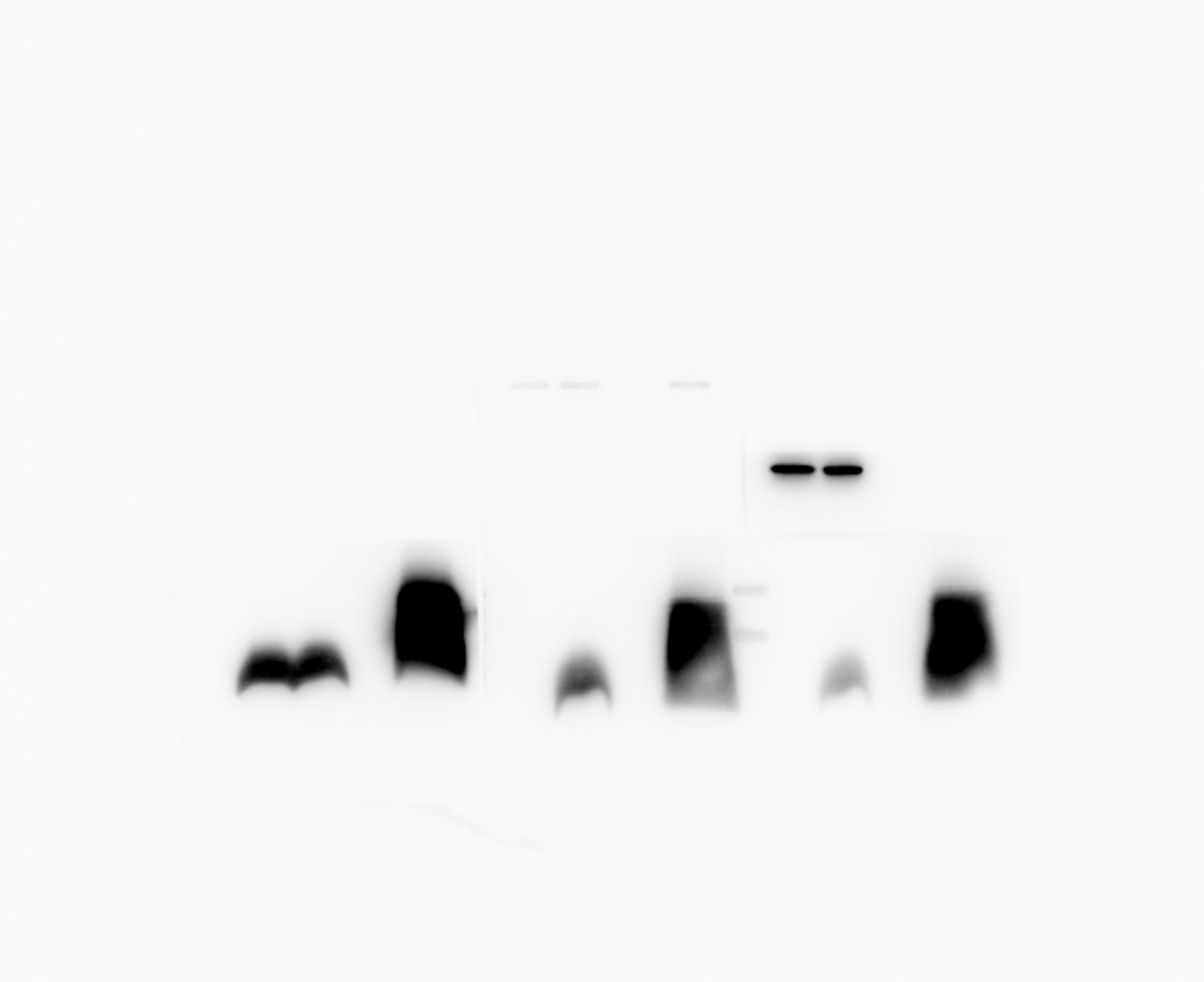

Supplement: Supplementary file 11 — Source data Fig. 4 [file 44319_2026_783_MOESM11_ESM.zip › Figure4/4L/Figure_4B-FLAG_Strep-Raw_data.Tif]

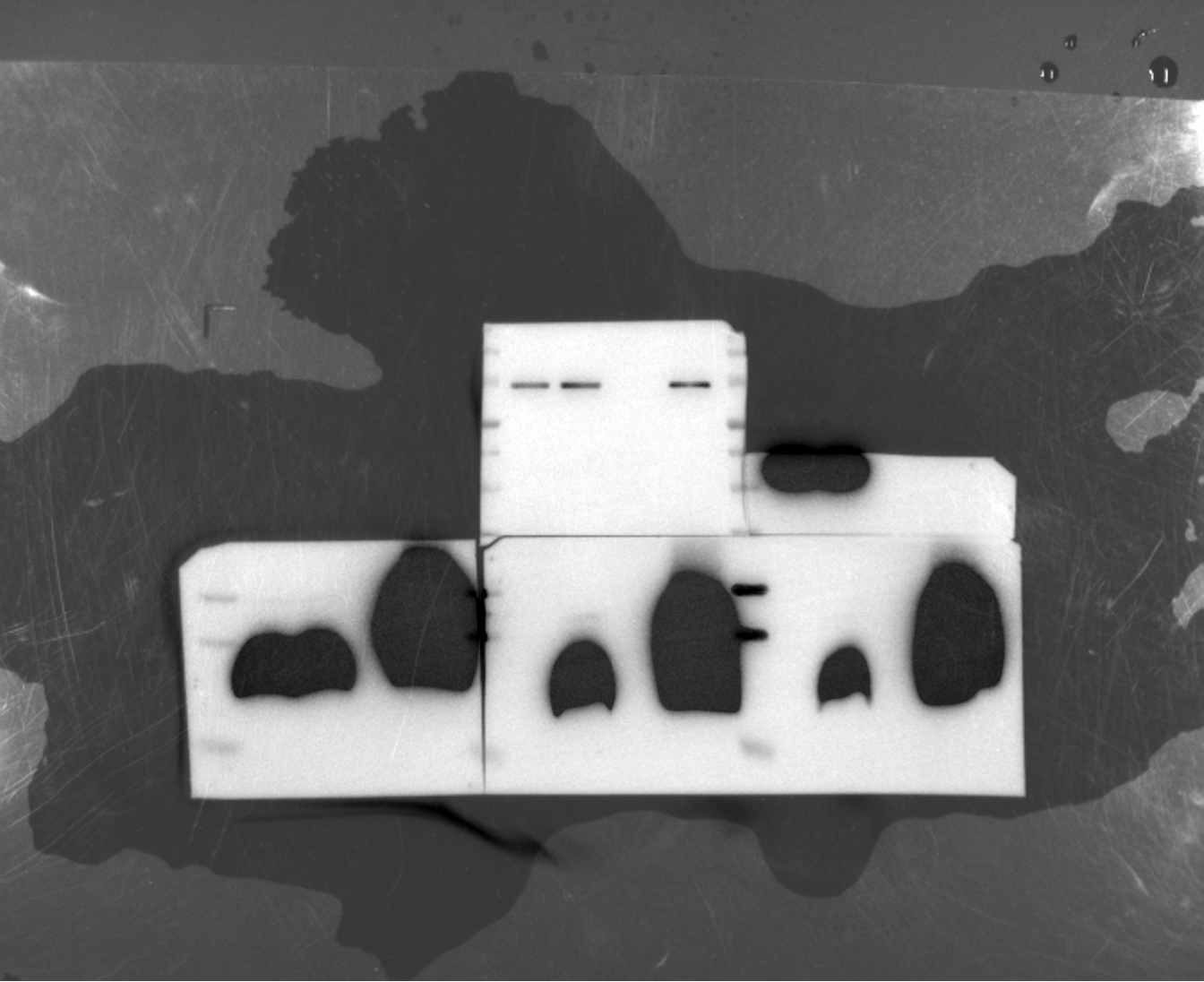

Supplement: Supplementary file 11 — Source data Fig. 4 [file 44319_2026_783_MOESM11_ESM.zip › Figure4/4L/Figure_4B-MIC60-Merge_data.tif]

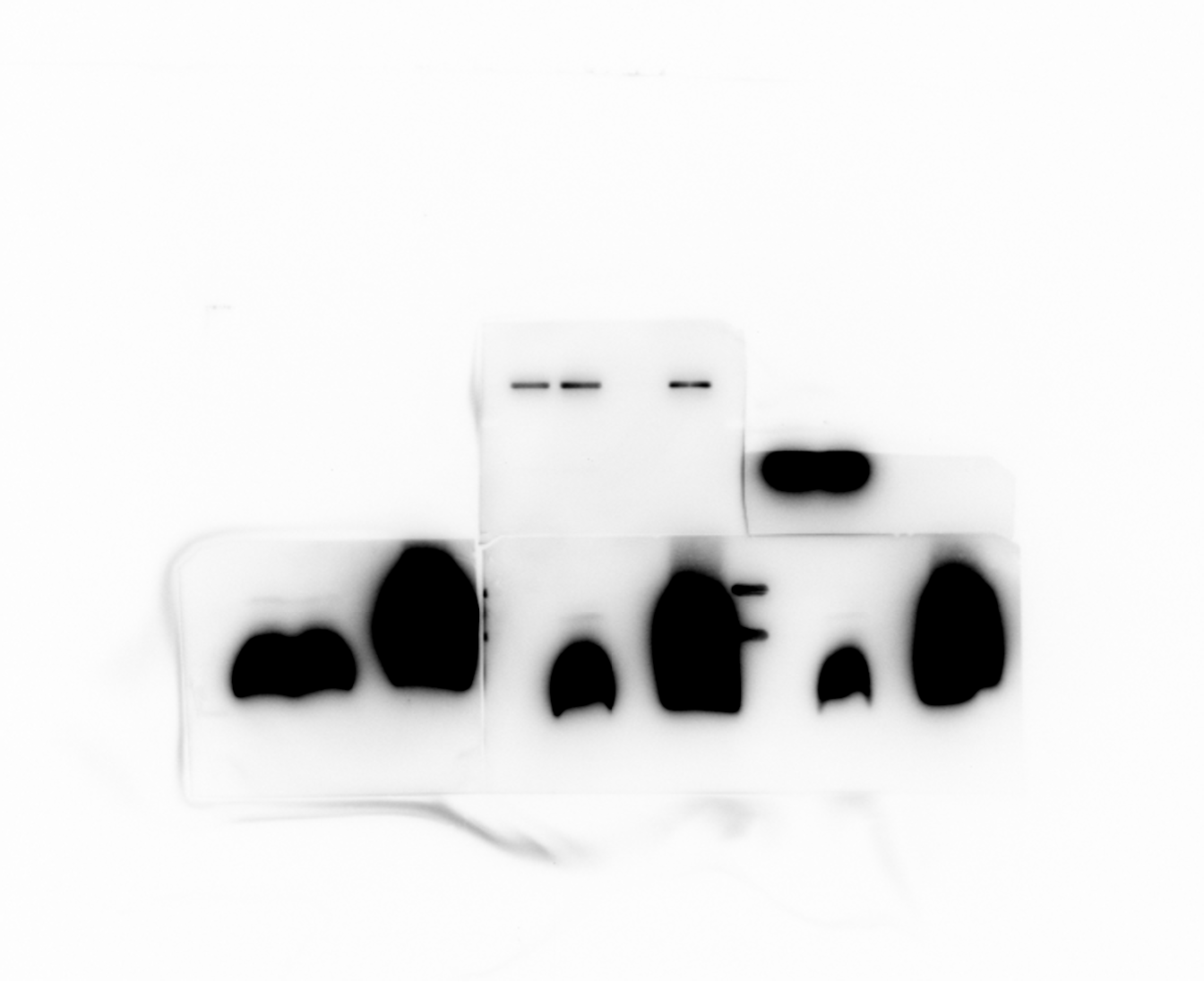

Supplement: Supplementary file 11 — Source data Fig. 4 [file 44319_2026_783_MOESM11_ESM.zip › Figure4/4L/Figure_4B-MIC60-Raw_data.tif]

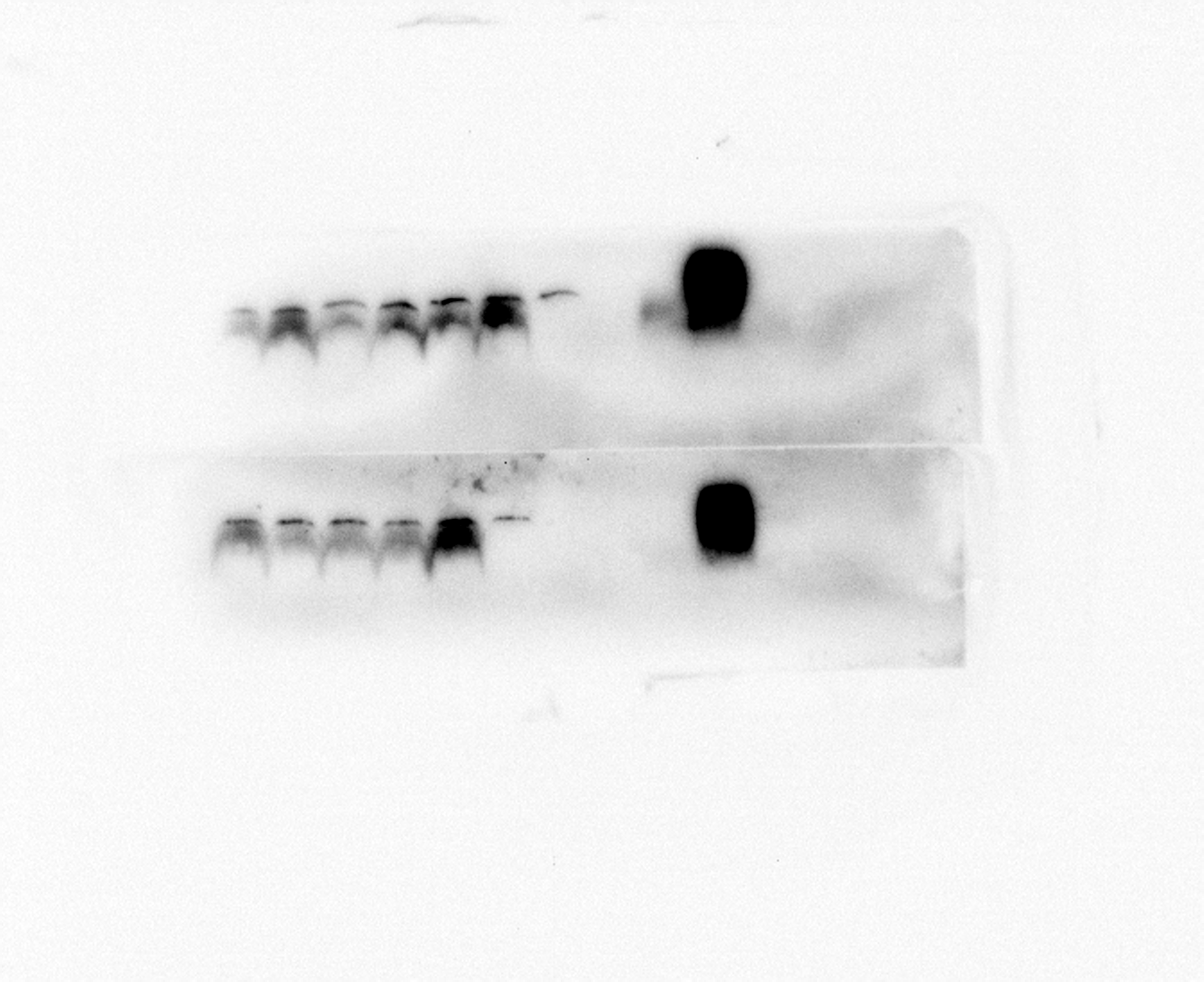

Supplement: Supplementary file 11 — Source data Fig. 4 [file 44319_2026_783_MOESM11_ESM.zip › Figure4/4M/Figure_4M- STREMI-Raw_data.Tif]

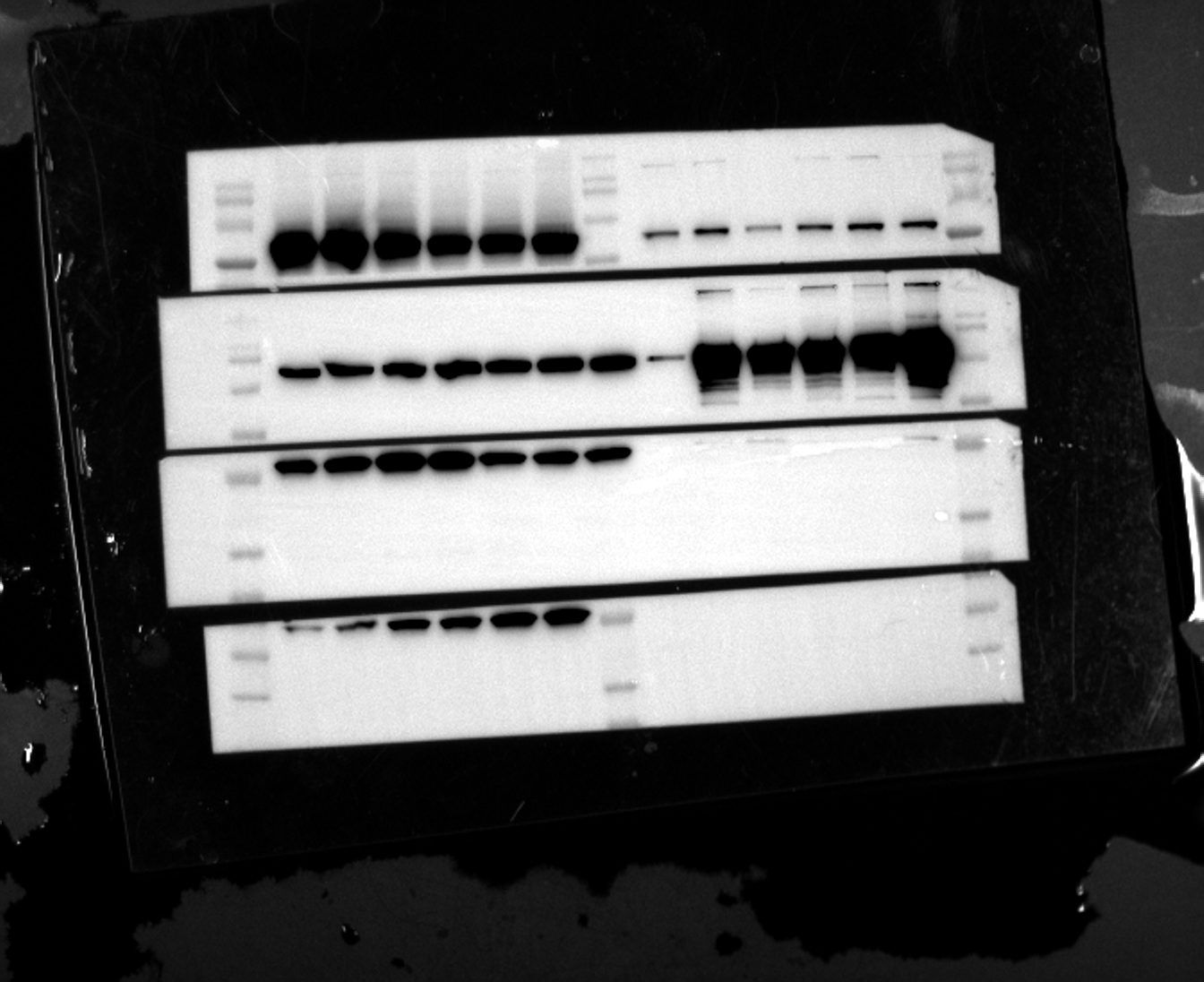

Supplement: Supplementary file 11 — Source data Fig. 4 [file 44319_2026_783_MOESM11_ESM.zip › Figure4/4M/Figure_4M- UQCRC1-Merge_data.Tif]

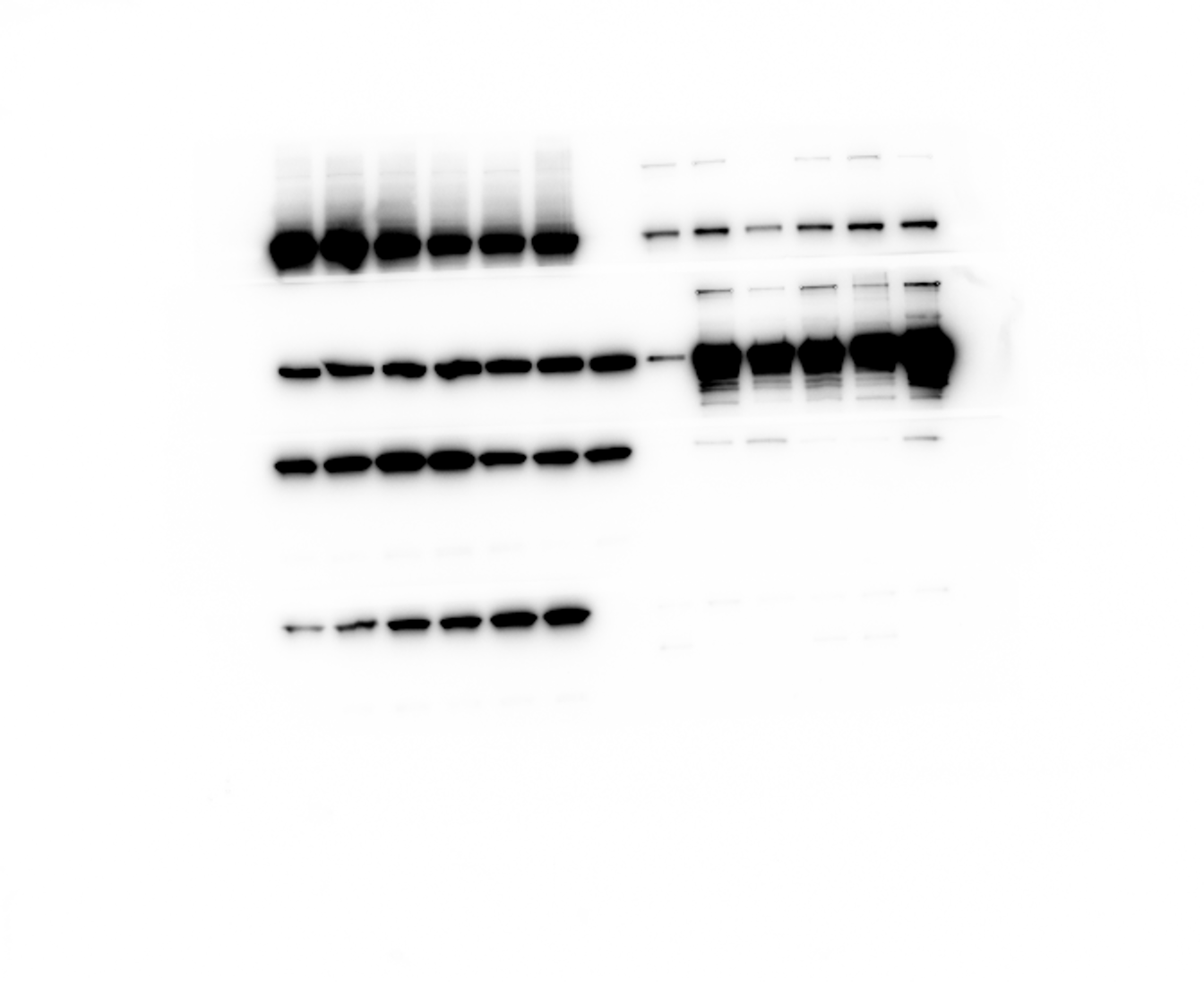

Supplement: Supplementary file 11 — Source data Fig. 4 [file 44319_2026_783_MOESM11_ESM.zip › Figure4/4M/Figure_4M- UQCRC1-Raw_data.Tif]

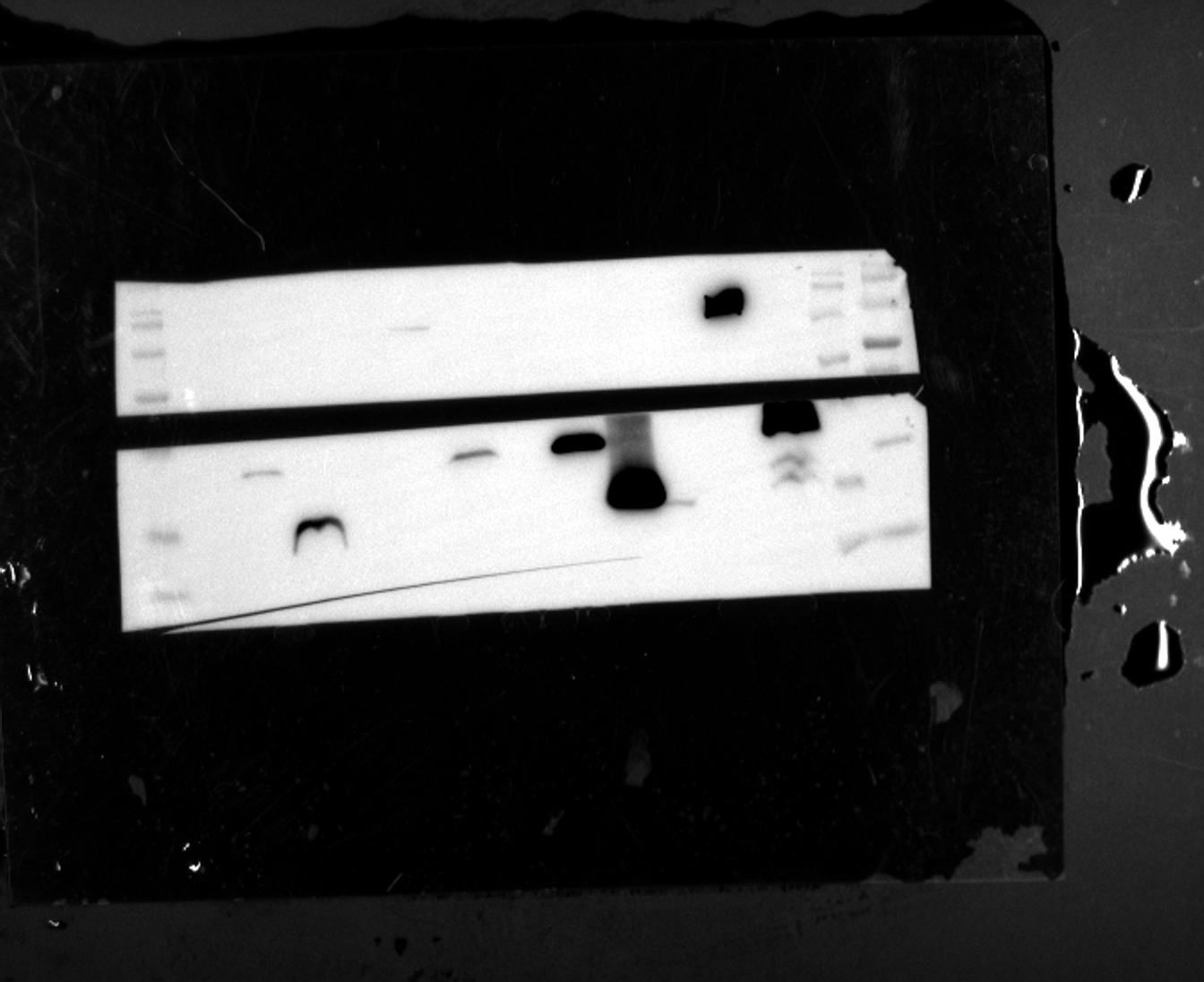

Supplement: Supplementary file 11 — Source data Fig. 4 [file 44319_2026_783_MOESM11_ESM.zip › Figure4/4M/Figure_4M- _FLAG-Merge_data.Tif]

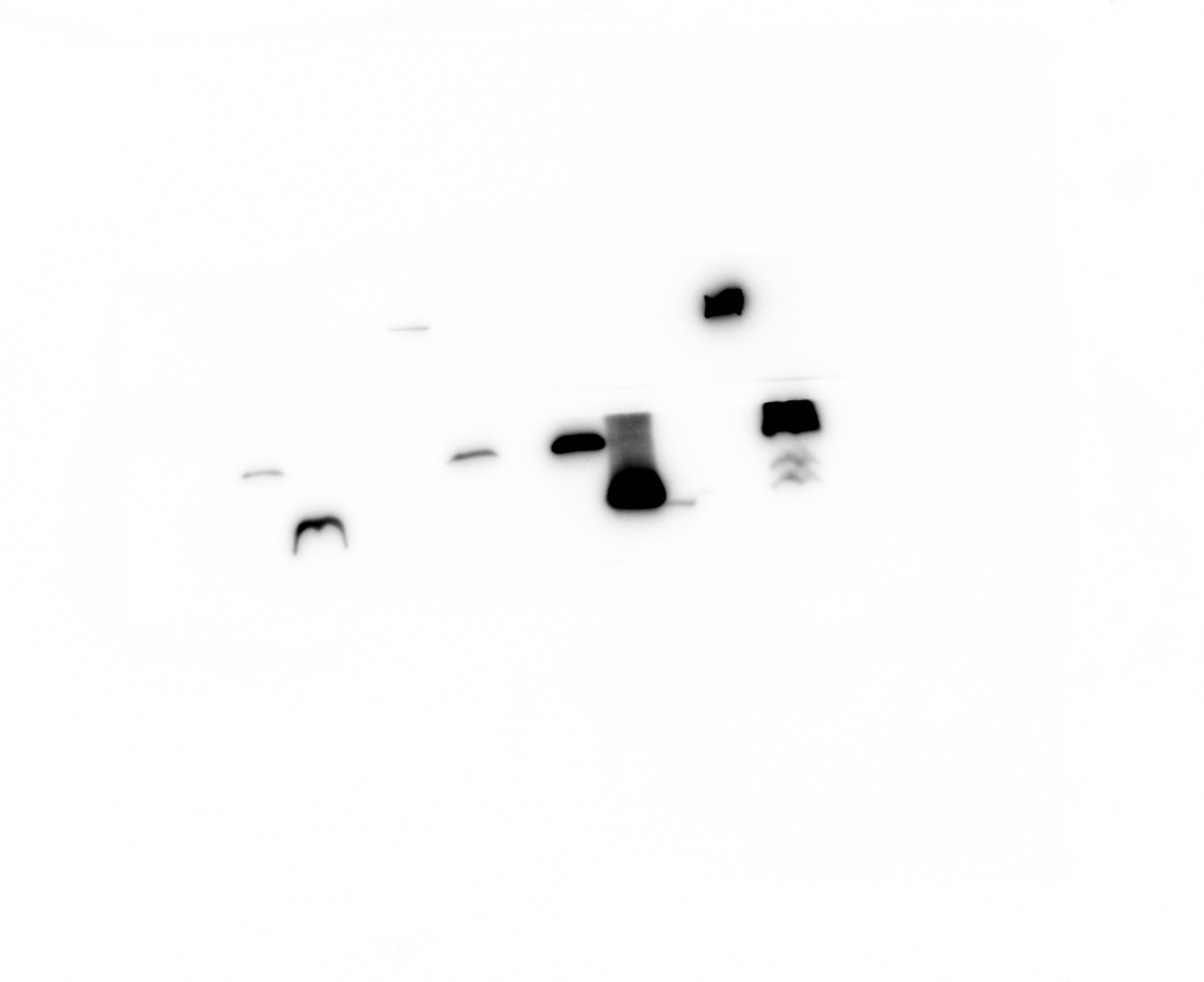

Supplement: Supplementary file 11 — Source data Fig. 4 [file 44319_2026_783_MOESM11_ESM.zip › Figure4/4M/Figure_4M- _FLAG-Raw_data.Tif]

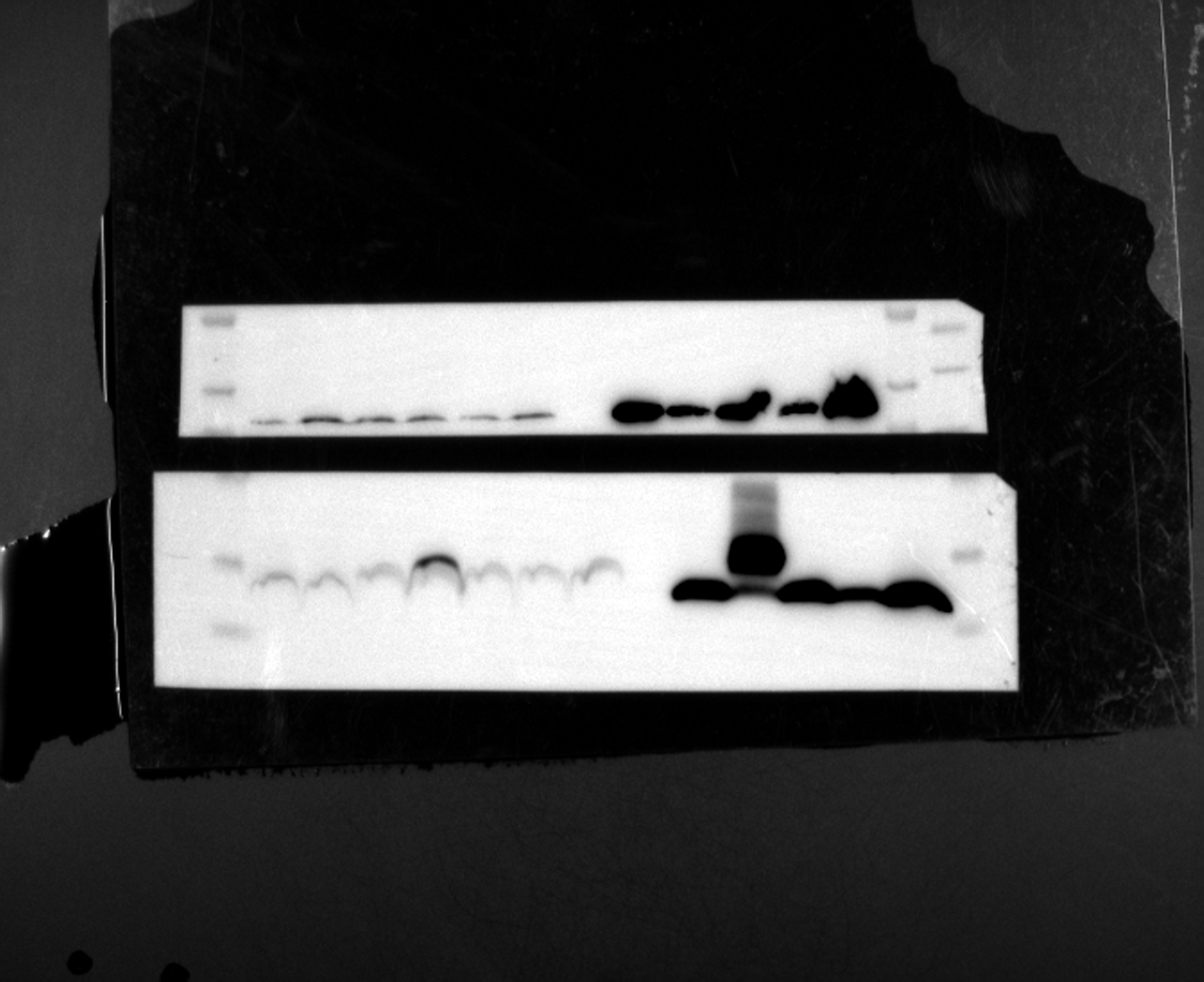

Supplement: Supplementary file 11 — Source data Fig. 4 [file 44319_2026_783_MOESM11_ESM.zip › Figure4/4M/Figure_4M- _MIC10-Merge_data.Tif]

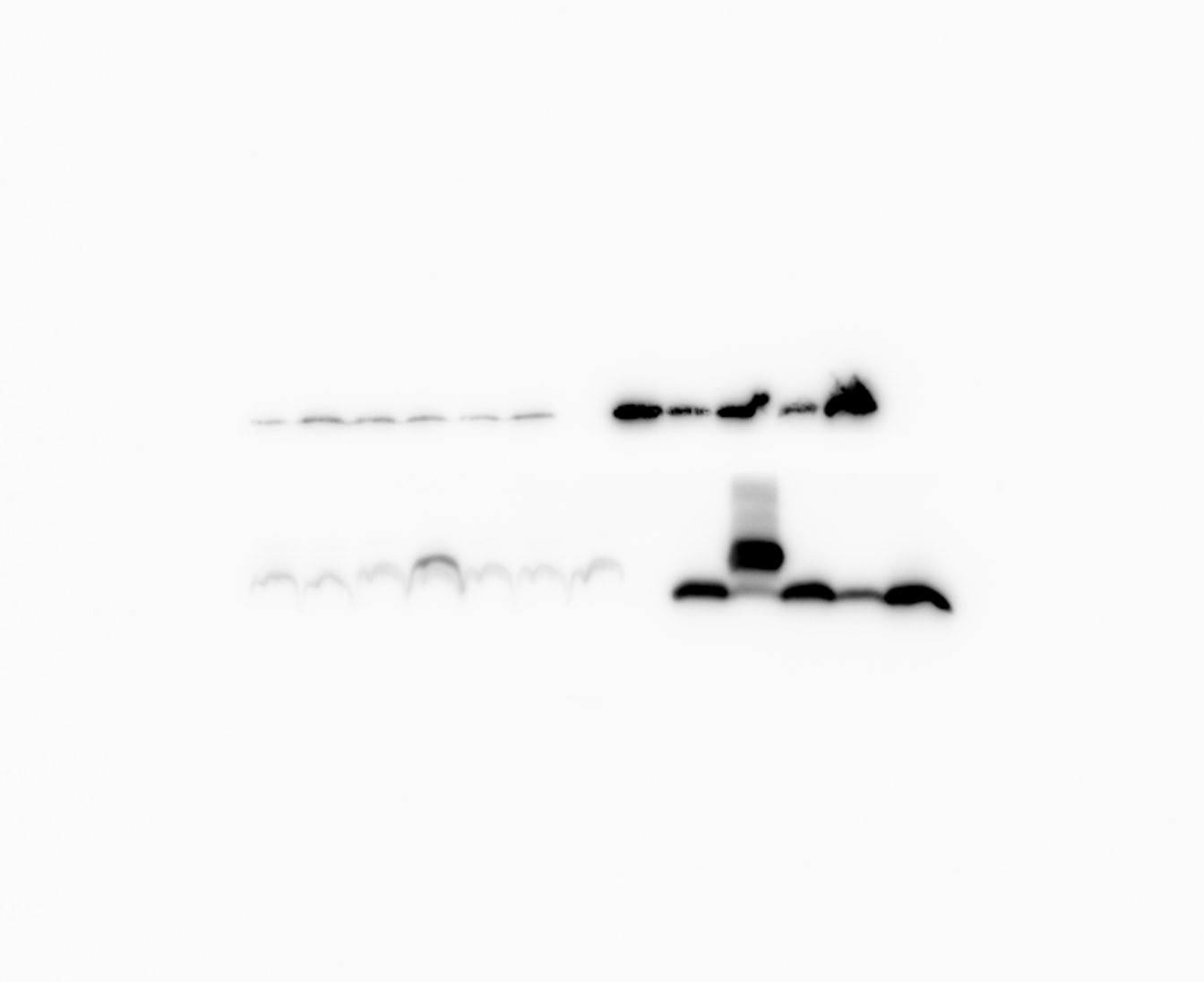

Supplement: Supplementary file 11 — Source data Fig. 4 [file 44319_2026_783_MOESM11_ESM.zip › Figure4/4M/Figure_4M- _MIC10-Raw_data.Tif]

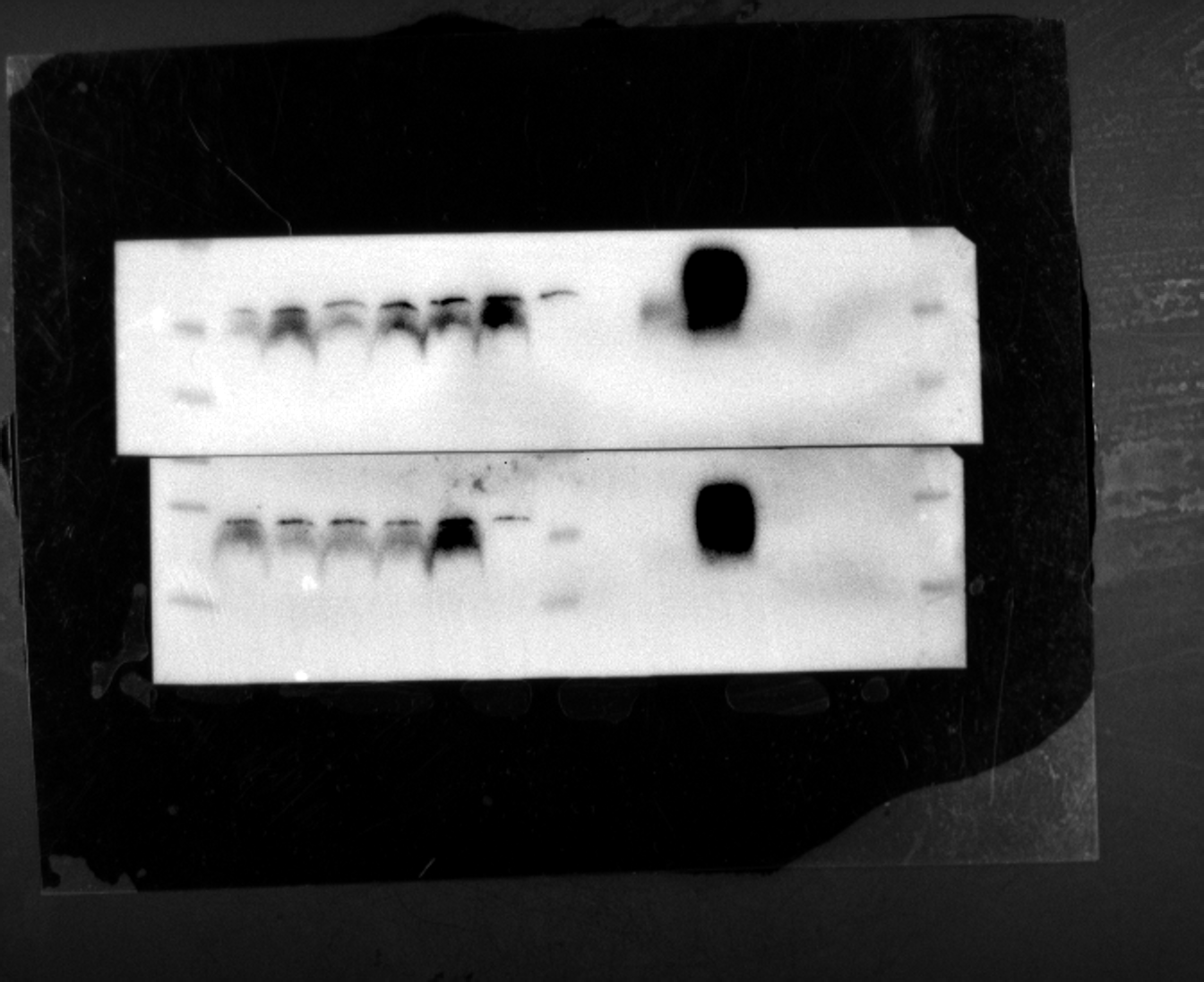

Supplement: Supplementary file 11 — Source data Fig. 4 [file 44319_2026_783_MOESM11_ESM.zip › Figure4/4M/Figure_4M- STREMI-Merge_data.Tif]

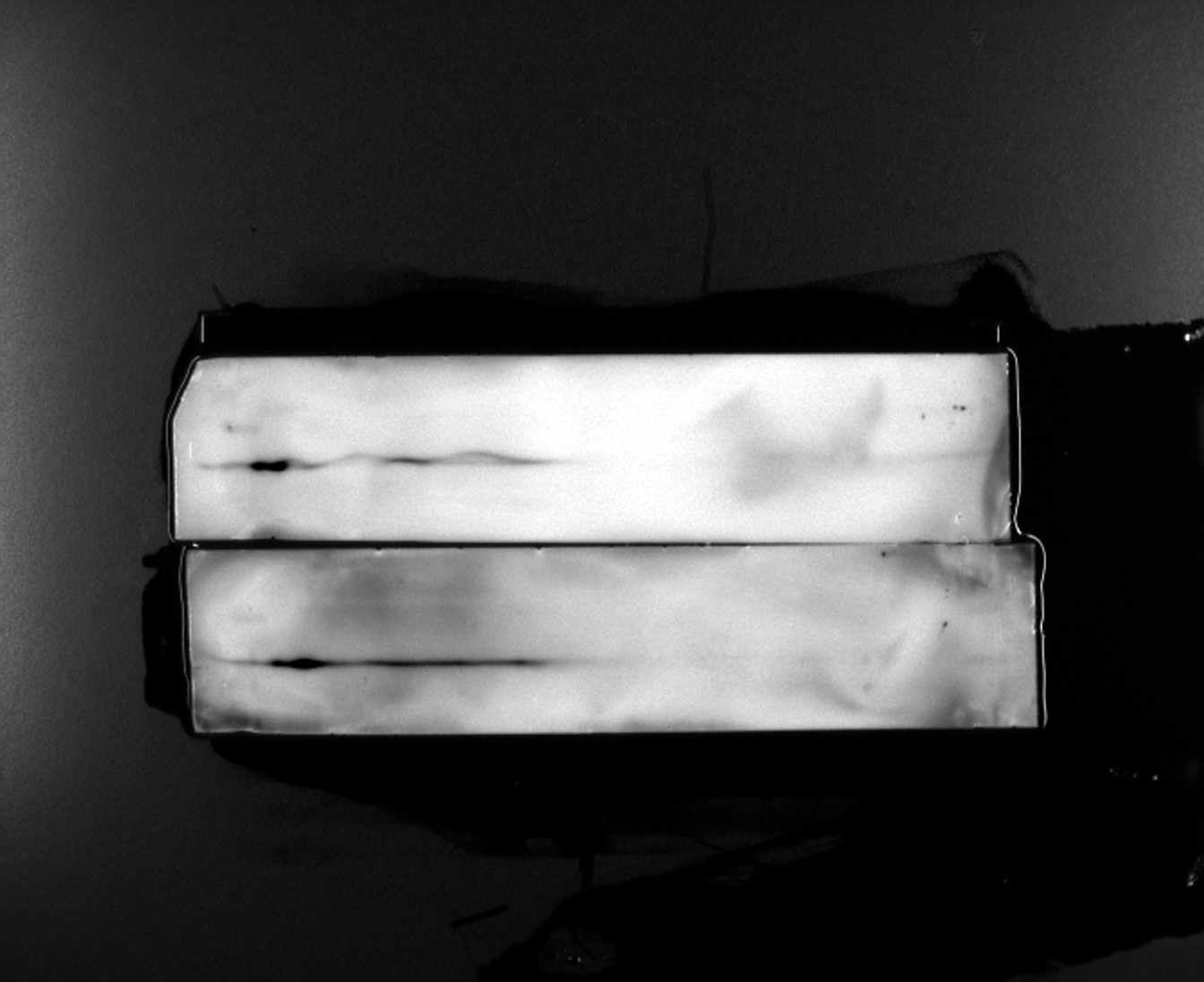

Supplement: Supplementary file 11 — Source data Fig. 4 [file 44319_2026_783_MOESM11_ESM.zip › Figure4/4N/MIC10-MERGE.Tif]

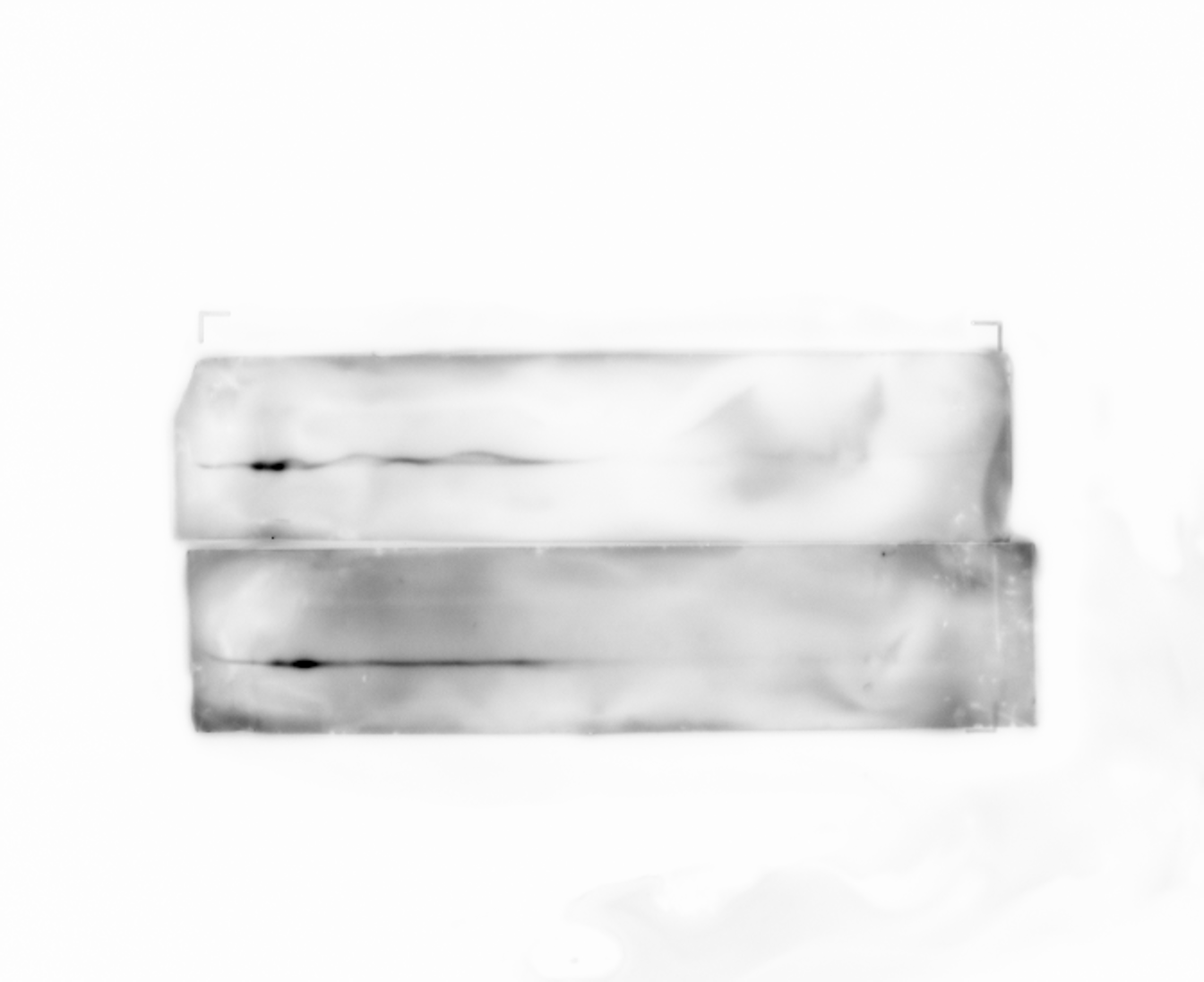

Supplement: Supplementary file 11 — Source data Fig. 4 [file 44319_2026_783_MOESM11_ESM.zip › Figure4/4N/MIC10-Raw.Tif]

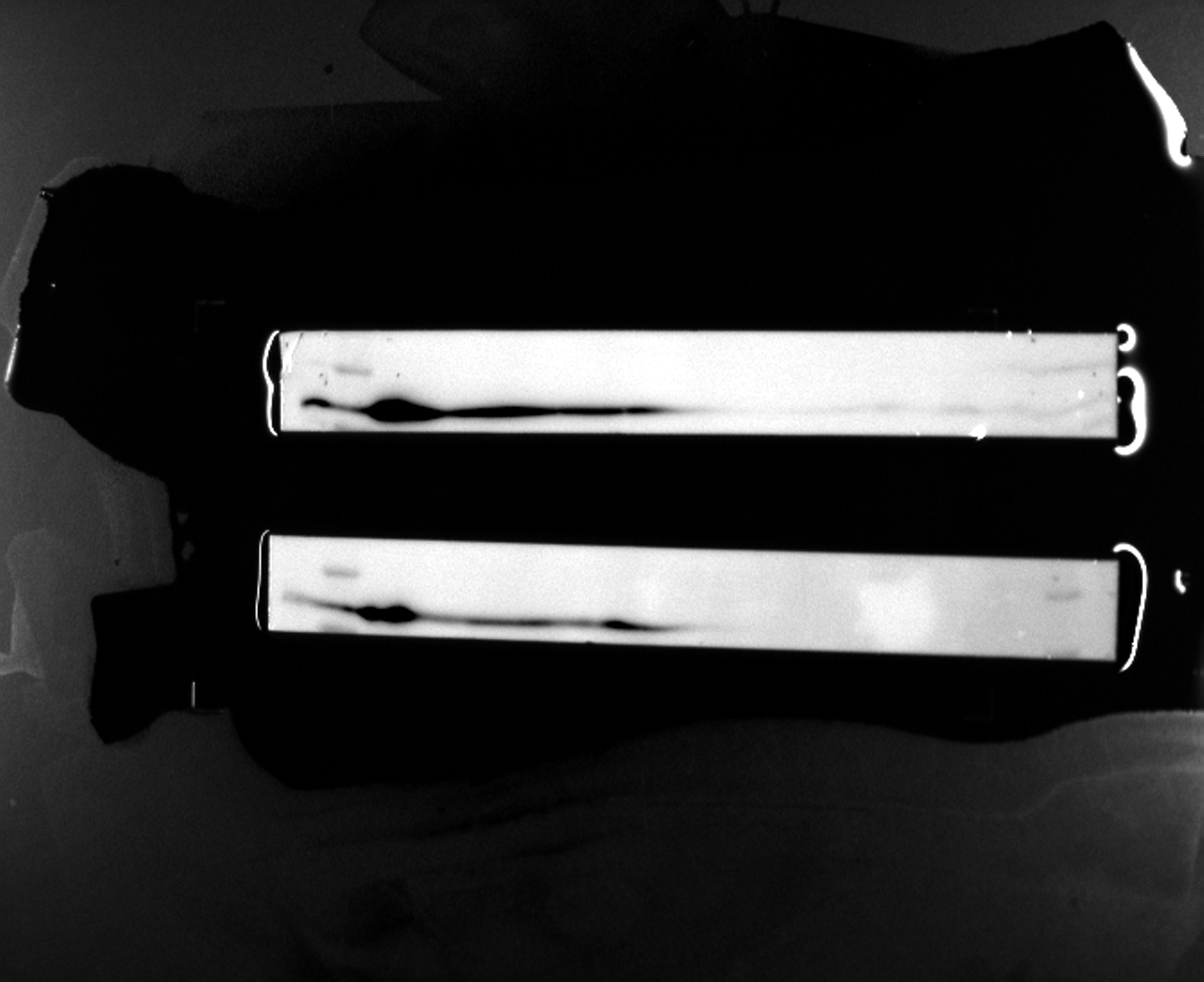

Supplement: Supplementary file 11 — Source data Fig. 4 [file 44319_2026_783_MOESM11_ESM.zip › Figure4/4N/MIC19-Merge.Tif]

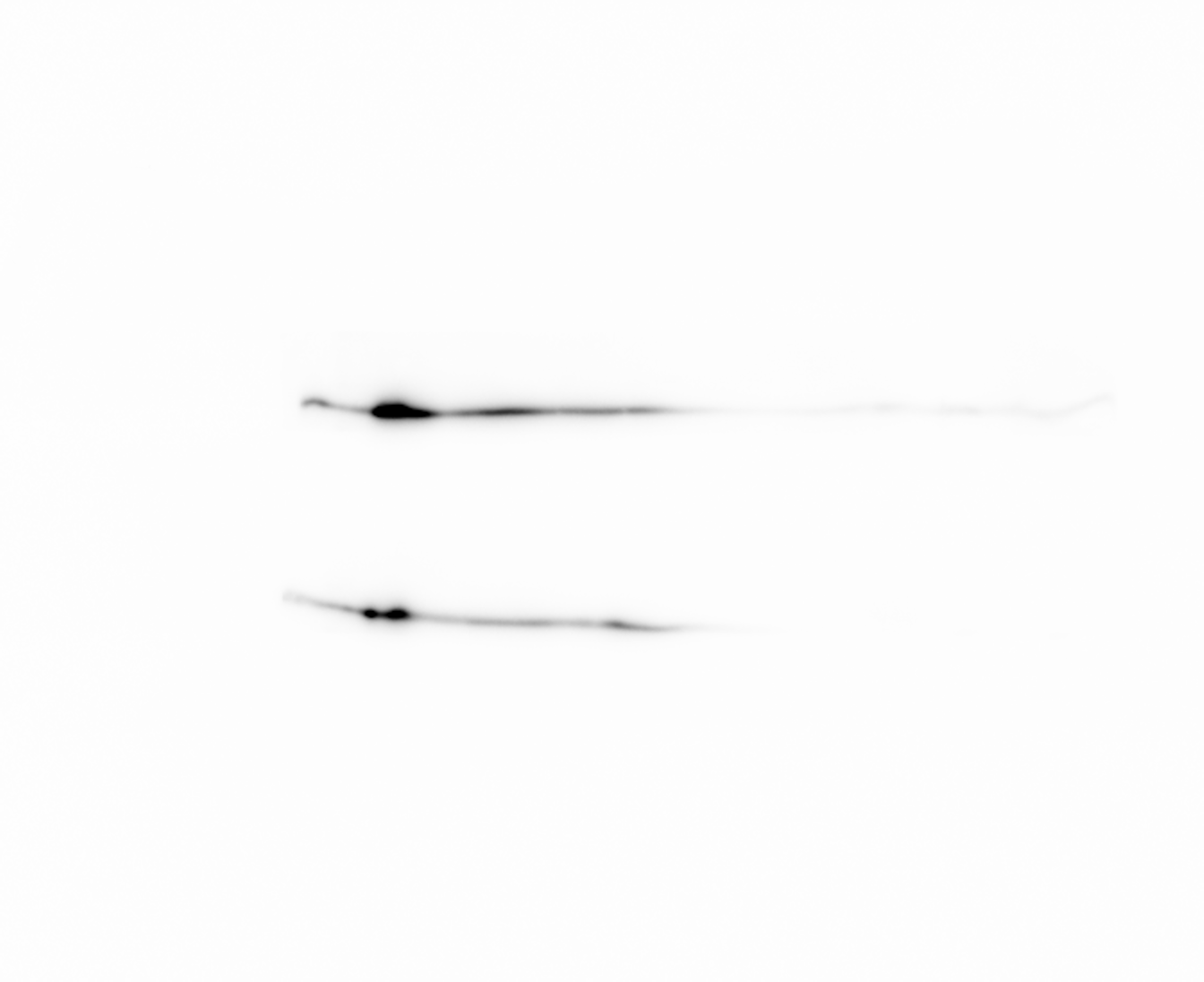

Supplement: Supplementary file 11 — Source data Fig. 4 [file 44319_2026_783_MOESM11_ESM.zip › Figure4/4N/MIC19-Raw.Tif]

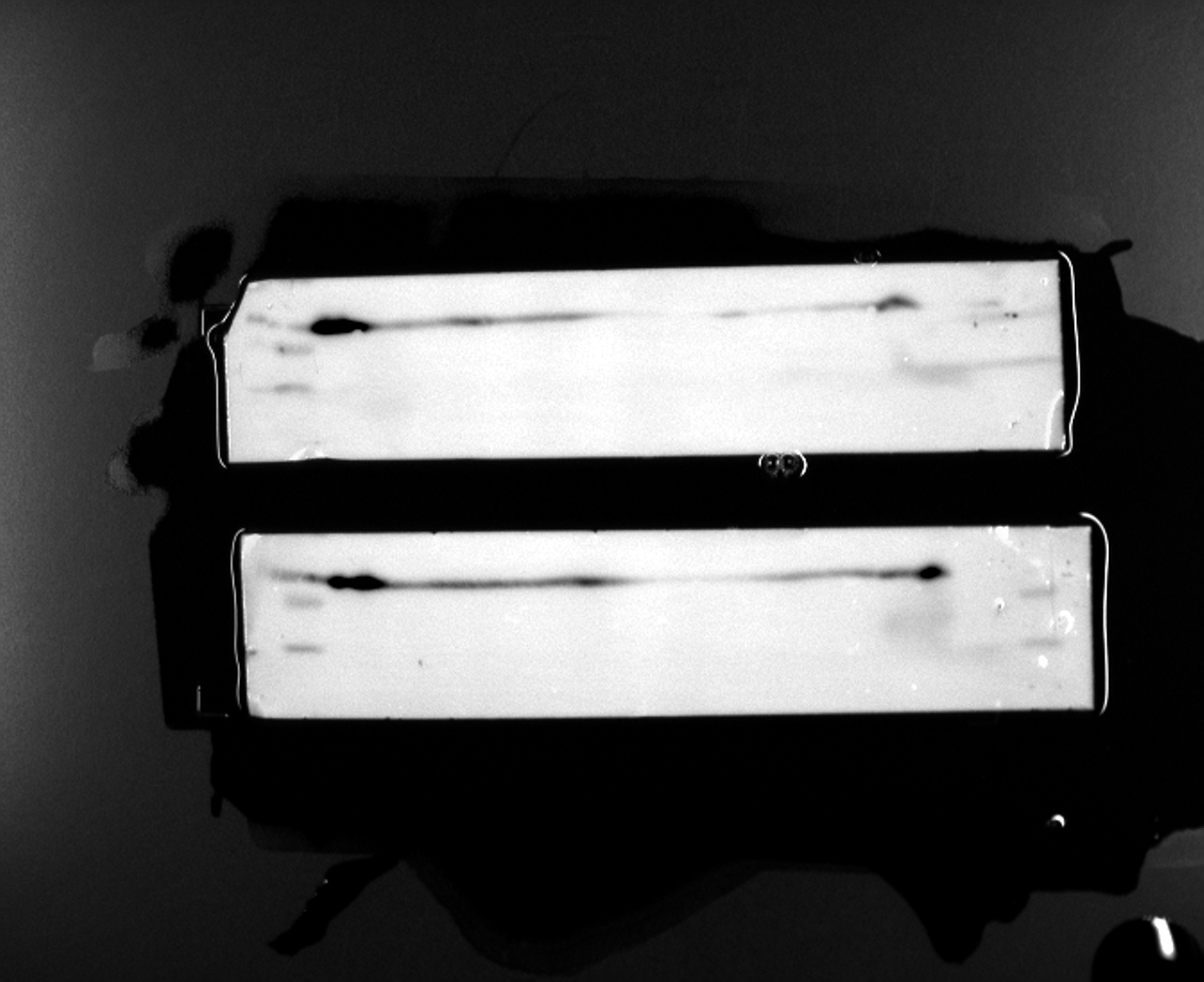

Supplement: Supplementary file 11 — Source data Fig. 4 [file 44319_2026_783_MOESM11_ESM.zip › Figure4/4N/MIC26-MERGE.Tif]

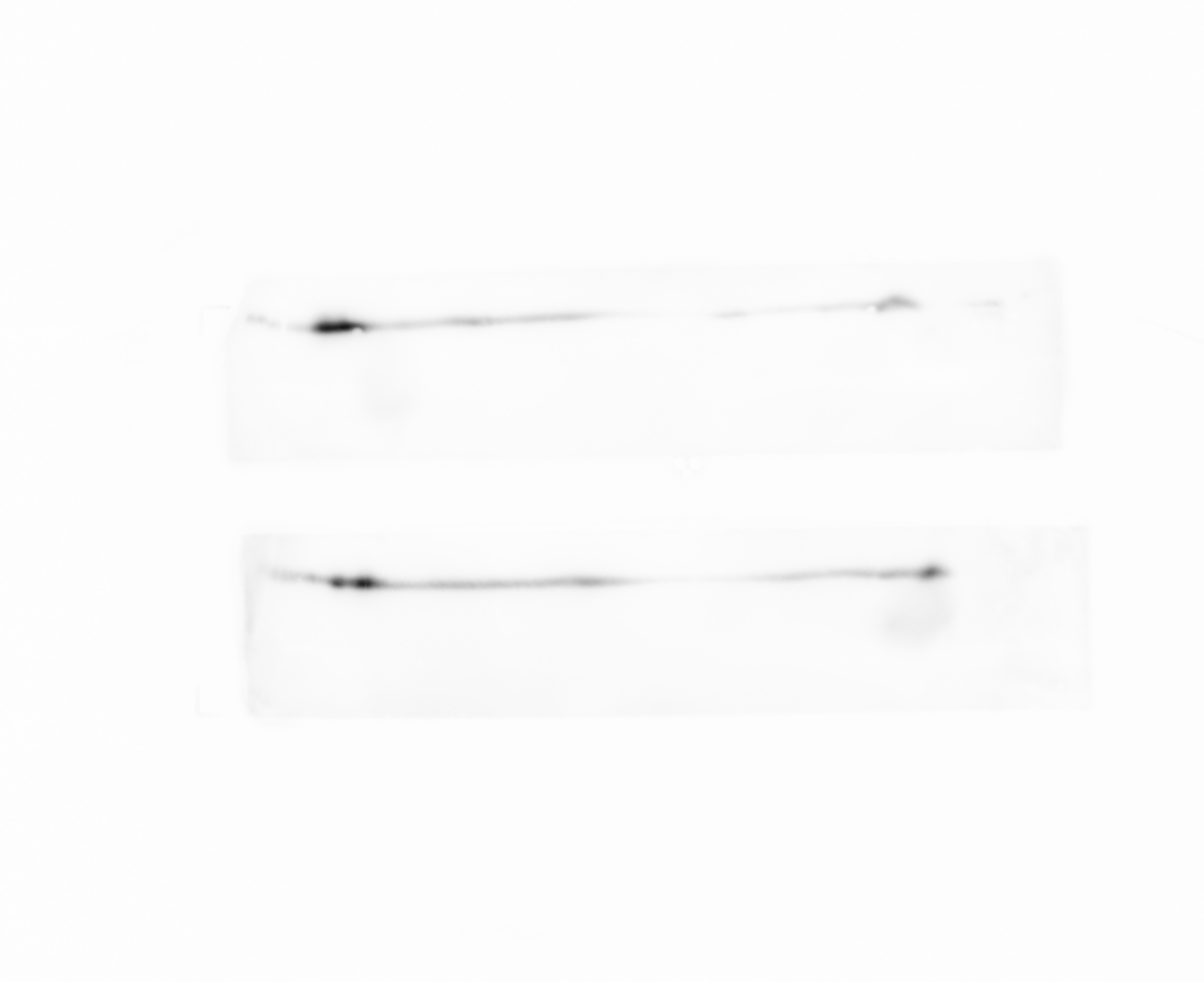

Supplement: Supplementary file 11 — Source data Fig. 4 [file 44319_2026_783_MOESM11_ESM.zip › Figure4/4N/MIC26-Raw.Tif]

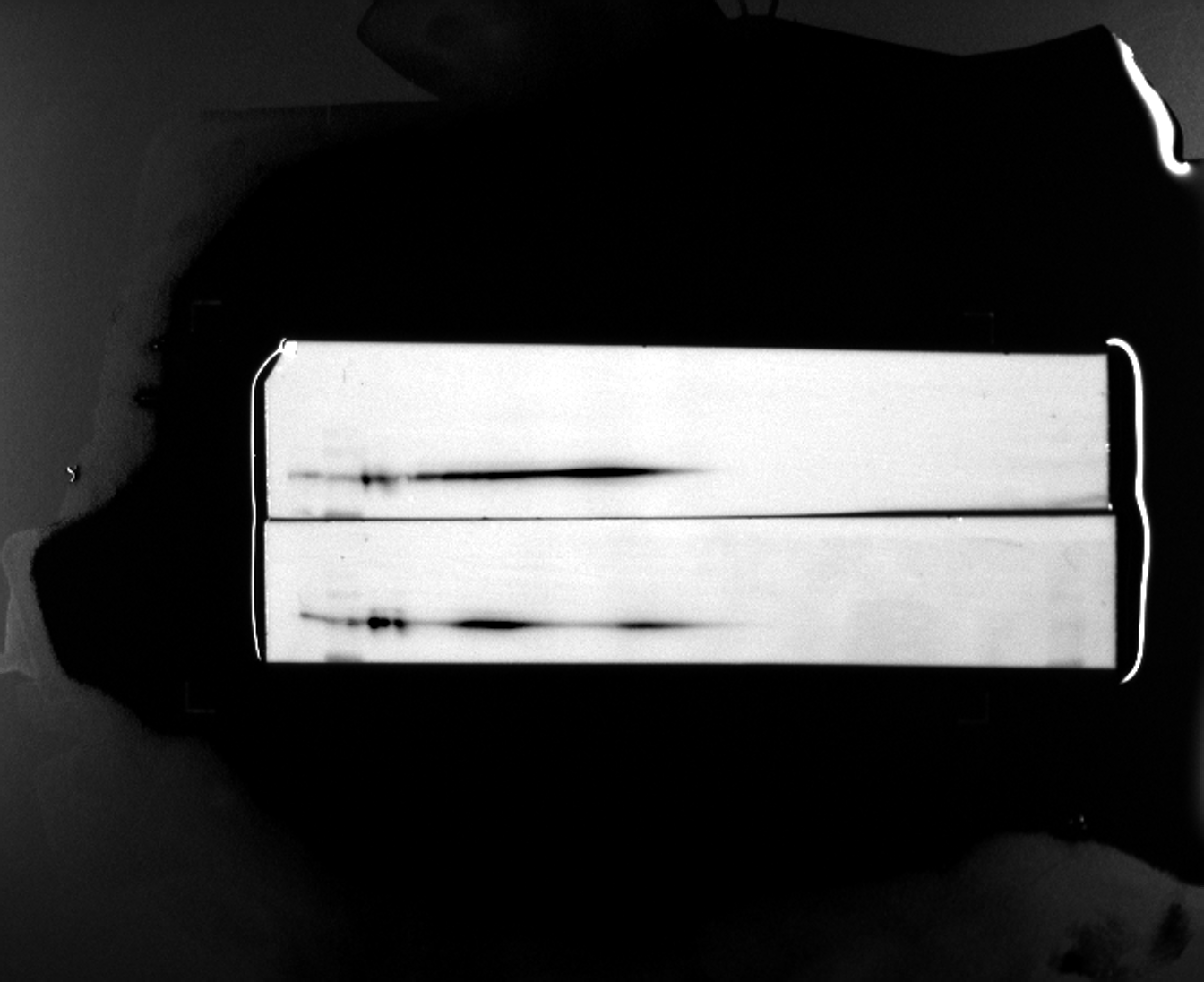

Supplement: Supplementary file 11 — Source data Fig. 4 [file 44319_2026_783_MOESM11_ESM.zip › Figure4/4N/MIC60-MERGE.Tif]

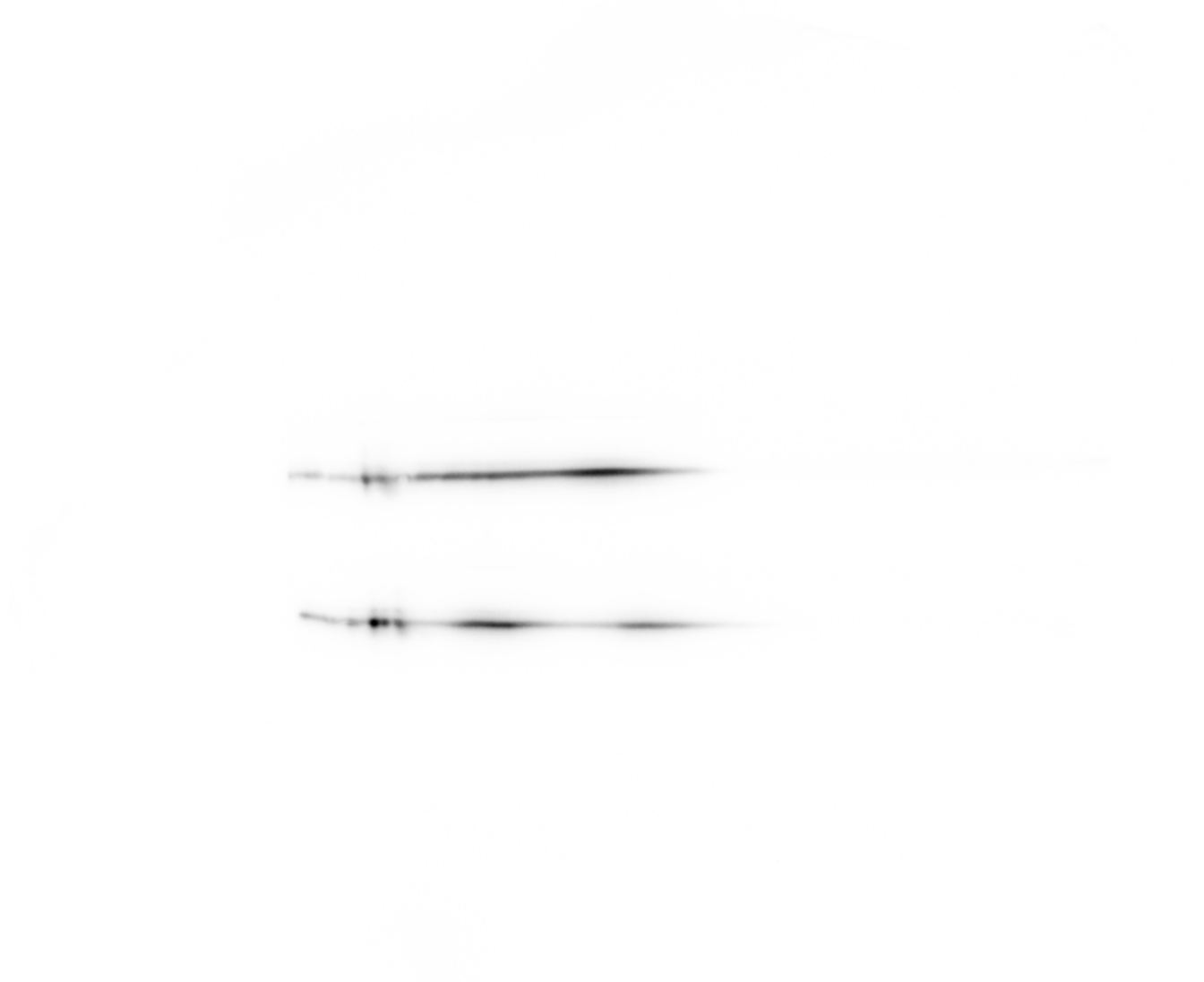

Supplement: Supplementary file 11 — Source data Fig. 4 [file 44319_2026_783_MOESM11_ESM.zip › Figure4/4N/MIC60-Raw.Tif]

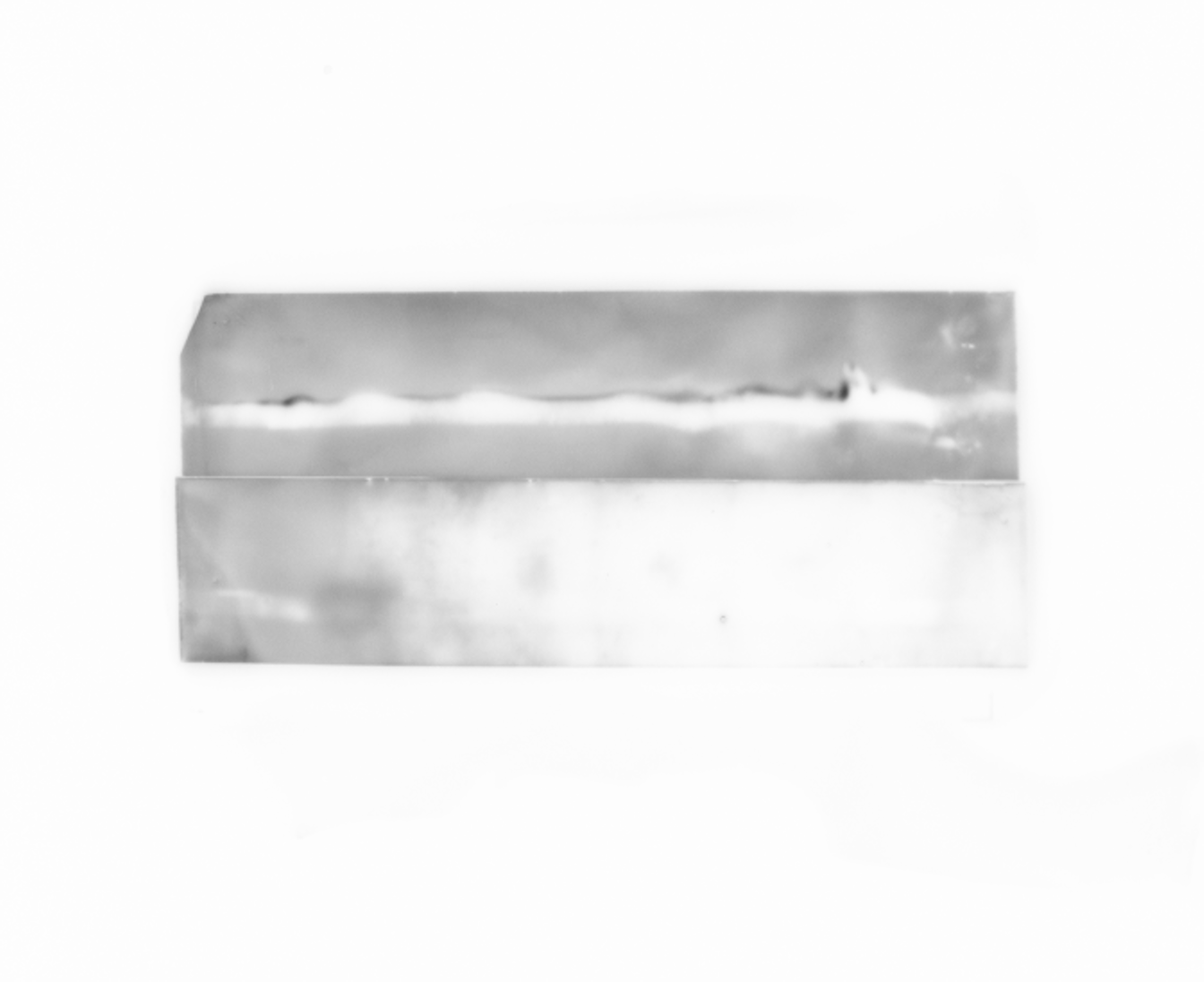

Supplement: Supplementary file 11 — Source data Fig. 4 [file 44319_2026_783_MOESM11_ESM.zip › Figure4/4N/STREMI-WT-KO-high exposure.Tif]

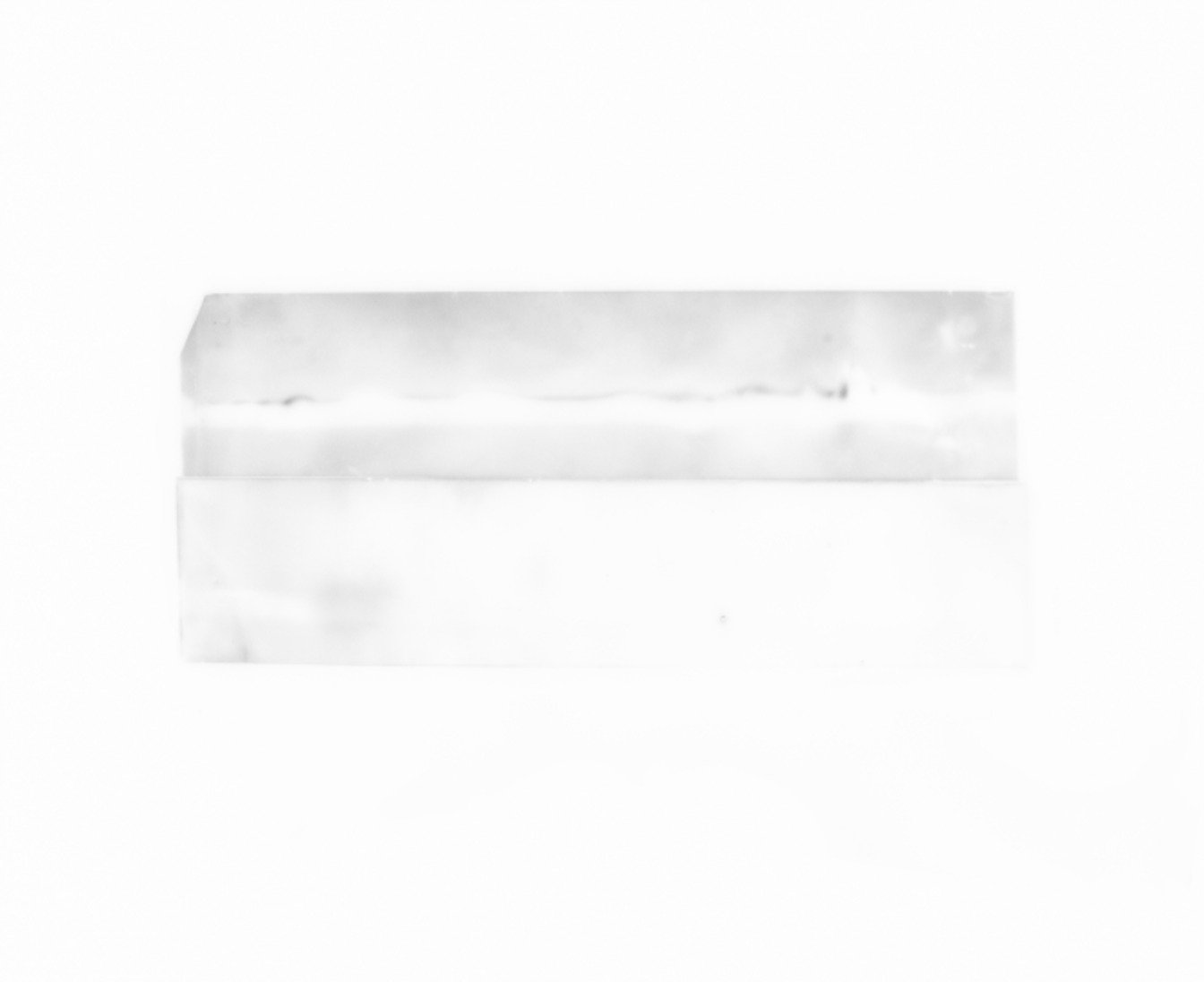

Supplement: Supplementary file 11 — Source data Fig. 4 [file 44319_2026_783_MOESM11_ESM.zip › Figure4/4N/STREMI-WT-KO-low exposure.Tif]

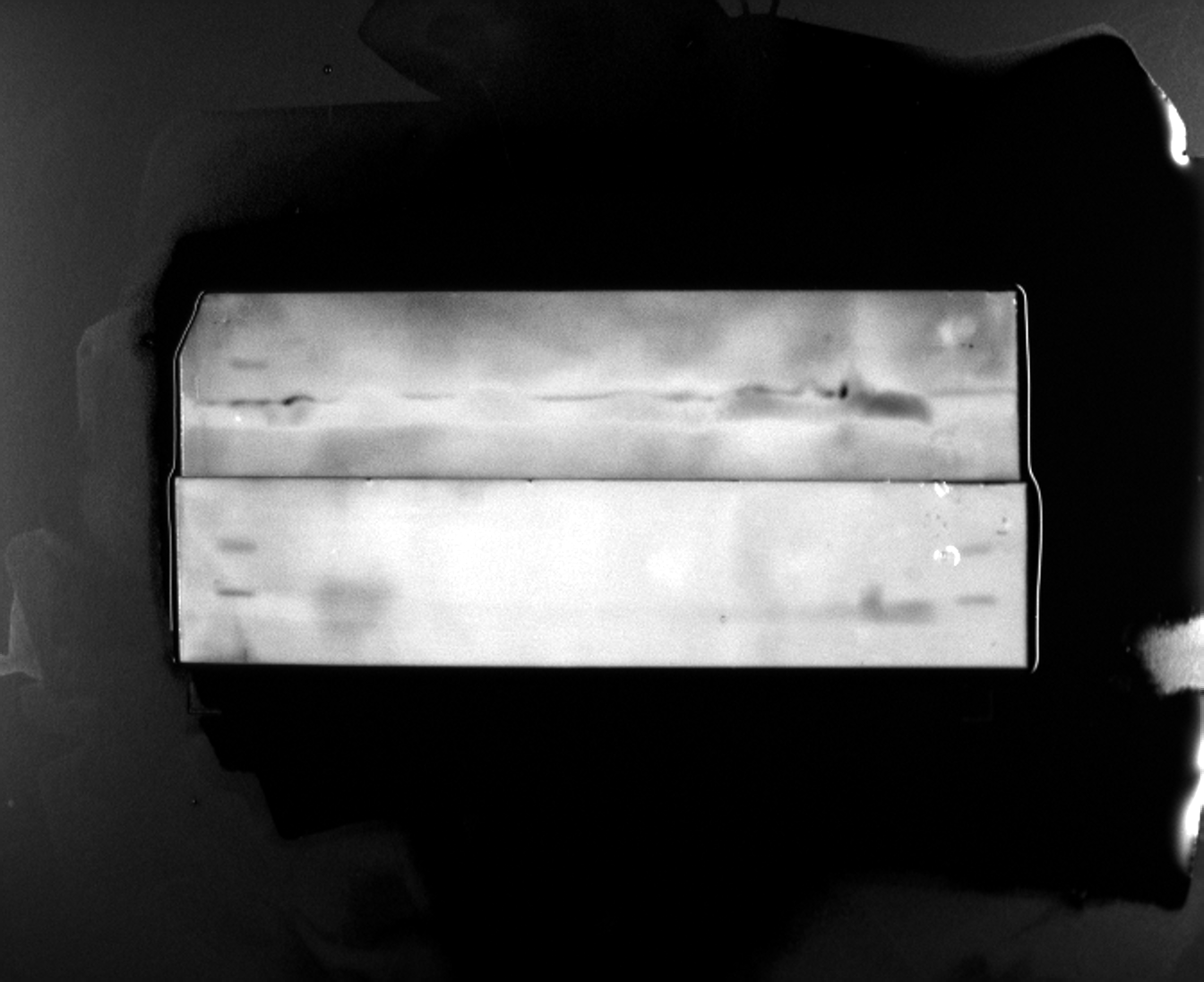

Supplement: Supplementary file 11 — Source data Fig. 4 [file 44319_2026_783_MOESM11_ESM.zip › Figure4/4N/STREMI-WT-KO-MERGE.Tif]

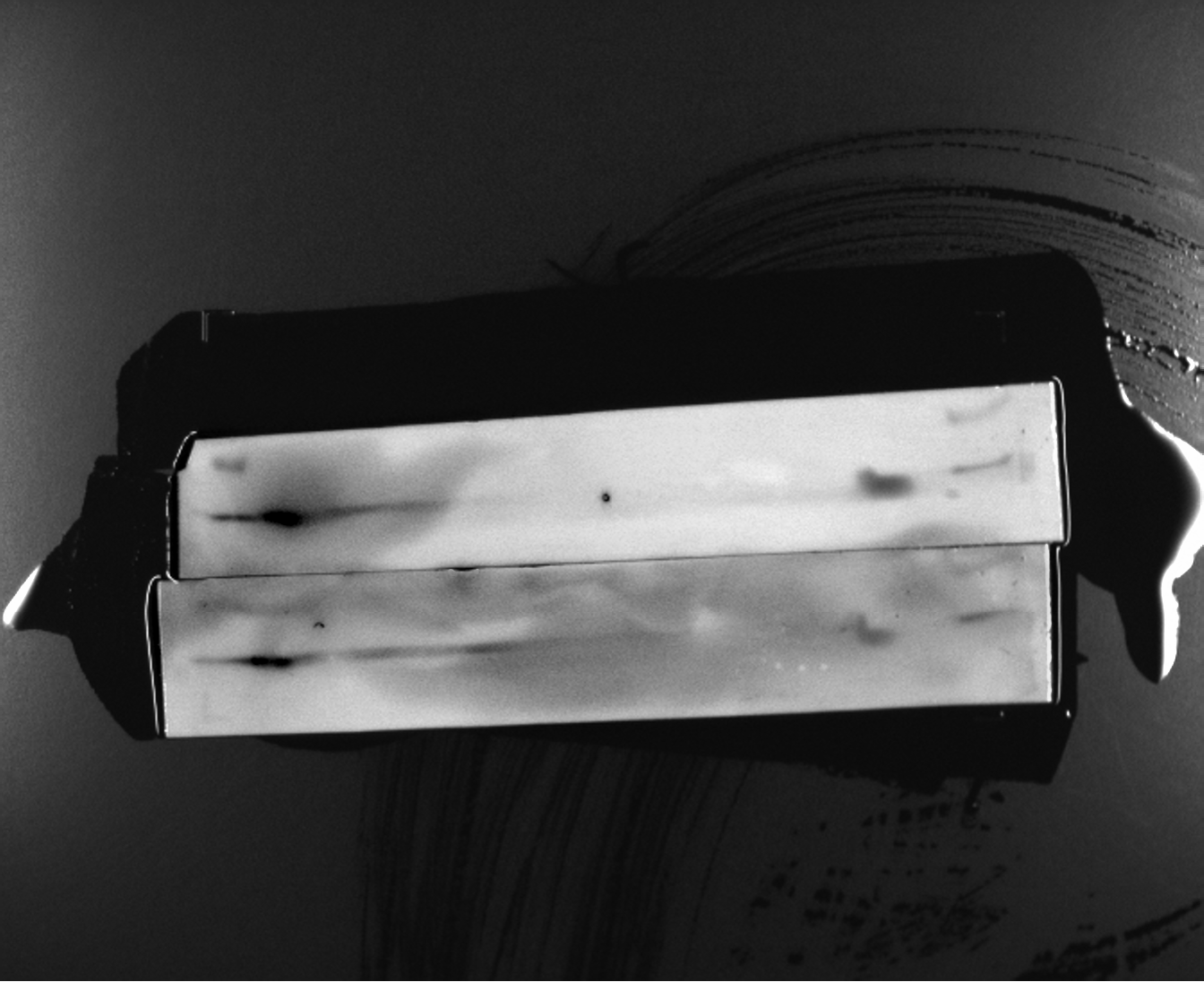

Supplement: Supplementary file 11 — Source data Fig. 4 [file 44319_2026_783_MOESM11_ESM.zip › Figure4/4O/MIC10-SCR-KO-Merge.Tif]

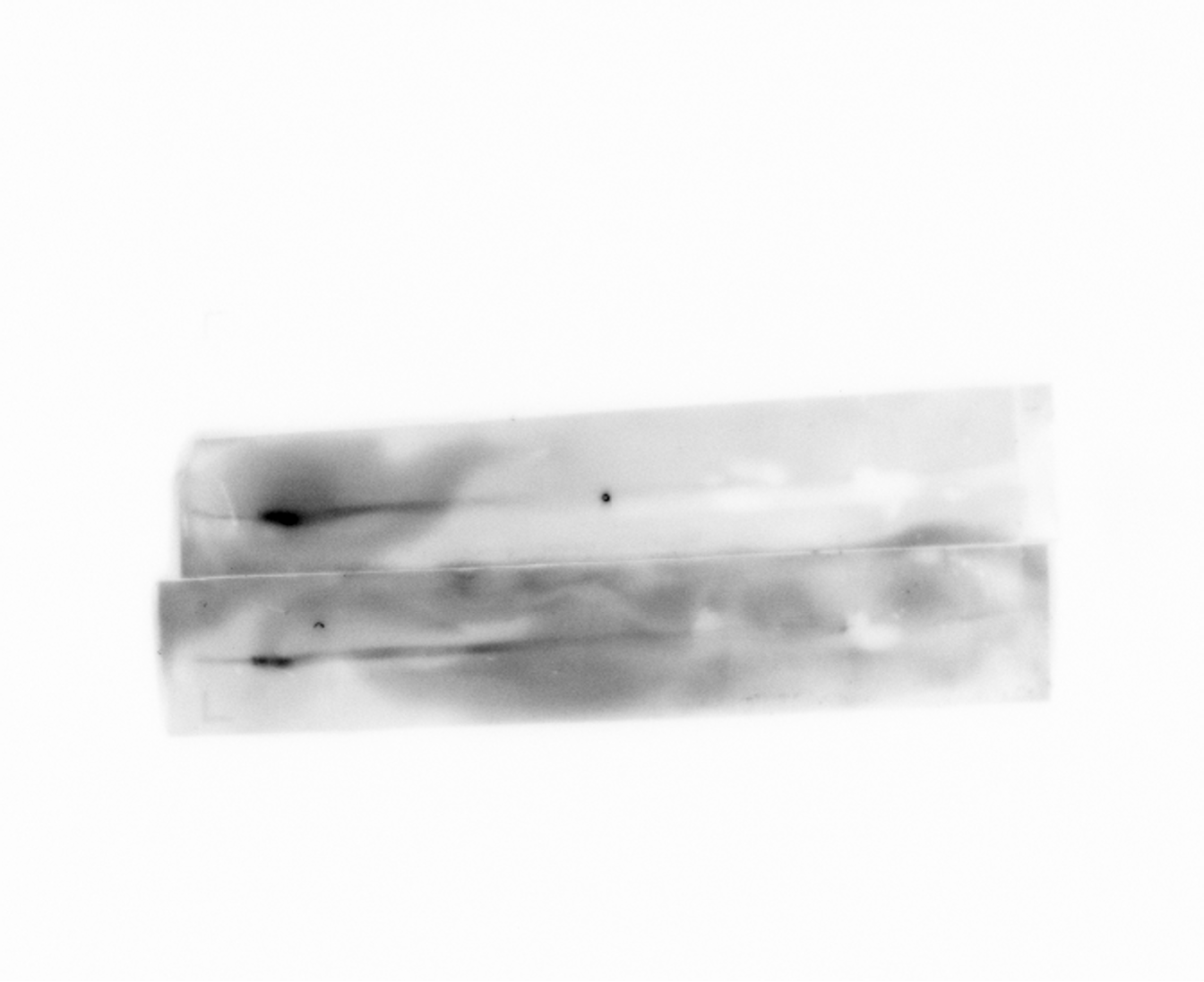

Supplement: Supplementary file 11 — Source data Fig. 4 [file 44319_2026_783_MOESM11_ESM.zip › Figure4/4O/MIC10-SCR-KO-Raw.Tif]

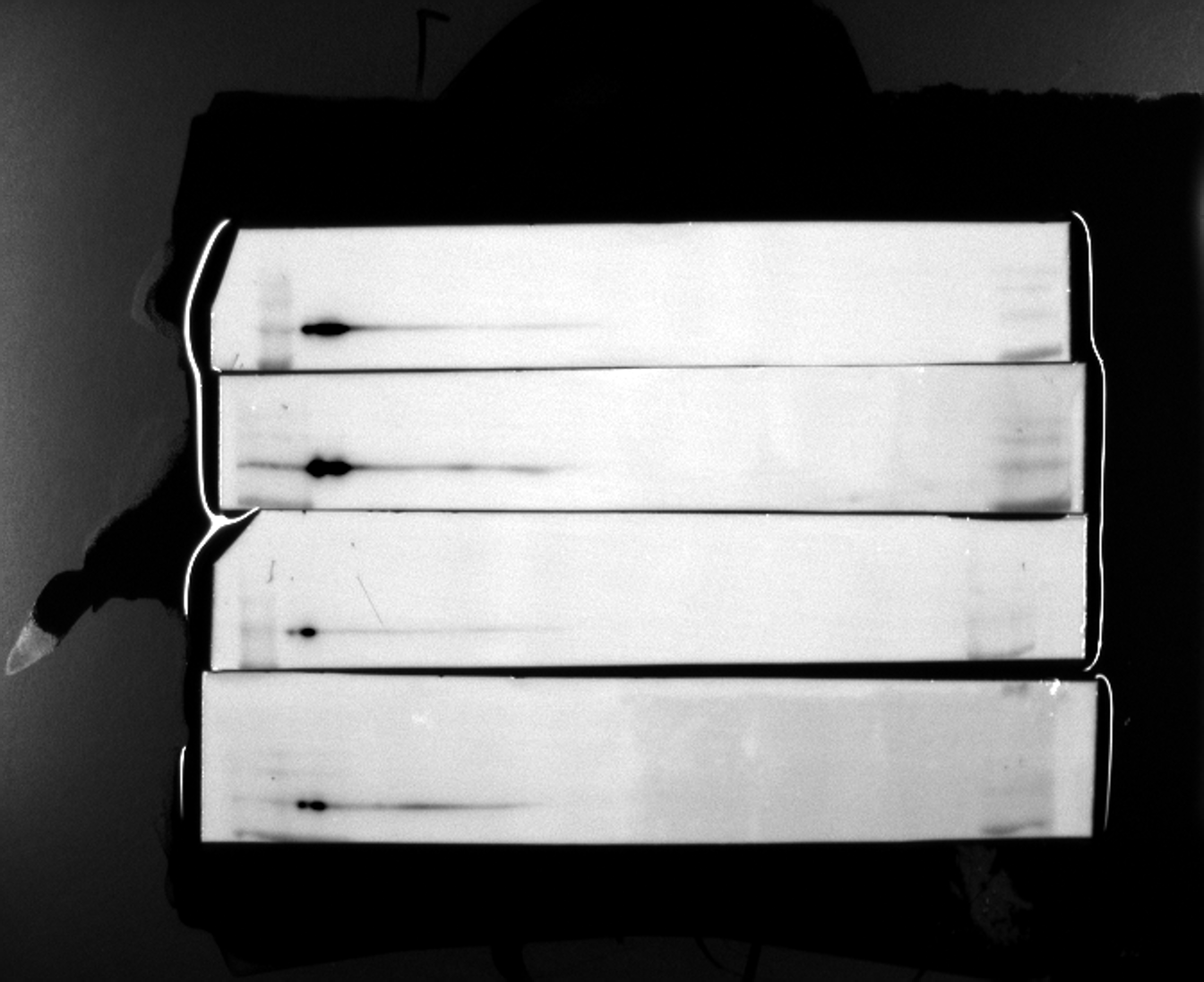

Supplement: Supplementary file 11 — Source data Fig. 4 [file 44319_2026_783_MOESM11_ESM.zip › Figure4/4O/MIC60-SCR-KO-MERGE.Tif]

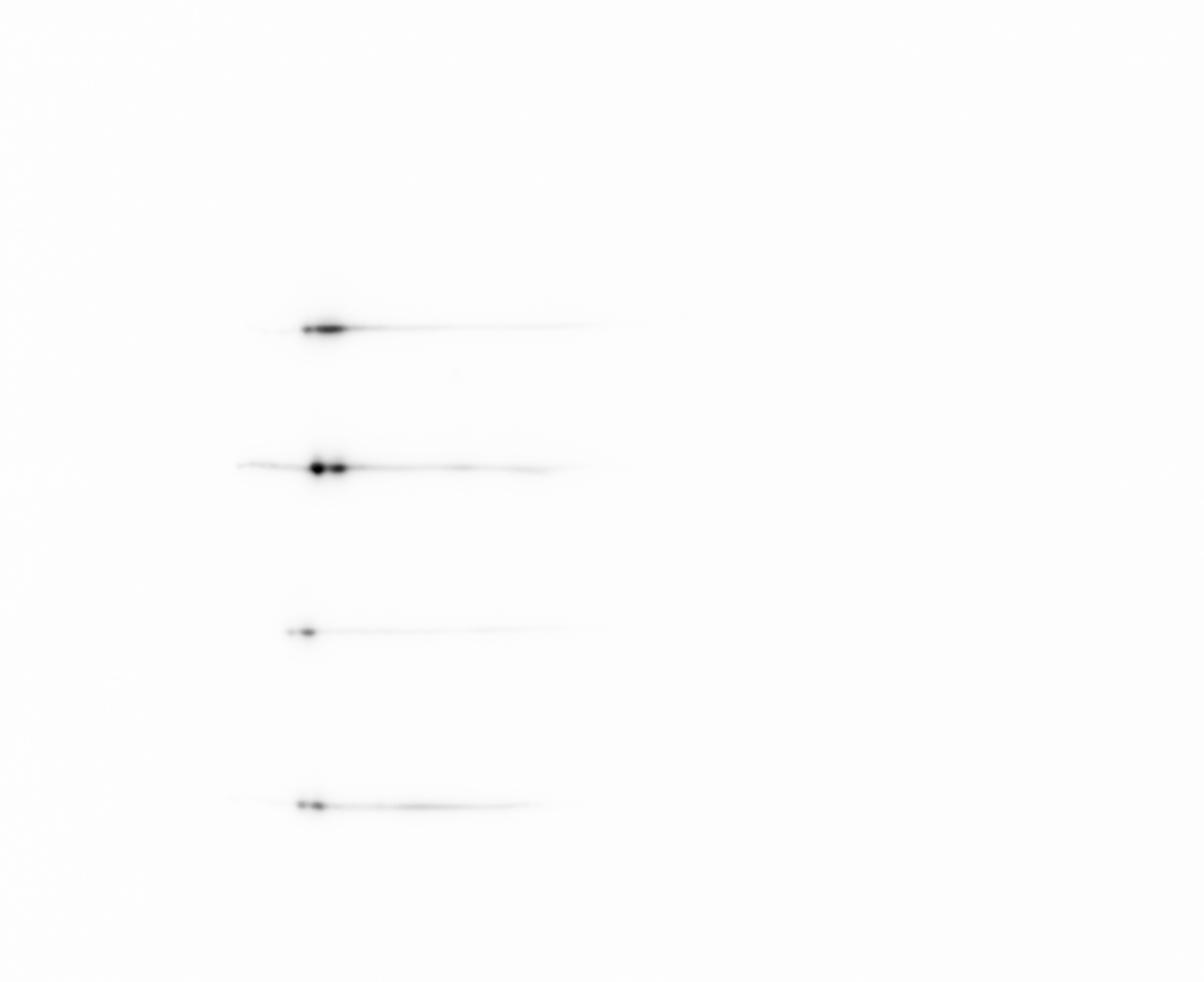

Supplement: Supplementary file 11 — Source data Fig. 4 [file 44319_2026_783_MOESM11_ESM.zip › Figure4/4O/MIC60-SCR-KO-Raw.Tif]

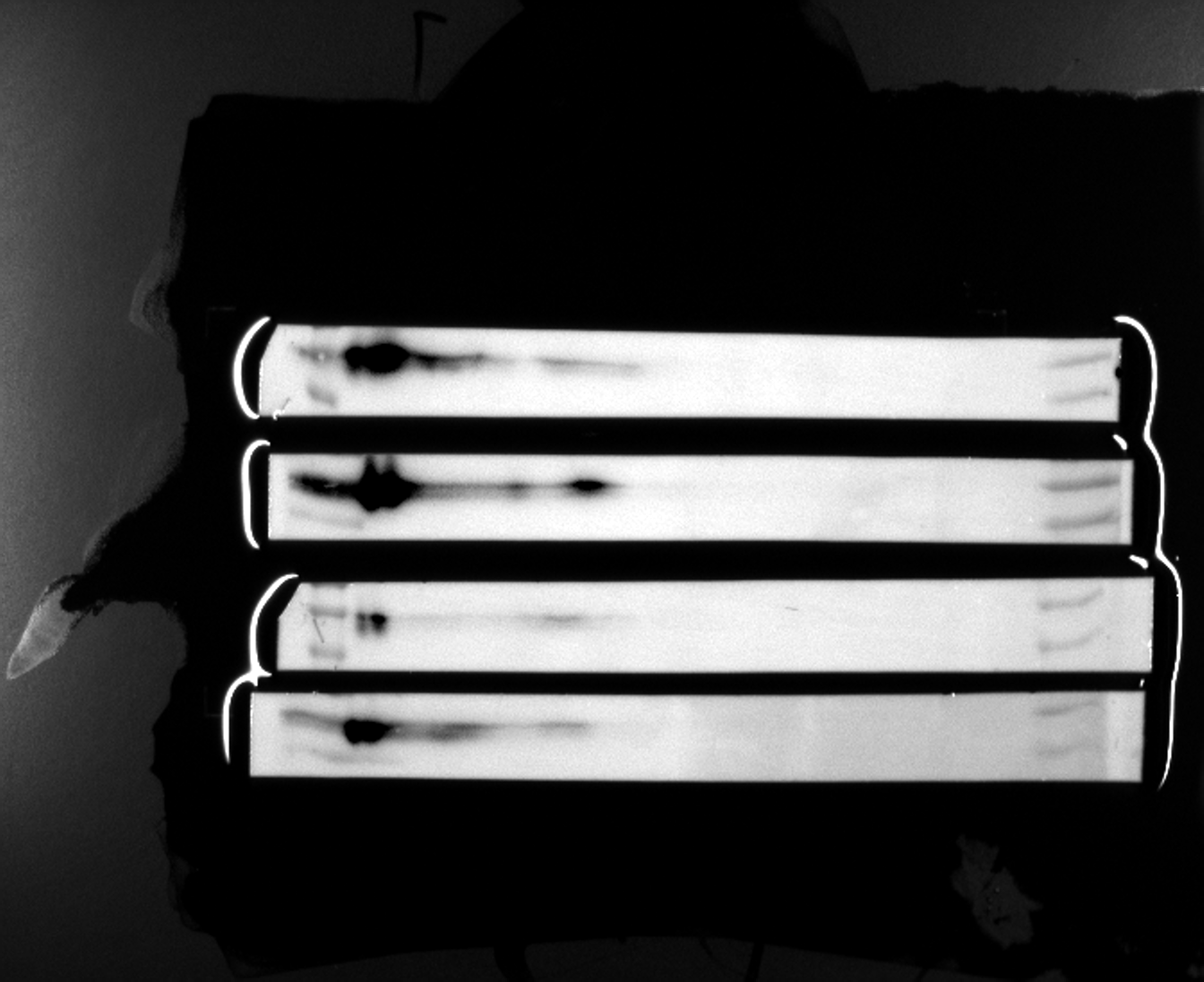

Supplement: Supplementary file 11 — Source data Fig. 4 [file 44319_2026_783_MOESM11_ESM.zip › Figure4/4O/SAMM50-SCR-KO-MERGE.Tif]

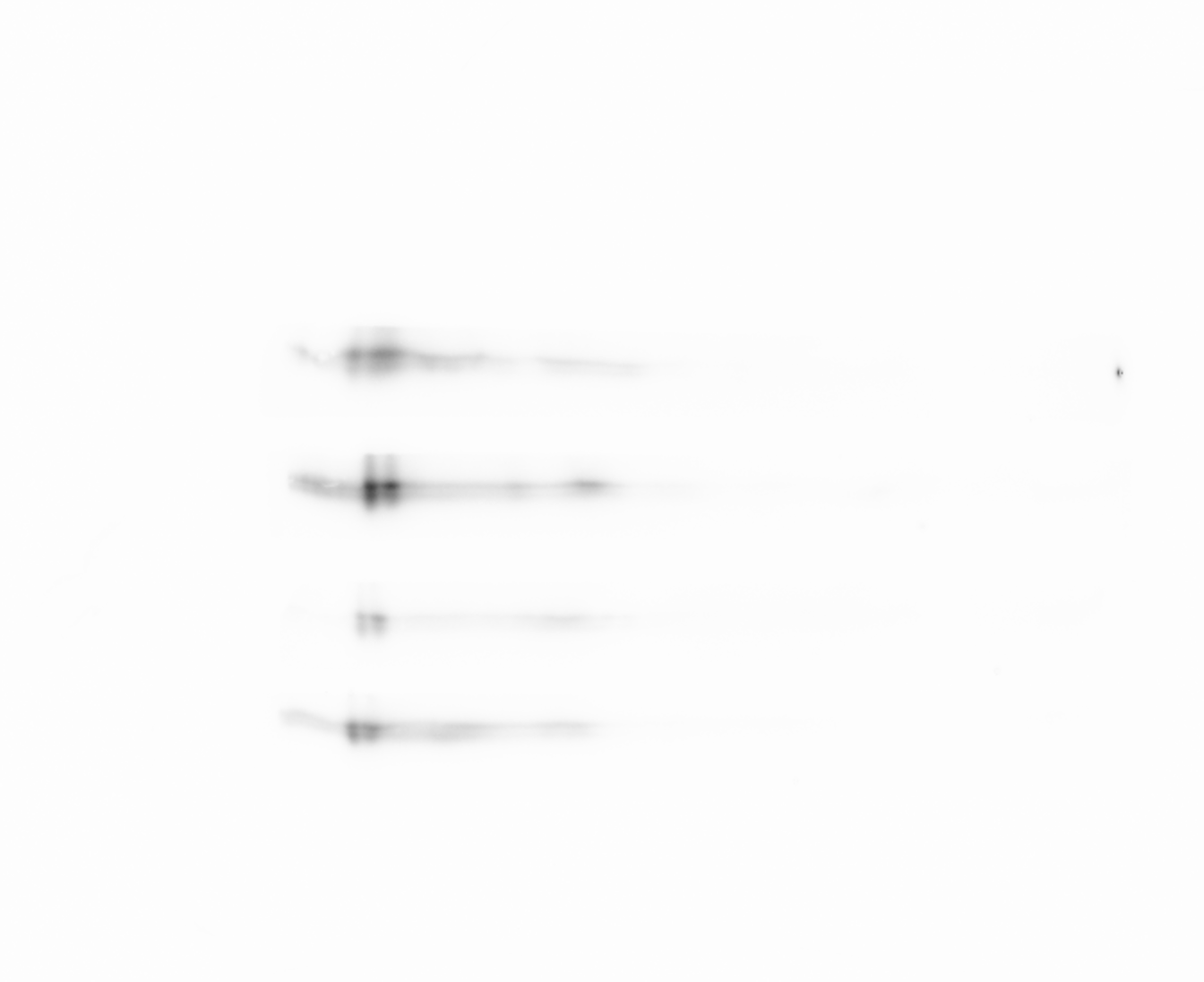

Supplement: Supplementary file 11 — Source data Fig. 4 [file 44319_2026_783_MOESM11_ESM.zip › Figure4/4O/SAMM50-SCR-KO-Raw.Tif]

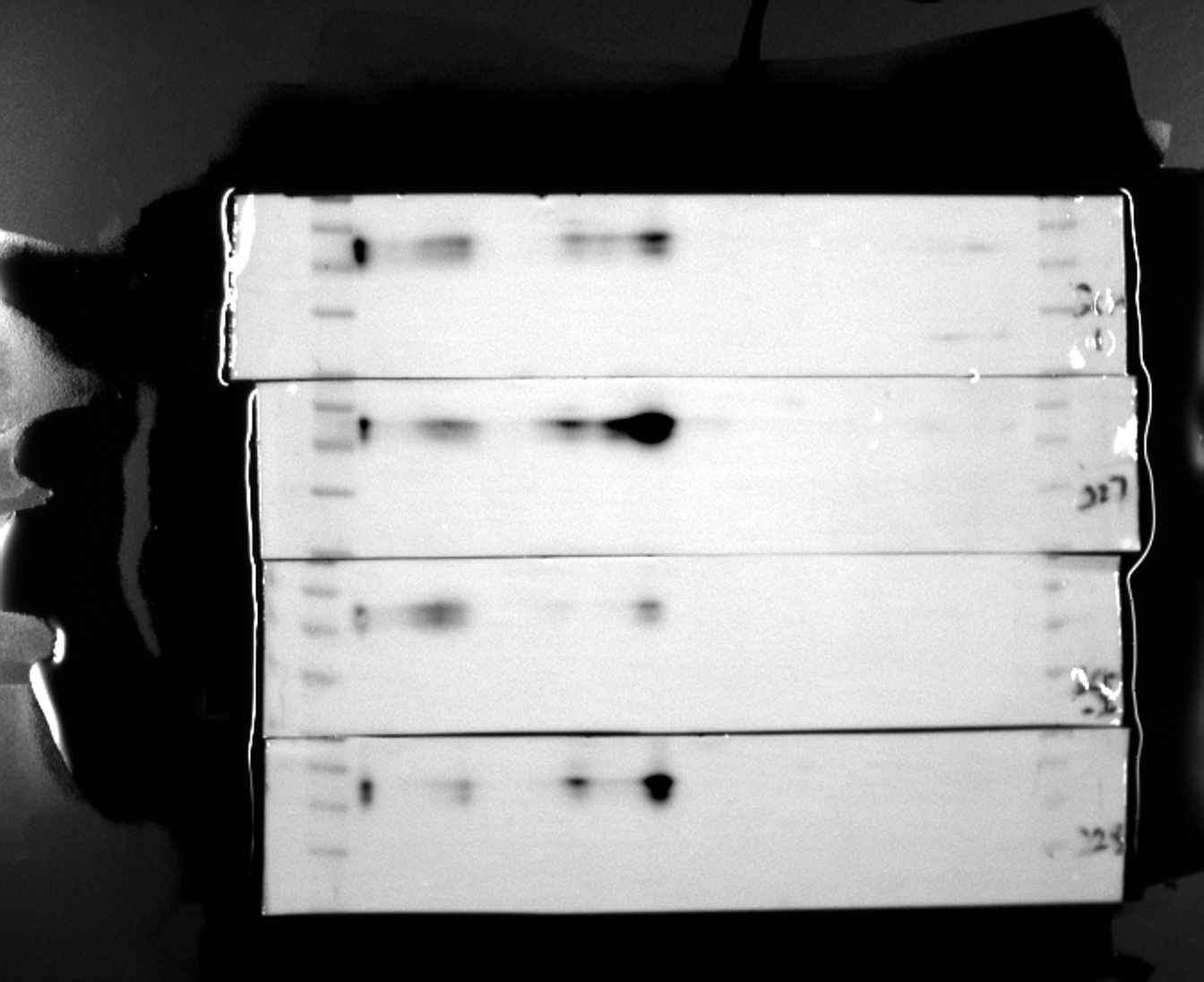

Supplement: Supplementary file 11 — Source data Fig. 4 [file 44319_2026_783_MOESM11_ESM.zip › Figure4/4O/UQCRC1-SCR-KO-Merge.Tif]

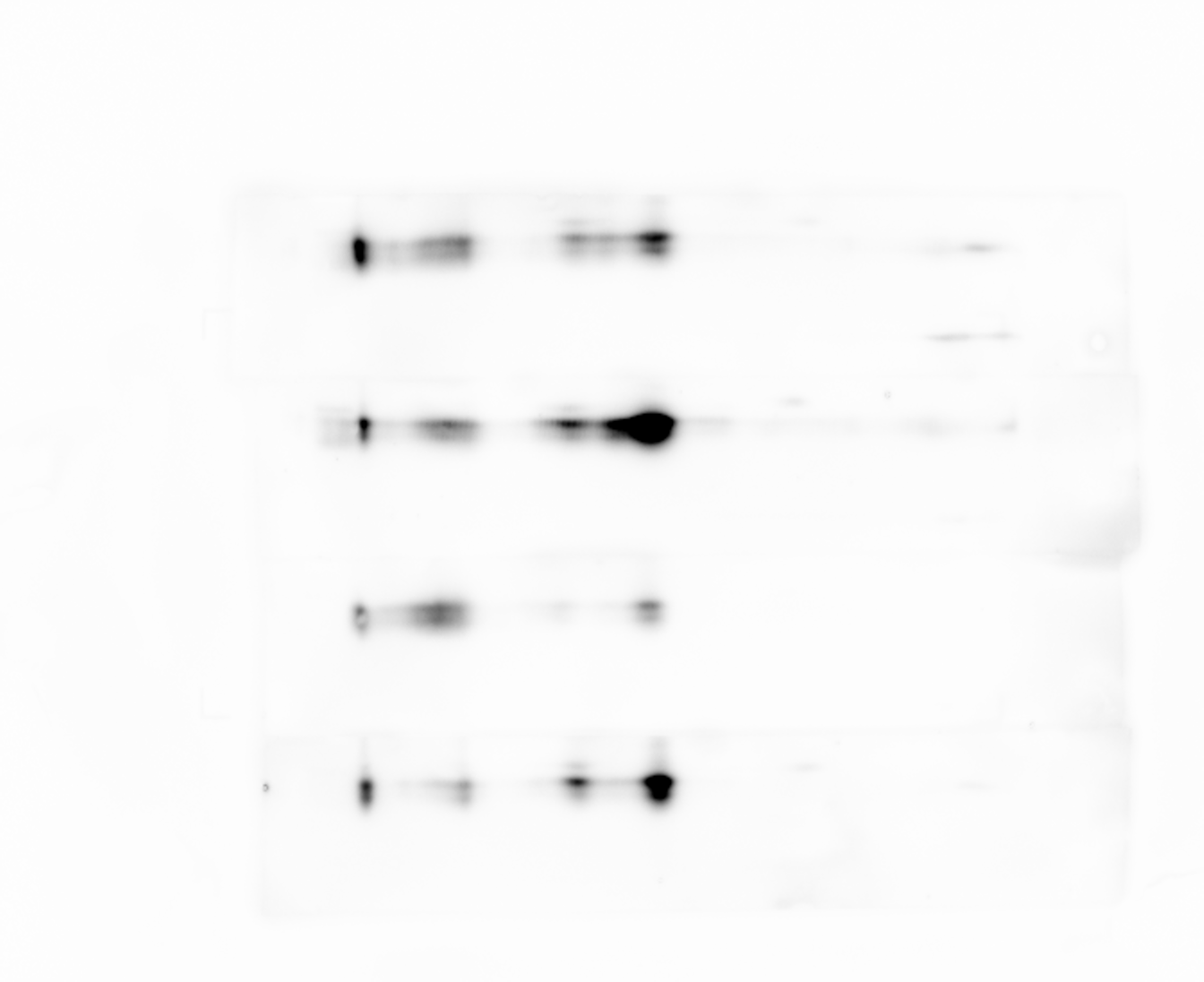

Supplement: Supplementary file 11 — Source data Fig. 4 [file 44319_2026_783_MOESM11_ESM.zip › Figure4/4O/UQCRC1-SCR-KO-Raw.Tif]

Source Data Fig. 5

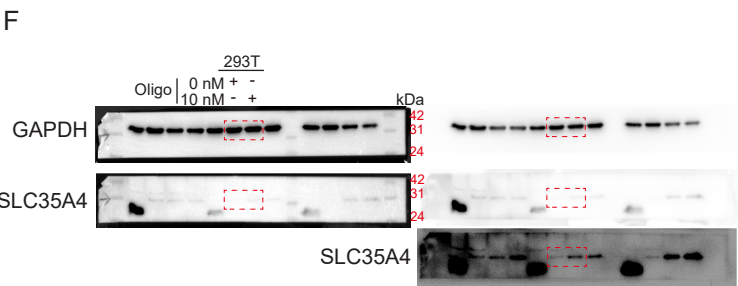

Supplement: Supplementary file 12 — Source data Fig. 5 [file 44319_2026_783_MOESM12_ESM.zip › Figure5/Figure_5_F_(Cropped_area_and marker).pdf]
