# Supplementary figures and images for: STREMI: a dual-function upstream ORF-encoded regulator of mitochondrial cristae architecture (part 2 of 2)
Source: EMBO Rep. 2026 May 2;27(12):3303–39. doi: 10.1038/s44319-026-00783-8 (PMC13303939; doi:10.1038/s44319-026-00783-8)

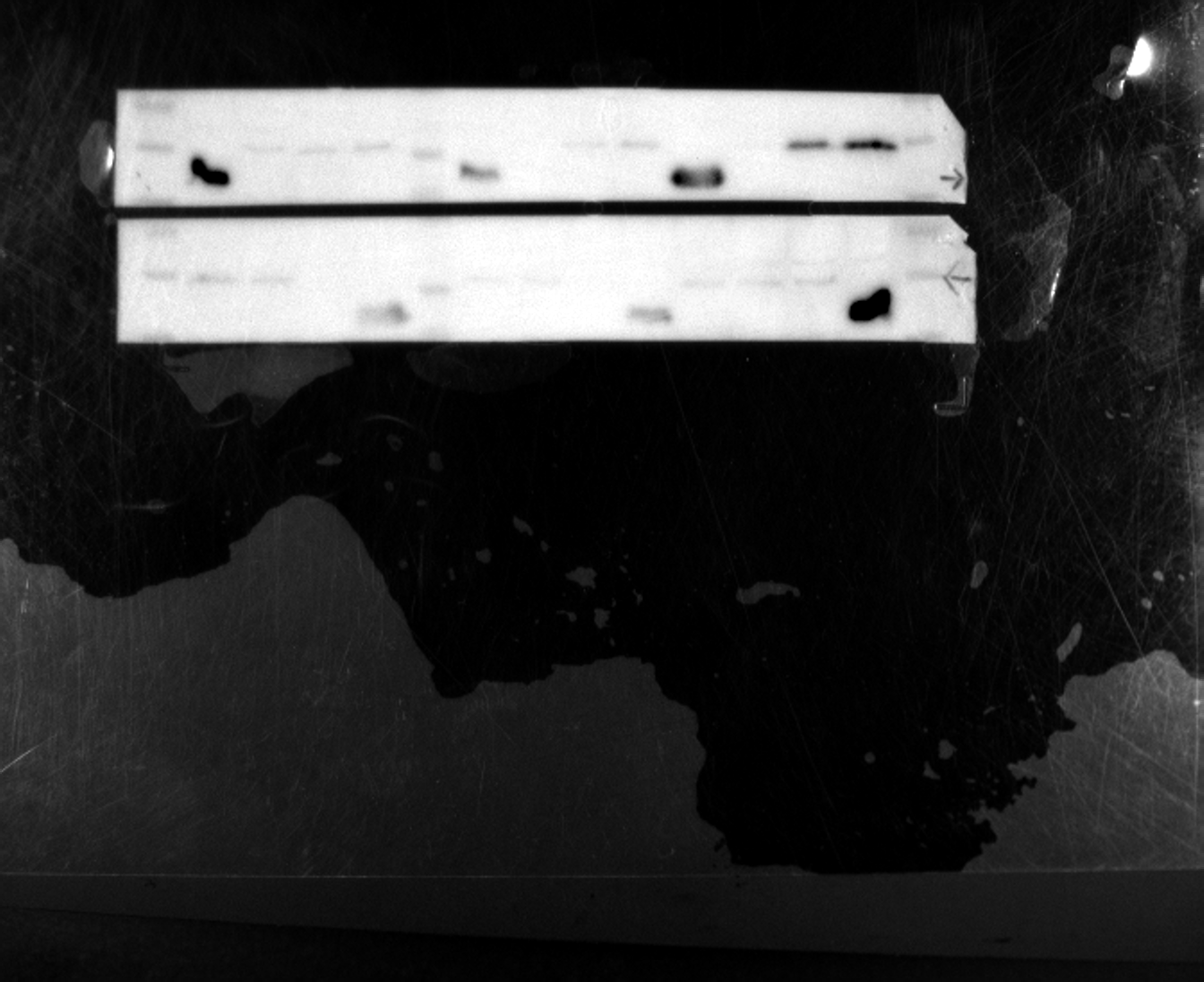

Supplement: Supplementary file 12 — Source data Fig. 5 [file 44319_2026_783_MOESM12_ESM.zip › Figure5/5F/Figure_5F_FLAG-Merge_data.Tif]

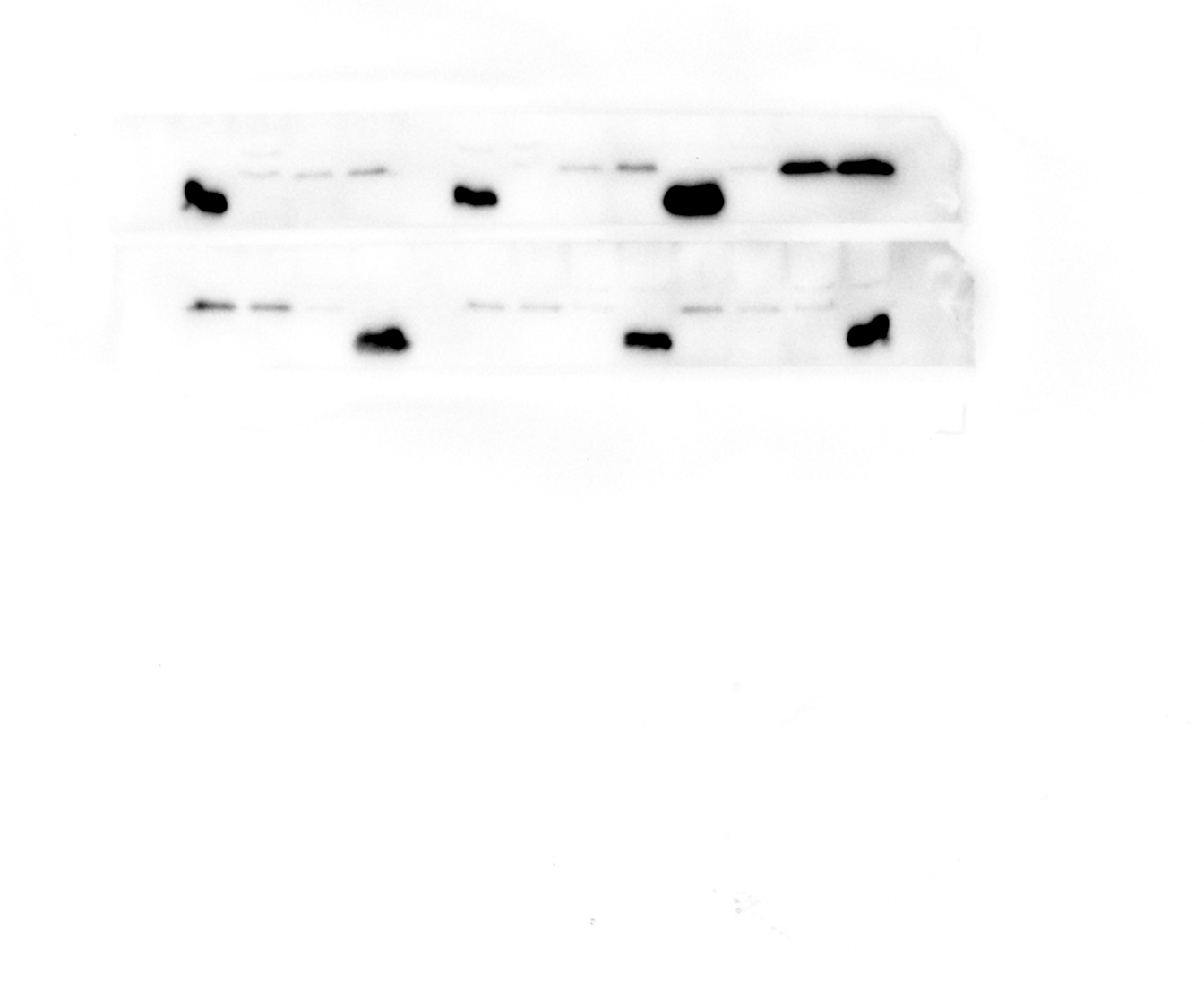

Supplement: Supplementary file 12 — Source data Fig. 5 [file 44319_2026_783_MOESM12_ESM.zip › Figure5/5F/Figure_5F_FLAG-Raw_data.Tif]

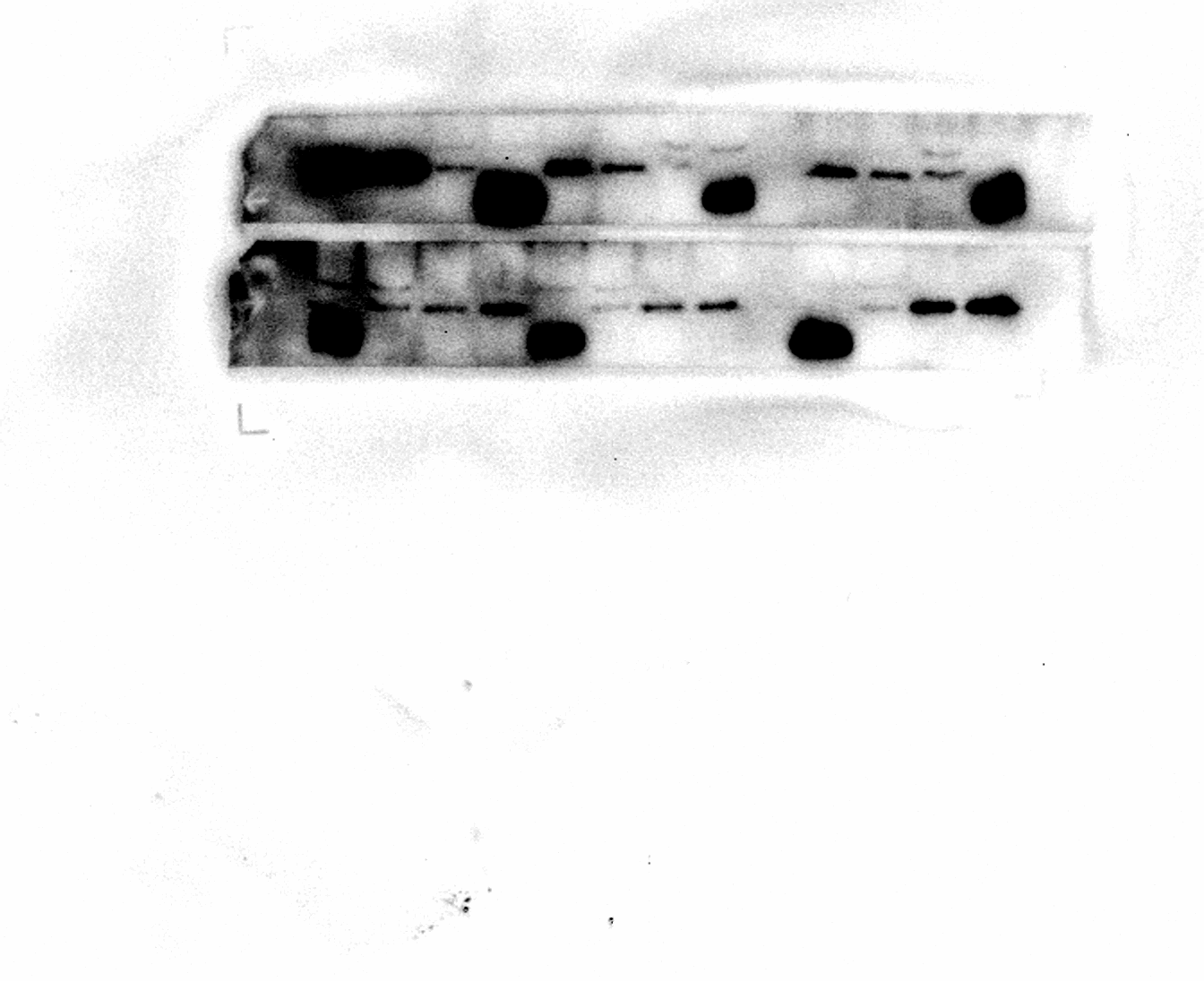

Supplement: Supplementary file 12 — Source data Fig. 5 [file 44319_2026_783_MOESM12_ESM.zip › Figure5/5F/Figure_5F_FLAG-Raw_data_long_exposure.Tif]

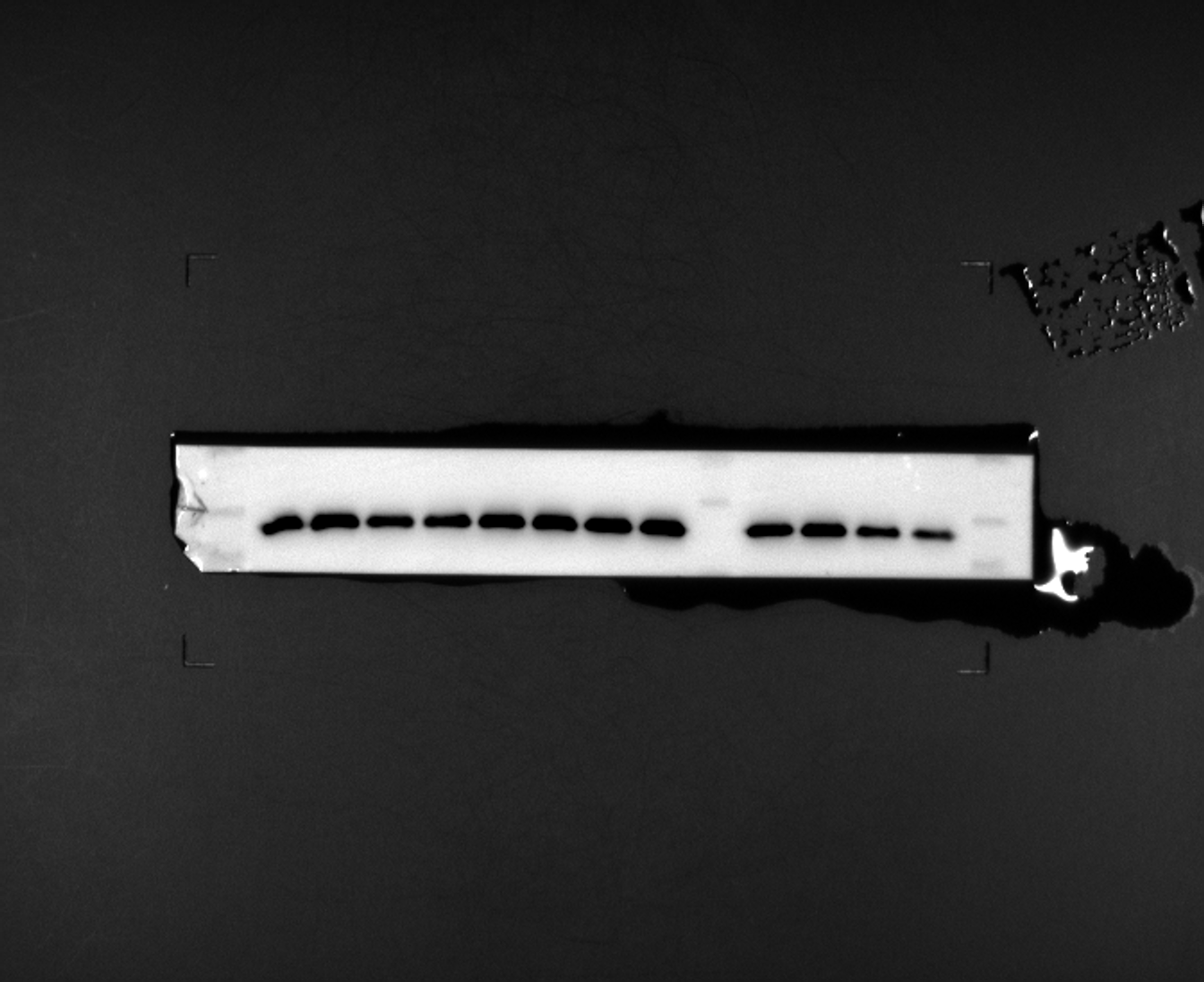

Supplement: Supplementary file 12 — Source data Fig. 5 [file 44319_2026_783_MOESM12_ESM.zip › Figure5/5F/Figure_5F_GAPDH-Merge_data.Tif]

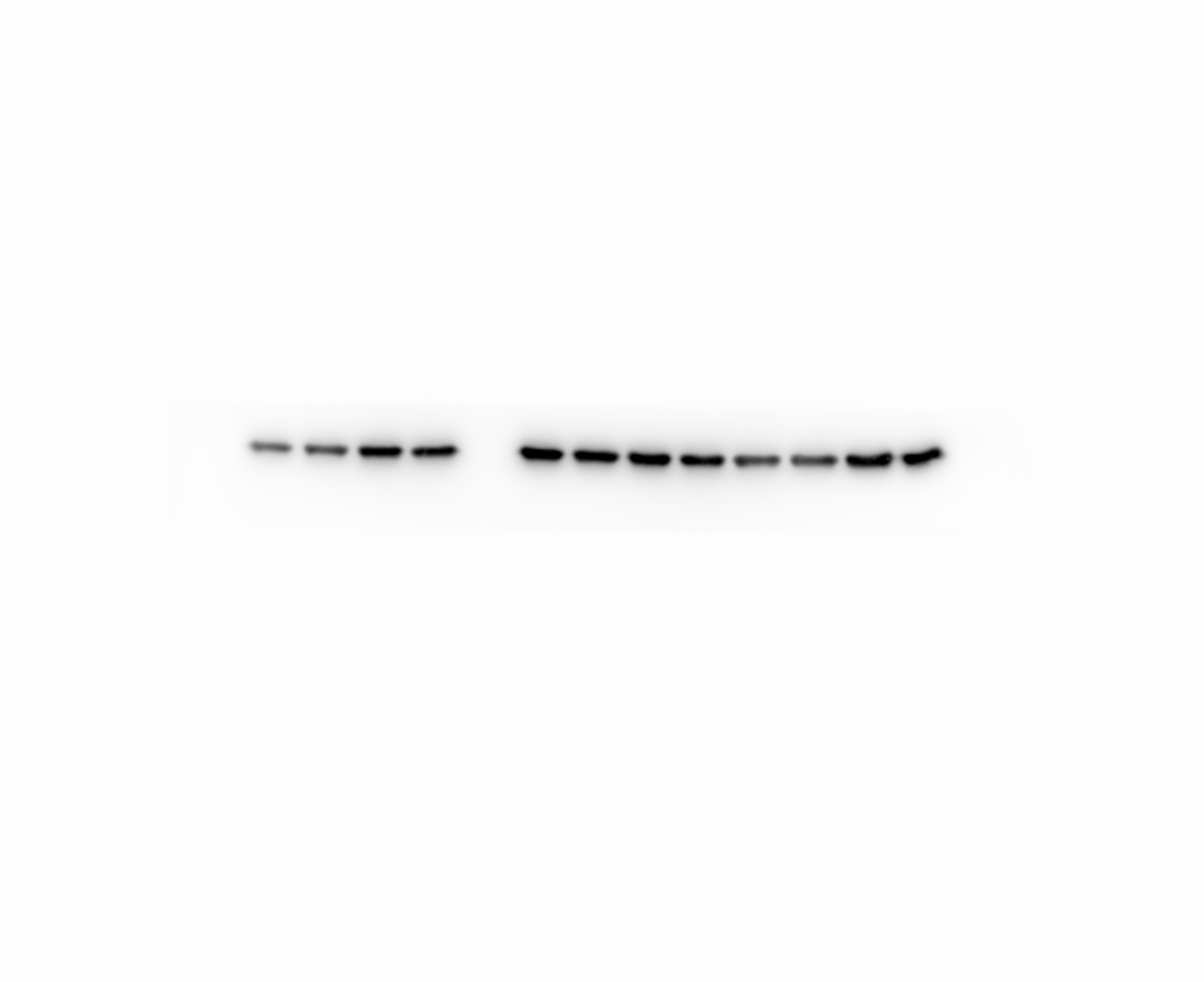

Supplement: Supplementary file 12 — Source data Fig. 5 [file 44319_2026_783_MOESM12_ESM.zip › Figure5/5F/Figure_5F_GAPDH-Raw_data.Tif.Tif]

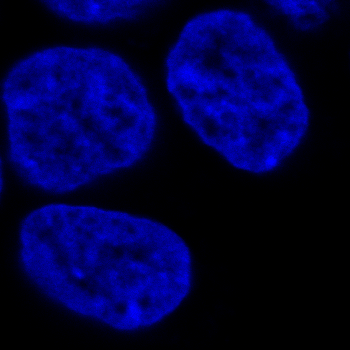

Supplement: Supplementary file 13 — Source data Fig. 6 [file 44319_2026_783_MOESM13_ESM.zip › Figure6/6A/DAPI.tif]

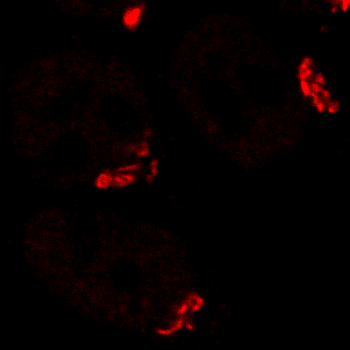

Supplement: Supplementary file 13 — Source data Fig. 6 [file 44319_2026_783_MOESM13_ESM.zip › Figure6/6A/GM130.tif]

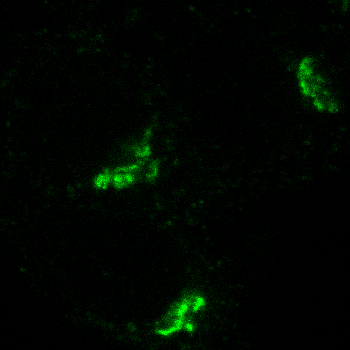

Supplement: Supplementary file 13 — Source data Fig. 6 [file 44319_2026_783_MOESM13_ESM.zip › Figure6/6A/HA.tif]

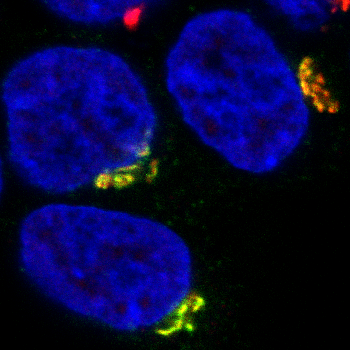

Supplement: Supplementary file 13 — Source data Fig. 6 [file 44319_2026_783_MOESM13_ESM.zip › Figure6/6A/Merge.tif]
