# Supplementary material for: STREMI: a dual-function upstream ORF-encoded regulator of mitochondrial cristae architecture
Source: EMBO Rep. 2026 May 2;27(12):3303–39. doi: 10.1038/s44319-026-00783-8 (PMC13303939; doi:10.1038/s44319-026-00783-8)
Supplement: Supplementary file 15 — Expanded View Figures [file 44319_2026_783_MOESM15_ESM.pdf]

Expanded View Figures

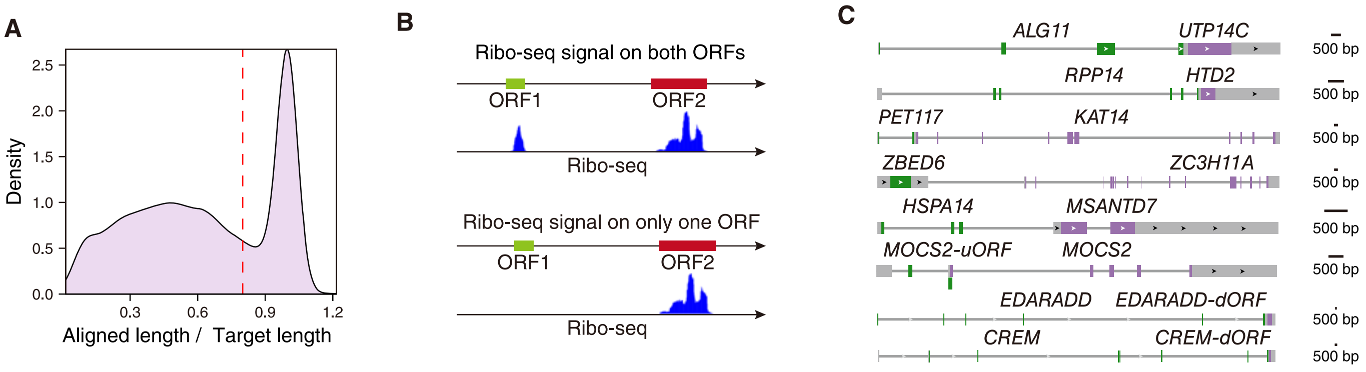

Figure EV1. Identification of human transcripts with multiple protein coding regions.

(**A**) Distribution of the ratio of aligned length to full length of the closest UniRef90 homolog among proteins with positive InterProScan annotations. (**B**) Schematic illustrating examples of candidate bicistronic transcripts that either pass (top) or fail (bottom) Ribo-seq signal screening. (**C**) Gene structures of remaining identified human bicistronic transcripts, corresponding to Fig. 1E.

 © The Author(s)

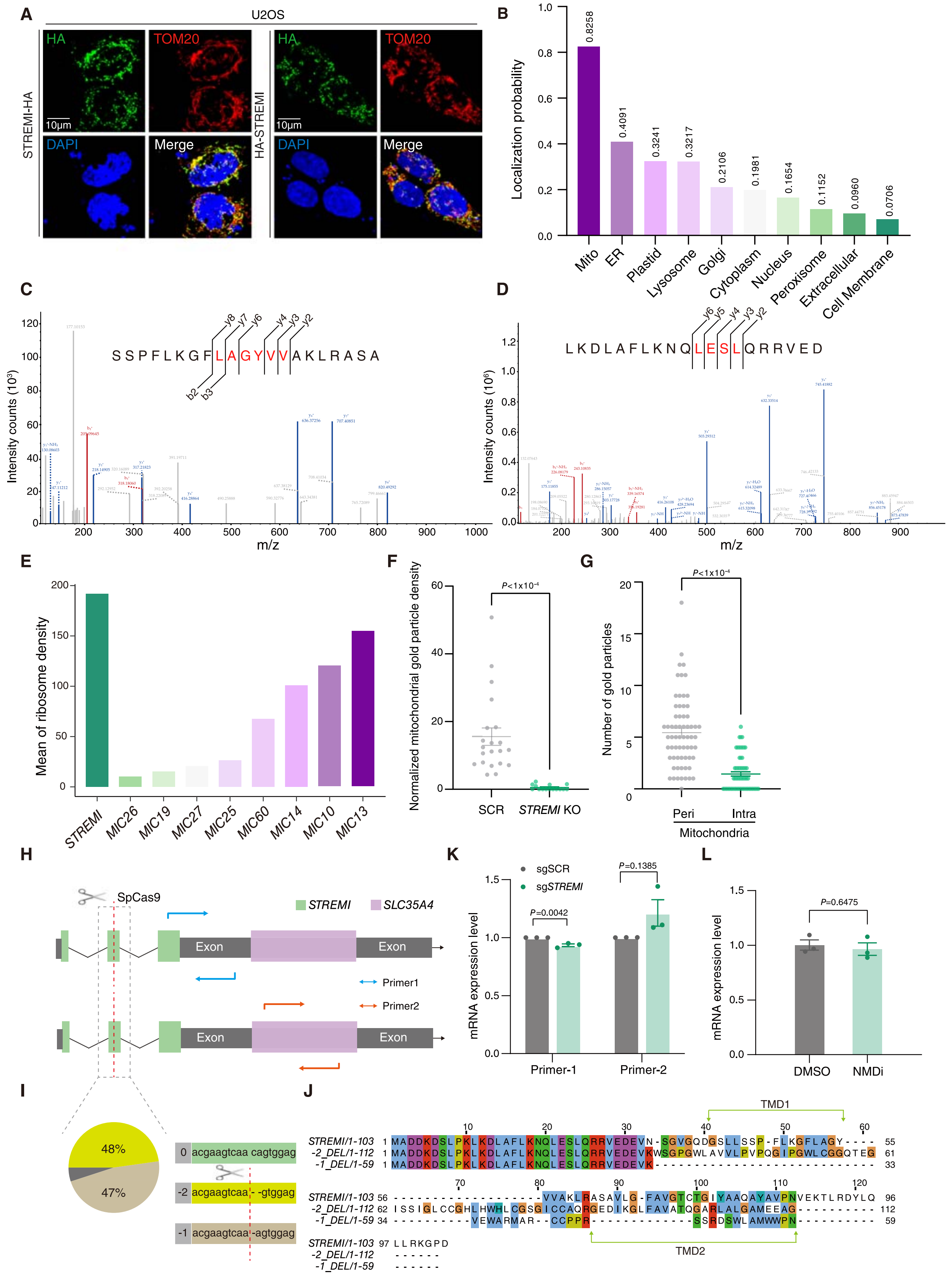
A
U2OS
STREMI-HA
HA-STREMI
HA
TOM20
DAPI
Merge
10μm
B
Localization probability
0.8258
0.4091
0.3241
0.3217
0.2106
0.1981
0.1654
0.1152
0.0960
0.0706
Mito
ER
Plastid
Lysosome
Golgi
Cytoplasm
Nucleus
Peroxisome
Extracellular
Cell Membrane
C
SSPFLKGFLAGYVVAKLRASA
Intensity counts (10³)
m/z
D
LKDLAFLKNQLESLQRRVED
Intensity counts (10⁶)
m/z
E
Mean of ribosome density
STREMI
MIC26
MIC19
MIC27
MIC25
MIC60
MIC14
MIC10
MIC13
F
Normalized mitochondrial gold particle density
P<1x10⁻⁴
SCR
STREMI KO
G
Number of gold particles
P<1x10⁻⁴
Peri
Intra
Mitochondria
H
SpCas9
STREMI
SLC35A4
Exon
Primer1
Primer2
K
sgSCR
sgSTREMI
P=0.0042
P=0.1385
mRNA expression level
Primer-1
Primer-2
L
P=0.6475
mRNA expression level
DMSO
NMDi
I
48%
47%
0 acgaagtcaa cagtggag
-2 acgaagtcaa - -gtggag
-1 acgaagtcaa -agtggag
J
TMD1
TMD2
STREMI/1-103
-2_DEL/1-112
-1_DEL/1-59

Figure EV2. The 5′ UTR of *SLC35A4* encodes a mitochondrial microprotein required for mitochondrial cristae morphogenesis.

(**A**) Immunofluorescence analysis of U2OS cells overexpressing N-terminally HA-tagged STREMI (HA-STREMI) or C-terminally HA-tagged STREMI (STREMI-HA), co-stained with mitochondrial marker TOM20. Scale bar, 10 µm. (**B**) Subcellular localization of STREMI predicted by DeepLoc 2.0. (**C**, **D**) MS/MS spectra of STREMI derived peptides identified in immunoprecipitation of Strep-tagged MIC10, a core MICOS subunit, in HEK293T cells via mass spectrometry analysis. (**E**) Ribosome occupancy across coding sequences (CDS) of *STREMI* and MICOS subunits. Mean ribosome density is shown for *STREMI* and subunits of MICOS. Ribosome footprints were extracted from the GWIPS-viz track of UCSC genome browser. (**F**) Mitochondrial gold particle density normalized to non-mitochondrial regions in SCR and *STREMI* KO HeLa cells. Data are presented as mean ± SEM ($n = 21$ mitochondria for SCR; $n = 15$ mitochondria for *STREMI* KO). *P* values are from unpaired two-tailed t tests ($P < 1 \times 10^{-4}$). (**G**) Quantification of gold particles in intra-mitochondrial and peri-mitochondrial regions. The peri-mitochondrial region was defined as the 50-nm zone surrounding the outer mitochondrial membrane. Data are presented as mean ± SEM ($n = 65$ mitochondria). *P* values are from paired two-tailed *t* tests ($P < 1 \times 10^{-4}$). (**H**) Schematic diagram showing the locations of qPCR primer pairs used to assess transcript abundance. (**I**) Distribution of genome-editing outcomes following SpCas9 targeting of the *STREMI* coding sequence. The frequencies of the two predominant frameshift alleles (−2 and −1) are indicated. (**J**) Predicted translation products from WT and the two predominant frameshift alleles in the *STREMI* KO line, shown as a sequence alignment. (**K**) qPCR analysis of transcript levels in SCR and *STREMI* KO cells. Data are presented as mean ± SEM ($n = 3$). *P* values are from two-tailed unpaired *t* tests: Primer 1 ($P = 0.0042$), Primer 2 ($P = 0.1385$). (**L**) qPCR analysis of transcript levels in *STREMI* KO cells following NMD inhibitor (NMDI14) treatment for 12 h. Data are presented as mean ± SEM ($n = 3$). *P* values are from two-tailed unpaired *t* tests ($P = 0.6475$).

 © The Author(s)

A

	Genotype				
Group	WT	HET	STREMI KO	X²(2)	*P* value
Male	14	32	11	1.1754	0.5556
Female	9	27	8	2.3182	0.3138
Total	23	59	19	3.1782	0.2041

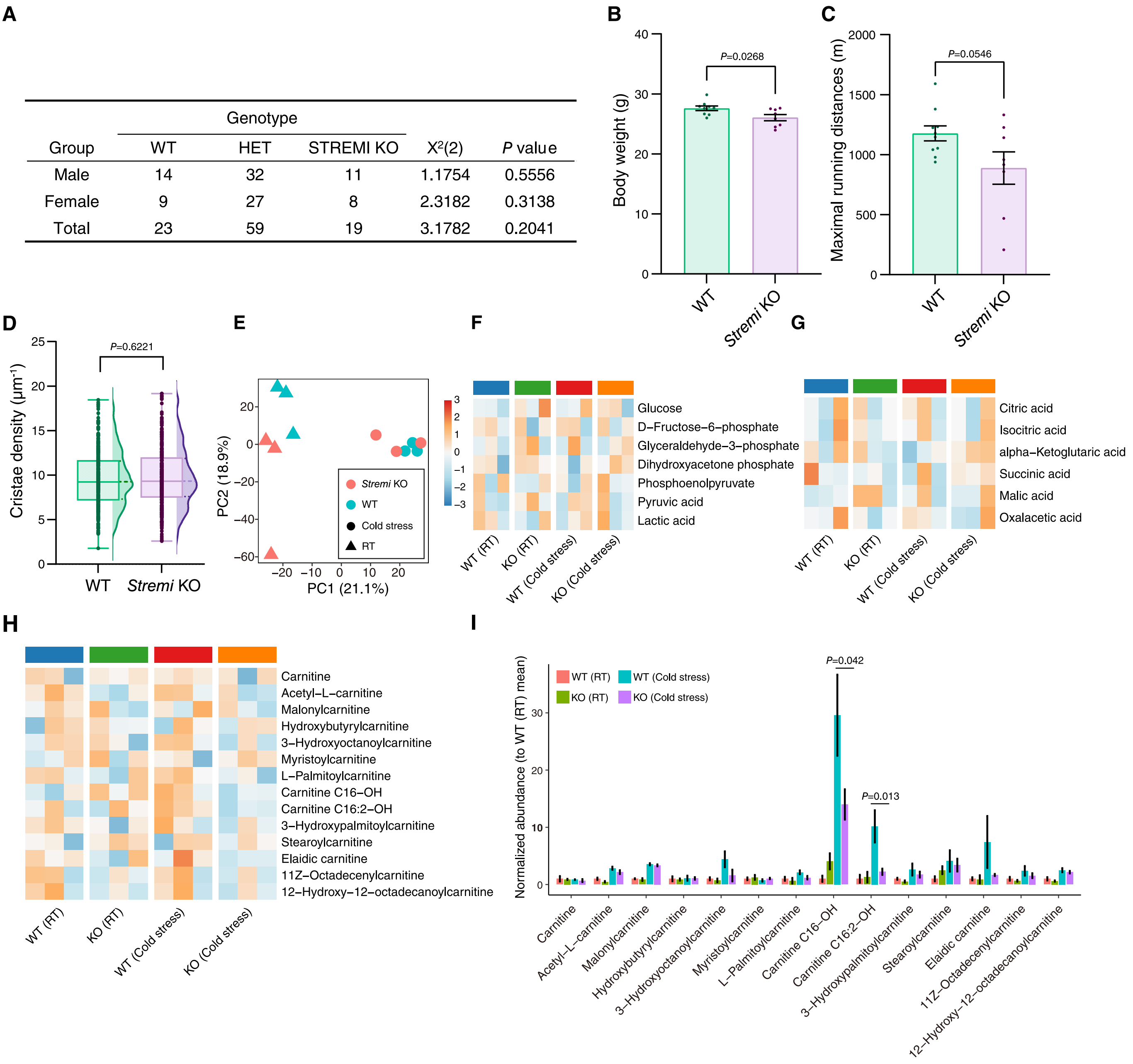

Figure EV3. *Stremi* deletion results in mild physiological phenotypes in mice.

(**A**) Genotype distribution compared with the expected Mendelian 1:2:1 ratio. Observed and expected numbers are indicated. Statistical significance was assessed by χ^2 goodness-of-fit test (df = 2). (**B**) Body weight of WT and *Stremi* KO mice. Data are presented as mean ± SEM (*n* = 9 mice for WT; *n* = 8 mice for *Stremi* KO). *P* values are from two-tailed unpaired *t* tests (*P* = 0.0268). (**C**) Maximum running distance of WT and *Stremi* KO mice during treadmill testing. Data are presented as mean ± SEM (*n* = 10 mice for WT; *n* = 8 mice for *Stremi* KO). *P* values are from two-tailed unpaired *t* tests (*P* = 0.0546). (**D**) Quantification of cristae density in heart mitochondria from WT and *Stremi* KO mice (*n* = 321 mitochondria for WT; *n* = 330 mitochondria for *Stremi* KO, 4 biological replicates). Box-and-whisker plots show the median (central line), interquartile range (box, 25th to 75th percentile), and minimum to maximum values (whiskers), with all individual data points displayed. *P* values are from two-tailed unpaired *t* tests (*P* = 0.6221). (**E**) Principal component analysis (PCA) of untargeted metabolomic profiles from brown adipose tissue (BAT) of wild-type (WT) and *Stremi*-knockout (KO) mice housed at room temperature (RT) or subjected to cold stress (4 °C for 5 days). (**F–H**) Heatmaps showing the relative abundance of metabolites from glycolysis (**F**), the tricarboxylic acid (TCA) cycle (**G**), and acylcarnitines (**H**). Values were log2-transformed and then z-scored across metabolites within each condition (RT and cold stress processed separately). (**I**) Acylcarnitine abundances in BAT normalized to WT at RT (compound-wise mean). Bars indicate mean normalized abundance and error bars represent SEM. *P* values were calculated using two-way ANOVA followed by Sidak's multiple-comparisons correction for within-compound comparisons (*n* = 3).

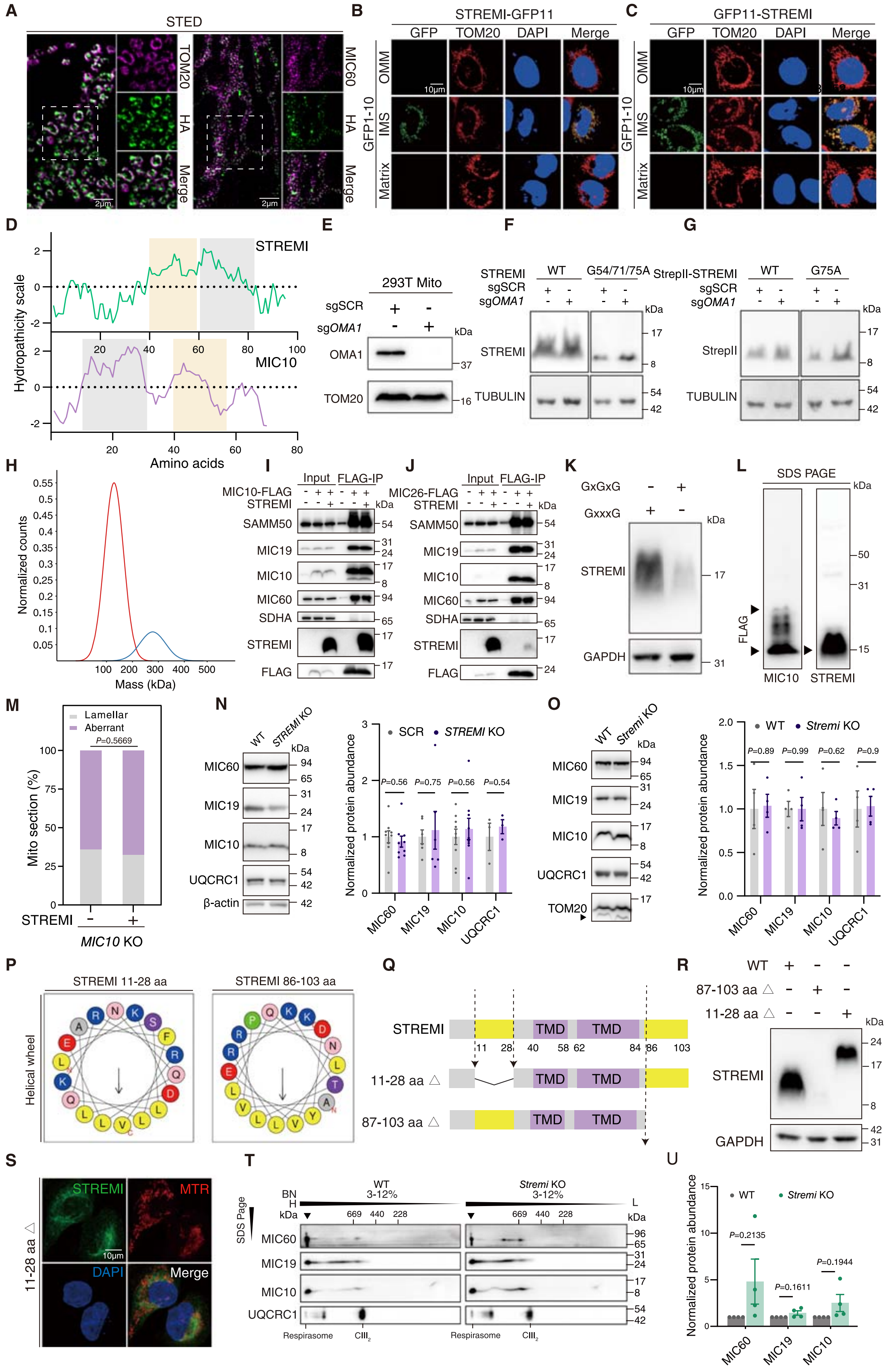

Figure EV4. STREMI oligomerizes with MIC10 and is required for optimal MICOS assembly.

(**A**) STED imaging of HA-STREMI co-stained with TOM20 (outer mitochondrial membrane marker) and MIC60 (inner mitochondrial membrane marker). Scale bars, 2 µm. (**B**, **C**) Representative confocal images of HeLa cells co-transfected with STREMI-GFP11 (**B**) or GFP11-STREMI (**C**) along with GFP1-10 targeted to the outer mitochondrial membrane (OMM), intermembrane space (IMS), or matrix. GFP fluorescence (green) and TOM20 staining (red) were used to assess the sub-mitochondrial localization of STREMI. Scale bars, 10 µm. (**D**) Hydropathy profiles of STREMI (green) and MIC10 (purple), generated using the Kyte-Doolittle scale, with hydrophobic regions highlighted. (**E**) Immunoblot analysis of OMA1 in mitochondrial lysates from SCR and *OMA1* KO 293T cells. (**F**) Immunoblotting of STREMI(G54/71/75 A) in control and *OMA1* KO HEK 293 T cells. (**G**) Immunoblotting of StrepII-STREMI(G75A) in control and *OMA1* KO HEK 293 T cells. (**H**) Mass photometry analysis of STREMI oligomerization using proteins expressed and purified from *E. coli*. (**I**, **J**) Validation of STREMI-MIC10 (**I**) and STREMI-MIC26 (**J**) associations under identical IP conditions. (**K**) Immunoblotting of STREMI mutant (L52/A69/C73G), in which the GxxxG-motif was replaced by GxGxG. (**L**) Immunoblotting of purified MIC10 and STREMI in SDS–PAGE. MIC10, but not STREMI, forms SDS-resistant oligomers (arrowed). (**M**) Percentage of mitochondria with aberrant morphology in *MIC10* KO with or without STREMI expression in HeLa cells ($n = 88$ mitochondria for MIC10 KO; $n = 144$ mitochondria for MIC10 KO with STREMI expression). P values were calculated using Fisher's exact test based on raw counts ($P = 0.5669$). (**N**) SDS–PAGE of whole cell lysate (WCL) from control and *STREMI* KO HEK293T cells. Protein abundance was normalized to the level of β-actin. Data are presented as mean ± SEM ($n = 9$, except for MIC19 where $n = 6$ and UQCRC1 where $n = 3$). P values are from two-tailed unpaired t tests: MIC60 ($P = 0.56$), MIC19 ($P = 0.75$), MIC10 ($P = 0.56$), UQCRC1 ($P = 0.54$). (**O**) SDS–PAGE of purified WT and *Stremi* KO BAT mitochondria followed by immunoblotting for the core subunits of MICOS. Protein abundance was normalized to the level of TOM20. Data are presented as mean ± SEM ($n = 4$). P values are from two-tailed unpaired t tests: MIC60 ($P = 0.89$), MIC19 ($P = 0.99$), MIC10 ($P = 0.62$), UQCRC1 ($P = 0.9$). (**P**) Helical wheel projections of STREMI N-terminal (11–28 aa) and C-terminal (87–103 aa) segments. Hydrophobic residues are shown in yellow, negatively charged in red, positively charged in blue, and hydrophilic in purple. (**Q**) STREMI domain architecture: transmembrane domain (TMD) flanked by predicted amphipathic helices (yellow). Truncations ΔN (11–28) and ΔC (87–103) target these helices. (**R**) Immunoblotting of STREMI truncation mutants ΔN (11–28) and ΔC (87–103). (**S**) Confocal images of STREMI truncation mutant ΔN (11–28), co-stained with MitoTracker (red) and DAPI (blue). The STREMI mutant exhibits mislocalization outside mitochondria. Scale bar, 10 µm. (**T**) Two-dimensional blue native electrophoresis of BAT mitochondria from WT and *Stremi* KO mice. The position of the largest MICOS assembly is marked by a triangle. (**U**) Normalized relative abundance of smaller MICOS assemblies compared with the largest MICOS assembly, quantified from BAT mitochondria of WT and *Stremi* KO mice. The WT control ratio was set to 1. Data are presented as mean ± SEM ($n = 4$). P values are from two-tailed paired t tests: MIC60 ($P = 0.2135$), MIC19 ($P = 0.1611$), MIC10 ($P = 0.1944$).

© The Author(s)

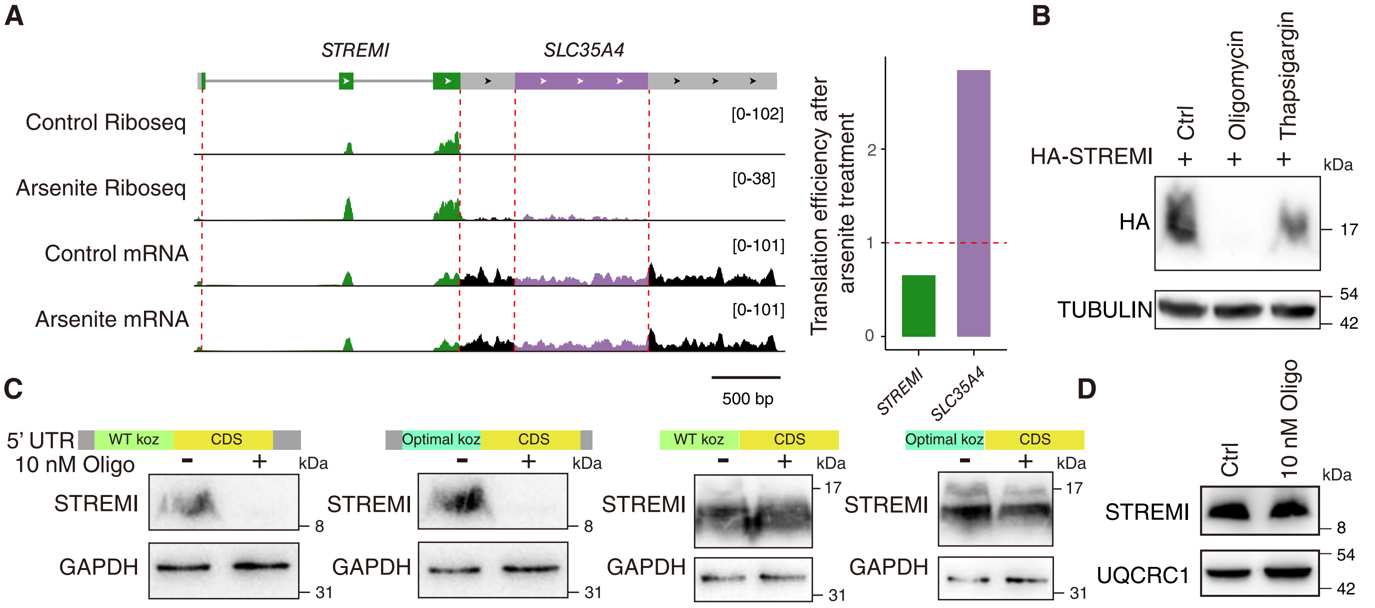

Figure EV5. The STREMI-encoding uORF mediates stress-responsive translation of *SLC35A4*.

(**A**) Ribosome footprint density on *SLC35A4* transcripts in control and arsenite-treated conditions. Data from Andreev et al (Andreev et al, 2015). (**B**) Immunoblotting of transiently expressed STREMI (within its native 5′UTR) in HEK293T cells treated with oligomycin and thapsigargin. (**C**) Dependence of ISR-responsive translation repression on the Kozak sequence and 5′UTR context. (**D**) Immunoblotting of endogenous STREMI in HEK293T cells after a 24-h treatment with oligomycin.

 © The Author(s)

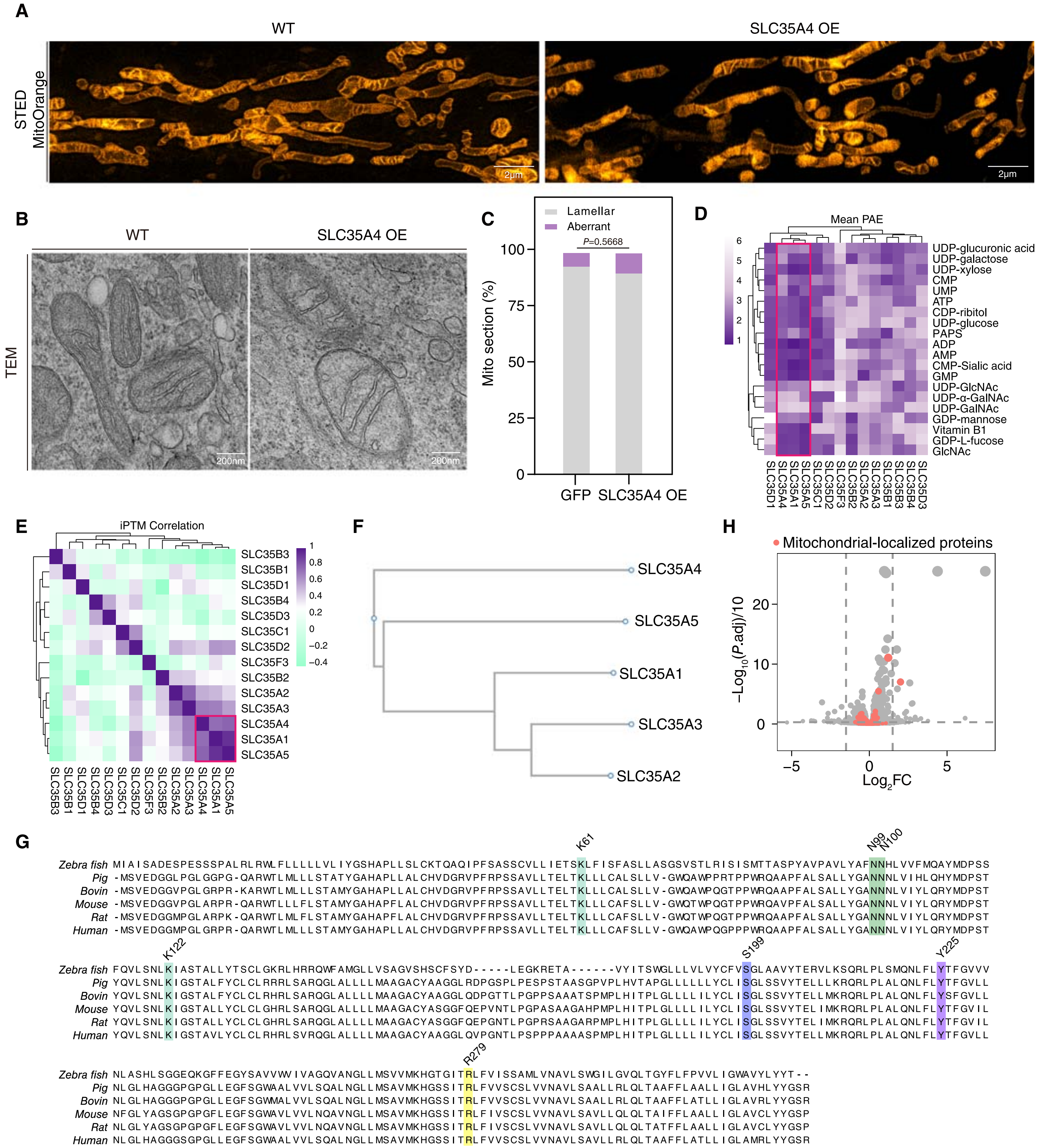
A
WT
SLC35A4 OE
STED MitoOrange
2μm
2μm
B
WT
SLC35A4 OE
TEM
200nm
200nm
C
Lamellar
Aberrant
P=0.5668
Mito section (%)
100
75
50
25
0
GFP
SLC35A4 OE
D
Mean PAE
6
5
4
3
2
1
UDP-glucuronic acid
UDP-galactose
UDP-xylose
CMP
UMP
ATP
CDP-ribitol
UDP-glucose
PAPS
ADP
AMP
CMP-Sialic acid
GMP
UDP-GlcNAc
UDP-α-GalNAc
UDP-GalNAc
GDP-mannose
Vitamin B1
GDP-L-fucose
GlcNAc
SLC35D1 SLC35A4 SLC35A1 SLC35A5 SLC35C1 SLC35D2 SLC35F3 SLC35A2 SLC35B2 SLC35A3 SLC35B1 SLC35B3 SLC35B4 SLC35D3
E
iPTM Correlation
1
0.8
0.6
0.4
0.2
0
−0.2
−0.4
SLC35B3
SLC35B1
SLC35D1
SLC35B4
SLC35D3
SLC35C1
SLC35D2
SLC35F3
SLC35B2
SLC35A2
SLC35A3
SLC35A4
SLC35A1
SLC35A5
SLC35B3 SLC35B1 SLC35D1 SLC35B4 SLC35D3 SLC35C1 SLC35D2 SLC35F3 SLC35B2 SLC35A2 SLC35A3 SLC35A4 SLC35A1 SLC35A5
F
SLC35A4
SLC35A5
SLC35A1
SLC35A3
SLC35A2
H
Mitochondrial-localized proteins
−Log10(P.adj)/10
20
10
0
−5
0
5
Log2FC
G
K61
N99
N100
Zebra fish MIAISADESPESSSPALRLRWLFLLLLLVLIYGSHAPLLSLCKTQAQIPFSASSCVLLIETSKLFISFASLLASGSVSTLRISISMTTASPYAVPAVLYAFNNHLVVFMQAYMDPSS
Pig -MSVEDGGLPGLGGPG-QARWTLMLLLSTATYGAHAPLLALCHVDGRVPFRPSSAVLLTELTKLLLCALSLLV-GWQAWPPRTPPWRQAAPFALSALLYGANNNLVIHLQHYMDPST
Bovin -MSVEDGGVPGLGRPR-KARWTLMLLLSTAMYGAHAPLLALCHVDGRVPFRPSSAVLLTELTKLLLCALSLLV-GWQAWPQGTPPWRQAAPFALSALLYGANNNLVIYLQRYMDPST
Mouse -MSVEDGGVPGLARPR-QARWTLLLFLSTAMYGAHAPFLALCHVDGRVPFRPSSAVLLTELTKLLLCAFSLLV-GWQTWPQGTPPWRQAVPFALSALLYGANNNLVIYLQRYMDPST
Rat -MSVEDGGMPGLARPK-QARWTLMLFLSTAMYGAHAPFLALCHVDGRVPFRPSSAVLLTELTKLLLCAFSLLV-GWQTWPQGTPPWRQAAPFALSALLYGANNNLVIYLQRYMDPST
Human -MSVEDGGMPGLGRPR-QARWTLMLLLSTAMYGAHAPLLALCHVDGRVPFRPSSAVLLTELTKLLLCAFSLLV-GWQAWPQGPPPWRQAAPFALSALLYGANNNLVIYLQRYMDPST
K122
S199
Y225
Zebra fish FQVLSNLKIASTALLYTSCLGKRLHRRQWFAMGLLVSAGVSHSCFSYD-----LEGKRETA------VYITSWGLLLVLVYCFVSGLAAVYTERVLKSQRLPLSMQNLFLYTFGVVV
Pig YQVLSNLKIGSTALFYCLCLRRRLSARQGLALLLLMAAGACYAAGGLRDPGSPLPESPSTAASGPVPLHVTAPGLLLLLLYCLISGLSSVYTELLLKRQRLPLALQNLFLYTFGVLL
Bovin YQVLSNLKIGSTALFYCLCLRHRLSARQGLALLLLMAAGACYAAGGLQDPGTTLPGPPSAAATSPMPLHITPLGLLLLILYCLISGLSSVYTELLMKRQRLPLALQNLFLYSFGVLL
Mouse YQVLSNLKIGSTALLYCLCLGHRLSARQGLALLLLMAAGACYASGGFQEPVNTLPGPASAAGAHPMPLHITPLGLLLLILYCLISGLSSVYTELIMKRQRLPLALQNLFLYTFGVIL
Rat YQVLSNLKIGSTALLYCLCLGHRLSARQGLALLLLMAAGACYASGGFQEPGNTLPGPRSAAGARPMPLHITPLGLLLLILYCLISGLSSVYTELIMKRQRLPLALQNLFLYTFGVIL
Human YQVLSNLKIGSTAVLYCLCLRHRLSVRQGLALLLLMAAGACYAAGGLQVPGNTLPSPPPAAAASPMPLHITPLGLLLLILYCLISGLSSVYTELLMKRQRLPLALQNLFLYTFGVLL
R279
Zebra fish NLASHLSGGEQKGFFEGYSAVVWIVAGQVANGLLMSVVMKHGTGITRLFVISSAMLVNAVLSWGILGVQLTGYFLFPVVLIGWAVYLYYT--
Pig NLGLHAGGGPGPGLLEGFSGWAALVVLSQALNGLLMSAVMKHGSSITRLFVVSCSLVVNAVLSAALLRLQLTAAFFLAALLIGLAVHLYYGSR
Bovin NLGLHAGGGPGPGLLEGFSGWMALVVLSQALNGLLMSAVMKHGSSITRLFVVSCSLVVNAVLSAALLRLQLTAAFFLATLLIGLAVRLYYGSR
Mouse NFGLYAGSGPGPGFLEGFSGWAVLVVLNQAVNGLLMSAVMKHGSSITRLFIVSCSLVVNAVLSAVLLQLQLTAIFFLAALLIGLAVCLYYGSP
Rat NLGLYAGSGPGPGFLEGFSGWAVLVVLNQAVNGLLMSAVMKHGSSITRLFIVSCSLVVNAVLSAVLLQLQLTATFFLAALLIGLAVCLYYGSP
Human NLGLHAGGGSGPGLLEGFSGWAALVVLSQALNGLLMSAVMKHGSSITRLFVVSCSLVVNAVLSAVLLRLQLTAAFFLATLLIGLAMRLYYGSR

Figure EV6. SLC35A4 induction drives coordinated upregulation of secretory and cytosolic chaperones.

(**A**) STED nanoscopy analysis of mitochondrial cristae morphology in HeLa cells overexpressing SLC35A4 stained with PK Mito-Orange dye. Scale bar, 2 µm. (**B**) Representative TEM images of mitochondria from HeLa cells stably expressing GFP (control) or SLC35A4. Scale bar, 200 nm. (**C**) Quantitative analysis of mitochondrial cristae morphology based on TEM images from stable HeLa cell lines transduced with either GFP (control) or SLC35A4. Bar chart showing percentage of mitochondrial cristae categories ($n = 53$ mitochondria for control; $n = 82$ mitochondria for SLC35A4 overexpressing cells). P values were calculated using Fisher's exact test based on raw counts ($P = 0.5668$). (**D**) Predicted interaction confidence scores (mean PAE) between SLC35 family transporters and various nucleotide sugar substrates, as modeled by AlphaFold3. (**E**) Correlation analysis of AlphaFold3-predicted transporter-substrate binding scores (ipTM). (**F**) Phylogenetic tree of the human SLC35A subfamily constructed from protein sequences. (**G**) Evolutionary conservation of the seven substrate-binding residues across species, mapped onto the SLC35A4 structure. (**H**) Differential gene expression profiling in SLC35A4-overexpressing versus control in HEK293T cells ($n = 3$). Volcano plot highlights mitochondrial-localized genes (red) identified by Benjamini–Hochberg-adjusted Wilcoxon rank-sum test.

 © The Author(s)

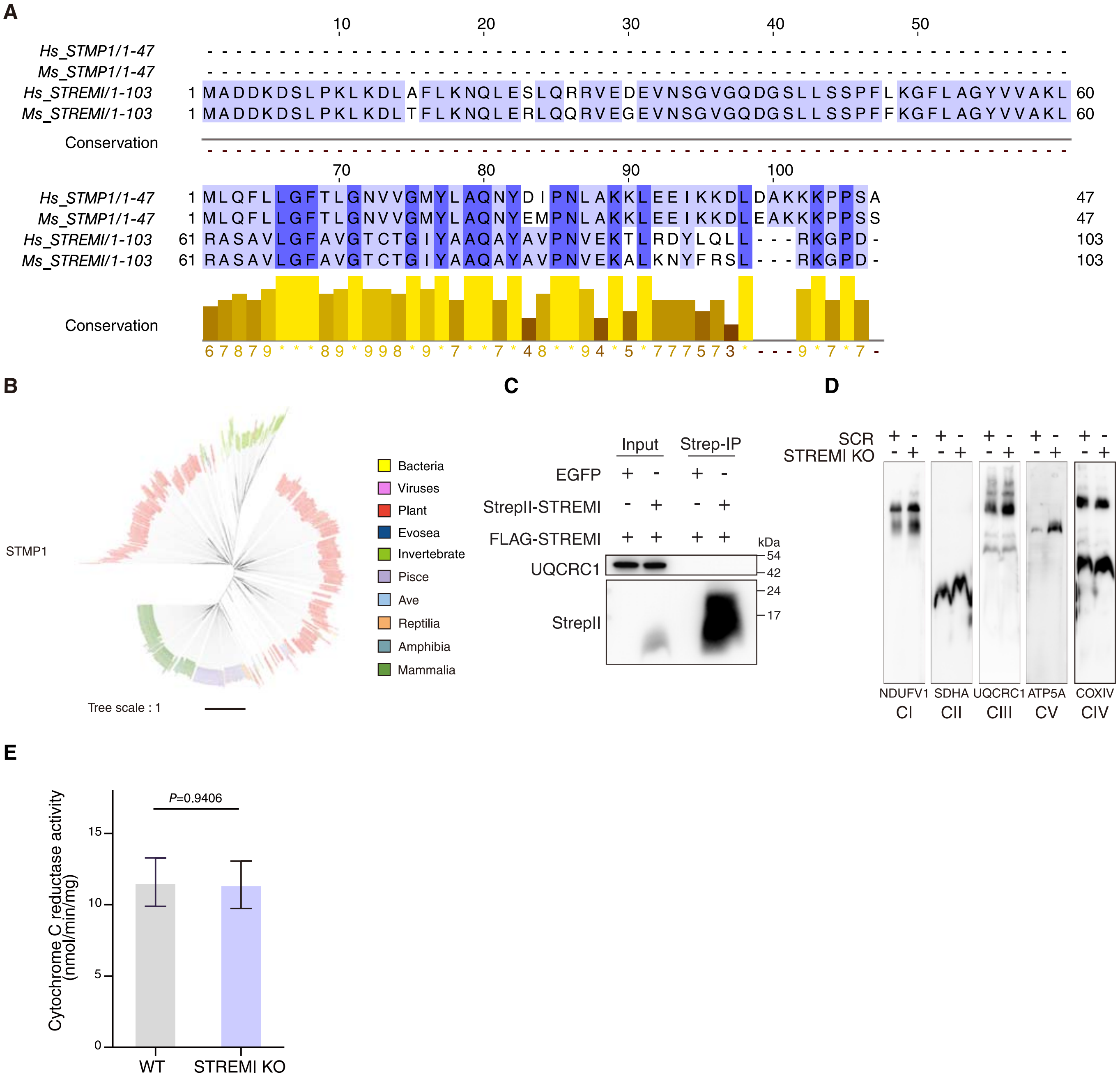

Figure EV7. Evolutionary analysis reveals the origin of gene symbiosis.

(**A**) Protein sequence alignment of human and murine STREMI and STMP1. (**B**) Phylogenetic tree of STMP1 homologs constructed using the maximum likelihood method. (**C**) Immunoprecipitation of Strep tagged STREMI, followed by immunoblotting for the CIII component UQCRC1. (**D**) Assembly of the electron transport chain (ETC) complexes analyzed by blue native-PAGE in STREMI knockout (KO) HEK293T cells. (**E**) CIII enzymatic activity in mitochondria from WT and STREMI KO HEK293T cells, normalized to total protein. Data are presented as mean ± SEM ($n = 4$). P values are from two-tailed unpaired t tests ($P = 0.9406$).

© The Author(s)

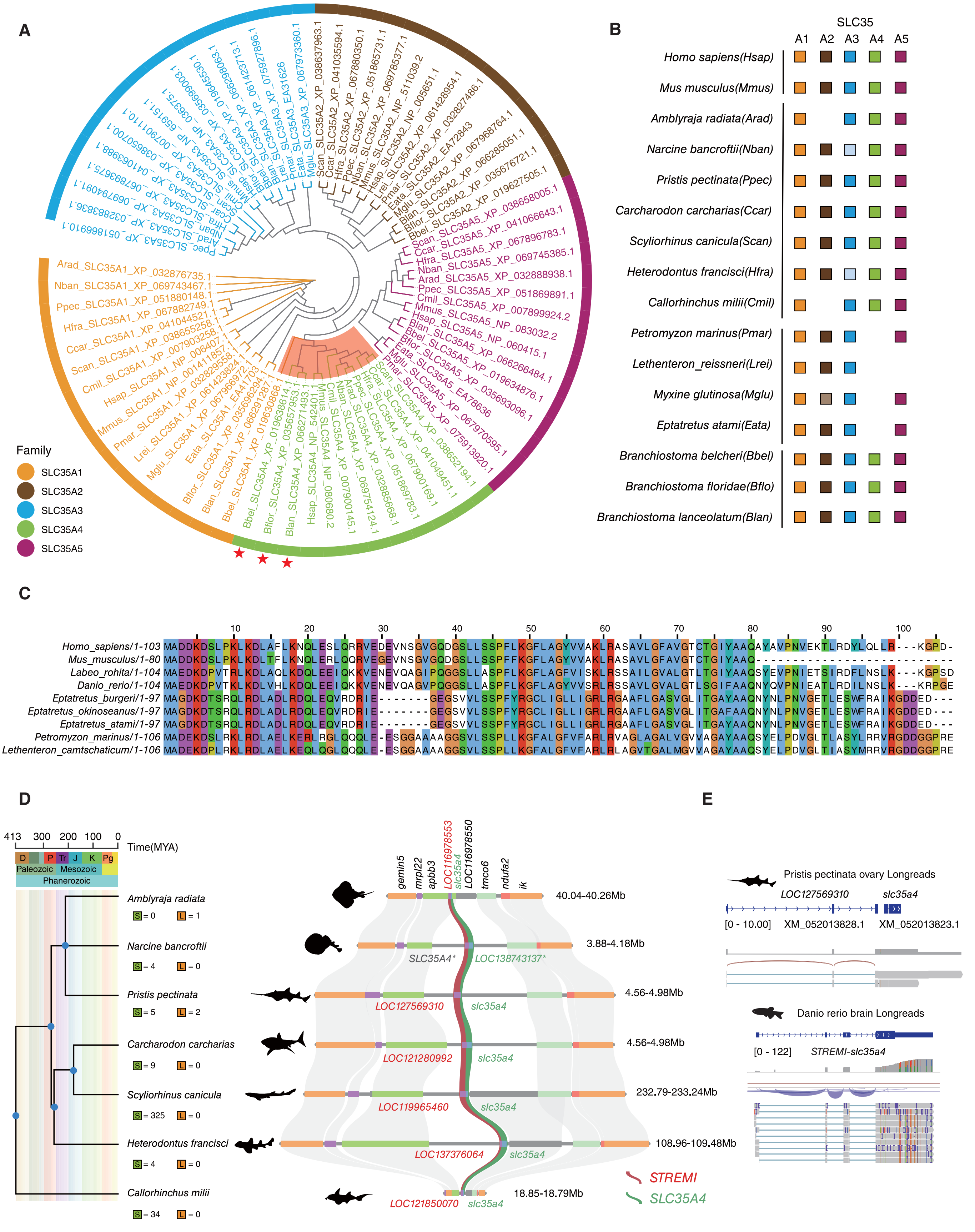
A
Family
SLC35A1
SLC35A2
SLC35A3
SLC35A4
SLC35A5
B
SLC35
A1 A2 A3 A4 A5
Homo sapiens(Hsap)
Mus musculus(Mmus)
Amblyraja radiata(Arad)
Narcine bancroftii(Nban)
Pristis pectinata(Ppec)
Carcharodon carcharias(Ccar)
Scyliorhinus canicula(Scan)
Heterodontus francisci(Hfra)
Callorhinchus milii(Cmil)
Petromyzon marinus(Pmar)
Lethenteron_reissneri(Lrei)
Myxine glutinosa(Mglu)
Eptatretus atami(Eata)
Branchiostoma belcheri(Bbel)
Branchiostoma floridae(Bflo)
Branchiostoma lanceolatum(Blan)
C
Homo_sapiens/1-103
Mus_musculus/1-80
Labeo_rohita/1-104
Danio_rerio/1-104
Eptatretus_burgeri/1-97
Eptatretus_okinoseanus/1-97
Eptatretus_atami/1-97
Petromyzon_marinus/1-106
Lethenteron_camtschaticum/1-106
D
413 300 200 100 0
Time(MYA)
Paleozoic Mesozoic
Phanerozoic
Amblyraja radiata
Narcine bancroftii
Pristis pectinata
Carcharodon carcharias
Scyliorhinus canicula
Heterodontus francisci
Callorhinchus milii
gemin5
mrpl22
apbb3
LOC116978553
slc35a4
LOC116978550
tmco6
ndufa2
ik
40.04-40.26Mb
3.88-4.18Mb
SLC35A4*
LOC138743137*
4.56-4.98Mb
LOC127569310
slc35a4
LOC121280992
232.79-233.24Mb
LOC119965460
108.96-109.48Mb
LOC137376064
18.85-18.79Mb
LOC121850070
STREMI
SLC35A4
E
Pristis pectinata ovary Longreads
LOC127569310
slc35a4
[0 - 10.00]
XM_052013828.1
XM_052013823.1
Danio rerio brain Longreads
[0 - 122]
STREMI-slc35a4

 © The Author(s)

Figure EV8. Evolutionary analysis of STREMI and SLC35A4 homologs in chordates.

(**A**) Protein phylogenetic tree of SLC35 family members across lancelet species and vertebrates. Branches are color-coded by gene family, with the SLC35A4 clade highlighted; red stars indicate SLC35A4 in lancelet species. (**B**) Presence–absence matrix showing the distribution of SLC35 homologs across major chordate and vertebrate lineages. (**C**) Multiple sequence alignment of representative STREMI homologs highlighting conserved residues across species. (**D**) Conserved synteny of the *STREMI–SLC35A4* locus in representative cartilaginous fishes, integrated with species phylogeny and available RNA-seq datasets (S short-read, L long-read). Asterisks indicate likely annotation errors in NCBI genome records, in which the names of *slc35a4* and the uORF-encoded *STREMI* homolog (*LOC138743137*) appear to be swapped. (**E**) Long-read Iso-Seq evidence supporting bicistronic transcription of the *STREMI-SLC35A4* locus in *Pristis pectinata* and *Danio rerio*. Gene models and mapped reads illustrate the shared transcript structure.

© The Author(s)
