# Supplementary material for: STREMI: a dual-function upstream ORF-encoded regulator of mitochondrial cristae architecture
Source: EMBO Rep. 2026 May 2;27(12):3303–39. doi: 10.1038/s44319-026-00783-8 (PMC13303939; doi:10.1038/s44319-026-00783-8)
Supplement: Supplementary file 1 — Peer Review File [file 44319_2026_783_MOESM1_ESM.pdf]

EMBO reports

STREMI: A Dual-Function Upstream ORF-Encoded Regulator of Mitochondrial Cristae Architecture

Ruiyang Guo, Yabo Guo, Runguo Shu, Jiajia Qian, Jiawei Wang, Ruobing Li, Ti Qin, Ziyi Wang, Hongtao Tian, Mengchen Wu, Long Zhou, Xiaogang Guo, and Shan Zhang

Corresponding author(s): Shan Zhang (shanzhang@zju.edu.cn) , Xiaogang Guo (gxg22222@zju.edu.cn), Long Zhou (longzhou@zju.edu.cn)

Review Timeline:

Submission Date:	4th Oct 25
Editorial Decision:	14th Nov 25
Revision Received:	16th Feb 26
Editorial Decision:	24th Mar 26
Revision Received:	31st Mar 26
Accepted:	9th Apr 26

Editor: Esther Schnapp

Transaction Report:

(Note: With the exception of the correction of typographical or spelling errors that could be a source of ambiguity, letters and reports are not edited. Depending on transfer agreements, referee reports obtained elsewhere may or may not be included in this compilation. Referee reports are anonymous unless the Referee chooses to sign their reports.)

1st Editorial Decision **14th Nov 2025**

Dear Dr. Zhang,

Thank you for the submission of your manuscript to EMBO reports. We have now received the full set of referee reports that is pasted below.

As you will see, all referees acknowledge that the findings are interesting. However, they also have a number of suggestions for how the study could be improved, and I think all suggestions are good. It would be great if you could address all points raised, but I am uncertain how much you can or are willing to do to improve the evolutionary analysis, for example, or in how much more depth the mouse model can be characterised. It would be helpful if you could send me a proposed revision plan (as point-by-point response) to explain what you can do, and we can take it as a basis to discuss the exact revision requirements. This could also be done in a video chat, if you like.

I would thus like to invite you to revise your manuscript with the understanding that the referee concerns must be fully addressed and their suggestions taken on board. Please address all referee concerns in a complete point-by-point response. Acceptance of the manuscript will depend on a positive outcome of a second round of review. It is EMBO reports policy to allow a single round of major revision only and acceptance or rejection of the manuscript will therefore depend on the completeness of your responses included in the next, final version of the manuscript.

We realize that it is difficult to revise to a specific deadline. In the interest of protecting the conceptual advance provided by the work, we recommend a revision within 3 months (14th Feb 2026). Please discuss the revision progress ahead of this time with the editor if you require more time to complete the revisions.

IMPORTANT NOTE: we perform an initial quality control of all revised manuscripts before re-review. Your manuscript will FAIL this control and the handling will be DELAYED if the following APPLIES:
1) A data availability section providing access to data deposited in public databases is missing. If you have not deposited any data, please add a sentence to the data availability section that explains that.
2) Your manuscript contains statistics and error bars based on n=2. Please use scatter blots in these cases. No statistics should be calculated if n=2.

When submitting your revised manuscript, please carefully review the instructions that follow below. Failure to include requested items will delay the evaluation of your revision.

1) a .docx formatted version of the manuscript text (including legends for main figures, EV figures and tables). Please make sure that the changes are highlighted to be clearly visible.

2) individual production quality figure files as .eps, .tif, .jpg (one file per figure). See https://wol-prod-cdn.literatumonline.com/pb-assets/embo-site/EMBOPress_Figure_Guidelines_061115-1561436025777.pdf for more info on how to prepare your figures.

3) We replaced Supplementary Information with Expanded View (EV) Figures and Tables that are collapsible/expandable online. A maximum of 5 EV Figures can be typeset. EV Figures should be cited as 'Figure EV1, Figure EV2" etc... in the text and their respective legends should be included in the main text after the legends of regular figures.

- For the figures that you do NOT wish to display as Expanded View figures, they should be bundled together with their legends in a single PDF file called *Appendix*, which should start with a short Table of Content. Appendix figures should be referred to in the main text as: "Appendix Figure S1, Appendix Figure S2" etc. See detailed instructions regarding expanded view here: <https://www.embopress.org/page/journal/14693178/authorguide#expandedview>

- Additional Tables/Datasets should be labeled and referred to as Table EV1, Dataset EV1, etc. Legends have to be provided in a separate tab in case of .xls files. Alternatively, the legend can be supplied as a separate text file (README) and zipped together with the Table/Dataset file.

4) a .docx formatted letter INCLUDING the reviewers' reports and your detailed point-by-point responses to their comments. As part of the EMBO Press transparent editorial process, the point-by-point response is part of the Review Process File (RPF), which will be published alongside your paper.

5) a complete author checklist, which you can download from our author guidelines <https://www.embopress.org/page/journal/14693178/authorguide>. Please insert information in the checklist that is also reflected in the manuscript. The completed author checklist will also be part of the RPF.

6) Please note that all corresponding authors are required to supply an ORCID ID for their name upon submission of a revised manuscript (<https://orcid.org/>). Please find instructions on how to link your ORCID ID to your account in our manuscript tracking system in our Author guidelines

<https://www.embopress.org/page/journal/14693178/authorguide#authorshipguidelines>

7) Before submitting your revision, primary datasets produced in this study need to be deposited in an appropriate public database (see https://www.embopress.org/page/journal/14693178/authorguide#datadeposition). Please remember to provide a reviewer password if the datasets are not yet public. The accession numbers and database should be listed in a formal "Data Availability" section placed after Materials & Method (see also https://www.embopress.org/page/journal/14693178/authorguide#datadeposition). Please note that the Data Availability Section is restricted to new primary data that are part of this study. * Note - All links should resolve to a page where the data can be accessed. *
If your study has not produced novel datasets, please mention this fact in the Data Availability Section.

8) At EMBO Press we ask authors to provide source data for the main manuscript figures. You will receive a separate email with instructions for providing source data with your revised manuscript, including how to upload and organize the files.

9) Our journal also encourages inclusion of *data citations in the reference list* to directly cite datasets that were re-used and obtained from public databases. Data citations in the article text are distinct from normal bibliographical citations and should directly link to the database records from which the data can be accessed. In the main text, data citations are formatted as follows: "Data ref: Smith et al, 2001" or "Data ref: NCBI Sequence Read Archive PRJNA342805, 2017". In the Reference list, data citations must be labeled with "[DATASET]". A data reference must provide the database name, accession number/identifiers and a resolvable link to the landing page from which the data can be accessed at the end of the reference. Further instructions are available at https://www.embopress.org/page/journal/14693178/authorguide#referencesformat

10) Regarding data quantification (see Figure Legends: https://www.embopress.org/page/journal/14693178/authorguide#figureformat)

The following points must be specified in each figure legend:

- the name of the statistical test used to generate error bars and P values,

- the number (n) of independent experiments (please specify technical or biological replicates) underlying each data point,

- the nature of the bars and error bars (s.d., s.e.m.),

- If the data are obtained from n Program fragment delivered error ``Can't locate object method "less" via package "than" (perhaps you forgot to load "than"?) at //ejpvfs23/sites23b/embor_www/letters/embor_decision_revise_and_review.txt line 56.' 2, use scatter blots showing the individual data points.

Discussion of statistical methodology can be reported in the materials and methods section, but figure legends should contain a basic description of n, P and the test applied.

- Please also include scale bars in all microscopy images.

11) The journal requires a statement specifying whether or not authors have competing interests (defined as all potential or actual interests that could be perceived to influence the presentation or interpretation of an article). In case of competing interests, this must be specified in your disclosure statement. Further information: https://www.embopress.org/competing-interests

12) All Materials and Methods need to be described in the main text using our 'Structured Methods' format, which is required for all research articles. According to this format, the Methods section includes a separate Reagents and Tools Table file (listing key reagents, experimental models, software and relevant equipment and including their sources and relevant identifiers) and a Methods and Protocols section describing the methods using a step-by-step protocol format. The aim is to facilitate adoption of the methodologies across labs. More information on how to adhere to this format as well as a downloadable template (.docx) for the Reagents and Tools Table can be found in our author guidelines: https://www.embopress.org/page/journal/14693178/authorguide#structuredmethods.

An example of a Method paper with Structured Methods can be found here: https://www.embopress.org/doi/full/10.1038/s44320-024-00037-6#sec-4

We would also welcome the submission of cover suggestions, or motifs to be used by our Graphics Illustrator in designing a cover.

As part of the EMBO publication's Transparent Editorial Process, EMBO reports publishes online a Review Process File (RPF) to accompany accepted manuscripts. This File will be published in conjunction with your paper and will include the referee reports, your point-by-point response and all pertinent correspondence relating to the manuscript.

You are able to opt out of this by letting the editorial office know (emboreports@embo.org). If you do opt out, the Review Process File link will point to the following statement: "No Review Process File is available with this article, as the authors have chosen not to make the review process public in this case."

I look forward to seeing a revised form of your manuscript when it is ready.

Best regards,
Esther

Esther Schnapp, PhD
Senior Editor
EMBO reports

Referee #1:

SLC35A4-MP is a 103 aa microprotein (Elife. 2015. PMID: 25621764) which is located at inner mitochondrial membrane (Protein Sci. 2025. PMID: 40545711) and plays important roles in cellular respiration and ATP generation (J Mol Biol. 2024. PMID: 38580077), and tissue lipid metabolism (Sci Adv. 2025. PMID: 40880489). In this manuscript, differentiating from the previous studies, Ruiyang Guo et al further provide strong evidence that SLC35A4-MP regulates mitochondrial cristae morphogenesis. Interestingly, SLC35A4-MP can directly mediate the stress-responsive translation of its canonical partner SLC35A4. By evolutionary analyses, the authors found that SLC35A4-MP and canonical SLC35A4 are originally independent genes and a retroposition event led to the insertion of canonical SLC35A4 into SLC35A4-MP locus. The manuscript has strong novelty but still needs improvement to be published.

Major concerns:

1. Please provide background information on SLC35A4 and SLC35A4-MP in the Introduction (eg Rocha papers). It also does not acknowledge that SCL35A4 uORF has been formally annotated as a protein since 2018, when it achieved PE1 status with the Human Proteome Project (Omenn et al, J Proteome Res, 2018; PMID 30099871).

2. The name SUMI should be approved by the HUGO Gene Nomenclature Committee in order for this term to be published. Have the authors obtained this approval? Otherwise it will not be useful to the research community.

3. When you knock out of SUMI (SLC35A4-MP) in HeLa cells (line 168), how do you not knock out the classical CDS (or canonical SLC35A4 protein, line 125) as the canonical SLC35C4 shares the same 5' UTR with SUMI (SLC35A4-MP)?

4. It might be not appropriate to conclude that mutating glycine motifs significantly destabilized SUMI with current data (Fig. 4H), unless you evaluate the protein degradation/stability of SUMI.

5. This study mainly focuses on the functional study of SLC35A4-MP instead of canonical SLC35A4, so "SLC35A4 induction drives coordinated upregulation of secretory and cytosolic chaperones" seems irrelevant to the title of this manuscript.

6. The authors provide strong evidence that SUMI oligomerizes with MIC10 but need to provide the explanation why SUMI oligomerizes with MIC10 to fine-tune the architecture of cristae, especially when using the word "fine-tune".

7. There is a rationale gap between Figure 4J-4L and the conclusion that SUMI can oligomerize together with MIC10 through their shared topology and motif. The authors need to explain why "through their shared topology and motif".

8. Do the authors think that SLC35A4-MP and SLC35A4 main protein get translated from the same individual mRNA, or does a given mRNA only translate one of these two potential proteins?

Minor concerns:
9. In Figure 2F, immunoblotting of MIC10 needs repeat. The band is not clear.
10. Please specify the abbreviations when it is first shown in the text, such as OMM, MICOS, TEM etc.
11. The authors should note that there are important uORF proteins that they have not referenced here, including ones with importance in the mitochondria, eg. PMID 33406399, 34593629

Referee #2:

In this manuscript, Guo R., Guo Y., and colleagues investigate a bicistronic transcript of SLC35A4 that encodes both a uORF-derived mitochondrial microprotein, termed SUMI (previously SLC35A4-MP), and the canonical Golgi-resident transporter SLC35A4. Using long-read sequencing, proteogenomics, and functional assays, the authors identify 14 bicistronic human transcripts and focus on SLC35A4 as a representative model. They show that SUMI localizes to the inner mitochondrial membrane, interacts with the MICOS subunit MIC10, and contributes to mitochondrial cristae morphogenesis. The study also proposes that the uORF functions as a stress-responsive translational regulator of the downstream SLC35A4 ORF and introduces an evolutionary "gene symbiosis" model for the origin of bicistronic genes through retroposition.
Overall, the manuscript is well written, logically structured, and technically rigorous. It provides a conceptual framework for the evolution of dual-function transcripts. However, several of the biological findings, particularly those concerning mitochondrial localization, cristae defects, and metabolic consequences, have been previously reported by Rocha et al. (J. Mol. Biol. 2024; Sci. Adv. 2025) and Ajala et al. (Protein Sci. 2025). These earlier studies already demonstrated that SLC35A4-MP is a conserved mitochondrial microprotein whose loss perturbs respiration, cristae organization, and fatty-acid oxidation in brown adipose tissue. In the present manuscript, the discussion of these prior works is vague and, in the case of Rocha et al. (Sci. Adv. 2025), the reference is entirely absent despite multiple overlapping claims. I understand that this omission is likely due to the very recent publication of that paper (August 2025). To allow a fair assessment of novelty, the authors must explicitly acknowledge all the earlier literature and delineate which aspects are genuinely new. In my view, the mechanistic dissection of SUMI-MIC10 interaction, the determination of membrane topology, and the proposed evolutionary model of bicistronic gene origin appear to be some main novel contributions. I also have concerns regarding the nomenclature and the evolutionary interpretation, which are detailed below.

Major comments
- The primary issue is the insufficient recognition of previously published results. The roles of SLC35A4-MP in mitochondrial respiration, oxidative stress responses, and cristae disruption have already been established, including the brown adipose tissue phenotypes observed under cold exposure and high-fat diet in Rocha et al. (Sci. Adv. 2025). The authors should clearly what biological aspects are not novel and fairly specify their new contributions.
- Regarding the evolutionary analysis, the manuscript reports the presence of two separate genes in sea lamprey and two consecutive genes in thorny skate, which is indeed an intriguing observation, as previous studies traced conservation only up to zebrafish. However, it is important to verify whether RNA-seq data support the independent transcription of these loci in thorny skate, or whether this might be an annotation artifact and the gene is in fact bicistronic in this species as well. Clarification is also needed for sea lamprey: although the proposed mechanism is interesting, the evidence is currently limited to one species, and potential issues in genome quality or annotation could influence the result. Is the same locus configuration retained in the 2025 sea lamprey genome assembly? Does the annotated SLC35A4 homolog in lamprey actually correspond to SLC35A3? According to the authors' hypothesis, could retroposition have been initially driven by the expression of SLC35A3? It would also be informative to examine whether SLC35A4-MP/SUMI is positionally conserved (e.g. using synteny) across lamprey, thorny skate and other vertebrate genomes, and whether orthologous sequences exist in other lampreys such as Lethenteron camtschaticum and in hagfish. Even if the corresponding protein is unannotated in protein databases, one might expect its transcription/sequence to be detectable in the genomes of these species, likely supporting a non-bicistronic configuration if the retroposition event happened after the split of lamprey/hagfish and the rest of vertebrates. Since the proposed retroposition mechanism is a central novelty of the manuscript, addressing these points would greatly strengthen the evidence for the evolutionary model.
- Finally, nomenclature consistency is crucial. Previous literature consistently refers to the peptide as SLC35A4-MP. Introducing a new name, "SUMI," without HGNC approval could generate unnecessary confusion in the field. I recommend retaining SLC35A4-MP as the primary name throughout the text, mentioning "SUMI" only as an alternative descriptor. However, if the authors consider that the new name "SUMI" better reflects a specific mechanistic or functional property not captured by the previous designation, they should explicitly justify its introduction and clarify its rationale in the manuscript.

Minor comments
- The evolutionary conservation of the SUMI peptide across is visualized in Figure 1 but restricted to a subset of species. Including a full multiple-sequence alignment including all species with SUMI in Fig. 7D and highlighting conserved motifs, particularly the GxxxG oligomerization motifs, and reporting percentage sequence identity across representative species would provide valuable context, especially for comparison with sea lamprey.
- Statistical analysis details should be clarified. Many figures (for example, Figure 5) display multiple p-values without apparent indication of multiple-testing correction. The authors should confirm that all p-values are properly adjusted and specify the correction method used.
- Line 466, the definition of a "good" Ribo-seq signal should be quantified. The thresholds used to support translation should be explicitly stated in the Methods section.
- Line 753: Was a similar E-value cutoff used as in the previous section? Please clarify what is meant by "adjacent." Was there a defined maximum distance threshold?
- Figure 7D: This information is valuable but should be complemented by a supplementary table listing all identified homologs in other species for the seven gene pairs, including their names/IDs, E-values, and percentage identities. Do the authors know why SUMI (LOC116958356) is annotated as DNPH1 in Ensembl for sea lamprey?

Referee #3:

The authors address the evolution of bicistronic ORFs and focus specifically on the role of a microprotein that is encoded upstream of the SLC35A4. This novel mitochondrial protein, termed SUMI, and its organization at the genomic level was studied already by several other groups (Ajala et al., 2025; Rocha et al., 2024; Rocha et al. 2025) and also by the same group (Zhang et al., 2020) in recent studies earlier - as the authors correctly point out. Thus, the novelty of this study is not in identifying a novel microprotein which affects mitochondrial respiration. It is also not in identifying an increasing number of bicistronic ORF having mitochondrial functions. The novelty is that this study shows convincingly that SUMI is interacting with the MICOS complex and that it assists in shaping cristae. This study helps to resolve several discrepancy and uncertainties regarding the localisation of SUMI and identify the SLC35A4-MP (SUMI) as a bona fide subunit of the MICOS complex. Moreover, the authors even generate a SUMI knockout mouse model. The authors provide also interesting information on the possible evolution of this bicistronic ORF and its stress-dependent regulation.
Overall, I think it is an important study. Yet, I think that many aspects of the already published studies are not really novel and take too much space in the manuscript. The mechanistic role of this novel MICOS-interaction protein is partly addressed but not in much detail. The phenotype of the mouse is not grossly described and need further emphasis as well as the role of SUMI on cristae junction formation and on the MICOS complex itself.

Major points.
1. Is SUMI localized at cristae junctions. Fig. S4A and the partial overlap with MIC60 is not convincing. Can the authors perform immune-gold labelling?
2. Does SUMI affect glycolysis and fatty acid oxidation in cells (or tissues)?
3. More detailed phenotyping of the mouse model is missing in my opinion. Please elaborate more.
4. What is the rough stoichiometry of SUMI in the MICOS complex and does it change under different metabolic or stress conditions? In this regard it would be interesting to show the unbound fractions of the IP shown in Fig. 2FG.
5. What is the native molecular weight of SUMI and does the MICOS complex change in size upon deletion of SUMI?

Minor:
4. Typo in line 203 "...MIC60, supporting its residence in the IMM and interaction with the MICOS complex (S4B Fig).": correct to "...(S4A Fig.)."

Point-by-point response

In the revised manuscript, we have sought to address all points raised by the reviewers. To facilitate evaluation, we have embedded the relevant figure panels directly in this response letter and labeled them using the panel identifiers from the revised manuscript. In addition, a small number of figures designated as Figure RX are provided solely to address specific reviewer comments and are not included in the main manuscript.

We are pleased that your suggestions helped us to present a more complete and coherent story. In particular, we now provide stronger evidence for the molecular mechanism by which the SLC35A4 uORF-encoded peptide contributes to MICOS assembly, as well as a clearer account of the evolutionary events that gave rise to the bicistronic transcript. We sincerely thank you for your thoughtful comments and constructive suggestions.

Referee #1:

SLC35A4-MP is a 103 aa microprotein (Elife. 2015. PMID: 25621764) which is located at inner mitochondrial membrane (Protein Sci. 2025. PMID: 40545711) and plays important roles in cellular respiration and ATP generation (J Mol Biol. 2024. PMID: 38580077), and tissue lipid metabolism (Sci Adv. 2025. PMID: 40880489). In this manuscript, differentiating from the previous studies, Ruiyang Guo et al further provide strong evidence that SLC35A4-MP regulates mitochondrial cristae morphogenesis. Interestingly, SLC35A4-MP can directly mediate the stress-responsive translation of its canonical partner SLC35A4. By evolutionary analyses, the authors found that SLC35A4-MP and canonical SLC35A4 are originally independent genes and a retroposition event led to the insertion of canonical SLC35A4 into SLC35A4-MP locus. The manuscript has strong novelty but still needs improvement to be published.

We sincerely thank you for the thoughtful evaluation of our manuscript and for highlighting its key novelties. We greatly appreciate the constructive suggestions provided. We have revised the manuscript accordingly and supplied with experimental evidences that support our claim.

Major concerns:

1. Please provide background information on SLC35A4 and SLC35A4-MP in the Introduction (eg Rocha papers). It also does not acknowledge that SCL35A4 uORF has been formally annotated as a protein since 2018, when it achieved PE1 status with the Human Proteome Project (Omenn et al, J Proteome Res, 2018; PMID 30099871).

We have revised the Introduction as suggested. Below is the section we incorporated, see line 75-90:

“To date, only a few human uORFs have been experimentally validated as encoding biologically active polypeptides (Hofman *et al*, 2024; Huang *et al*, 2021; Jayaram *et al*, 2021; Rathore *et al*, 2018; Rensvold *et al*, 2022). Among these, the 5’UTR of SLC35A4 was first reported in 2013 to encode a 103-amino acid microprotein and was designated PE1 by the Human Proteome Project in 2018(Omenn *et al*, 2018; Vanderperre *et al*, 2013). Our group subsequently identified this polypeptide as a mitochondrially localized microprotein, a finding later corroborated by multiple independent studies (Ajala *et al*, 2025; Liu *et al*, 2024; Yang *et al*, 2024; Zhang *et al*, 2020). More recent work has implicated this microprotein in maintaining mitochondrial architecture, oxidative stress responses, and respiratory function in both cultured cells and mouse models (Ajala *et al*., 2025; Rocha *et al*, 2024; Rocha *et al*, 2025). In mice, its deficiency

alters mitochondrial lipid composition under a high-fat diet and impairs fatty acid oxidation (Rocha *et al.*, 2025). Despite this growing interest, the precise molecular function of this uORF-encoded microprotein remains unresolved. Moreover, the functional and regulatory interplay between the upstream and downstream ORFs—and their evolutionary origin—has not been systematically addressed, leaving the broader biological significance of this protein-coding uORF unclear. "

2. The name SUMI should be approved by the HUGO Gene Nomenclature Committee in order for this term to be published. Have the authors obtained this approval? Otherwise it will not be useful to the research community.

We thank you for this considerate suggestion. We have been in discussion with HGNC regarding the nomenclature of this protein-coding uORF. At present, this uORF has not been assigned an approved symbol by HGNC. Within the bicistronic transcript, only the main ORF is annotated by HGNC as SLC35A4 (HGNC:20753).

Importantly, HGNC has established precedents for assigning distinct gene symbols to separate coding sequences within a single bicistronic transcript. For example, the uORF and main ORF of PIGY are named PYURF (HGNC:44317) and PIGY (HGNC:28213), respectively. Similarly, the uORF and main ORF of SNRPN are named SNURF (HGNC:11171) and SNRPN (HGNC:11164), respectively. Consistent with this, in CCDS and Ensembl, this uORF is still recorded with a placeholder designation (e.g., "novel protein"; Table R1).

In our communications with HGNC, they agreed that this protein-coding ORF should receive an official name (see the email thread provided in the Supplementary File). However, they noted that our originally proposed name, SUMI, is not ideal because it is a poor search term and overlaps with an existing word; for example, a PubMed search for "SUMI" returns over 4,000 results. HGNC therefore suggested two alternatives: SMICI ("small integral membrane MICOS interactor") and STREMI ("SLC35A4 stress response regulating MICOS interactor"). We favor STREMI because it references SLC35A4 and captures the function we describe—regulation of the stress-responsive expression of SLC35A4. Accordingly, we have updated the manuscript to use **STREMI** throughout.

Final approval typically benefits from alignment among groups that have characterized the ORF (including our own prior work on this locus in our 2020 Nature Communications paper). At present, HGNC has paused further action at our request to maintain confidentiality prior to acceptance. In summary, our goal is to provide this currently unnamed ORF with a clear, functionally grounded designation. If you have a specific naming suggestion, we would be happy to consider it and adopt it if HGNC agrees that it is appropriate.

Table R1. Annotation status of the SLC35A4 uORF in databases

Database	Name	Identifier	Link
HGNC	Unannotated	Unannotated	Not available
CCDS	LOC131768270	CCDS93794	https://www.ncbi.nlm.nih.gov/CCDS/CcdsBrowse.cgi?REQUEST=CCDS&DATA=CCDS93794
Ensembl	novel protein	ENSG00000293600	https://useast.ensembl.org/Homo_sapiens/Transcript/Summary?db=core;g=ENSG00000293600;r=5:140564819-140569100;t=ENST00000715679

3. When you knock out of SUMI (SLC35A4-MP) in HeLa cells (line 168), how do you not knock out the classical CDS (or canonical SLC35A4 protein, line 125) as the canonical SLC35C4 shares the same 5' UTR with SUMI (SLC35A4-MP)?

The STREMI knockout was generated using a single gRNA targeting the N-terminal region of STREMI. Amplicon sequencing revealed two frameshift alleles (-2 and -1), while leaving the protein-coding sequence of the downstream canonical ORF (SLC35A4) intact (Fig. EV2H-J).

To determine whether SLC35A4 transcript abundance was altered in this line, we performed qPCR to assess steady-state mRNA levels. qPCR assays using primer pairs spanning either the 5’ UTR or the SLC35A4 coding region indicated that overall transcript abundance remained largely unchanged in the knockout cells (Fig. EV2K). Consistent with this, treatment with an inhibitor of nonsense-mediated decay (NMD) did not significantly affect transcript levels (Fig. EV2L). Together, these data indicate that the frameshift mutation did not activate NMD and that the mutant transcript is preserved.

It is also important to note that the uORF start codon and Kozak context are unaltered in this mutant. Therefore, translation initiation at the mutated uORF is expected to remain active and to continue to strongly repress downstream SLC35A4 translation. Likewise, the stress-dependent upregulation mechanism for SLC35A4 is preserved, as it depends on initiation at the uORF start codon.

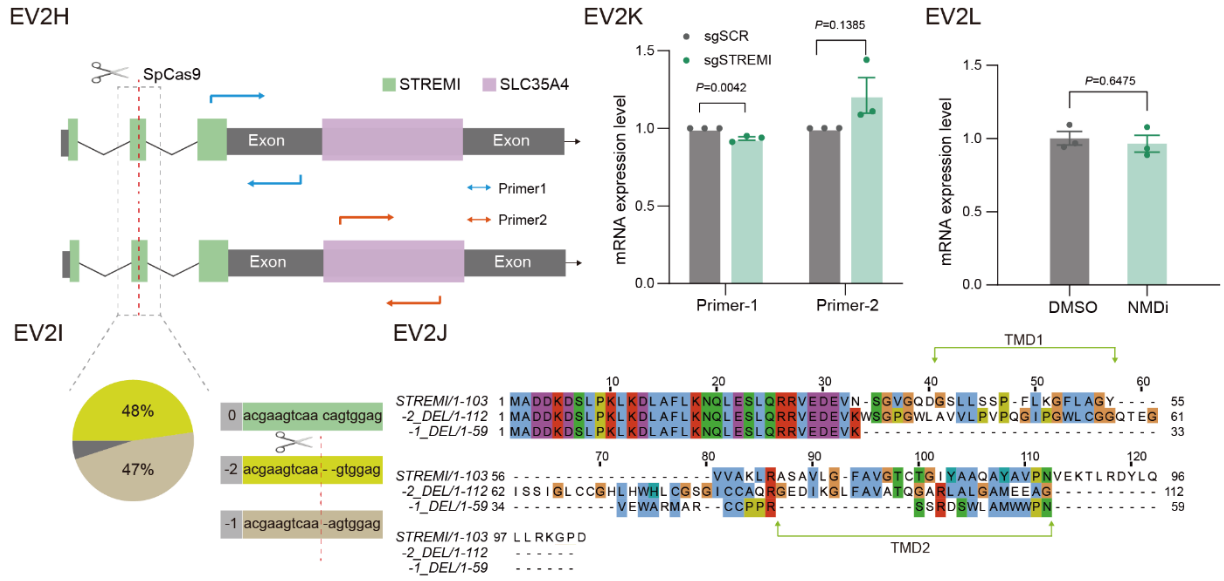

Mutation type and transcript abundance of the STREMI KO line in HeLa

(EV2H) Schematic diagram showing the locations of qPCR primer pairs used to assess transcript abundance.

(EV2I) Distribution of genome-editing outcomes following SpCas9 targeting of the STREMI coding sequence. The frequencies of the two predominant frameshift alleles (-2 and -1) are indicated.

(EV2J) Predicted translation products from WT and the two predominant frameshift alleles in the STREMI KO line, shown as a sequence alignment.

(EV2K) qPCR analysis of transcript levels in SCR and STREMI KO cells. Data are presented as mean ± SEM (n = 3 independent experiments). P values are from two-tailed unpaired t tests.

(EV2L) qPCR analysis of transcript levels in STREMI KO cells following NMD inhibitor (NMDI14) treatment for 12 h. Data are presented as mean ± SEM (n = 3 independent experiments). *P* values are from two-tailed unpaired t tests.

4. It might be not appropriate to conclude that mutating glycine motifs significantly destabilized SUMI with current data (Fig. 4H), unless you evaluate the protein degradation/stability of SUMI.

We thank you for raising this important point. To determine whether the low steady-state level of the glycine mutant reflects enhanced post-translational degradation, we assessed mutant stability by cycloheximide chase. We found that the original glycine mutant—where three glycine residues were substituted with alanine—was highly unstable, with a half-life of <4 h (Fig. 4H, I). We also examined a weaker mutant in which only a single glycine residue in the second transmembrane domain was replaced with alanine. This single-residue mutant remained markedly unstable, although it showed a modestly longer half-life (~4 h) than the triple mutant (Fig. 4J, K).

Given that STREMI is a mitochondrial inner membrane protein, we hypothesized that its quality control might involve OMA1, an inner-membrane protease reported to process MICOS subunits(Kaser *et al*, 2003; Tang *et al*, 2020). Consistent with this idea, the STREMI glycine mutants were partially stabilized in an OMA1 knockout line (Fig. EV4E-G). Collectively, these data support a critical role for the GxxxG glycine motif in maintaining STREMI stability and indicate that disruption of this motif promotes proteolytic turnover, at least in part via an OMA1-dependent pathway.

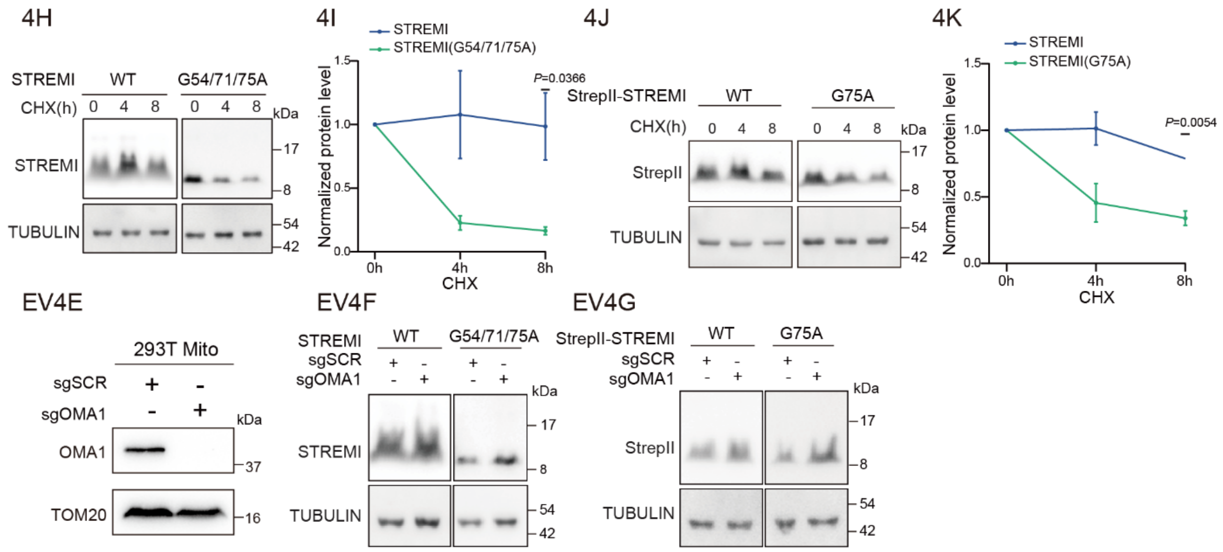

Protein stability of STREMI glycine-rich motif mutants
(4H) Cycloheximide-chase analysis of the STREMI triple glycine mutant (G54/71/75A).
(4I) Quantification of degradation kinetics from panel (H) (n = 3 per group). Data are mean ± SEM. *P* values were calculated using an unpaired two-tailed t test.
(4J) Cycloheximide-chase analysis of the STREMI single glycine mutant (G75A).
(4K) Quantification of degradation kinetics from panel (J) (n = 3 per group). Data are mean ± SEM. *P* values were calculated using an unpaired two-tailed t test.
(EV4E) Immunoblot analysis of OMA1 in mitochondrial lysates from SCR and OMA1 KO HEK293T cells.
(EV4F) Immunoblot analysis of STREMI (G54/71/75A) in control and OMA1 KO HEK293T cells.
(EV4G) Immunoblot analysis of StrepII-STREMI (G75A) in control and OMA1 KO HEK293T cells.

5. This study mainly focuses on the functional study of SLC35A4-MP instead of canonical SLC35A4, so "SLC35A4 induction drives coordinated upregulation of secretory and cytosolic chaperones" seems irrelevant to the title of this manuscript.

In this study, we are interested in both the molecular function of STREMI and the potential protein-level

interplay between STREMI and the downstream canonical ORF. Given that this bicistronic transcript encodes two membrane proteins with distinct organelle localizations— STREMI in mitochondria and SLC35A4 in the Golgi—it is tempting to speculate that such dual coding might mediate functional crosstalk between the two organelles. To explore this possibility, we examined whether SLC35A4 upregulation—induced by inhibitors of mitochondrial respiration (oligomycin) or ER proteostasis stress (thapsigargin) (Fig. 5G)—affects mitochondrial morphology or protein expression. Our data indicate that increased SLC35A4 protein levels have minimal impact on mitochondrial morphology or the expression of mitochondrial proteins (Fig. EV6B, C, H). Instead, SLC35A4 induction predominantly triggers upregulation of secretory-pathway and cytosolic chaperones.

These findings suggest that although STREMI and SLC35A4 share a transcript, their protein functions are not necessarily coupled. Consistently, our evolutionary analysis indicates that the two proteins originally evolved independently, and that an ancient retroposition event likely led to the emergence of this bicistronic transcript. Together, these analyses are integral to the complete characterization of this bicistronic transcript and help address readers' natural curiosity about the functional relationship between STREMI and SLC35A4.

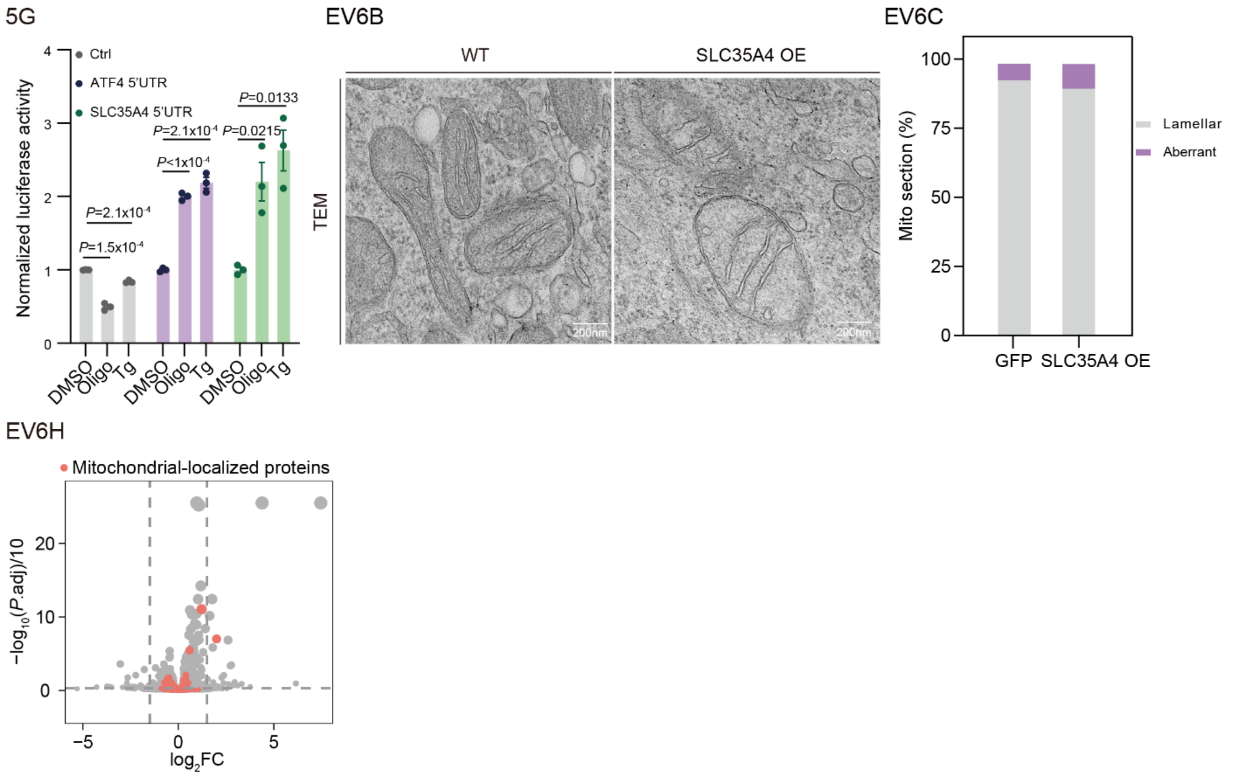

Expression and functional relationship between STREMI and SLC35A4
(5G) Translation regulation of downstream ORFs under integrated stress response (ISR) inducers oligomycin (Oligo) and thapsigargin (Tg). Data are presented as mean ± SEM (n = 3).
(EV6B) Representative TEM images of mitochondria from HeLa cells stably expressing GFP (control) or SLC35A4. Scale bar, 200 nm.
(EV6C) Quantitative analysis of mitochondrial cristae morphology based on TEM images from stable HeLa cell lines transduced with either GFP (control) or SLC35A4. Bar chart showing percentage of mitochondrial cristae categories (n=53 mitochondria for control; n=82 mitochondria for SLC35A4 overexpressing cells).
(EV6H) Differential gene expression profiling in SLC35A4-overexpressing versus control in HEK293T cells. Volcano plot highlights mitochondrial-localized genes (red) identified by Benjamini-Hochberg-adjusted Wilcoxon rank-sum test.

6. The authors provide strong evidence that SUMI oligomerizes with MIC10 but need to provide the explanation why SUMI oligomerizes with MIC10 to fine-tune the architecture of cristae, especially when using the word "fine-tune".

To further define the relationship between STREMI and MICOS, we examined MICOS assembly in cultured cells by BN-PAGE followed by second-dimension SDS-PAGE. Consistent with prior reports, we detected a high-molecular-weight MICOS-containing species migrating near the respirasome region, along with multiple MICOS assemblies in the ~500-700 kDa range (Fig. 4O, P) (Huynen *et al*, 2016). In addition, endogenous STREMI displayed a broad mobility distribution on BN-PAGE and substantially overlapped with MIC10 and MIC26 signals (Fig. 4N), in line with reports that MIC10-subcomplex components exist in a range of oligomeric states that contribute to membrane shaping(Bohnert *et al*, 2015; Guarani *et al*, 2015; Rampelt *et al*, 2018).

Loss of STREMI did not grossly remodel the overall MICOS assembly pattern, consistent with the largely unchanged steady-state abundance of MICOS subunits in the knockout cells (Fig. 4O). However, we reproducibly observed an increased relative abundance of lower-molecular-weight MICOS species in STREMI-deficient cells, suggesting a shift in assembly equilibrium toward smaller forms (Fig. 4O,P). Because previous studies indicate that the highest-molecular-weight MICOS-containing species also includes MIB components, these results further suggest that STREMI contributes to maintaining higher-order MICOS/MIB assemblies(Huynen *et al.*, 2016).

Together, these data support two non-mutually exclusive models. First, STREMI may act as an accessory component of the MIC10 subcomplex, analogous to MIC26. Alternatively, STREMI may function as an assembly or maintenance factor that transiently engages MICOS intermediates and promotes formation or stability of higher-order assemblies. This latter model is consistent with the broader principle that multiple mitochondrial complex assembly factors act through shared local structural features or motifs with their cognate complex components(Cogliati *et al*, 2016; Laube *et al*, 2024; Parey *et al*, 2019).

Because structures of intact mammalian MICOS assemblies remain unsolved, future structural analyses will be important to define how STREMI engages MICOS and how it influences MICOS assembly-state distributions. We have updated the manuscript to incorporate these data and to replace the previously vague term “fine-tune” with more precise language, see Fig.4 and line 223. Thank you for this helpful suggestion.

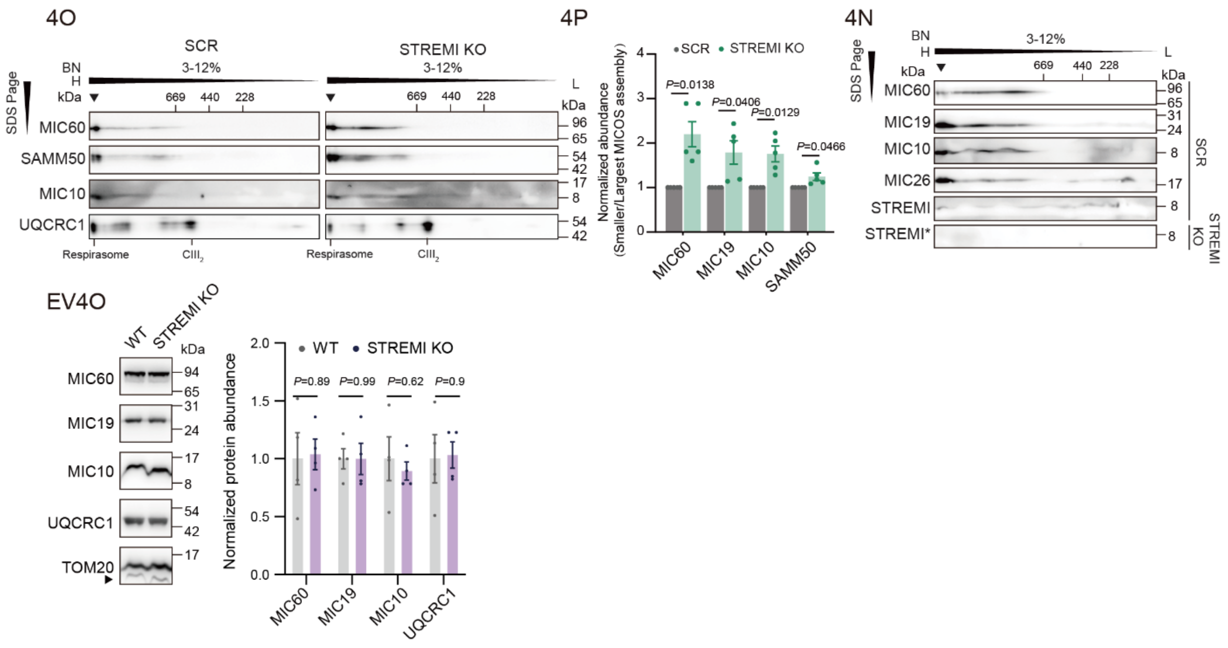

STREMI is required for optimal MICOS assembly

(4O) Two-dimensional blue native electrophoresis of mitochondrial lysates from SCR control and STREMI KO 293T cells. The position of the largest MICOS assembly is marked by a triangle.

(4P) Normalized relative abundance of smaller MICOS assemblies compared with the largest MICOS assembly, quantified from SCR control and STREMI KO 293T cells. The SCR control ratio was set to 1. Data are presented as mean ± SEM (n = 5). P values were calculated using two-tailed paired t tests.

(4N) Two-dimensional blue native electrophoresis of mitochondrial lysates from SCR control and STREMI KO 293T cells. Asterisks (*) denote the STREMI KO sample. The position of the largest MICOS assembly is marked by a triangle.

(EV4O) SDS-PAGE of purified WT and SUMISTREMI KO BAT mitochondria followed by immunoblotting for the core subunits of MICOS. Protein abundance was normalized to the level of TOM20. Data are presented as mean ± SEM (n = 4). P values are from two-tailed unpaired t tests.

7. There is a rationale gap between Figure 4J-4L and the conclusion that SUMI can oligomerize together with MIC10 through their shared topology and motif. The authors need to explain why "through their shared topology and motif".

We thank you for pointing this out, and we have revised the manuscript to avoid confusion. The shared topology refers to the fact that both STREMI and MIC10 contain two transmembrane helices and adopt a N^{IMS}-C^{IMS} topology (Fig. 4B-F). The shared motif refers to the GxxxG motif, which is known to mediate transmembrane-domain oligomerization (Fig. 4G, H, L)(Barbot *et al*, 2015; Bohnert *et al.*, 2015; Russ & Engelman, 2000). Additionally, we examined which MICOS subunits can directly associate with STREMI under overexpression conditions and found that MIC10 showed the strongest interaction (Fig. 4M). Together, these results indicate that STREMI can oligomerize with MIC10 and shares key topological and motif features with MIC10, see line 247-266.

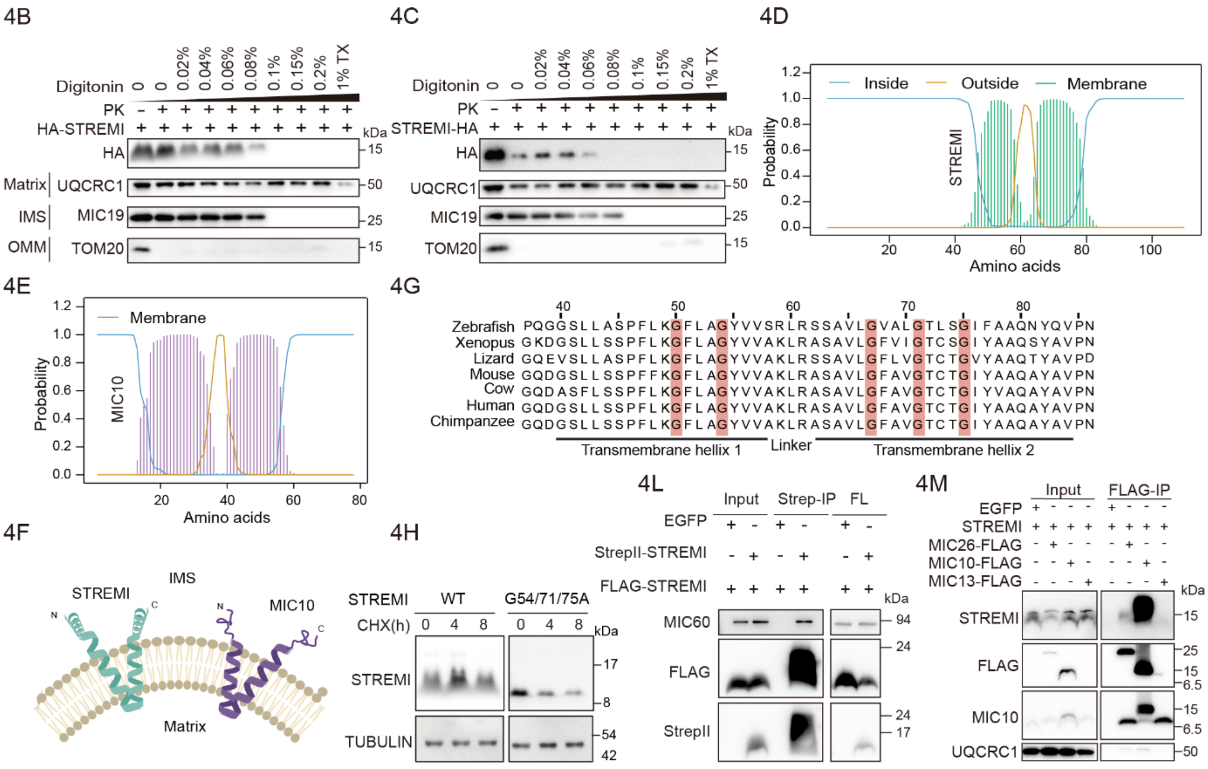

STREMI shares key topological and motif features with MIC10

(4B, C) Topology of STREMI analyzed by proteinase K digestion with progressive membrane solubilization using digitonin. STREMI carried an N-terminal HA tag (B) or a C-terminal HA tag (C). TOM20, MIC19, and UQCRC1 were used as markers for the outer membrane (cytosol exposed), intermembrane space (IMS), and matrix, respectively.
(4D, E) Transmembrane domains of STREMI (D) and MIC10 (E) predicted by DeepTMHMM (1.0.42).
(4F) Topology of STREMI (green) and MIC10 (purple) in the mitochondrial inner membrane (IM).
(4G) Multiple sequence alignment of transmembrane segments of STREMI homologs, with evolutionarily conserved GxxxG motifs highlighted in red.
(4H) Protein stability of SUMI (G54/71/75A) analyzed by cycloheximide chase.
(4L) FLAG- and StrepII-STREMI co-expression in HEK293T cells with Strep-Tactin pulldown, showing FLAG-STREMI and endogenous MIC60 in different fractions (FT, flow through).
(4M) STREMI interaction screening with FLAG-MIC26/10/13 via FLAG immunoprecipitation.

8. Do the authors think that SLC35A4-MP and SLC35A4 main protein get translated from the same individual mRNA, or does a given mRNA only translate one of these two potential proteins?

Our data suggest that leaky scanning underlies the translational regulation of this bicistronic transcript. First, disruption of the STREMI start codon increases the translational accessibility of the downstream ORF (Fig. 5D). Second, activation of SLC35A4 translation is accompanied by a reciprocal reduction in the translation signal of STREMI (Fig. EV5A). Third, no IRES element is predicted within this transcript.

Together, we favor a cap-dependent scanning model with context-dependent leaky scanning. During a given ribosome-scanning event, only one of the two proteins—either STREMI or SLC35A4—is translated. Under basal conditions, STREMI is preferentially translated, whereas activation of the integrated stress response shifts ribosome usage toward the SLC35A4 ORF. Across multiple scanning events on the same mRNA

molecule, both STREMI and SLC35A4 can be translated, but in a mutually exclusive manner per individual scanning event.

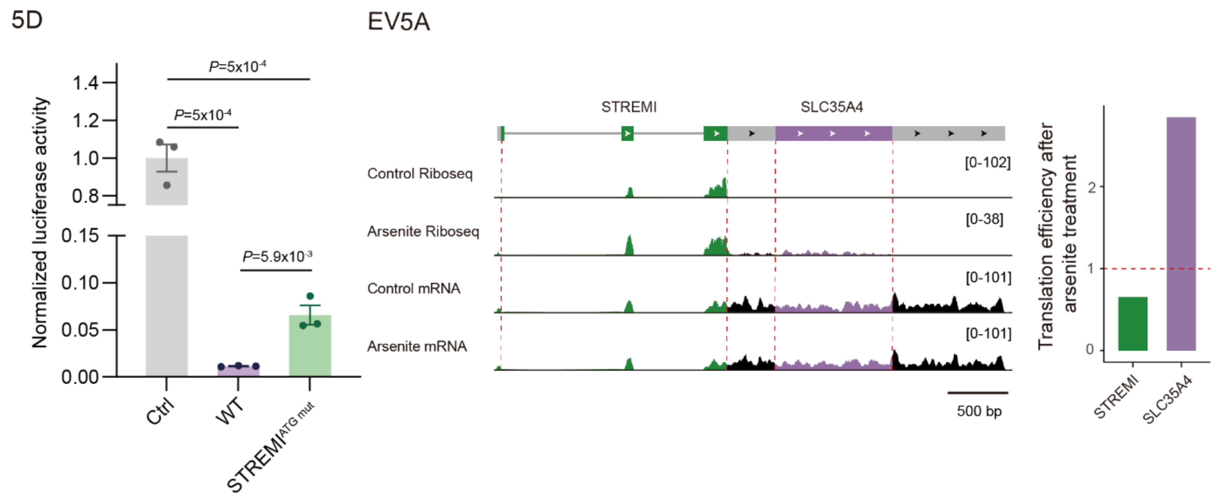

Leaky scanning-dependent translational switching between STREMI and SLC35A4
(5D) Dual-luciferase assay of Ctrl, WT and STREMIATG mut variants. Data are presented as mean ± SEM (n = 3). P values are from two-tailed unpaired t tests followed by Holm-Bonferroni correction for multiple comparisons. (EV5A) Ribosome footprint density on SLC35A4 transcripts in control and arsenite-treated conditions. Data from(Andreev *et al*, 2015).

Minor concerns:
9. In Figure 2F, immunoblotting of MIC10 needs repeat. The band is not clear.

We repeated the STREMI immunoprecipitation and obtained a more clearly resolved MIC10 signal (Fig. 2F).

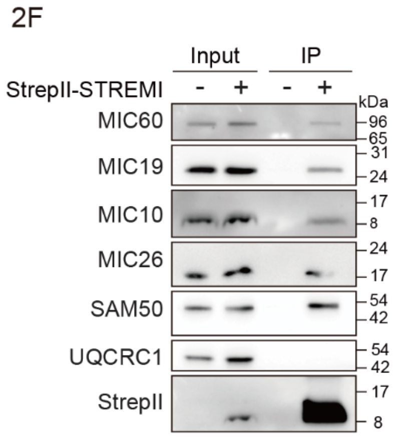

(2F) Immunoblot analysis of interactions between MICOS subunits and StrepII-STREMI following immunoprecipitation via the StrepII tag.

10. Please specify the abbreviations when it is first shown in the text, such as OMM, MICOS, TEM etc.

Thank you for the reminder. We have revised the manuscript accordingly.

11. The authors should note that there are important uORF proteins that they have not referenced here, including ones with importance in the mitochondria, eg. PMID 33406399, 34593629

We thank you for these valuable suggestions. We have incorporated the recommended references, please see line 76-77.

Referee #2:

In this manuscript, Guo R., Guo Y., and colleagues investigate a bicistronic transcript of SLC35A4 that encodes both a uORF-derived mitochondrial microprotein, termed SUMI (previously SLC35A4-MP), and the canonical Golgi-resident transporter SLC35A4. Using long-read sequencing, proteogenomics, and functional assays, the authors identify 14 bicistronic human transcripts and focus on SLC35A4 as a representative model. They show that SUMI localizes to the inner mitochondrial membrane, interacts with the MICOS subunit MIC10, and contributes to mitochondrial cristae morphogenesis. The study also proposes that the uORF functions as a stress-responsive translational regulator of the downstream SLC35A4 ORF and introduces an evolutionary "gene symbiosis" model for the origin of bicistronic genes through retroposition.
Overall, the manuscript is well written, logically structured, and technically rigorous. It provides a conceptual framework for the evolution of dual-function transcripts. However, several of the biological findings, particularly those concerning mitochondrial localization, cristae defects, and metabolic consequences, have been previously reported by Rocha et al. (J. Mol. Biol. 2024; Sci. Adv. 2025) and Ajala et al. (Protein Sci. 2025). These earlier studies already demonstrated that SLC35A4-MP is a conserved mitochondrial microprotein whose loss perturbs respiration, cristae organization, and fatty-acid oxidation in brown adipose tissue. In the present manuscript, the discussion of these prior works is vague and, in the case of Rocha et al. (Sci. Adv. 2025), the reference is entirely absent despite multiple overlapping claims. I understand that this omission is likely due to the very recent publication of that paper (August 2025). To allow a fair assessment of novelty, the authors must explicitly acknowledge all the earlier literature and delineate which aspects are genuinely new. In my view, the mechanistic dissection of SUMI-MIC10 interaction, the determination of membrane topology, and the proposed evolutionary model of bicistronic gene origin appear to be some main novel contributions. I also have concerns regarding the nomenclature and the evolutionary interpretation, which are detailed below.

We thank you for your professional assessment of our study. We would first like to note that, following discussions with HGNC, we now refer to this protein-coding uORF as **STREMI**, and we use this designation throughout our point-by-point response. Please see our response to Point 2 of Reviewer 1 for additional details.

As you noted, our study makes two key advances. First, we provide mechanistic context for STREMI function by defining its dual-transmembrane topology and its association with MIC10, which together support a coherent model for how STREMI contributes to mitochondrial cristae regulation. Second, we comprehensively examine the expression and functional relationship between the STREMI-SLC35A4 ORF pair and clarify the evolutionary origin of their bicistronic architecture, helping to explain why the upstream and canonical ORFs can be functionally distinct despite being co-transcribed. Importantly, neither of these two aspects has been addressed in the published literature to date.

Major comments

- The primary issue is the insufficient recognition of previously published results. The roles of SLC35A4-MP in mitochondrial respiration, oxidative stress responses, and cristae disruption have already been established, including the brown adipose tissue phenotypes observed under cold exposure and high-fat diet in Rocha et al. (Sci. Adv. 2025). The authors should clearly what biological aspects are not novel and fairly specify their new contributions.

We thank you for this suggestion. We are committed to providing a complete literature context and clearly defining the novelty of our work. We have also explicitly acknowledged the prior literature on this uORF in the Introduction, Results, and Discussion sections (see line 75-90; 125-140; 426-436; 443-449).

Below is the paragraph we added to the Introduction:

"To date, only a few human uORFs have been experimentally validated as encoding biologically active polypeptides (Hofman *et al.*, 2024; Huang *et al.*, 2021; Jayaram *et al.*, 2021; Rathore *et al.*, 2018; Rensvold *et al.*, 2022). Among these, the 5'UTR of SLC35A4 was first reported in 2013 to encode a 103-amino acid microprotein and was designated PE1 by the Human Proteome Project in 2018(Omenn *et al.*, 2018; Vanderperre *et al.*, 2013). Our group subsequently identified this polypeptide as a mitochondrially localized microprotein, a finding later corroborated by multiple independent studies (Ajala *et al.*, 2025; Liu *et al.*, 2024; Yang *et al.*, 2024; Zhang *et al.*, 2020). More recent work has implicated this microprotein in maintaining mitochondrial architecture, oxidative stress responses, and respiratory function in both cultured cells and mouse models (Ajala *et al.*, 2025; Rocha *et al.*, 2024; Rocha *et al.*, 2025). In mice, its deficiency alters mitochondrial lipid composition under a high-fat diet and impairs fatty acid oxidation (Rocha *et al.*, 2025). Despite this growing interest, the precise molecular function of this uORF-encoded microprotein remains unresolved. Moreover, the functional and regulatory interplay between the upstream and downstream ORFs—and their evolutionary origin—has not been systematically addressed, leaving the broader biological significance of this protein-coding uORF unclear. "

Regarding molecular mechanism, prior studies reported conflicting submitochondrial localizations, placing the microprotein either at the outer membrane or the inner membrane(Ajala *et al.*, 2025; Rocha *et al.*, 2024; Yang *et al.*, 2024). Our study resolves this discrepancy through detailed topology analyses, demonstrating that STREMI is a dual-pass transmembrane microprotein localized to the mitochondrial inner membrane. In addition, we performed an unbiased interactome analysis and identified MICOS as its primary interaction partner, with MIC10 showing the strongest association, consistent with their shared topology and conserved motifs. Together, these results provide a mechanistic framework linking STREMI to MICOS biology and clarify the molecular basis of STREMI's mitochondrial function.

- Regarding the evolutionary analysis, the manuscript reports the presence of two separate genes in sea lamprey and two consecutive genes in thorny skate, which is indeed an intriguing observation, as previous studies traced conservation only up to zebrafish. However, it is important to verify whether RNA-seq data support the independent transcription of these loci in thorny skate, or whether this might be an annotation artifact and the gene is in fact bicistronic in this species as well. Clarification is also needed for sea lamprey: although the proposed mechanism is interesting, the evidence is currently limited to one species, and potential issues in genome quality or annotation could influence the result. Is the same locus configuration retained in the 2025 sea lamprey genome assembly? Does the annotated SLC35A4 homolog in lamprey actually correspond to SLC35A3? According to the authors' hypothesis, could retroposition have been initially driven by the expression of SLC35A3? It would also be informative to examine whether SLC35A4-MP/SUMI is positionally conserved (e.g. using synteny) across lamprey, thorny skate and other vertebrate genomes, and whether orthologous sequences exist in other lampreys such as Lethenteron camtschaticum and in hagfish. Even if the corresponding protein is unannotated in protein databases, one might expect its transcription/sequence to be detectable in the genomes of these species, likely supporting a non-bicistronic configuration if the retroposition event happened after the split of lamprey/hagfish and the rest of vertebrates. Since the proposed retroposition mechanism is a central novelty of the manuscript, addressing these points would greatly strengthen the evidence for the evolutionary model.

We thank you for these insightful suggestions regarding a more detailed evolutionary analysis. Below, we address each point in turn.

Independent transcription vs. bicistronic transcript in thorny skate

To address this point, we searched the NCBI Sequence Read Archive for transcriptome datasets from thorny skate (*Amblyraja radiata*) and identified a single RNA-seq dataset (SRX5956897). Unfortunately, we did

not recover informative reads mapping across the STREMI-SLC35A4 locus in this dataset. We therefore expanded our analysis to other cartilaginous fishes and identified a long-read RNA-seq dataset from wide sawfish (*Pristis pectinata*), in which long reads span the STREMI-SLC35A4 region (Fig. EV8E). Notably, multiple reads cover both the STREMI and SLC35A4 ORFs, even though the current genome annotation represents them as two independent transcripts—similar to the annotated configuration in thorny skate (Fig. EV8E and 7C). In addition, synteny-based analyses in cartilaginous fishes captured a tight genomic association between the ORFs encoding STREMI and SLC35A4 orthologs (Fig. EV8D). These observations suggest that when STREMI lies immediately upstream of an intronless SLC35A4, the bicistronic transcript is likely the predominant form. Accordingly, we replaced thorny skate with sawfish in the revised manuscript.

Locus configuration in the 2025 sea lamprey genome assembly

In the updated 2025 sea lamprey (*Petromyzon marinus*) genome assembly, the locus configuration remains unchanged (Fig. 7C). We performed comprehensive homology searches (tBLASTn) and protein-family annotation (HMMER) to identify SLC35A family members in sea lamprey (Methods, line 859-866). We recovered four SLC35A family members, and phylogenetic analysis assigned them to SLC35A1/2/3/5, consistent with current gene annotations (Fig. EV8A, B and Dataset EV5) (Table R2). Importantly, we could not identify a convincing SLC35A4 ortholog in sea lamprey, and none of the other SLC35A family members map to the vicinity of the STREMI locus (Fig. EV8A, B and Dataset EV5) (Table R2).

Conservation of STREMI in other lamprey and hagfish species

We next conducted a comprehensive homology search across lamprey and hagfish lineages. Using tBLASTn followed by manual curation, we recovered full-length or near full-length STREMI homolog coding sequences from two lamprey species and three hagfish species (Fig. EV8C and Dataset EV5). The GxxxG motif(s) within the transmembrane segment(s), as well as the positively charged residues separating the two transmembrane domains, are highly conserved across these species (Fig. EV8C). These data support conservation of STREMI in both lampreys and hagfish.

Like our observations in sea lamprey, we also did not identify a confident SLC35A4 ortholog in brown hagfish (Eptatretus atami), and the identified SLC35A1/2/3/5 orthologs are not located near the STREMI locus (Table R2) (Marlétaz et al., 2024).

Which SLC35A family member contributed to the intronless SLC35A4 via retroposition?

To investigate the evolutionary origin of SLC35A4, we examined basal chordate genomes and found that multiple lancelet species encodeSLC35A4 homologs as multi-exon transcripts (Fig. 7C and EV8A, B). In contrast, our searches did not identify a STREMI homolog in lancelet species. Together, these observations are consistent with SLC35A4 being present earlier in chordate evolution, with STREMI arising later in vertebrate evolution.

As described above, STREMI is conserved in lamprey and hagfish, whereas an SLC35A4 ortholog cannot be confidently identified in these cyclostomes. In jawed vertebrates, by contrast, STREMI and SLC35A4 (which is consistently intronless) are found in close proximity and frequently appear as a bicistronic configuration (Fig. EV8D, E). We attempted to extend this synteny-based analysis to lamprey genomes, but did not obtain a robust result, potentially due to extensive chromosomal rearrangements and assembly/annotation complexity in this lineage(Smith *et al*, 2013; Smith *et al*, 2018).

Taken together, these data support the following working model. STREMI likely emerged at or before the last common ancestor of extant vertebrates, given its conservation in both jawless and jawed vertebrates, whereas SLC35A4 has a deeper origin within chordates. Subsequently, SLC35A4 appears to have been lost in extant jawless vertebrates. In the jawed vertebrates, a retroposition event likely generated the intronless SLC35A4 adjacent to STREMI, followed by loss of the ancestral multi-exon SLC35A4. This interpretation

is consistent with the model that the retroposition event giving rise to the present STREMI-SLC35A4 configuration occurred after the divergence of the lamprey/hagfish lineage from the rest of vertebrates.

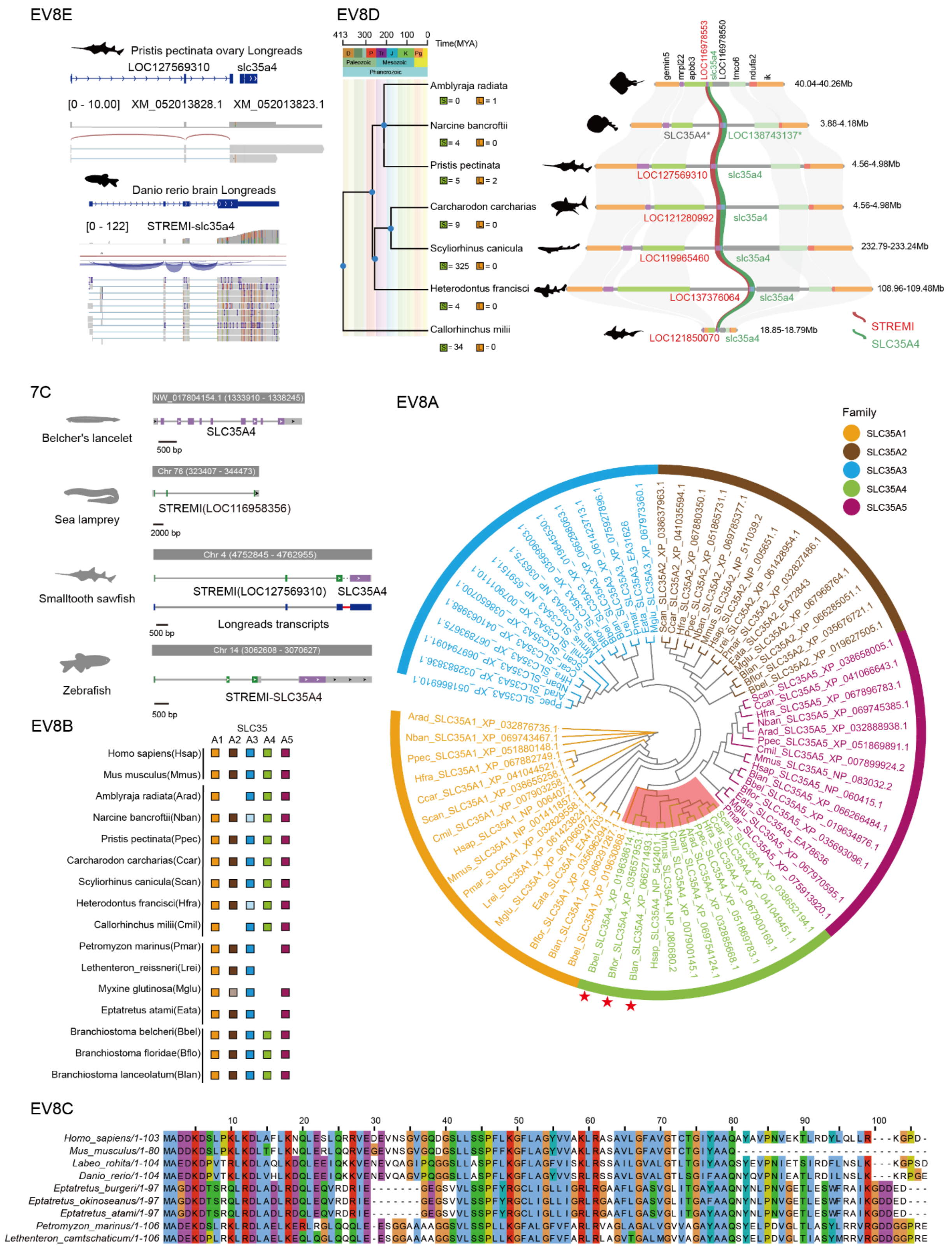

Evolutionary analysis of STREMI and SLC35A4 homologs in chordates
(EV8E) Long-read Iso-Seq evidence supporting bicistronic transcription of the STREMI-SLC35A4 locus in *Pristis pectinata* and *Danio rerio*. Gene models and mapped reads illustrate the shared transcript structure.
(7C) Representative gene models of STREMI and SLC35A4 across selected species.
(EV8D) Conserved synteny of the STREMI-SLC35A4 locus in representative cartilaginous fishes, integrated with species phylogeny and available RNA-seq datasets (S, short-read; L, long-read). Asterisks indicate likely annotation errors in NCBI genome records, in which the names of slc35a4 and the uORF-encoded STREMI homolog (LOC138743137) appear to be swapped.
(EV8A) Protein phylogenetic tree of SLC35 family members across lancelet species and vertebrates. Branches are color-coded by gene family, with the SLC35A4 clade highlighted; red stars indicate SLC35A4 in lancelet species.
(EV8B) Presence-absence matrix showing the distribution of SLC35 homologs across major chordate and vertebrate lineages.
(EV8C) Multiple sequence alignment of representative STREMI homologs highlighting conserved residues across species.

Table R2. STREMI homologs and SLC35A family members in *Petromyzon marinus* and *Eptatretus atami*

Organism Name	Gene name	Genome location	ID
Petromyzon marinus	SLC35A1	chr51:10792031-10817102	XP_032829558.1
Petromyzon marinus	SLC35A2	chr46:14463664-14479395	XP_032827486.1
Petromyzon marinus	SLC35A3	chr35:341163-366295	XP_075927896.1
Petromyzon marinus	SLC35A5	chr84:3008322-3039724	XP_075913920.1
Petromyzon marinus	LOC116958356 (STREMI)	chr76:421478-464030	XM_032980917
Eptatretus atami	SLC35A1	chr2:50246782-50291455	EA41703(Marlétaz *et al*, 2024)
Eptatretus atami	SLC35A2	chr3:127718304-127787023	EA72843
Eptatretus atami	SLC35A3	chr7:95315518-95387362	EA31626
Eptatretus atami	SLC35A5	chr3:97482732-97502571	EA78636
Eptatretus atami	EA32229 (STREMI)	chr7:82777881-82791255	EA32229

- Finally, nomenclature consistency is crucial. Previous literature consistently refers to the peptide as SLC35A4-MP. Introducing a new name, "SUMI," without HGNC approval could generate unnecessary confusion in the field. I recommend retaining SLC35A4-MP as the primary name throughout the text, mentioning "SUMI" only as an alternative descriptor. However, if the authors consider that the new name "SUMI" better reflects a specific mechanistic or functional property not captured by the previous designation, they should explicitly justify its introduction and clarify its rationale in the manuscript.

We thank you for this suggestion. This uORF has not yet been assigned an approved HGNC symbol and is still listed under placeholder annotations in CCDS and Ensembl. Our primary goal is to provide this uORF with a clear, functionally grounded designation.

In the literature, the translation product has been referred to as AltSLC35A4 or SLC35A4-MP (Ajala *et al.*, 2025; Rocha *et al.*, 2024; Rocha *et al.*, 2025). However, these names do not adequately convey the

molecular and functional properties of the microprotein and may be misconstrued as indicating an SLC35A4 isoform or a protein that functions directly together with SLC35A4. As detailed in our response to Point 2 of Reviewer 1, we discussed nomenclature with HGNC and adopted the name STREMI ("SLC35A4 stress response regulating MICOS interactor"), which captures the dual functions of this uORF.

Minor comments

- The evolutionary conservation of the SUMI peptide across is visualized in Figure 1 but restricted to a subset of species. Including a full multiple-sequence alignment including all species with SUMI in Fig. 7D and highlighting conserved motifs, particularly the GxxxG oligomerization motifs, and reporting percentage sequence identity across representative species would provide valuable context, especially for comparison with sea lamprey.

1G

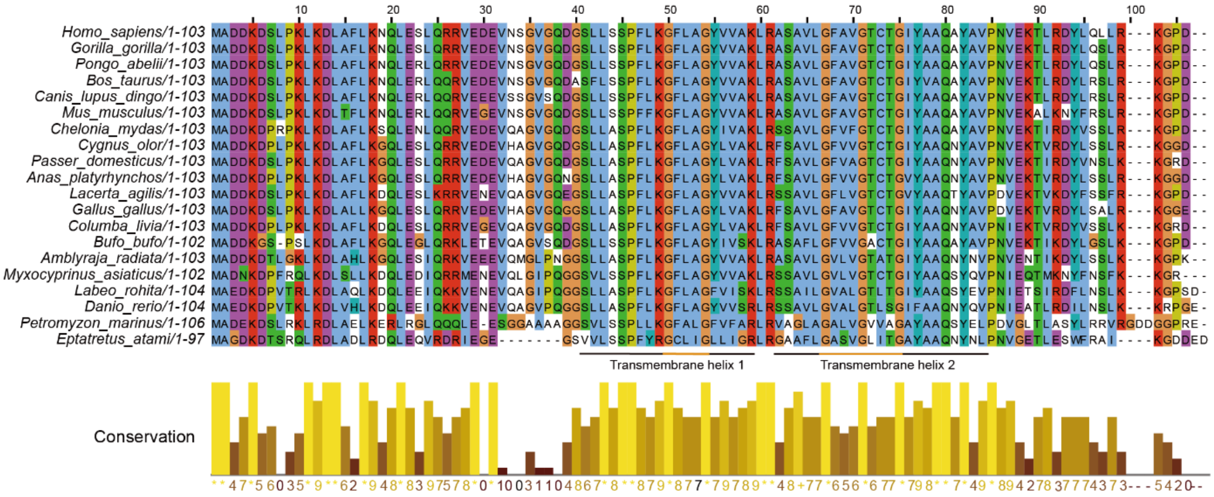

We have repeated the multiple sequence alignment, now including protein sequences from sea lamprey (*Petromyzon marinus*) and brown hagfish (*Eptatretus atami*) (Fig. 1G). This analysis clearly shows that the GxxxG motifs are conserved within both transmembrane domains across all examined species.

- Statistical analysis details should be clarified. Many figures (for example, Figure 5) display multiple p-values without apparent indication of multiple-testing correction. The authors should confirm that all p-values are properly adjusted and specify the correction method used.

We thank you for raising this issue. We reviewed our statistical analyses and applied multiple-testing correction using the Holm-Bonferroni method in Figure 5 panels where appropriate. Our conclusions remain unchanged after correction. The statistical methods are specified in the corresponding figure legends.

- Line 466, the definition of a "good" Ribo-seq signal should be quantified. The thresholds used to support translation should be explicitly stated in the Methods section.

We performed additional Ribo-seq analyses to establish more standardized filtering criteria. Briefly, we analyzed nine human tissue Ribo-seq datasets using PRICE and RiboCode. ORFs supported by a P value ≤ 1×10^{-5} in either PRICE or RiboCode were considered to have strong evidence of translation. These results have been included in Dataset EV1; see Methods (line 507-518) for details.

- Line 753: Was a similar E-value cutoff used as in the previous section? Please clarify what is meant by "adjacent." Was there a defined maximum distance threshold?

For the identification of bicistronic ORF pairs across species, we performed tblastn searches using the human bicistronic ORFs as queries and applied the same E-value cutoff as in the previous section (1e-5).

Here, "adjacent" refers to ORF pairs that are neighboring in the genome and not separated by any other annotated gene. We did not impose a fixed maximum genomic distance threshold because intron lengths can vary substantially across species. Instead, we extracted the genomic coordinates for each candidate ORF pair and first determined whether the two ORFs reside on the same chromosome or contig. For pairs located on the same chromosome/contig, we calculated the physical distance between them and manually inspected each case to confirm true adjacency (i.e., direct genomic neighborhood without intervening annotated genes).

- Figure 7D: This information is valuable but should be complemented by a supplementary table listing all identified homologs in other species for the seven gene pairs, including their names/IDs, E-values, and percentage identities. Do the authors know why SUMI (LOC116958356) is annotated as DNPH1 in Ensembl for sea lamprey?

The table detailing Fig. 7D has now been provided as Dataset EV6. We also do not understand why LOC116958356 is annotated as DNPH1. We confirm that there is no detectable sequence homology between STREMI and DNPH1, suggesting that this may reflect an error in the Ensembl Rapid annotation.

Referee #3:

The authors address the evolution of bicistronic ORFs and focus specifically on the role of a microprotein that is encoded upstream of the SLC35A4. This novel mitochondrial protein, termed SUMI, and its organization at the genomic level was studied already by several other groups (Ajala et al., 2025; Rocha et al., 2024; Rocha et al. 2025) and also by the same group (Zhang et al., 2020) in recent studies earlier - as the authors correctly point out. Thus, the novelty of this study is not in identifying a novel microprotein which affects mitochondrial respiration. It is also not in identifying an increasing number of bicistronic ORF having mitochondrial functions. The novelty is that this study shows convincingly that SUMI is interacting with the MICOS complex and that it assists in shaping cristae. This study helps to resolve several discrepancy and uncertainties regarding the localisation of SUMI and identify the SLC35A4-MP (SUMI) as a bona fide subunit of the MICOS complex. Moreover, the authors even generate a SUMI knockout mouse model. The authors provide also interesting information on the possible evolution of this bicistronic ORF and its stress-dependent regulation.

Overall, I think it is an important study. Yet, I think that many aspects of the already published studies are not really novel and take too much space in the manuscript. The mechanistic role of this novel MICOS-interaction protein is partly addressed but not in much detail. The phenotype of the mouse is not grossly described and need further emphasis as well as the role of SUMI on cristae junction formation and on the MICOS complex itself.

We thank you for highlighting the significance of our study and for your constructive suggestions regarding the mechanistic role of this uORF-encoded microprotein. We also note that, following discussions with HGNC, we now refer to this protein-coding uORF as STREMI, and we use this designation throughout our point-by-point response. Please see our response to Point 2 of Reviewer 1 for additional details.

Major points.

1. Is SUMI localized at cristae junctions. Fig. S4A and the partial overlap with MIC60 is not convincing. Can the authors perform immune-gold labelling?

We thank you for this insightful suggestion. We performed immunogold labeling with silver enhancement in scramble control and STREMI-knockout (KO) HeLa cells to determine the subcellular localization of STREMI. In cells expressing a scrambled gRNA, gold particles were strongly enriched over mitochondria (Fig. EV2F). By contrast, mitochondrial colocalization was rarely observed in the STREMI KO line, supporting the specificity of the antibody signal (Fig. EV2F). Notably, gold particles were preferentially distributed along the mitochondrial envelope, with substantially fewer particles detected within the mitochondrial interior (Fig. EV2G), a pattern previously reported for multiple MICOS subunits(Harner *et al*, 2011) . In mitochondria where cristae architecture was clearly resolved, 46.5% of gold particles were positioned in close proximity (<50 nm) to cristae junctions, , comparable to the distribution reported for MICOS subunits (Fig. 2H, I) (Guarani *et al.*, 2015; Harner *et al.*, 2011). Together with our biochemical evidence supporting inner mitochondrial membrane (IMM) localization, these data indicate that STREMI is not randomly distributed along the IMM, but instead exhibits preferential enrichment near cristae junctions.

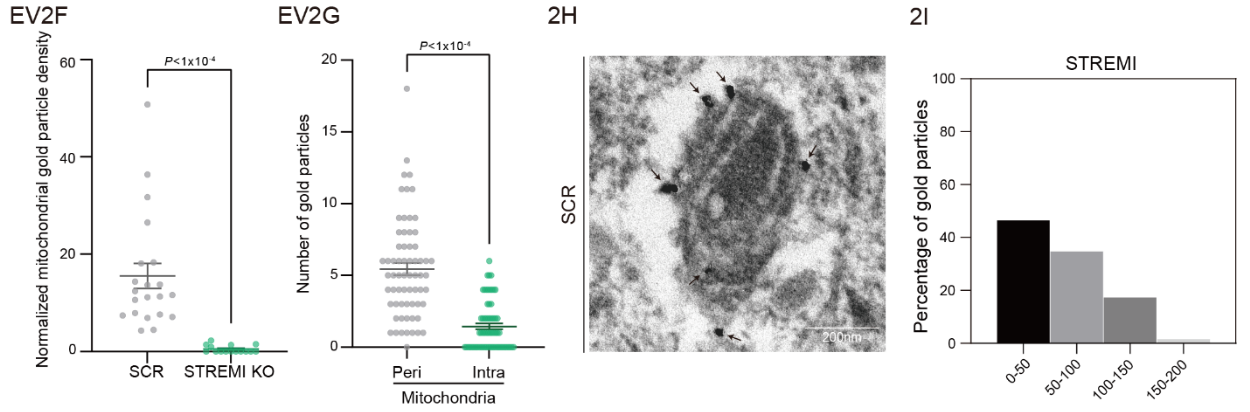

STREMI is preferentially localized to the cristae junction
(EV2F) Mitochondrial gold particle density normalized to non-mitochondrial regions in SCR and STREMI KO HeLa cells. P values are from unpaired two-tailed t tests.
(EV2G) Quantification of gold particles in intra-mitochondrial and peri-mitochondrial regions. The peri-mitochondrial region was defined as the 50-nm zone surrounding the outer mitochondrial membrane. Data are presented as mean ± SEM (n = 65 mitochondria). P values are from paired two-tailed t tests.
(2H) Immunogold detection of endogenous STREMI in HeLa cells. Scale bar, 200 nm. Gold particles are indicated by arrows.
(2I) The histograms show the fraction of gold particles within the indicated distance to the crista junction in nanometers in HeLa cells.

2. Does SUMI affect glycolysis and fatty acid oxidation in cells (or tissues)?

To determine whether loss of STREMI causes metabolic defects in mice, we performed untargeted metabolomics on WT and STREMI KO animals under basal conditions and after cold challenge. We focused on brown adipose tissue (BAT), the tissue in which STREMI KO produces the most pronounced mitochondrial ultrastructural abnormalities. Principal component analysis (PCA) showed no clear separation between WT and KO samples, indicating that STREMI deficiency does not induce a global metabolic reprogramming (Fig. EV3E). Consistent with this, we did not detect reproducible changes in the abundance of canonical glycolytic or tricarboxylic acid (TCA) cycle intermediates (Fig. EV3F, G).

In contrast, we observed a specific and significant reduction in multiple long-chain hydroxy-acylcarnitines following cold stress, consistent with decreased fatty-acid oxidation (FAO) flux in BAT (Fig. EV3H, I)(Choi *et al*, 2024; Simcox *et al*, 2017). Notably, defects in FAO have been reported in several mitochondrial membrane-shaping perturbations, including disruption of MICOS component MIC26 (Guo *et al*, 2023; Li *et al*, 2021; Ngo *et al*, 2023). We therefore favor the interpretation that the FAO signature in STREMI KO BAT likely reflects a secondary consequence of altered mitochondrial architecture rather than a primary metabolic function of STREMI.

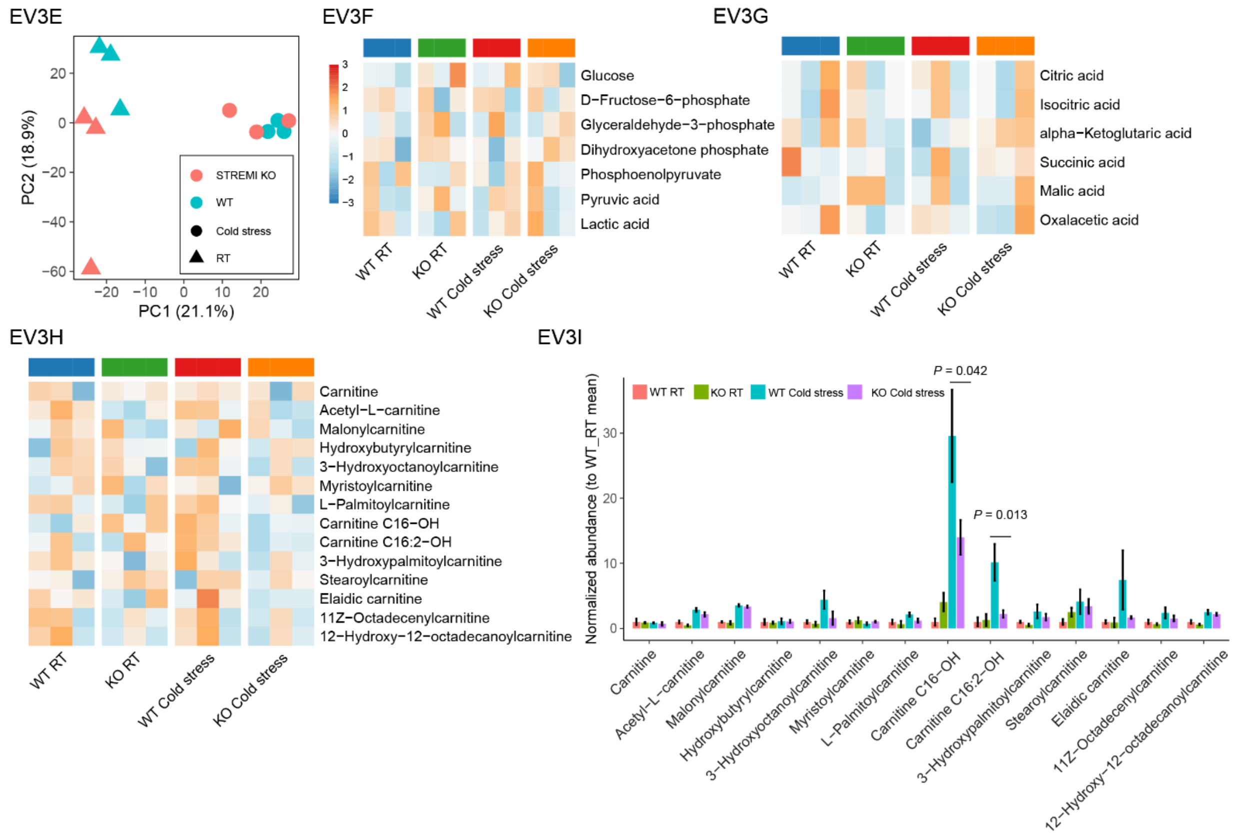

Metabolomic analysis of brown adipose tissue in STREMI-deficient mice

(EV3E) Principal component analysis (PCA) of untargeted metabolomic profiles from brown adipose tissue (BAT) of wild-type (WT) and STREMI-knockout (KO) mice housed at room temperature (RT) or subjected to cold stress (4 °C for 5 days).

(EV3F-H) Heatmaps showing the relative abundance of metabolites from glycolysis (panel F), the tricarboxylic acid (TCA) cycle (panel G), and acylcarnitines (panel H). Values were $\log_2$-transformed and then z-scored across metabolites within each condition (RT and cold stress processed separately).

(EV3I) Acylcarnitine abundances in BAT normalized to WT at RT (compound-wise mean). Bars indicate mean normalized abundance and error bars represent SEM. P values were calculated using two-way ANOVA followed by Sidak's multiple-comparisons correction for within-compound comparisons.

3. More detailed phenotyping of the mouse model is missing in my opinion. Please elaborate more.

In our breeding experiments, STREMI knockout animals were recovered at the expected Mendelian ratios (Fig. EV3A). We observed only a modest reduction in body weight at 12 weeks (Fig. EV3B) and did not detect additional abnormalities in growth or behavior, indicating that STREMI is dispensable for normal development under standard laboratory conditions. To assess whether STREMI loss causes functional impairment, we challenged the animals with an endurance treadmill protocol and observed a mild reduction in exhaustion distance (Fig. EV3C). Consistent with our observations, Rocha et al. reported that mice lacking this uORF are viable and do not exhibit overt phenotypic abnormalities (Rocha *et al.*, 2025) . In line with their finding that the uORF-encoded peptide functions in brown adipose tissue, we likewise observed that the most pronounced phenotype was at the level of mitochondrial ultrastructure (Fig. 3L) (Rocha *et al.*, 2025).

More broadly, several mouse deletion models of mitochondria-localized microproteins show minimal baseline phenotypes, suggesting that loss of these factors can be well tolerated under standard laboratory housing conditions(Makarewich *et al*, 2018; Makarewich *et al*, 2022; Stein *et al*, 2018; Zhang *et al*, 2023). We have also recently described a compensatory mechanism that buffers up to ~50% loss of respiratory chain complex III via formation of a high-molecular-weight supercomplex(Liang *et al*, 2025). Nevertheless, the STREMI coding sequence is under strong purifying selection across vertebrates, implying that its retention confers an evolutionary advantage. A key next step is therefore to identify physiological stress contexts in which this advantage becomes apparent. We are actively pursuing this direction by subjecting STREMI-deficient animals to challenges—for example, probing neural function during aging—to determine whether loss of STREMI leads to measurable phenotypic deficits. Because these experiments require extended time frames, we plan to report them separately and, in the present study, focus on defining the molecular function of STREMI.

EV3A

Group	Genotype			$X^2(2)$	P value
	WT	HET	STREMI KO		
Male	14	32	11	1.1754	0.5556
Female	9	27	8	2.3182	0.3138
Total	23	59	19	3.1782	0.2041

EV3B EV3C

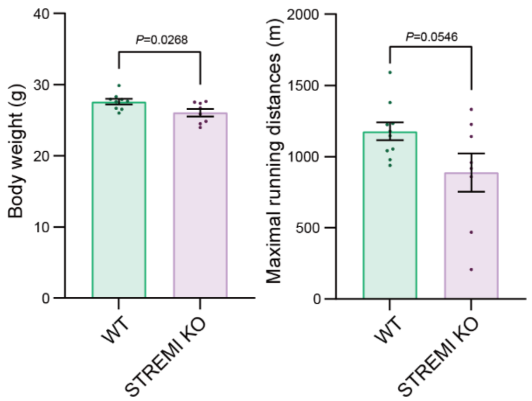

STREMI deletion results in mild physiological phenotypes under basal conditions
(EV3A) Genotype distribution compared with the expected Mendelian 1:2:1 ratio. Observed and expected numbers are indicated. Statistical significance was assessed by χ^2 goodness-of-fit test (df = 2).
(EV3B) Body weight of WT and STREMI KO mice. Data are presented as mean ± SEM (n = 9 mice for WT; n = 8 mice for STREMI KO). P values are from two-tailed unpaired t tests.
(EV3C) Maximum running distance of WT and STREMI KO mice during treadmill testing. Data are presented as mean ± SEM (n = 10 mice for WT; n = 8 mice for STREMI KO). P values are from two-tailed unpaired t tests.

4. What is the rough stoichiometry of SUMI in the MICOS complex and does it change under different metabolic or stress conditions? In this regard it would be interesting to show the unbound fractions of the IP shown in Fig. 2FG.

Because the uORF encoding STREMI also mediates stress-responsive translation of SLC35A4, we asked whether endogenous STREMI levels decrease upon activation of the integrated stress response (ISR) by oligomycin. Endogenous STREMI protein levels remained largely unchanged after 24 h of treatment with 10 nM oligomycin, likely reflecting the high stability of the pre-existing protein pool (Fig. EV5D). These data suggest that uORF-mediated attenuation of STREMI translation can facilitate stress-dependent induction of SLC35A4 without compromising steady-state STREMI abundance.

We also quantified the unbound (flow-through) fractions in Fig. 2F-G following immunoprecipitation (IP) of STREMI and MIC10, respectively. After STREMI IP, we did not detect a substantial depletion of MICOS subunits in the flow-through (Fig. R3A, B). Conversely, following MIC10 IP, we observed a modest reduction in STREMI and several MICOS subunits in the flow-through, although none of these

changes reached statistical significance (Fig. R3C, D). The limited depletion could reflect incomplete IP efficiency and/or a role for STREMI as an assembly factor rather than a stoichiometric component of the mature complex. We have explored this possibility in greater depth in our response to Point 5 (see below).

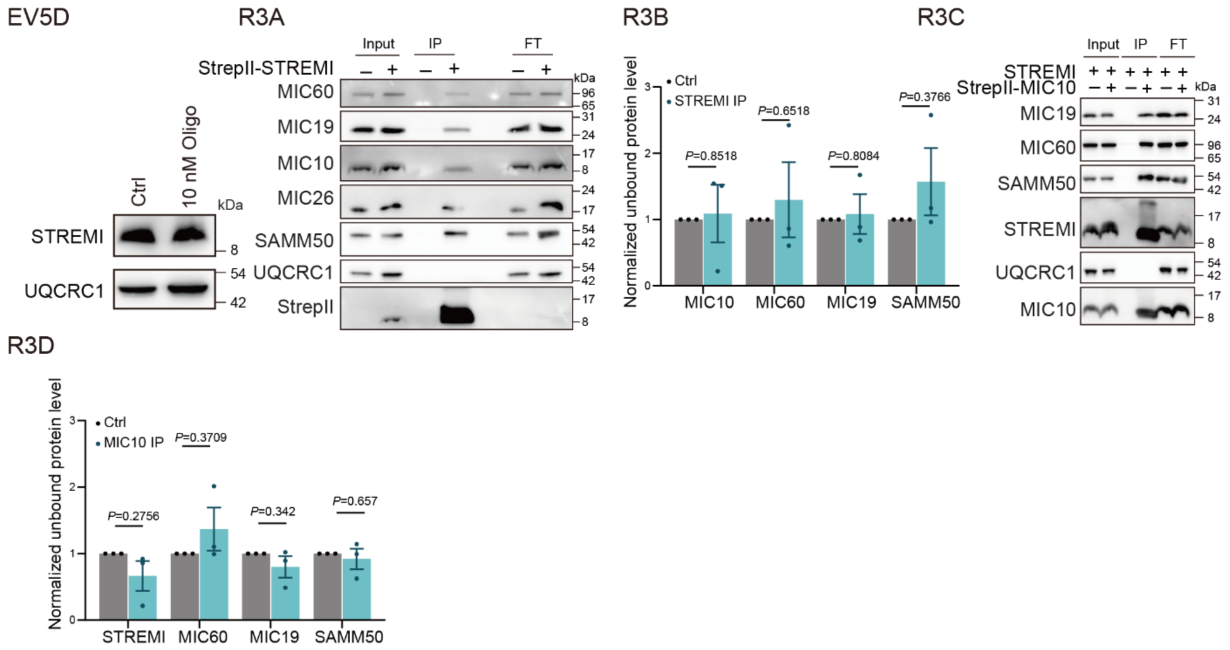

Dynamic incorporation of STREMI into the MICOS complex

(EV5D) Immunoblotting of endogenous STREMI in HEK293T cells after a 24-hour treatment with oligomycin.
(R3A) Immunoblotting of interactions between MICOS subunits and STREMI.
(R3B) Quantification of the unbound fraction of MICOS subunit in the flow-through (FT) following StrepII-tagged STREMI immunoprecipitation from HEK293T mitochondrial lysates compared to control IP. Data are presented as mean ± SEM (n = 3 independent experiments). Statistical significance was determined by two-tailed paired t tests.
(R3C) StrepII-tagged MIC10 was immunoprecipitated from HEK293T cell mitochondria, followed by immunoblotting of MICOS subunits and STREMI.
(R3D) Quantification of the unbound fraction of STREMI and MICOS subunit in the flow-through (FT) following StrepII-tagged MIC10 immunoprecipitation from HEK293T mitochondrial lysates compared to control IP. Data are presented as mean ± SEM (n = 3 independent experiments). Statistical significance was determined by two-tailed paired t tests.

5. What is the native molecular weight of SUMI and does the MICOS complex change in size upon deletion of SUMI?

We thank you for raising this important point. In the revised manuscript, we addressed it using BN-PAGE followed by second-dimension SDS-PAGE. As previously shown by immunoblotting and complexome profiling, MICOS subunits resolve into multiple native assemblies rather than a single, discrete complex(Huynen *et al.*, 2016). The largest species corresponds to the mitochondrial intermembrane space bridging (MIB) complex, which contains MICOS together with outer-membrane components such as SAMM50, metaxins, and DNAJC11 (Fig. 4O) (Huynen *et al.*, 2016). A core MICOS assembly is typically detected around ~700 kDa. In addition, smaller subcomplexes and subassemblies with distinct mobilities are thought to reflect variable oligomerization states, particularly among constituents of the MIC10 subcomplex (Barbot *et al.*, 2015; Bohnert *et al.*, 2015; Rampelt *et al.*, 2018). Consistent with this behavior, the STREMI-specific signal exhibited a broad mobility distribution (Fig. 4N). A similar broad distribution was observed for MIC26, a MIC10 subcomplex component (Fig. 4N).

We next asked whether loss of STREMI alters the native distribution of MICOS assemblies. Overall, the major MICOS-containing species remained detectable in STREMI KO mitochondria, indicating that bulk MICOS assembly is not grossly disrupted (Fig. 4O). However, we observed a reproducible shift toward increased relative abundance of smaller MICOS assemblies compared with the largest MIB-associated species (Fig. 4P). A similar mobility shift was also observed in mouse brown adipose tissue (Fig. EV4T, U). In line with these native-PAGE results, STREMI immunoprecipitation robustly enriched essentially all MIB components, and immunogold labeling showed pronounced enrichment of STREMI at the mitochondrial envelope—an interface where inner and outer membranes are physically coupled (Fig. 2E).

Together, these data suggest two non-mutually exclusive models. First, STREMI may act as an accessory component of the MIC10 subcomplex, analogous to MIC26. Alternatively, STREMI may function as an assembly or maintenance factor that transiently engages MICOS intermediates and promotes formation or stability of higher-order assemblies. This latter model is consistent with the broader principle that multiple mitochondrial complex assembly factors act through shared local structural features or motifs with their cognate complex components(Cogliati *et al.*, 2016; Laube *et al.*, 2024; Parey *et al.*, 2019). Because structures of intact mammalian MICOS assemblies remain unsolved, future structural analyses will be important to define how STREMI engages MICOS and how it influences MICOS assembly-state distributions.

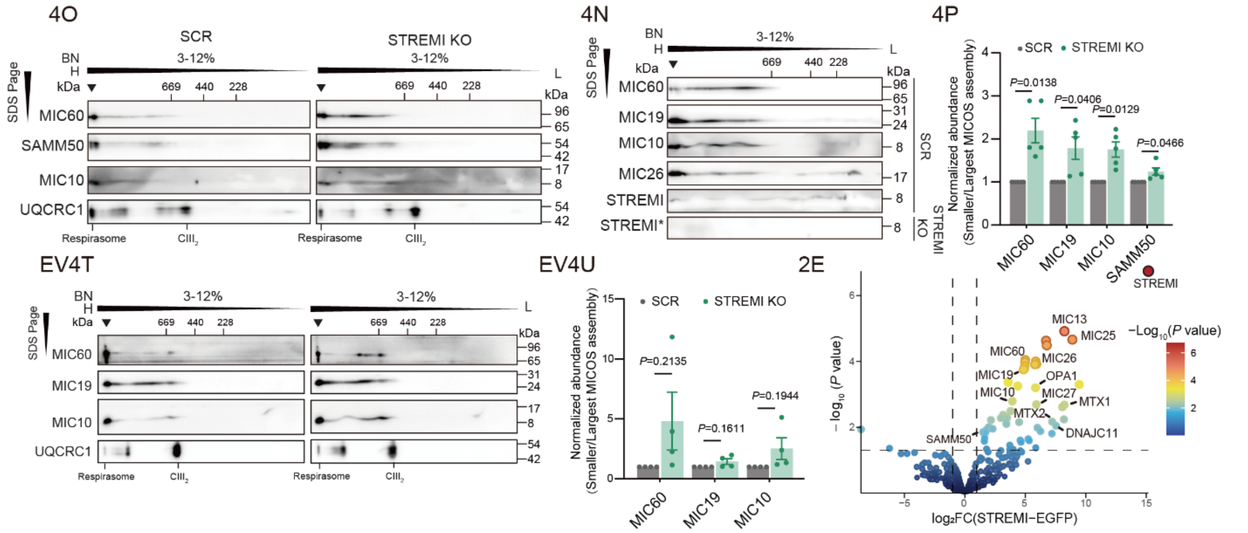

STREMI shapes MICOS assembly

(4O) Two-dimensional blue native electrophoresis of mitochondrial lysates from SCR control and STREMI KO 293T cells. The position of the largest MICOS assembly is marked by a triangle.

(4N) Two-dimensional blue native electrophoresis of mitochondrial lysates from SCR control and STREMI KO 293T cells. Asterisks (*) denote the STREMI KO sample. The position of the largest MICOS assembly is marked by a triangle.

(4P) Normalized relative abundance of smaller MICOS assemblies compared with the largest MICOS assembly, quantified from SCR control and STREMI KO 293T cells. The SCR control ratio was set to 1. Data are presented as mean ± SEM (n = 5). P values were calculated using two-tailed paired t tests.

(EV4T) Two-dimensional blue native electrophoresis of BAT mitochondria from WT and STREMI KO mice. The position of the largest MICOS assembly is marked by a triangle.

(EV4U) Normalized relative abundance of smaller MICOS assemblies compared with the largest MICOS assembly, quantified from BAT mitochondria of WT and STREMI KO mice. The WT control ratio was set to 1. Data are presented as mean ± SEM (n=4). P values are from two-tailed paired t tests.

(2E) Volcano plot of proteins enriched in STREMI immunoprecipitation (IP) versus EGFP control, identified by mass spectrometry (MS). P values are from Benjamini-Hochberg-adjusted two-tailed unpaired t tests.

Minor:

4. Typo in line 203 "...MIC60, supporting its residence in the IMM and interaction with the MICOS complex (S4B Fig).": correct to "...(S4A Fig.)."

We are sorry for this oversight and have corrected it. The panel is now referred to as Fig. EV4A (see line 230).

References

Ajala I, Tiaiba I, Grondin B, Lipuma D, Jmii S, Benlyamani H, Cappadocia L, Vanderperre B (2025) The dual-coding gene SLC35A4 protects against oxidative stress. *Protein Sci* 34: e70197

Andreev DE, O'Connor PB, Fahey C, Kenny EM, Terenin IM, Dmitriev SE, Cormican P, Morris DW, Shatsky IN, Baranov PV (2015) Translation of 5' leaders is pervasive in genes resistant to eIF2 repression. *Elife* 4: e03971

Barbot M, Jans DC, Schulz C, Denkert N, Kroppen B, Hoppert M, Jakobs S, Meinecke M (2015) Mic10 oligomerizes to bend mitochondrial inner membranes at cristae junctions. *Cell Metab* 21: 756-763

Bohnert M, Zerbes RM, Davies KM, Mühleip AW, Rampelt H, Horvath SE, Boenke T, Kram A, Perschil I, Veenhuis M *et al* (2015) Central role of Mic10 in the mitochondrial contact site and cristae organizing system. *Cell Metab* 21: 747-755

Choi J, Smith DM, Scafidi S, Riddle RC, Wolfgang MJ (2024) Carnitine palmitoyltransferase 1 facilitates fatty acid oxidation in a non-cell-autonomous manner. *Cell Rep* 43: 115006

Cogliati S, Calvo E, Loureiro M, Guaras AM, Nieto-Arellano R, Garcia-Poyatos C, Ezkurdia I, Mercader N, Vázquez J, Enriquez JA (2016) Mechanism of super-assembly of respiratory complexes III and IV. *Nature* 539: 579-582

Guarani V, McNeill EM, Paulo JA, Huttlin EL, Fröhlich F, Gygi SP, Van Vactor D, Harper JW (2015) QIL1 is a novel mitochondrial protein required for MICOS complex stability and cristae morphology. *Elife* 4

Guo X, Hu J, He G, Chen J, Yang Y, Qin D, Li C, Huang Z, Hu D, Wei C *et al* (2023) Loss of APOO (MIC26) aggravates obesity-related whitening of brown adipose tissue via PPARα-mediated functional interplay between mitochondria and peroxisomes. *Metabolism* 144: 155564

Harner M, Körner C, Walther D, Mokranjac D, Kaesmacher J, Welsch U, Griffith J, Mann M, Reggiori F, Neupert W (2011) The mitochondrial contact site complex, a determinant of mitochondrial architecture. *Embo j* 30: 4356-4370

Hofman DA, Ruiz-Orera J, Yannuzzi I, Murugesan R, Brown A, Clauser KR, Condurat AL, van Dinter JT, Engels SAG, Goodale A *et al* (2024) Translation of non-canonical open reading frames as a cancer cell survival mechanism in childhood medulloblastoma. *Mol Cell* 84: 261-276.e218

Huang N, Li F, Zhang M, Zhou H, Chen Z, Ma X, Yang L, Wu X, Zhong J, Xiao F *et al* (2021) An Upstream Open Reading Frame in Phosphatase and Tensin Homolog Encodes a Circuit Breaker of Lactate Metabolism. *Cell Metab* 33: 128-144.e129

Huynen MA, Mühlmeister M, Gotthardt K, Guerrero-Castillo S, Brandt U (2016) Evolution and structural organization of the mitochondrial contact site (MICOS) complex and the mitochondrial intermembrane space bridging (MIB) complex. *Biochim Biophys Acta* 1863: 91-101

Jayaram DR, Frost S, Argov C, Liju VB, Anto NP, Muraleedharan A, Ben-Ari A, Sinay R, Smoly I, Novoplansky O *et al* (2021) Unraveling the hidden role of a uORF-encoded peptide as a kinase inhibitor of PKCs. *Proc Natl Acad Sci U S A* 118

Kaser M, Kambacheld M, Kisters-Woike B, Langer T (2003) Oma1, a novel membrane-bound metallopeptidase in mitochondria with activities overlapping with the m-AAA protease. *J Biol Chem* 278: 46414-46423

Laube E, Schiller J, Zickermann V, Vonck J (2024) Using cryo-EM to understand the assembly pathway of respiratory complex I. *Acta Crystallogr D Struct Biol* 80: 159-173

Li Z, Shen W, Wu G, Qin C, Zhang Y, Wang Y, Song G, Xiao C, Zhang X, Deng G *et al* (2021) The role of SAMM50 in non-alcoholic fatty liver disease: from genetics to mechanisms. *FEBS Open Bio* 11: 1893-1906
Liang C, Padavannil A, Zhang S, Beh S, Robinson DRL, Meisterknecht J, Cabrera-Orefice A, Koves TR, Watanabe C, Watanabe M *et al* (2025) Formation of I(2)+III(2) supercomplex rescues respiratory chain defects. *Cell Metab* 37: 441-459.e411
Liu Z, Guo F, Zhu Y, Qin S, Hou Y, Guo H, Lin F, Chen PR, Fan X (2024) Bioorthogonal photocatalytic proximity labeling in primary living samples. *Nat Commun* 15: 2712
Makarewich CA, Baskin KK, Munir AZ, Bezprozvannaya S, Sharma G, Khemtong C, Shah AM, McAnally JR, Malloy CR, Szweda LI *et al* (2018) MOXI Is a Mitochondrial Micropeptide That Enhances Fatty Acid β-Oxidation. *Cell Rep* 23: 3701-3709
Makarewich CA, Munir AZ, Bezprozvannaya S, Gibson AM, Young Kim S, Martin-Sandoval MS, Mathews TP, Szweda LI, Bassel-Duby R, Olson EN (2022) The cardiac-enriched microprotein mitolamban regulates mitochondrial respiratory complex assembly and function in mice. *Proc Natl Acad Sci U S A* 119
Marlétaz F, Timoshevskaya N, Timoshevskiy VA, Parey E, Simakov O, Gavriouchkina D, Suzuki M, Kubokawa K, Brenner S, Smith JJ *et al* (2024) The hagfish genome and the evolution of vertebrates. *Nature* 627: 811-820
Ngo J, Choi DW, Stanley IA, Stiles L, Molina AJA, Chen PH, Lako A, Sung ICH, Goswami R, Kim MY *et al* (2023) Mitochondrial morphology controls fatty acid utilization by changing CPT1 sensitivity to malonyl-CoA. *Embo j* 42: e111901
Omenn GS, Lane L, Overall CM, Corrales FJ, Schwenk JM, Paik YK, Van Eyk JE, Liu S, Snyder M, Baker MS *et al* (2018) Progress on Identifying and Characterizing the Human Proteome: 2018 Metrics from the HUPO Human Proteome Project. *J Proteome Res* 17: 4031-4041
Parey K, Haapanen O, Sharma V, Köfeler H, Züllig T, Prinz S, Siegmund K, Wittig I, Mills DJ, Vonck J *et al* (2019) High-resolution cryo-EM structures of respiratory complex I: Mechanism, assembly, and disease. *Sci Adv* 5: eaax9484
Rampelt H, Wollweber F, Gerke C, de Boer R, van der Klei IJ, Bohnert M, Pfanner N, van der Laan M (2018) Assembly of the Mitochondrial Cristae Organizer Mic10 Is Regulated by Mic26-Mic27 Antagonism and Cardiolipin. *J Mol Biol* 430: 1883-1890
Rathore A, Chu Q, Tan D, Martinez TF, Donaldson CJ, Diedrich JK, Yates JR, 3rd, Saghatelian A (2018) MIEF1 Microprotein Regulates Mitochondrial Translation. *Biochemistry* 57: 5564-5575
Rensvold JW, Shishkova E, Sverchkov Y, Miller IJ, Cetinkaya A, Pyle A, Manicki M, Brademan DR, Alanay Y, Raiman J *et al* (2022) Defining mitochondrial protein functions through deep multiomic profiling. *Nature* 606: 382-388
Rocha AL, Pai V, Perkins G, Chang T, Ma J, De Souza EV, Chu Q, Vaughan JM, Diedrich JK, Ellisman MH *et al* (2024) An Inner Mitochondrial Membrane Microprotein from the SLC35A4 Upstream ORF Regulates Cellular Metabolism. *J Mol Biol* 436: 168559
Rocha AL, Schmedt C, Perkins G, Pinto A, Diedrich JK, Shan H, Plucińska K, Vieira de Souza E, Vaughan JM, Foster M *et al* (2025) Abnormal mitochondrial structure and function in brown adipose tissue of SLC35A4-MP knockout mice. *Sci Adv* 11: eads7381
Russ WP, Engelman DM (2000) The GxxxG motif: a framework for transmembrane helix-helix association. *J Mol Biol* 296: 911-919
Simcox J, Geoghegan G, Maschek JA, Bensard CL, Pasquali M, Miao R, Lee S, Jiang L, Huck I, Kershaw EE *et al* (2017) Global Analysis of Plasma Lipids Identifies Liver-Derived Acylcarnitines as a Fuel Source for Brown Fat Thermogenesis. *Cell Metab* 26: 509-522.e506

Smith JJ, Kuraku S, Holt C, Sauka-Spengler T, Jiang N, Campbell MS, Yandell MD, Manousaki T, Meyer A, Bloom OE *et al* (2013) Sequencing of the sea lamprey (Petromyzon marinus) genome provides insights into vertebrate evolution. *Nat Genet* 45: 415-421, 421e411-412

Smith JJ, Timoshevskaya N, Ye C, Holt C, Keinath MC, Parker HJ, Cook ME, Hess JE, Narum SR, Lamanna F *et al* (2018) The sea lamprey germline genome provides insights into programmed genome rearrangement and vertebrate evolution. *Nat Genet* 50: 270-277

Stein CS, Jadiya P, Zhang X, McLendon JM, Abouassaly GM, Witmer NH, Anderson EJ, Elrod JW, Boudreau RL (2018) Mitoregulin: A lncRNA-Encoded Microprotein that Supports Mitochondrial Supercomplexes and Respiratory Efficiency. *Cell Rep* 23: 3710-3720.e3718

Tang J, Zhang K, Dong J, Yan C, Hu C, Ji H, Chen L, Chen S, Zhao H, Song Z (2020) Sam50-Mic19-Mic60 axis determines mitochondrial cristae architecture by mediating mitochondrial outer and inner membrane contact. *Cell Death Differ* 27: 146-160

Vanderperre B, Lucier JF, Bissonnette C, Motard J, Tremblay G, Vanderperre S, Wisztorski M, Salzet M, Boisvert FM, Roucou X (2013) Direct detection of alternative open reading frames translation products in human significantly expands the proteome. *PLoS One* 8: e70698

Yang H, Li Q, Stroup EK, Wang S, Ji Z (2024) Widespread stable noncanonical peptides identified by integrated analyses of ribosome profiling and ORF features. *Nat Commun* 15: 1932

Zhang S, Guo Y, Fidelito G, Robinson DRL, Liang C, Lim R, Bichler Z, Guo R, Wu G, Xu H *et al* (2023) LINC00116-encoded microprotein mitoregulin regulates fatty acid metabolism at the mitochondrial outer membrane. *iScience* 26: 107558

Zhang S, Reljić B, Liang C, Kerouanton B, Francisco JC, Peh JH, Mary C, Jagannathan NS, Olexiouk V, Tang C *et al* (2020) Mitochondrial peptide BRAWNIN is essential for vertebrate respiratory complex III assembly. *Nat Commun* 11: 1312

1st Revision - Editorial Decision 24th Mar 2026

Dear Dr. Zhang

Thank you for the submission of your revised manuscript. We have now received the enclosed reports from referees 1 and 2 and I am happy to say that both support the publication of your study now. Only a few editorial requests will need to be addressed before we can proceed with the official acceptance of your manuscript:

- Please submit the main ms file without any figures. Only the main and EV figures legends need to be included.

- Please place the Disclosure and Competing Interest Statement after the Acknowledgments.

- The author credits need to be removed from the ms file. All credits need to be entered during online ms submission.

- This FUNDING INFO is missing in our online submission system: the ZJU100 Young Professor grant from School of Medicine, Zhejiang University and Zhejiang University Laboratory Animal Center. Please add.

- The 6 datasets are provided in a zip folder, however, each Dataset needs to be uploaded separately and the readme file with the legends is not needed as each legend needs to be (or already is) provided in a separate tab of each Excel file. Dataset EV3 has Dataset EV1 title/legend, please correct.

* Figure Legends - Comments *

- Please note that the exact p values are not provided in the legends of figures 1M, 2C, F, J, K; EV2 F, G, please provide exact values as reasonable.
- Please indicate the statistical test used for data analysis in the legends of figures 6L-N
- Please note that the box plots need to be defined in terms of minima, maxima, centre, bounds of box and whiskers, and percentile in the legends of figures 3G, J; EV3 D
- Please note that information related to n is missing in the legends of figures 1E, 6I, EV2 F; EV3 I, EV4 U
- Please note that the error bars are not defined in the legend of figure EV2 F

If I understand correctly, it currently remains unclear whether you can call the microprotein STREMI. This would need to be clarified before the official acceptance of this ms. If you chose this name, this should also be noted in the ms abstract.

EMBO press papers are accompanied online by A) a short (1-2 sentences) summary of the findings and their significance, B) 2-3 bullet points highlighting key results and C) a synopsis image that is exactly 550 pixels wide and 200-600 pixels high (the height is variable). The synopsis image should provide a sketch of the major findings, like a graphical abstract. Please note that text needs to be readable at the final size. Please send us this information along with the final manuscript.

I look forward to seeing a final version of your manuscript as soon as possible.

Best regards,
Esther

Esther Schnapp, PhD
Senior Editor
EMBO reports

Referee #1:

The authors have addressed my prior comments.

Referee #2:

The authors have made substantial efforts to address all reviewers' concerns and have significantly improved the manuscript. Especially my previous critique has been address fully and I have no additional concerns. I would like to congratulate the authors for their detailed answers and to a much improved paper.

The authors have addressed all minor editorial requests.

Shan Zhang
Zhejiang University School of Medicine
Department of Biochemistry, Department of Cardiology of The First Affiliated Hospital
China

Dear Dr. Zhang,

I am very pleased to accept your manuscript for publication in the next available issue of EMBO reports. Thank you for your contribution to our journal.

Your manuscript will be processed for publication by EMBO Press. It will be copy edited and you will receive page proofs prior to publication. Please note that you will be contacted by Springer Nature Author Services to complete licensing and payment information.

You may qualify for financial assistance for your publication charges - either via a Springer Nature fully open access agreement or an EMBO initiative. Check your eligibility: https://link.springer.com/journal/44319/how-to-publish-with-us

Should you be planning a Press Release on your article, please get in contact with embo_production@springernature.com as early as possible in order to coordinate publication and release dates.

If you have any questions, please do not hesitate to contact the Editorial Office. Thank you for your contribution to EMBO Reports.

Best regards,
Esther

Esther Schnapp, PhD
Senior Editor
EMBO reports

>>> Please note that it is EMBO Reports policy for the transcript of the editorial process (containing referee reports and your response letter) to be published as an online supplement to each paper. If you do NOT want this, you will need to inform the Editorial Office via email immediately. More information is available here: https://link.springer.com/partners/embo-press/editorial-policies#Peer%20review

EMBO Press Author Checklist

Corresponding Author Name: Shan Zhang	
Journal Submitted to: EMBO Reports	
Manuscript Number: EMBOR-2025-62854V2	

USEFUL LINKS FOR COMPLETING THIS FORM
The EMBO Journal - Author Guidelines
EMBO Reports - Author Guidelines
Molecular Systems Biology - Author Guidelines
EMBO Molecular Medicine - Author Guidelines

Reporting Checklist for Life Science Articles (updated January
This checklist is adapted from Materials Design Analysis Reporting (MDAR) Checklist for Authors. MDAR establishes a minimum set of requirements in transparent reporting in the life sciences (see Statement of Task: 10.31222/osf.io/9sm4x). Please follow the journal's guidelines in preparing your
Please note that a copy of this checklist will be published alongside your article.

Abridged guidelines for figures
1. Data
The data shown in figures should satisfy the following conditions:
- the data were obtained and processed according to the field's best practice and are presented to reflect the results of the experiments in an accurate and unbiased manner.
- ideally, figure panels should include only measurements that are directly comparable to each other and obtained with the same assay.
- plots include clearly labeled error bars for independent experiments and sample sizes. Unless justified, error bars should not be shown for technical
- if n<5, the individual data points from each experiment should be plotted. Any statistical test employed should be justified.
- Source Data should be included to report the data underlying figures according to the guidelines set out in the authorship guidelines on Data

2. Captions
Each figure caption should contain the following information, for each panel where they are relevant:
- a specification of the experimental system investigated (eg cell line, species name).
- the assay(s) and method(s) used to carry out the reported observations and measurements.
- an explicit mention of the biological and chemical entity(ies) that are being measured.
- an explicit mention of the biological and chemical entity(ies) that are altered/varied/perturbed in a controlled manner.
- the exact sample size (n) for each experimental group/condition, given as a number, not a range;
- a description of the sample collection allowing the reader to understand whether the samples represent technical or biological replicates (including how many animals, litters, cultures, etc.).
- a statement of how many times the experiment shown was independently replicated in the laboratory.
- definitions of statistical methods and measures:
  - common tests, such as t-test (please specify whether paired vs. unpaired), simple χ2 tests, Wilcoxon and Mann-Whitney tests, can be unambiguously identified by name only, but more complex techniques should be described in the methods section;
  - are tests one-sided or two-sided?
  - are there adjustments for multiple comparisons?
  - exact statistical test results, e.g., P values = x but not P values < x;
  - definition of 'center values' as median or average;
  - definition of error bars as s.d. or s.e.m.

Please complete ALL of the questions below.
Select "Not Applicable" only when the requested information is not relevant for your study.

Materials

Newly Created Materials	Information included in the manuscript?	In which section is the information available? (Reagents and Tools Table, Materials and Methods, Figures, Data Availability Section)
New materials and reagents need to be available; do any restrictions apply?	Yes	Reagents and Tools Table

Antibodies	Information included in the manuscript?	In which section is the information available? (Reagents and Tools Table, Materials and Methods, Figures, Data Availability Section)
For **antibodies** provide the following information:<br>- Commercial antibodies: RRID (if possible) or supplier name, catalogue number and or/clone number<br>- Non-commercial: RRID or citation	Yes	Reagents and Tools Table

DNA and RNA sequences	Information included in the manuscript?	In which section is the information available? (Reagents and Tools Table, Materials and Methods, Figures, Data Availability Section)
**Short novel DNA or RNA including primers, probes**: provide the sequences.	Yes	Reagents and Tools Table

Cell materials	Information included in the manuscript?	In which section is the information available? (Reagents and Tools Table, Materials and Methods, Figures, Data Availability Section)
**Cell lines:** Provide species information, strain. Provide accession number in repository **OR** supplier name, catalog number, clone number, and/**OR** RRID.	Yes	Reagents and Tools Table
**Primary cultures:** Provide species, strain, sex of origin, genetic modification status.	Not Applicable	
Report if the cell lines were recently **authenticated** (e.g., by STR profiling) and tested for mycoplasma contamination.	Yes	Reagents and Tools Table

Experimental animals	Information included in the manuscript?	In which section is the information available? (Reagents and Tools Table, Materials and Methods, Figures, Data Availability Section)
**Laboratory animals or Model organisms:** Provide species, strain, sex, age, genetic modification status. Provide accession number in repository **OR** supplier name, catalog number, clone number, **OR** RRID.	Yes	Reagents and Tools Table
**Animal observed in or captured from the field:** Provide species, sex, and age where possible.	Not Applicable	
Please detail **housing and husbandry conditions**.	Yes	Materials and Methods

Plants and microbes	Information included in the manuscript?	In which section is the information available? (Reagents and Tools Table, Materials and Methods, Figures, Data Availability Section)
**Plants:** provide species and strain, ecotype and cultivar where relevant, unique accession number if available, and source (including location for collected wild specimens).	Not Applicable	
**Microbes:** provide species and strain, unique accession number if available, and source.	Yes	Reagents and Tools Table

Human research participants	Information included in the manuscript?	In which section is the information available? (Reagents and Tools Table, Materials and Methods, Figures, Data Availability Section)
If collected and within the bounds of privacy constraints report on age, sex and gender or ethnicity for all study participants.	Not Applicable	

Core facilities	Information included in the manuscript?	In which section is the information available? (Reagents and Tools Table, Materials and Methods, Figures, Data Availability Section)
If your work benefited from core facilities, was their service mentioned in the acknowledgments section?	Yes	Acknowledgments

Design

Study protocol	Information included in the manuscript?	In which section is the information available? (Reagents and Tools Table, Materials and Methods, Figures, Data Availability Section)
If study protocol has been **pre-registered, provide DOI in the manuscript**. For clinical trials, provide the trial registration number **OR** cite DOI.	Not Applicable	
Report the **clinical trial registration number** (at ClinicalTrials.gov or equivalent), where applicable.	Not Applicable	

Laboratory protocol	Information included in the manuscript?	In which section is the information available? (Reagents and Tools Table, Materials and Methods, Figures, Data Availability Section)
Provide DOI OR other citation details if **external detailed step-by-step protocols** are available.	Yes	Materials and Methods, References

Experimental study design and statistics	Information included in the manuscript?	In which section is the information available? (Reagents and Tools Table, Materials and Methods, Figures, Data Availability Section)
Include a statement about **sample size** estimate even if no statistical methods were used.	Yes	Figure legend
Were any steps taken to minimize the effects of subjective bias when allocating animals/samples to treatment (e.g. **randomization procedure**)? If yes, have they been described?	Not Applicable	
Include a statement about **blinding** even if no blinding was done.	Yes	Materials and Methods
Describe **inclusion/exclusion criteria** if samples or animals were excluded from the analysis. Were the criteria pre-established?<br><br>If sample or data points were omitted from analysis, report if this was due to attrition or intentional exclusion and provide justification.	Not Applicable	
For every figure, are **statistical tests** justified as appropriate? Do the data meet the assumptions of the tests (e.g., normal distribution)? Describe any methods used to assess it. Is there an estimate of variation within each group of data? Is the variance similar between the groups that are being statistically compared?	Yes	Figure legend, Quantification and statistical analysis

Sample definition and in-laboratory replication	Information included in the manuscript?	In which section is the information available? (Reagents and Tools Table, Materials and Methods, Figures, Data Availability Section)
In the figure legends: state number of times the experiment was **replicated** in laboratory.	Yes	Figure legend
In the figure legends: define whether data describe **technical or biological replicates**.	Yes	Figure legend

Ethics

Ethics	Information included in the manuscript?	In which section is the information available? (Reagents and Tools Table, Materials and Methods, Figures, Data Availability Section)
Studies involving **human participants**: State details of **authority granting ethics approval** (IRB or equivalent committee(s), provide reference number for approval.	Not Applicable	
Studies involving **human participants**: Include a statement confirming that **informed consent** was obtained from all subjects and that the experiments conformed to the principles set out in the WMA Declaration of Helsinki and the Department of Health and Human Services Belmont Report.	Not Applicable	
Studies involving **human participants:** For publication of **patient photos**, include a statement confirming that consent to publish was obtained.	Not Applicable	
Studies involving experimental **animals**: State details of **authority granting ethics approval** (IRB or equivalent committee(s), provide reference number for approval. Include a statement of compliance with ethical regulations.	Yes	Materials and Methods
Studies involving **specimen and field samples:** State if relevant **permits** obtained, provide details of authority approving study; if none were required, explain why.	Not Applicable	

Dual Use Research of Concern (DURC)	Information included in the manuscript?	In which section is the information available? (Reagents and Tools Table, Materials and Methods, Figures, Data Availability Section)
Could your study fall under dual use research restrictions? Please check biosecurity documents and list of **select agents and toxins** (CDC): https://www.selectagents.gov/sat/list.htm	Not Applicable	
If you used a select agent, is the security level of the lab appropriate and reported in the manuscript?	Not Applicable	
If a study is subject to dual use research of concern regulations, is the name of the **authority granting approval and reference number** for the regulatory approval provided in the manuscript?	Not Applicable	

Reporting

The MDAR framework recommends adoption of discipline-specific guidelines, established and endorsed through community initiatives. Journals have their own policy about requiring specific guidelines and recommendations to complement MDAR.

Adherence to community standards	Information included in the manuscript?	In which section is the information available? (Reagents and Tools Table, Materials and Methods, Figures, Data Availability Section)
State if relevant guidelines or checklists (e.g., **ICMJE, MIBBI, ARRIVE, PRISMA**) have been followed or provided.	Not Applicable	
For **tumor marker prognostic studies,** we recommend that you follow the **REMARK** reporting guidelines (see link list at top right). See author guidelines, under 'Reporting Guidelines'. Please confirm you have followed these guidelines.	Not Applicable	
For **phase II and III randomized controlled trials**, please refer to the **CONSORT** flow diagram (see link list at top right) and submit the CONSORT checklist (see link list at top right) with your submission. See author guidelines, under 'Reporting Guidelines'. Please confirm you have submitted this list.	Not Applicable	

Data Availability

Data availability	Information included in the manuscript?	In which section is the information available? (Reagents and Tools Table, Materials and Methods, Figures, Data Availability Section)
Have **primary datasets** been deposited according to the journal's guidelines (see 'Data Deposition' section) and the respective accession numbers provided in the Data Availability Section?	Yes	Data Availability Section
Were **human clinical and genomic datasets** deposited in a public access-controlled repository in accordance to ethical obligations to the patients and to the applicable consent agreement?	Yes	Data Availability Section
Are **computational models** that are central and integral to a study available without restrictions in a machine-readable form? Were the relevant accession numbers or links provided?	Not Applicable	
If publicly available data were reused, provide the respective **data citations in the reference list**.	Yes	References
